# Is soil management system really important? comparison of microbial community diversity and structure in soils managed under organic and conventional regimes with some view on soil properties

**DOI:** 10.1371/journal.pone.0256969

**Published:** 2021-09-09

**Authors:** Kamila Rachwał, Klaudia Gustaw, Waldemar Kazimierczak, Adam Waśko

**Affiliations:** 1 Department of Biotechnology, Microbiology and Human Nutrition, Faculty of Food Science and Biotechnology, University of Life Sciences in Lublin, Lublin, Poland; 2 Department of Biotechnology and Environmental Sciences, John Paul II Catholic University of Lublin, Lublin, Poland; South China Agricultural University, CHINA

## Abstract

The fertility and productive value of soil are closely related to the physical and chemical properties of the soil as well as its biological activity. This activity is related to the intensity of microbially catalysed processes of transformation of organic and mineral substances contained in the soil. These processes are closely correlated with the abundance and biodiversity of soil microorganisms, especially bacteria, and the activity of enzymes produced by them. In this article we have compared some physicochemical properties of soil derived from conventional and organic farms and microbial communities inhabiting these ecosystems. We aim to investigate whether the soil management regime affects the abundance and diversity of these environments in terms of bacteria. Some differences in microbial communities were observed, but the rhizosphere of plants from organic and conventional soils does not harbour separate microbiomes. Albeit, the method of fertilization influences the diversity of soil microorganisms. A greater diversity of bacteria was observed in soils from farms where organic fertilizers were applied. Soil pH and activity of some soil enzymes were also shown to differ between organic and conventional soil cropping systems.

## Introduction

Agroecosystems are defined as natural ecosystems which have been altered by humans to produce food, fibre, fuel and other products [1]. Although they retain many of the features of natural ecosystems, they are also characterised by the frequent occurrence of agrochemicals and associated changes in biodiversity [2]. The extent of soil changes caused by cultivation, including alterations in biodiversity, is closely related to the type and intensity of soil exploitation. The soil management system is therefore a one of the cornerstones of farming and key factor affecting soil functions. This is extremely relevant because soil provides basic ecosystem services, including nutrient cycling, water regulation, processing of organic materials and toxic compounds, pest and disease control [3].

Soil properties are largely determined by agronomic practices [4]. The soil management system has a direct impact on soil fertility and yields by influencing the physical, chemical and biological properties of the soil [5, 6]. One of the important soil characteristic that can vary depending on the cropping system is bulk density. This is a very variable feature, determined by the degree of arrangement of the soil grains [7]. The bulk density reflects the ability of the soil to function as a structural element [8]. High soil bulk density inhibits root development, impedes and limits plant uptake of water and nutrients, and reduces fertilizer efficiency [9]. The acid-base properties of the soil are another very important physicochemical characteristic of the soil, which have a significant impact on soil processes, nutrient assimilation, plant and microbial development. Soil pH directly determines the direction and speed of biological and physicochemical processes in soils [10]. Optimal pH ensures adequate nutrition of plants and their good growth. Furthermore, the concentration of H^+^ ions in the soil environment affects its enzymatic activity. Through altering solubility and ionisation, pH influences enzyme dynamics, substrate degradation and cofactor attachment [11].

Soil enzymes are essential for catalysing many biogeochemically significant reactions. These molecules may be bound to living cells (inside or on their surface), secreted by them into the environment, or form a complex with the soil matrix [12]. Enzyme activity is an indicator of the biological state of the soil. Enzymatic processes are very sensitive to changes in this environment driven by natural and anthropogenic factors related to soil management and its physical and chemical properties [13]. Enzymes participate in soil nutrient cycling, stabilisation of soil structure, mineral release and delivery to plants, nitrogen fixation and degradation of pollutants. Enzyme activity is closely associated with soil organisms, especially microorganisms [14]. Enzymes secreted by the bacteria enable the utilization of available sources of N, P, and K, which in the case of rhizosphere-inhabiting bacteria also has a beneficial effect on plant growth [15].

In addition to the above mentioned changes in physical and chemical properties, the soil management system also affects the diversity and abundance of soil microorganisms. Soil microbial diversity is an significant indicator of the biological stability of soils and the intensity of biochemical processes occurring in the soil [16]. At a system level, the microbiome plays an integral role in virtually all soil processes [17]. Bacteria are considered as early warning indicators of soil quality due to their rapid response and sensitivity to environmental changes [18, 19]. It was previously reported that the presence of bacteria in agroecosystems can be influenced by agronomic practices such as tillage, irrigation and use of agrochemicals [20]. Soil microbial structure can be disturbed by repeated application of chemical fertilizers. Also organic fertilizers (such as animal manure, composted organic matter) can alter the structure and activity of soil bacterial community [21]. The abundance, activity and composition of microorganisms largely determine the sustainable productivity of agricultural land. Thus, the ability to manage the soil microbiome to influence the presence of beneficial and non-beneficial organisms may be a promising approach to improve sustainable agricultural production. Considering the integral role of the soil microbiome in agricultural production, the diversity of soil bacterial communities can serve as a biodiagnostic tool with great potential. However, the high complexity and diversity of soil microorganisms and the technical limitations of the proper measurement of their composition have so far limited our understanding of the relationship between soil management system and microbial diversity. New high-throughput DNA sequencing technologies offer the means to study soil microorganisms with greater resolution, coverage and efficiency, and can shed more light on how microbial communities respond to different agricultural systems [20]. The development of the field of metagenomics allows a more accurate understanding of the structure of microbial communities in samples of different nature, including soils. Recently, it has become an important tool for assessing the diversity of microbial communities. It allows the identification of bacteria at different taxonomic levels, which is particularly important in the study of soil environments that harbour unique and diverse microorganisms.

In order to successfully identify a beneficial consortium of soil bacteria, it is important to have a deeper understanding of how the community structure of these microorganisms changes in response to different agricultural practices. Studies of the soil microbiome under different fertilization conditions have already been undertaken, but these field experiments were conducted under strictly controlled conditions. This allowed a fair comparison of the effect of the cropping system itself on soil microbial communities, but it is unclear if this is applicable to the much more variable and random conditions encountered on actual farms. We therefore decided to compare soil samples from conventionally and organically managed farmsteads. The main objective was to analyse the microbial community, in terms of its number and diversity of bacteria inhabiting the rhizosphere of plants, and to examine some properties of the soil that may also be altered by different soil management practices. Therefore, this study aim to compare the effects of conventional and organic soil management systems on the complexity of *Daucus carrota* rhizosphere microbial communities, soil biological activity and selected physicochemical properties of soil.

## Materials and methods

### Rhizospheric soil sample collection

Soil samples were collected from the rhizosphere of carrot (*Daucus carota* L.). Soil samples directly adjacent to the plant roots were collected. Samples were obtained from ten different locations in Lubelskie Voivodeship, in the east of Poland (average annual temperature 8.9°C, precipitation 750 mm). Details of sampling locations can be found in the (S1 Map). Five of the soil samples came from organic farms, where natural fertilizers such as compost and manure were used (samples E1-E5). The applied compost concentration was 5 L/m^2^, while the manure concentration was 50 m^3^/ha. Another five samples was derived from conventional agriculture systems, where mainly mineral fertilizers were used (samples K1 –K5). Samples were obtained from farms where the following fertilisers were applied: K1—ammonium nitrate (at a concentration of 32 kg/ ha) with combination of manure (30 m^3^/ha), K2 and K3 ammonium nitrate (80 kg/ha and 100 kg/ha, respectively), K4 –ammonium nitrate (100 kg/ha) and fertilizer with mineral complex containing nitrogen, phosphorus, potassium and sulphur (2 kg/ha), K5—fertilizer with mineral complexes containing nitrogen, phosphorus, potassium, magnesium, sulphur, boron, manganese and zinc (420 kg/ha). At the time of sampling from the rhizosphere all the carrots were in the maturation phase. The research was approved by the Voivodship Fund for Environmental Protection and Water Management. Since the samples were obtained from farms, not experimental fields, no permits were required to conduct this work.

### Determination of bacterial and fungal abundance in soil samples

The count of soil microorganisms in the individual samples was determined using the plate count method, in 3 replicates. 1 g of soil was weighed and poured into a flask containing 9 ml of saline and the mixture was shaken for a few minutes in order to wash out microorganisms from soil particles. It was allowed to wait until the solids had fallen to the bottom. Serial dilutions of soil (10^−1^–10^−6^) were prepared. The soil solution was treated as a 10^−1^ dilution. 0.1 ml of each of the dilutions of bacterial suspensions (from 10^−2^ to 10^−6^) was distributed with a glass spreader on two plates with nutrient agar (for bacteria) or PDA (for fungi). After three days of incubation at room temperature the colonies were counted and then the number of microbial units capable of forming colonies (CFU) in 1 g of soil was determined using the formula: CFU/g of soil = average number of colonies per dilution* dilution factor * volume of inoculum (ml)^-1^.

### Extraction and purification of genomic DNA from soil samples

A direct method of DNA extraction from the soil was applied. DNA was extracted from the 250 mg of soil samples of the carrot rhizosphere using commercially available GeneMATRIX Soil DNA Purification Kit (EURx, Gdańsk, Poland). DNA was isolated from each soil sample in duplicate and both replicates were used for further analysis. DNA isolation was conducted according to a manufacturer instruction. DNA quantity and quality of each sample were determined using NanoDrop1000 spectrophotometer (Thermo Fisher Scientific, C.A., USA).

### DNA new generation sequencing

PCR amplifications were conducted using primer sets that encompass the V3 and V4 hypervariable regions of 16S rRNA gene which enable the analysis of taxonomic groups of both bacteria and archaea. For amplification of the selected region and preparation of the library, primers 341F (5’-CCTACGGGNGGCWGCAG-3’) and 785R (5’-GACTACHVGGGTATCTAATCC-3’) were used. The amplicons were prepared on samples of isolated DNA in two-stage PCR. The PCR reaction was carried out using Q5 Hot Start High-Fidelity 2X Master Mix (New England BioLabs, Ipswich, MA, United States) under conditions in accordance with the manufacturer’s recommendations. Sequencing was performed using Illumina MiSeq platform (Illumina, San Diego, CA, USA) in paired end mode in two readings of 250 base pairs. Automatic primary analysis of the data was carried out on the MiSeq device using MiSeq Reporter (MSR) v2.6 software. The analysis consisted of automatic sample demultiplexing and generating of fastq files containing raw readings.

### Accession numbers

Data obtained from metagenomic sequencing were deposited in the Sequence Read Archive (Project ID PRJNA753303).

### Sequencing data analysis

The bioinformatic analysis, providing classification of readings to species level, was conducted with the QIIME software package based on GreenGenes v13_8 reference sequence database [22, 23]. The analysis included the removal of adaptive sequences, analysis of reading quality and removal of low-quality sequences (quality < 20, minimum length 30) in the cutadapt program [24]. Next, paired sequences using fastq-join algorithm were combined, clustering was performed based on selected base of reference sequences with unclust algorithm and sequence chimeras were removed with usearch61 algorithm. Taxonomy was assigned to base of reference sequences using unclust algorithm [25, 26]. Sequences showing >97% similarity were clustered at the species level, forming an operational taxonomic unit (OTU). The analyses resulted in the construction of OTU tables containing the number of sequences observed for each taxonomic unit (OTU) in all samples. Krona charts were built with these taxonomic data for all the samples [27].

### Microbial community analysis

16S rRNA gene sequecing data processing was performed using Galaxy Platform (https://usegalaxy.org/). Data were organisesed in paired collection. Illumina paired-end reads were analyzed using the Mothur software (v1.3.3.0) following MiSeq standard operating procedure with minor modifications [28]. Paired reads were assembled into contigs. Quality control was performed, reads were filtered based on quality and length. Sequences were aligned to SILVA bacteria reference database [29]. Unique sequences were screened and pre-clustering was performed. Chimera sequences were found and removed. Non-bacterial sequences were also removed. Sequences were clustered into OTUs with cutoff of 0.03. Rarefaction curves at a 97% sequence similarity cutoff value were constructed. Simpson and Shannon indices of diversity and richness estimators such as abundance-based coverage estimator (ACE) and Chao1 index were calculated based on OTUs using Mothur [30–33]. Also heatmaps were generated with Mothur using Bray-Curtis dissimilarity for sample clustering. Unweighted pair group method with arithmetic mean (UPGMA) tree and principal coordinate analysis (PCoA) performed in PAST software were used to visualize relationships between samples [34].

### Determination of soil enzymatic activities

Enzyme activities were measured since their changes in the rhizosphere zone reflect environmental disturbances affecting both soil and plants. They are related to the transformation of elements in the soil, resulting mainly from the activity of microorganisms. Acid phosphatase (EC 3.1.3.2) and β-glucosidase (EC 3.2.1.21) activities was evaluated using appropriate substrates (*p-*nitrophenyl phosphate and nitrophenyl-β-glucopyranoside, respectively) [35]. Enzyme assays were performed for each soil sample in triplicate. In addition, control samples were prepared (with no substrate added). The soil samples was mixed with 50 mM acetate buffer (pH 5.0) to obtain solution 1 g * ml^-1^. 150 μl of soil suspension was mixed with 150 μl 5 mM substrate solution (in case of tested samples) or with 50 mM acetate buffer (in control samples) and incubated in room temperature (22˚C) for 1 h. After incubation samples were centrifuged (5 min, 5000 rpm) and 100 μl of supernatant was transferred to a new eppendorf tube containing 200 μl of 0.05 M NaOH. Absorbance of each sample was measured at 410 nm. Final absorbance of each sample was calculated by substracting the sample control absorbance. A calibration curve was prepared for p-nitrophenol (pNP) solutions (in 50 mM acetate buffer) in range from 0.025 mM to 1 mM. Enzymatic activity of acid phosphatase and β-glucosidase was calculated as μmoles of product released during reaction using equation:

$$Enzyme\;activity\;(\mu moles\;pNP*h^{-1}*{g\;dry\;mass}^{-1})=\frac{final\;absorbance}{C*t(h)*m(g)},\;\mathrm{where}:$$

t- incubation time (1 h), m–dry mass of soil sample (g), C—a slope of a pNP standard curve.

The activity of urease (EC 3.5.1.5) was assayed according to method developed by Kandeler and Gerber [36]. This method is based on urea hydrolysis into the ammonia, which amount is further determined. Briefly, this method was based on mixing of 1 g (each sample in triplicates) with 500 μl of 80 mM urea and further incubation of samples at 18°C for 2 h. Subsequently, 10 ml of 2 M KCl was added and soil samples were extracted for 30 min with shaking (300 rpm). After extraction, samples were centrifuged for 5 min at 2900 x *g* and filtered through Whatman filter paper. 0.5 ml of a clear filtrate was mixed with 2.5 ml of distilled water and 1.5 ml 0.1 M phosphate buffer pH 7.6. The amount of ammonia was evaluated in the sample using Nessler’s reagent. The content of the tube was mixed on vortex, 0.5 ml of Nessler’s reagent was added, the tubes were sealed and vortexed again. A reagent sample with 0.5 ml of the washing solution instead of the examined sample was prepared. Samples were incubated for 15 min and the absorbance was measured at 436 nm by setting the spectrophotometer zero on the reagent sample. A calibration curve was prepared using (NH_4_)_2_SO_4_ standard solution in concentrations of 0.01 mg * ml^-1^–0.1 mg * ml^-1^. Urease activity was calculated according to the formula:

$$Urease\;activity\;(\mathrm{mg}\;N‐{\mathrm{NH}}_{4}^{+}*h^{‐1}*g^{‐1}\;soil)=\frac{(S-C)*v(ml)*100}{t(h)*m(g)*\%\mathrm{dry}\;\mathrm{mass}},\;\mathrm{where}:$$

S–amount of N in 1 ml of filtrate of the test sample (mg)

C—amount of N in 1 ml of filtrate of control sample (mg)

v–volume of filtrate (1,5 ml)

t–incubation time (2 h)

m—weight of the wet soil (0,1 g)

100 * %^-1^ d.m.—conversion factor to dry matter of the sample

Glutaminase (EC 3.5.1.2) activity was measured according to Omura and co-workers [37]. In this assay, as a result of the L-glutamine deamination, ammonia is formed, the amount of which can be determined in reaction with Nessler’s reagent. Briefly, 0.1 g of each sample (in triplicates) was mixed with 50 μl of toluene and sealed tightly. After 5 min, 500 μl of the substrate solution (0.25 M L-glutamine in 0.1 M phosphate buffer pH 7.6) was added, the content was mixed and placed in a thermostat at 28˚C for 23 h. One control with distilled water instead of substrate was also prepared for each soil sample. After incubation, 30 μl 5 M HCl solution was added. After next 5 min washing solution (0.5 M KCl in 0.2 M NaOH) was added, samples were mixed vigorously and shaken for 10 min at 30 rpm. The samples were centrifuged (5 min, 4 000 rpm) and filtered through Whatman filter paper. 0.5 ml of a clear filtrate was mixed with 2.5 ml of distilled water and 1.5 ml 0.1 M phosphate buffer pH 7.6. The content of ammonia in the samples was evaluated using Nessler’s reagent, as described above. Amount of ammonia in samples were calculated using calibration curve using formula:

$${Enzyme\;activity\;(mg\;N*{g\;dry\;mass}^{-1}*h}^{-1}=\frac{(S-C)*v*100}{t(h)*m(g)*\%d.m.}$$

Protease (EC 3.4.4) activity was measured using sodium caseinate as a substrate (modified method developed by Ladd and Butler [38]). 0.1 g soil was mixed with 1.25 ml of 2% substrate solution and 1.5 ml of 50 mM Tris buffer pH 8.1. Each sample was prepared in triplicate and with one control sample without a substrate. Samples were incubated in 50˚C for 1 h and after this time cooled to 20˚C. 1 ml of 15% trichloroacetic acid was added, samples were mixed and centrifuged (10 min, 10 000 rpm). From each sample 1 ml of supernatant was mixed with 1.5 ml 2.8 N sodium carbonate and 0.5 ml Folin Reagent. After 10 min of incubation an absorbance of samples was measured at 700 nm by setting the spectrophotometer zero on the reagent sample (Tris buffer added instead of supernatant from samples). The results were calculated from the calibration curve prepared using a set of tryptophan solutions with concentrations ranging from 0.01 mg/ml– 0.15 mg/ml. Enzyme activity was calculated from the calibration curve using the formula:

$$Enzyme\;activity\;(\mu g\;Tyr*{{g\;dry\;mass}^{-1}*h}^{-1}=\frac{(S-C)*100}{t(h)*m(g)*\%d.m.},\;\mathrm{where}:$$

S–the tyrosine content of the test sample read from the standard curve (μg)

C—the tyrosine content of the control sample read from the standard curve (μg)

t–incubation time (h)

m—weight of the wet soil (0.1 g)

100 * %^-1^ d.m.—conversion factor to dry matter of the sample.

### Soil properties

In the soil samples, the pH value was determined by potentiometric method. The active (current) acidity, caused by the presence of hydrogen ions in pore solution, was determined by measuring the pH of the soil suspension in distilled water. The potential acidity (exchangeable, caused by exchangeable H^+^ and Al^3+^ ions, which are poorly bound by the soil sorption complex) was determined by measuring the soil pH in 1 M KCl solution. For each sample the measurement was carried out in three repetitions. The average of the three measurements was taken as the result.

Also the bulk density of soils was determined. For this purpose, 10 ml of air-dry soil was measured with a measuring cylinder. The prepared sample was weighed and the volumetric density of the soil was calculated as the ratio of the wet soil mass to the soil sample volume. It was calculated using a formula:

$\rho =\frac{m}{v}$, where: m is a weight of soil sample [g] and v is soil density by volume [g/ cm^3^].

The last soil property determined in the study was the water content of the soil, measured on the basis of mass. To determine the gravimetric soil water content, weights of wet soil of known mass were prepared, dried at 105°C for 4 h (until the soil samples were completely dry) and then weighed again (oven-dry weight). The water content of the soil was calculated according to the equation: The gravimetric soil water content (%) = (mass of moist soil [g]–mass of oven-dried soil [g]/ mass od oven-dried soil [g]) * 100.

### Statistical analysis

The Statistica software v. 13.3 (StatSoft, Tulsa, OK, United States) was used to determine whether differences in soil physio-chemical properties, the microbial diversity and richness between samples derived from organic and conventional farms were statistically significant. Student’s t-test was used to determine the statistical significance of the differences observed.

## Results

### Soil properties

It was observed that in conventionally managed soils, the mean soil pH was slightly lower (6.13 ± 0.37 in H_2_O and 4.87 ± 0.64 in KCl) than in organically managed soils (7.08 ± 0.52 in H_2_O and 5.91 ± 0.72 in KCl) (Table 1). According to conventional criteria, the difference between the types of soil studied was considered statistically significant (*p* <0.05). The soils from conventional agriculture system were from slightly acidic to acidic. On the other hand, the substantial majority of soils obtained from organic farms were neutral, only one of the samples had a slightly acidic pH value. The lowest soil pH was observed in samples K4 and K5 from conventional farms.

**Table 1:** pH values of soils from covnventional and organic farming systems, determined in water and potassium chloride.

Soil management system	Sample ID	Average pH_water_	Average pH _KCl_	Soil type
**Organic**	E1	7.29	6.72	Neutral
	E2	6.10	4.72	Slightly acidic
	E3	7.04	6.12	Neutral
	E4	7.46	6.50	Neutral
	E5	7.52	6.48	Neutral
**Conventional**	K1	6.81	5.94	Slightly acidic
	K2	6.12	5.14	Slightly acidic
	K3	6.08	4.44	Medium acidic
	K4	5.67	4.06	Acidic
	K5	5.97	4.76	Medium acidic

Another of the specified parameters concerning soil properties was its bulk density. The average bulk density of the samples tested ranged from 0.761 cm^3^ to 1.246 cm^3^ (Table 2). The study showed that the conventionally and organically-farmed soils had similar values of this parameter and all were within the the range of values suitable for plant growth. Therefore, no significant effect of soil management on soil bulk density was observed in case of the tested samples.

**Table 2:** Average soil water content and bulk density in the tested soil samples.

Soil management system	Sample ID	Soil water content (SWC) %	Soil bulk density [cm^3^]
**Organic**	**E1**	21.54	0.99
	**E2**	12.98	0.881
	**E3**	26.58	1.159
	**E4**	20.21	0.879
	**E5**	19.29	1.133
**Conventional**	**K1**	15.22	0.816
	**K2**	17.74	0.884
	**K3**	17.33	0.761
	**K4**	17.65	1.246
	**K5**	4.36	1.05

The measurement of soil water content (SWC) revealed some differences between the tested soil samples from organic and conventional farms. The SWC of the samples ranged between 4.35% and 21.53% (Table 2). Differences between the dry weight of soil samples may depend on the soil type itself, but are most likely also related to the soil management system. In most samples obtained from conventional farms, the soil water content was lower than in samples from organic farms although differences were not statistically significant (*p* > 0.05).

### Bacterial and fungal abundance in soil samples

**Fig 1:**
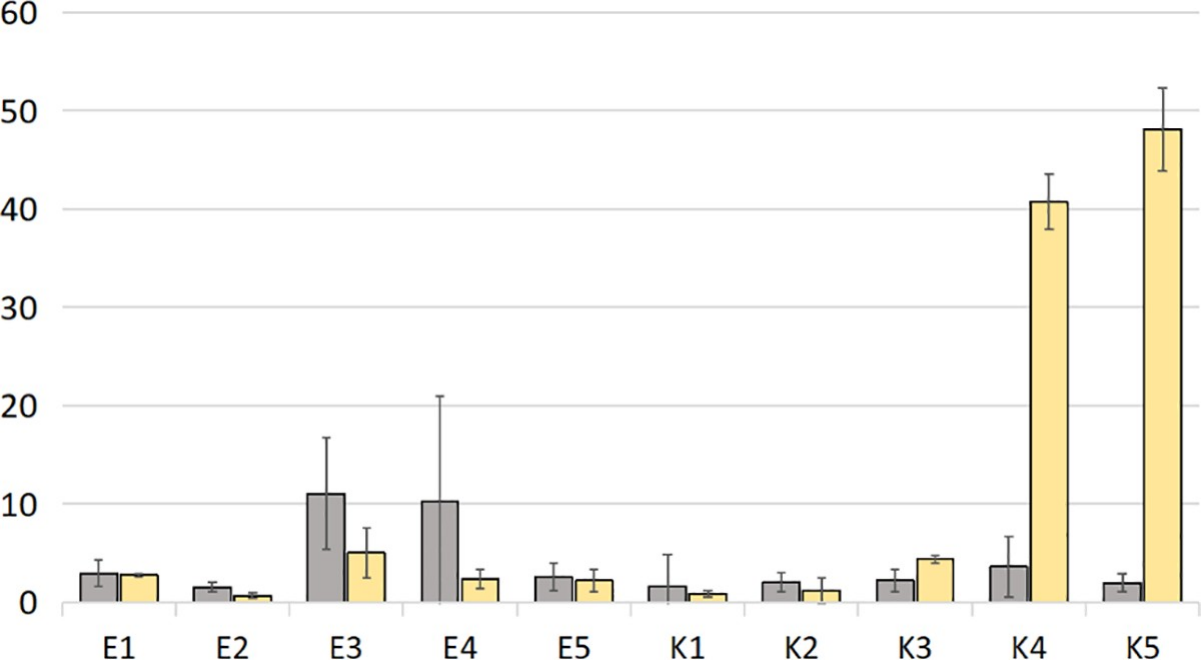
Bacterial and fungal abundance in tested soil samples. The grey bars indicate the amount of bacteria [CFU g^-1^ of soil] x 10^7^ and yellow bars indicate the amount of fungi [CFU/ 1 g of soil] x 10^6^.

Soil samples were diluted and seeded on agar plates for bacterial and fungal growth. Different colony morphology of microorganisms on agar plates were observed. CFU counts for total bacteria and fungi shown significant differences between the amount of these microorganisms found in samples from organic and conventional farms (Fig 1).

All samples from organic farms contained more bacterial than fungal cells. Their amount in these samples ranged from 1.5 x 10^7^ to 11.04 x 10^7^ (mean = 5.65 ± 4.11). In contrast, in samples from conventional farms bacterial cell count was between 1.6 x 10^7^ and 3.6 x 10^7^ (mean = 2.27 ± 0.69). Moreover, two out of 5 samples derived from conventional farms were more abundant in fungi than bacteria (Fig 1). In K4 and K5 amount of bacteria was 3.6 x 10^7^ and 1.96 x 10^7^, while number of fungi was estimated as 4.7 x 10^7^ and 4.8 x 10^7^, respectively. The average amount of fungi in the organic samples was 2.58 x 10^6^ (± 1.4), while in the conventional samples 19.04 x 10^6^ (± 20.87).

### Structure of the soil bacterial communities

Metagenomic analyses allowed us to compare bacterial OTUs in soil samples with the aim of identifying differences between samples from organic and conventional farms. DNA was isolated for each soil sample in duplicate. Two independent DNA samples were prepared for each of the soils included in the study (E1.1—E5.2 from organic and K1.1—K5.2 from conventional). As a result of the NGS analysis an average of 90094.6 (range from 54981 to 116237) reads per sample were classified into OTUs. Summarising all the samples analysed, the study identified 45 phyla, 146 classes, 288 orders, 429 families, 660 genera and 709 species belonging to both bacteria and archea. An average of 90085 (76%) reads were assigned at type level and class level. At lower taxonomic levels an average of 89384 (75.4%), 78954 (66.6%), 51421 (43.5%), 21209 (17.8%) and 2432 (2%) were assigned at the class, order, family, genus and species level, respectively.

**Fig 2:**
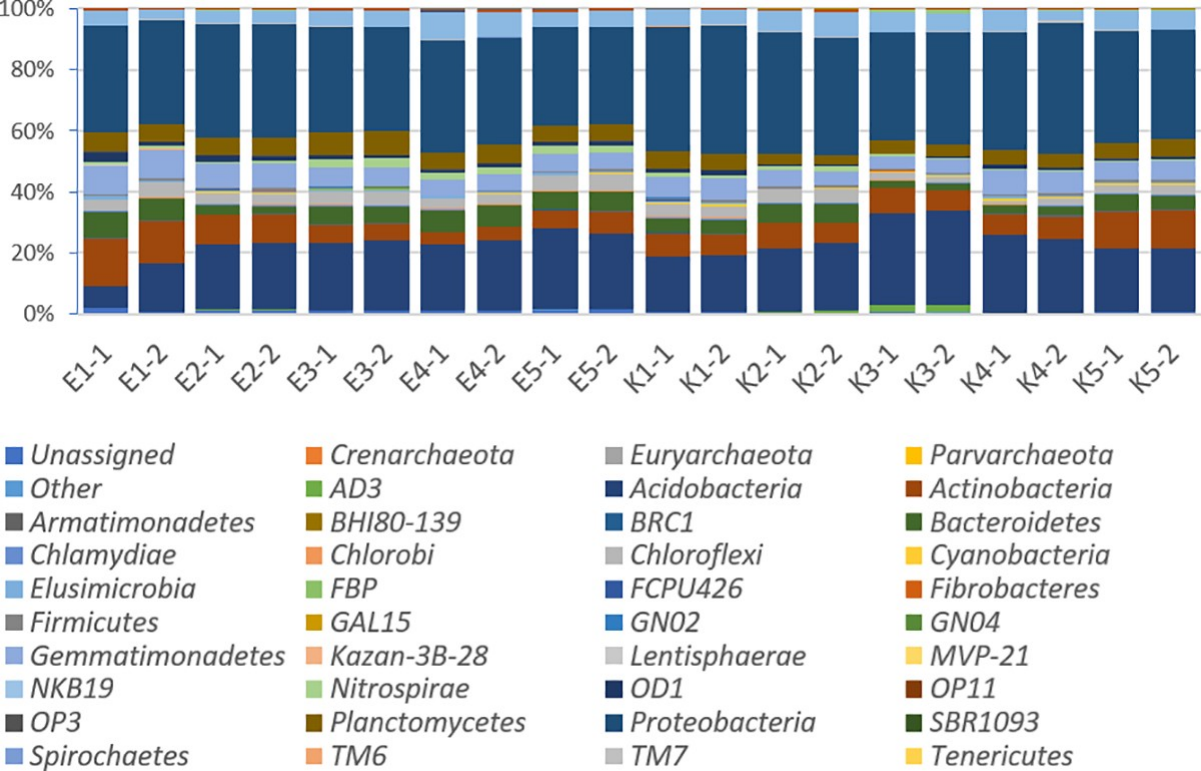
Relative abundance of bacteria and archea at the phylum level in all tested specimens (samples marked with E are from organic farms, while those with K label are from conventional farms).

In soil samples from organic farms on the kingdom level 98.689% reads were classified as bacteria, 0.032% archea, while 1.28% of readings could not be assigned. In the samples from conventional agricultural holdings 99.43% represented bacteria, 0.025% archea and 0.0054% of readings could not be assigned. Although some differences were observed in the composition of the microbial communities present in the samples, the taxonomic groups at the phylum level were generally quite similar in all the soils examined (Fig 2, Table 3).

**Table 3:** The average relative abundance of bacteria at the phylum level in all samples (the table presents only the top 10 microbial phyla in relative abundance, representing more than 2% of the total readings).

Phylum	Average % of total reads	
	Ecological	Convetional
*Proteobacteria*	38.65%	34.93%
*Acidobacteria*	23.09%	21.45%
*Actinobacteria*	8.48%	8.96%
*Gemmatimonadetes*	5.80%	7.22%
*Verrucomicrobia*	5.47%	6.90%
*Planctomycetes*	4.68%	5.18%
*Bacteroidetes*	3.76%	4.44%
*Chloroflexi*	4.78%	3.73%
*AD3*	2.28%	0.00%

We found 45 prokaryotic phyla in twenty tested samples. In all samples dominant phyla were identified as *Proteobacteria* and *Acidobacteria*. The other abundantly present phyla included *Actinobacteria*, *Gemmatimonadetes*, *Planctomycetes*, *Verrucomicrobia*, *Chloroflexi*, *Bacteroidetes* and *AD3* (Fig 2, Table 3). The presence of 1.28% and 0.55% of unclassified phyla for ecological and conventional farms, respectively, implies that tested soils may also harbor many novel bacterial or archaeal organisms. No significant differences were found between conventionally and organically managed soil samples in the abundance of dominant bacterial phyla. The exception occurred in phylum *AD3*, which was detected only in soil from the organic farm.

Differences between samples from organic and conventional farms have also been observed in the taxonomic groups at order level. Quantitative differences were found in the order of bacteria: *Acidimicrobiales*, *Myxococcales*, *iii1-15*, *Pedosphaerales* and *Solibacterales*. Bacteria belonging to the order *Holophagales*, *Ellin6513*, *Chthoniobacterales* and *Saprospirales* appeared only in few soil samples from conventional farms, and their presence was not demonstrated in samples from organic farms. The opposite situation was observed for *Acidimicrobiales*, *Cytophagales*, *MND1* and *Nitrospirales*, which appeared only in single soil samples from organic farms. The exact structure of the microbial community in the individual soil samples was visualised using the Krona Chart (S1 Fig). A more detailed list of bacterial taxa detected in soil samples, summarized in OTU tables for each level, is provided in S1 Table.

### Alpha and beta diversity of bacterial communities

**Fig 3:**
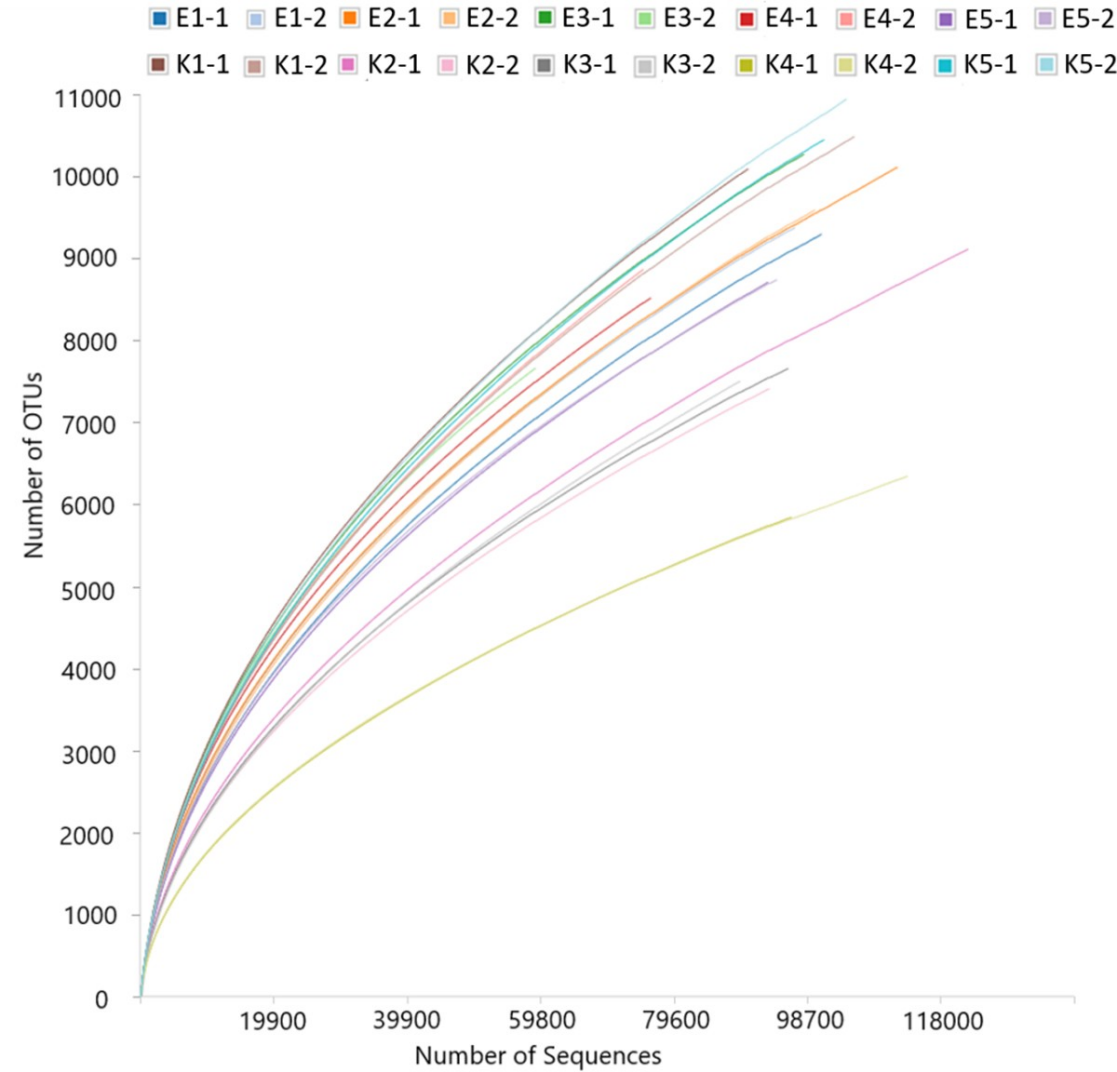
Rarefaction curves of the microbial communities of soil samples derived from conventional (K1-1 – K5-2) and from organic farmsteads (E1-1 – E5-2). Operational taxonomic units (OTU) at a 0.03 distance level.

Rarefaction curves were generated for each sample in order to assess the depth of sampling. Analysis was performed based on OTUs assignment at a 97% similiarity. As shown in Fig 2, the rarefaction curves for majority of samples rise high and display more curvature toward the horizontal. None of these curves reached the plateau level, but some of them nearly arrived to that point. The shape of most rarefaction curves indicate that the sequencing depth reached in this study was not the maximum, but was sufficient to reliably capture the relative complete diversity of these bacterial communities. The number of read sequences (between 54981 for E3.2 and 116237 for K2.1) indicated that the reasonable amount of data was obtained by NGS sequencing (Fig 3). Overall rarefaction analysis revealed that the amount of detected taxa was not dependent on the type of cropping system from which the sample was obtained.

To investigate the α-diversity of microbial communities in tested samples, four metrics were calculated: the Shannon and Simpson indices of diversity, abundance-based coverage estimator (ACE) and Chao1 (a species richness index). The overall microbial richness in the samples derived from ecological and organic farms were not significantly different (Table 4). In contrast, the Shannon and Simpson indices revealed statistically significant (*p*-value <0.05) alterations in the diversity of bacterial communities resulting from the soil management system (Table 4). Indices of diversity was lower in soils obtained from conventional farms.

**Table 4:** Species richness and diversity statistics in soil samples.

	Richness		Diversity	
Sample ID	ACE	Chao1 Index	Simpson index	Shannon index
**E1-1**	26,579	19,492	0.003149	7.155433
**E1-2**	25,117	18,933	0.002052	7.325198
**E2-1**	28,090	21,111	0.002439	7.322669
**E2-2**	27,767	19,984	0.002589	7.258487
**E3-1**	26,646	19,875	0.001983	7.49092
**E3-2**	17,733	13,912	0.002074	7.414196
**E4-1**	21,705	16,572	0.002106	7.373137
**E4-2**	25,227	18,545	0.002062	7.407801
**E5-1**	23,582	18,126	0.003098	7.145678
**E5-2**	22,662	17,591	0.002671	7.221705
**K1-1**	27,358	20,715	0.002099	7.504372
**K1-2**	29,083	21,615	0.002345	7.416237
**K2-1**	30,992	21,276	0.004222	6.875301
**K2-2**	23,276	16,447	0.004487	6.783115
**K3-1**	23,103	16,543	0.004308	6.793589
**K3-2**	24,470	17,062	0.004531	6.729931
**K4-1**	20,380	13,866	0.008714	6.288097
**K4-2**	21,295	14,836	0.008313	6.306989
**K5-1**	28,704	21,288	0.002149	7.437832
**K5-2**	31,857	23,126	0.002095	7.475408

**Fig 4:**
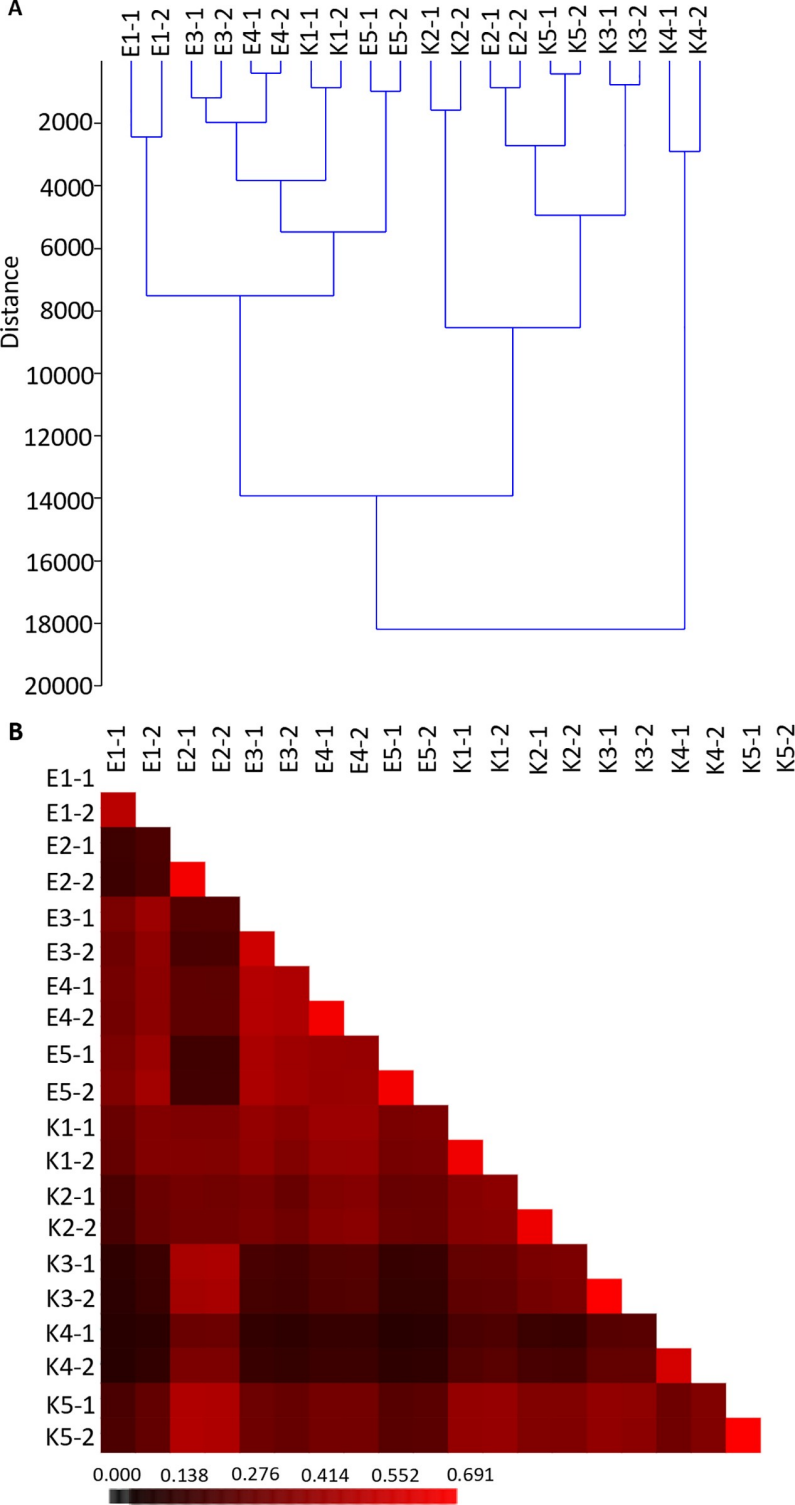
β-diversity between bacterial communities in tested samples. UPGMA dendrogram (A) was constructed in PAST software using Ward’s method and heatmap (B) was built in Mothur based on Bray-Curtis distance. Labels along the left and top sides of heatmap and the upper side of dendrogram represent individual soil samples. Each square within the heatmap represents a single pairwise comparison between the samples. The color code, as indicated by the bar below, represents the similarity of the samples, where darker color represents more dissimilar and brighter indicates more similar communities. Distance trees and heatmaps were constructed at distance of 0.03.

UPGMA clustering method was used to reveal the differences between bacterial communities from conventional and organic farms. Microbial profiles were hierarchically clustered with Ward’s minimum variance method implemented in PAST software. This method allowed to group samples into clusters using analysis of variance. Trees were generated using genus level OTU tables as part of the beta diversity analyses. UPGMA clustering analysis revealed that the shortest relative distance was emerged between the communities in pairs of samples derived from the same soil probes (samples marked with the same letter and first number) (Fig 4A). This high similarity confirms the homogeneity of the samples. Majority of the samples from the same type of agricultural system group together making a cluster. The exceptions are samples E2 and K1, which show more similarity to samples from opposite type of farming system. Samples E1, E3, E4, E5 and K1 were clustered together as one group. Samples K2, K5, K3 and E2 formed another group. K4’s distinctive features put it in a completely separate group from the other samples. Clustering analysis results indicated that majority of samples clustered according to type of soil management system.

Visualization of differences between samples using a heat map based on Bray-Curtis distance confirms that microbial communities in soil samples from organic and conventional farms are largely different (Fig 4B). This analysis also showed greater similarity between samples from farms with the same management regime and confirmed that sample K4 has the least similarity with the other samples.

**Fig 5:**
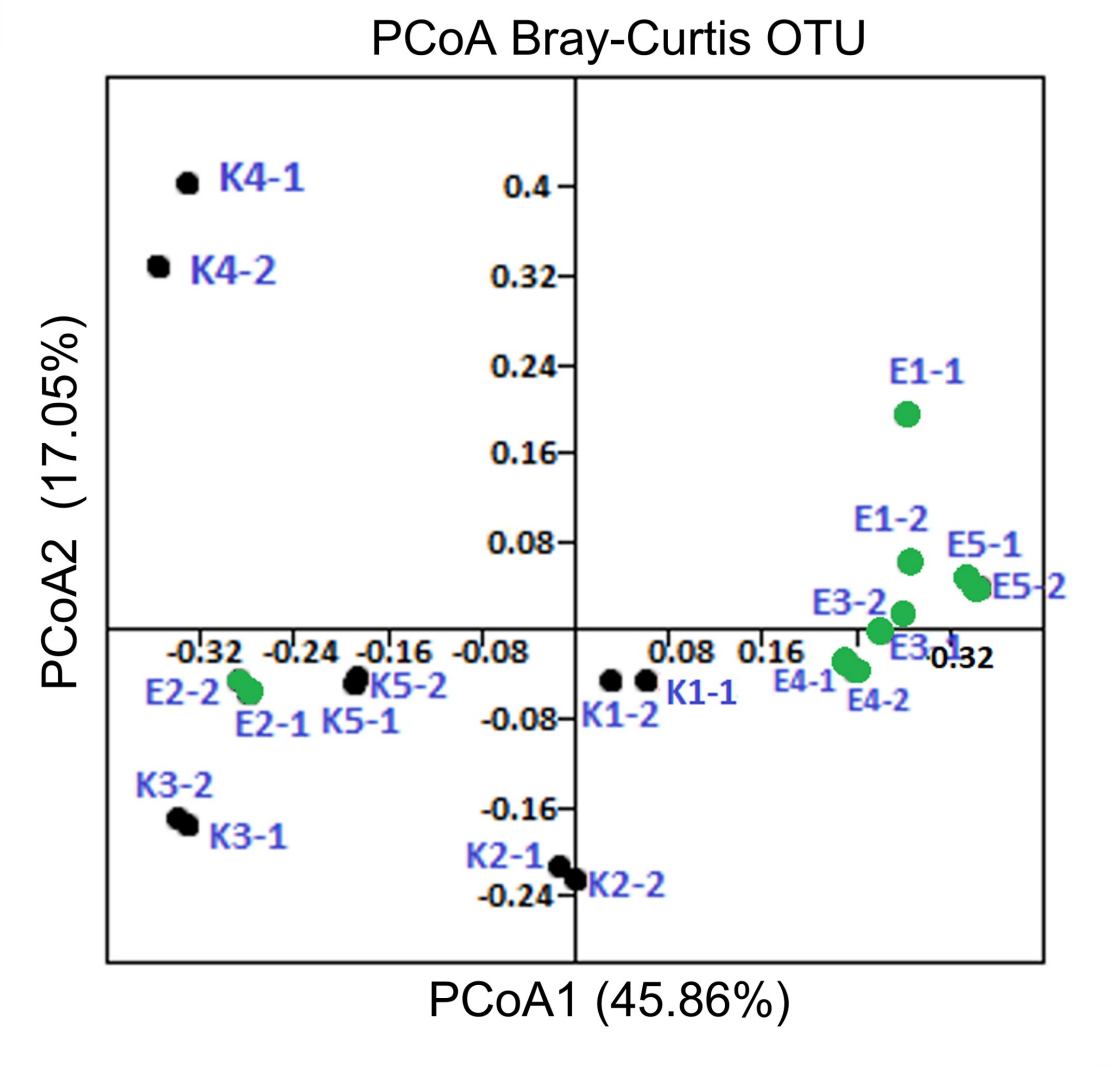
Visualisation of differences between carrot rhizosphere bacterial communities in soils from conventional (K1-1 - K5-2) and organic (E1-1 - E5-2) farms. Clustering based on Bray–Curtis dissimilarity of microbial community profiles in a principal coordinate plot.

PCoA plots were used to assess the variation in the composition of microbial communities between samples and to visualise potential clustering of samples (Fig 5). The plot shows a cluster of eight out of ten samples from organic farms (E1-1, E1-2, E3-1, E3-2, E4-1, E4-2 and E5-1 and E5-2). Most similar to these samples were K1-1 and K1-2 from a convention farm. PCoA analysis also allowed to observe correlation between samples form conventional farm (K3-1, K3-2, K5-1, K5-2) and one soil sample from organic farm (E2-1 and E2-2) and a separate clustering of K4-1 and K4-2. This is consistent with the results from the analyses described above. The PCoA plots also show that all samples collected from organic farms, except E2, cluster relatively close to each other, whereas samples from conventional farms show more diversity among themselves and only two replicates of the same sample show high similarity.

### Enzymes activity

**Fig 6:**
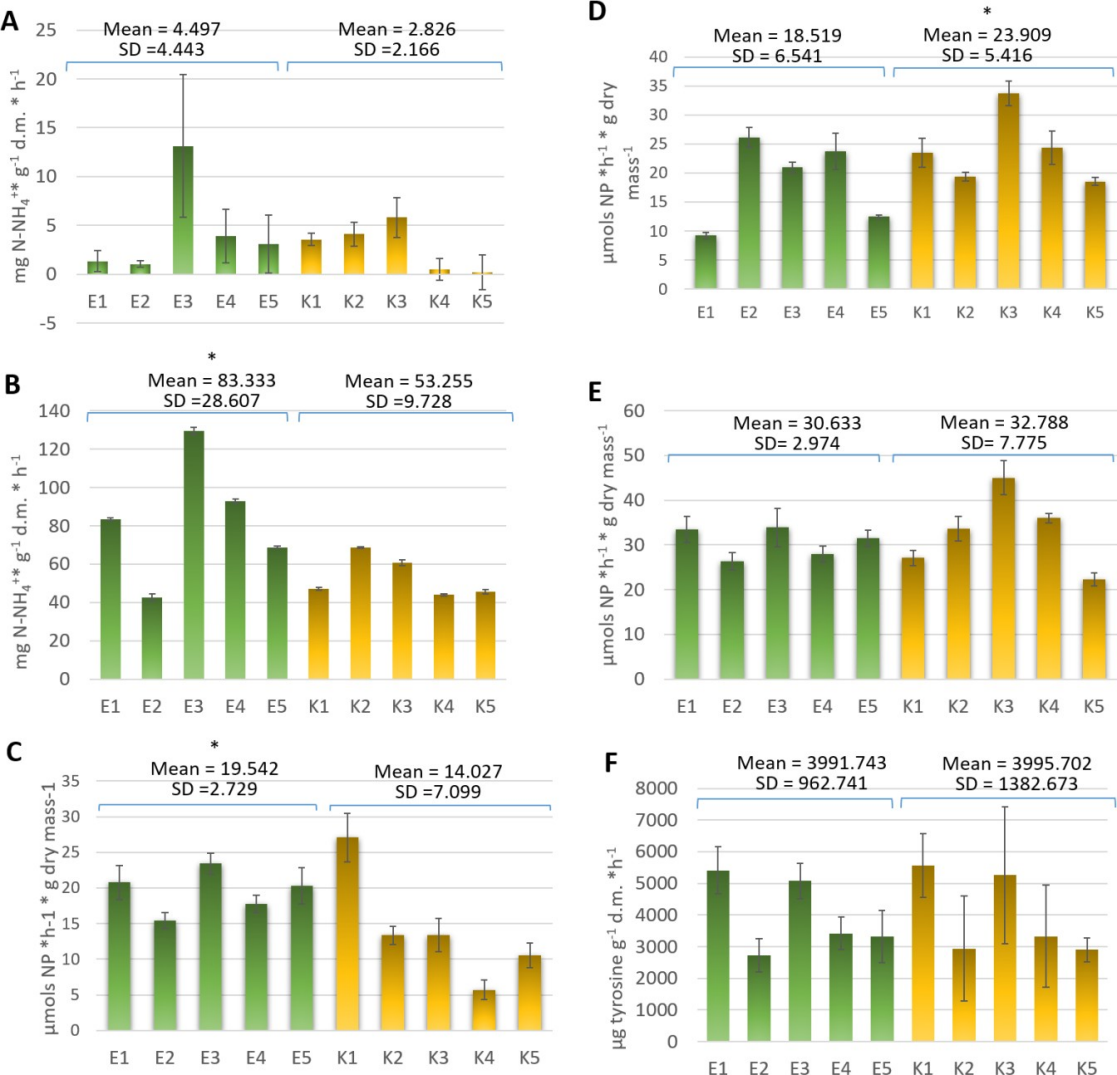
Activity of urease (A), L-glutaminase (B), β-glucosidase (C), acid phosphatase (D), alkaline phosphatase (E), and protease (F) in tested samples derived from organic (green) and conventional (yellow) farming systems.

To determine the effect of soil management system on soil activity, the activities of several soil enzymes were measured, i.e. urease, L-glutaminase, protease, acid phosphatase, alkaline phosphatase and β-glucosidase. The urease assay showed relatively low activity of this enzyme in all samples tested (Fig 6A). No statistically significant differences were observed between samples from organic and conventional crops. The highest activity of this enzyme was reported in sample E3 from organic farm, whereas particularly low activity of this enzyme was observed in samples from farms where large amounts of fertilizers containing a complex of different basic mineral components are applied (samples K4 and K5). The average L-glutaminase activity was shown to be significantly higher (*p* < 0.05) in soil samples from organic farms compared to samples from conventionally managed farms (Fig 6B). Also for this enzyme the lowest activity was observed in samples from soils where mixed mineral fertilizers are used. The same correlations were observed for the beta-glucosidase activity (Fig 6C). As shown in Fig 6D, statistically significant (*p* < 0.05) differences were found between acid phosphatase activity in samples from organic and conventional farms. Higher activity of this enzyme was observed in soil from conventional farming system. On the other hand, there were no significant differences in alkaline phosphatase activity between the samples tested (Fig 6E). The results of the protease assay showed no significant differences (*p* < 0.05) in the mean value of this parameter between soil samples derived from conventional and organic farms (Fig 6F). Overall, these results suggest that organic fertilizers have a more positive effect on enzyme activity than mineral fertilizers.

## Discussion

Organic and conventional farming systems place different emphasis on certain aspects of crop production, environmental protection and the maintenance of biodiversity of soil organisms [39]. Different agricultural practices can cause variation in soil properties, including physico-chemical and biological characteristics. In the present study soil samples obtained from conventional and organic farms from the same region (Lubelskie Voivodeship) were compared. Research of small spatial extent and local scale was undertaken to avoid climatic differences and significant variations in soil type. To date, the majority of studies concerning the microbiome from conventional and organic farms have focused on studies based on controlled field experiments. We aim to connect these investigations with agricultural practice and suggest that the results need to be verified under practical conditions. For this reason it was decided to collect samples from real farms to determine the actual bacterial diversity and soil characteristics under less controlled conditions than in the experimental fields. Furthermore, it was chosen to collect rhizosphere samples from the same plant species (carrot) on all farms to avoid differences in soil microbiome or properties resulting from the presence of dissimilar plants [40].

This study compared the physicochemical properties (pH, soil water content and bulk density) of soils derived from farms employing conventional and organic soil management systems. Bulk densities of the conventionally and organically-farmed soils were not significantly different. The value of this parameter in all samples was relatively low and optimal for plant growth. Soil water content was lower in conventionally cultivated soils, raising the conclusion that increased mineral fertilization reduces soil moisture. These results are in accordance with previous study conducted by Reganold [41] in the Palouse area of Spokane (Washington). The most significant differences between soil samples from conventionally and organically managed farms were observed in the pH value. It was found that the average soil pH was significantly higher in samples from organic farms. Previous studies conducted in New Zealand and Indonesia have also shown that soil pH can be significantly affected by the management regime [42, 43]. Such differences in pH were reflected in variations in bacterial and fungal abundance. The amount of fungi was significantly higher in samples from conventional farms. On the other hand, the organically-farmed soils were slightly more abundant in bacteria. Fungi exceeding bacterial abundance were found in particular in samples K4 and K5 from farms where high amounts of complex mineral fertilizers are applied. Moreover, it was observed that a neutral soil pH favours the growth of bacteria. Fungi predominate in acidic soils (they are most abundant in samples with the lowest pH—i.e. K4 and K5), which is consistent with the fact that fungi prefer a more acidic growth environment than bacteria. The higher acidity of soils on conventional farms is related to the use of mineral fertilizers. Previous research has shown that excessive use of mineral fertilizers, especially those containing ammonium nitrate and superphosphate and also potassium salt, aggravates the loss of organic matter in the soil, reduces soil fertility and accelerates acidification [44]. On the other hand, manure used on organic farms is considered as a beneficial soil conditioner, which favourably changes the pH of the soil towards neutral [45].

Changes in pH also are correlated with differences in the enzymatic activity of soils. It was revealed that the soil management system affected the activity of enzymes such as L-glutaminase, acid phosphatase and beta-glucosidase. In our study, the only enzyme with a higher activity in conventionally-farmed soils was acid phosphatase. This is most probably related to the lower pH of these soil samples, as this enzyme shows higher activity at acidic pH. The organic soil management system exerted a positive effect on the activity of L-glutaminase (involved in nitrogen metabolism) and beta-glucosidase (involved in the carbon cycle). Also in a recent work by Pittarello et al. [46] it was observed that the mean enzyme activity in conventionally cultivated soils were significantly lower than in organically managed soils.

Soil enzymes catalyze and facilitate decomposition and nutrient cycling, and they respond promptly to soil management changes. Their activities may be influenced by various soil parameters, such as pH, moisture, available substrates and soil management system [47]. It was previously shown that the activity of enzymes involved in N, C and P cycling increased when nitrogen was added to the soil, suggesting that soil microorganisms produce more enzymes under conditions of high nitrogen content [48]. However, our study showed that the addition of mineral N-containing fertilizers did not enhance the activity of most of the investigated enzymes. β-glucosidase, involved in the carbon cycle, is linked to the transformation, composition and cycle of soil organic matter. This enzyme is an indicator not only of alterations in soil biological activity, but also of the effect of agrotechnical measures on the soil environment. In our study, we observed a decline in beta-glucosidase activity in soils obtained from conventional farms. This is presumably a consequence of the use of large quantities of mineral fertilisers, which have been shown to significantly reduce the content of this enzyme in the soil. We also observed a reduction in L-glutaminase activity in soils from conventional farms. According to the literature data, there is a correlation between the activity of L-glutaminase and the content of organic carbon in the soil [49]. Thus, the application of organic fertilizers, increasing the organic carbon content, may promote its activity. In contrast, lower L-glutaminase activity can be explained by the agrochemicals used on conventional farms, as it is not significantly correlated with differences in soil pH. In the literature, there are reports showing that a decrease in the activity of this enzyme is correlated with the presence of trace elements (such as Ag, Hg), herbicides, fungicides and insecticides in the soil [50]. Recognising that the extracellular enzymes present in the rhizosphere of plants, involved in the cycling of various elements (carbon, nitrogen, phosphorus), react differently to organic and inorganic fertilization, it is possible to adapt fertilization to support soil activity.

Alterations in enzyme activity in the carrot rhizosphere among the studied samples affect the availability and conversion of nutrients in the soil. This may be caused by the different composition of the microbial community inhabiting these soils, but it may also affect the structure of this community. The diverse composition of bacterial communities in soil is of great significance to soil quality and productivity. In this study, 16S rRNA sequencing was used to comprehensively understand the diversity of microbial communities in soil samples obtained from conventional and organic farms. We presumed that these soils might be colonised by different microbiomes. Phylogenetic studies revealed differences in microbial composition among individual samples at all taxonomic levels, but these results did not allow for the identification of microorganisms specific to a particular cultivation system. The majority of dominant bacterial taxa were similar in both management systems. Relative abundance of bacterial communities was not correlated with soil management. Based on the values of the alpha-diversity indices ACE and Chao1, it was concluded that the type of soil cultivation system does not contribute significantly to changes in soil bacterial richness. However, Shannon and Simpson’s diversity indicators revealed that diversity of the carrot rhizosphere microbiomes was significantly lower in soils obtained from conventional farms than in soil derived from organic farms. This indicates that the use of organic fertilizers has a beneficial effect on bacterial biodiversity in this environment. Studies on maize have also shown that mineral fertilisers and organic manure have a different effect on microbiological parameters. It has been established that long-term manure application leads to changes in the soil microbial community and increases diversity and activity of soil microorganisms [51].

Beta diversity analyses provided a comparison of differences in microbial community structure resulting from soil management practices. The hierarchical clustering showed similarities between samples E1, E3, E4, E5 derived from organic farms and sample K1 from conventional farm. Only the microbiota from a E2 specimens cluster distinctly from the other samples derived from organic farms and is more similar to samples K2, K3, K4 and K5 obtained from conventional farms. In turn, sample K4 was notably distinct from all other specimens. The data obtained indicated that the bacterial community structure in conventionally and organically managed soils differed in most cases. Heatmap based on Bray- Curtis distance have confirmed these correlations between the samples. Similarly, PCoA showed that bacterial OTUs from soils cultivated under different management practices for the majority of samples clearly clustered into different groups. This leads us to believe that soil managment practices affect the composition of soil-dwelling microbial communities. The type of fertilizer being used is particularly important. Fertilization influences the diversity of soil microorganisms by directly affecting the nutrient content of the soil. Reoccurring extensive use of mineral fertilizers can adversely affect soil quality [21]. Long-term use of nitrogen fertilizers, or mineral multicomponent fertilizers, affects the nitrogen cycle and related bacteria [20]. It has been observed that the response of microbial communities to soil management is very complex and consists of many factors. Although studies confirm the positive impact of organic farming on biodiversity, it is also noted that properly managed conventional farms may not necessarily have a negative impact on soil microorganisms and their diversity, especially if organic fertilizers are introduced to the soil in addition to mineral fertilizers.

The Pearson Correlation coefficient was used to determine if there was a correlation between the abundance of each bacterial phylum and the activity of the enzymes tested (data not shown). The analysis revealed that there are some relationships between certain groups of bacteria and the activity of selected enzymes. Among the enzymes involved in the nitrogen cycle, urease activity correlated with the presence of *AD3* (also known as *Dormibacteraeota*) and protease activity was connected with *AD3*, *Bacteroidetes* and *Gammatimonadetes* phyla, while glutaminase activity was associated with the occurrence of *Bacteroidetes*. In contrast, beta-galactosidase activity involved in the carbon cycle was most strongly correlated with the presence of *Planctomycetes* and *Bacteroidetes*. Acid phosphatase activity was highest in samples where bacterial phyla such as *Acidobacteria*, *Verrucomicrobia*, *AD3* and *Proteobacteria* were most abundantly represented. In turn, alkaline phosphatase was associated with the presence of *AD3* and *Acidobacteria*. This is related to the functions performed in the soil by each group of bacteria. *AD3* phylum members, which in this study were found only in one sample derived from conventional farm, has previously been found to be able to survive under low resource conditions and is probably able to use trace amounts of CO as an additional energy source [52]. Bacteria belonging to *Bacteroidetes* was reported to affect the dissolution of organic nitrogen in soil and have proteolytic properties. *Bacteroidetes* together with *Proteobacteria* promote mineralization of organic matter and increase soluble organic nitrogen. Furthermore, these phylla are correlated with the availability of organic carbon and the mineralization rate of this element [53]. *Planctomycetes* was found to participate in the global carbon and nitrogen cycles [54]. Acidobacteria, on the other hand, are involved in the phosphorus cycle in nature, and literature data confirm that genes encoding alkaline phosphatase are present in this group of bacteria [55].

## Conclusions

Metagenomic analyses identified 16S rRNA profiles of microorganisms colonizing organic and conventional agricultural soils (carrot rhizosphere). Some similarities and differences in biodiversity and species distributions in both soil management systems were detected, which might be associated with the different fertilization strategies. Soil pH may also be a factor that directly contributes to the relationships demonstrated. Variations in the activity of certain soil enzymes were also a variable associated with the composition of these communities. The metagenomic profiles obtained contribute to a better understanding of soil microbial communities under different soil management regimes. However, the response of bacterial communities to soil management is very complex. To understand the exact relationships that occur there, further research involving the multitude of factors that interact in this environment is required.

## Supporting information

S1 Map Location of soil sampling sites from conventional (K1—K5) and organic (E1-E5) farms. (MHTML)

S1 Fig Structure of the microbial community in the individual soil samples visualised using the Krona Chart. (HTML)

S1 Table List of bacterial taxa detected in all soil samples, summarized in OUT tables (a table for each taxonomic level is provided in a separate worksheet). (XLS)
