# Supplementary material for: Is soil management system really important? comparison of microbial community diversity and structure in soils managed under organic and conventional regimes with some view on soil properties
Source: PLoS One. 2021 Sep 9;16(9):e0256969. doi: 10.1371/journal.pone.0256969 (PMC8428661; doi:10.1371/journal.pone.0256969)
Supplement: S1 Fig — (HTML) [file pone.0256969.s002.html]

Javascript must be enabled to view this page.

magnitude
score


E1.1
E1.2
E2.1
E2.2
E3.1
E3.2
E4.1
E4.2
E5.1
E5.2
K1.1
K1.2
K2.1
K2.2
K3.1
K3.2
K4.1
K4.2
K5.1
K5.2

 .99999999999993
 1.00000000000035
 1.00000000000026
 1.00000000000013
 1.00000000000026
 .99999999999998
 1.00000000000001
 .999999999999983
 1.0000000000004
 1.00000000000008
 1.00000000000012
 .999980063000844
 1.00000000000011
 1.00000000000018
 .999977636890179
 .999999999999901
 1.0000000000002
 1.00000000000002
 .999999999999968
 1.00000000000004

 1671.67593160896
 1364.24596038372
 1579.61453091734
 1432.98332849781
 1577.55381558708
 967.083592513707
 1395.3941007676
 1393.99929722113
 1867.6829870128
 1734.1100413541
 1287.94253919387
 1509.3203939627
 2069.15778108579
 1757.12667257398
 2035.62036653982
 2027.82291235668
 2241.66948753037
 2452.03129719088
 1251.20613470602
 1239.85334945185

 .0190157825515
 .00937876726722
 .0129974740946
 .0128886111545
 .0117928559221
 .0119132063804
 .0102769436103
 .0100686560709
 .0138528138528
 .015680192987
 .0076287793953
 .00762590214921
 .00277880537178
 .00286005899573
 .00609394742433
 .00494073551245
 .00410783631507
 .00408675626699
 .00747533247533
 .00693025970236

 1735
 848
 1307
 1155
 1104
 655
 770
 745
 1280
 1456
 654
 765
 323
 255
 545
 406
 366
 433
 697
 665

 .0190157825515
 .00937876726722
 .0129974740946
 .0128886111545
 .0117928559221
 .0119132063804
 .0102769436103
 .0100686560709
 .0138528138528
 .015680192987
 .0076287793953
 .00762590214921
 .00277880537178
 .00286005899573
 .00609394742433
 .00494073551245
 .00410783631507
 .00408675626699
 .00747533247533
 .00693025970236

 1735
 848
 1307
 1155
 1104
 655
 770
 745
 1280
 1456
 654
 765
 323
 255
 545
 406
 366
 433
 697
 665

 .0190157825515
 .00937876726722
 .0129974740946
 .0128886111545
 .0117928559221
 .0119132063804
 .0102769436103
 .0100686560709
 .0138528138528
 .015680192987
 .0076287793953
 .00762590214921
 .00277880537178
 .00286005899573
 .00609394742433
 .00494073551245
 .00410783631507
 .00408675626699
 .00747533247533
 .00693025970236

 1735
 848
 1307
 1155
 1104
 655
 770
 745
 1280
 1456
 654
 765
 323
 255
 545
 406
 366
 433
 697
 665

 .0190157825515
 .00937876726722
 .0129974740946
 .0128886111545
 .0117928559221
 .0119132063804
 .0102769436103
 .0100686560709
 .0138528138528
 .015680192987
 .0076287793953
 .00762590214921
 .00277880537178
 .00286005899573
 .00609394742433
 .00494073551245
 .00410783631507
 .00408675626699
 .00747533247533
 .00693025970236

 1735
 848
 1307
 1155
 1104
 655
 770
 745
 1280
 1456
 654
 765
 323
 255
 545
 406
 366
 433
 697
 665

 .0190157825515
 .00937876726722
 .0129974740946
 .0128886111545
 .0117928559221
 .0119132063804
 .0102769436103
 .0100686560709
 .0138528138528
 .015680192987
 .0076287793953
 .00762590214921
 .00277880537178
 .00286005899573
 .00609394742433
 .00494073551245
 .00410783631507
 .00408675626699
 .00747533247533
 .00693025970236

 1735
 848
 1307
 1155
 1104
 655
 770
 745
 1280
 1456
 654
 765
 323
 255
 545
 406
 366
 433
 697
 665

 .0190157825515
 .00937876726722
 .0129974740946
 .0128886111545
 .0117928559221
 .0119132063804
 .0102769436103
 .0100686560709
 .0138528138528
 .015680192987
 .0076287793953
 .00762590214921
 .00277880537178
 .00286005899573
 .00609394742433
 .00494073551245
 .00410783631507
 .00408675626699
 .00747533247533
 .00693025970236

 1735
 848
 1307
 1155
 1104
 655
 770
 745
 1280
 1456
 654
 765
 323
 255
 545
 406
 366
 433
 697
 665

 .0190157825515
 .00937876726722
 .0129974740946
 .0128886111545
 .0117928559221
 .0119132063804
 .0102769436103
 .0100686560709
 .0138528138528
 .015680192987
 .0076287793953
 .00762590214921
 .00277880537178
 .00286005899573
 .00609394742433
 .00494073551245
 .00410783631507
 .00408675626699
 .00747533247533
 .00693025970236

 1735
 848
 1307
 1155
 1104
 655
 770
 745
 1280
 1456
 654
 765
 323
 255
 545
 406
 366
 433
 697
 665

 .000416483998246
 2.322572082686E-04
 3.3811332763193E-04
 .000278974267414
 .000416595453768
 2.000691147851E-04
 .0004004004004
 2.432695426529E-04
 4.004329004331E-04
 3.553889894036E-04
 1.049832026876E-04
 2.392439889951E-04
 7.742801345524E-05
 7.85114234121E-05
 6.261670747931E-04
 .000547618468128
 3.816022806345E-04
 1.6045001510084E-04
 1.394251394251E-04
 1.980074200677E-04

 19.2105263157895
 8.23809523809848
 12.3529411764717
 10.4400000000044
 17.5641025641026
 9.18181818181818
 12.6
 8.33333333333018
 18.6756756756807
 11.6060606060656
 3.22222222222275
 13.2500000000072
 2.55555555555518
 2.14285714285714
 19.3571428571524
 16.3777777777879
 30.2352941176471
 13.3529411764675
 4.5384615384633
 12.3684210526401

 .000032880315651
 6.63592023624E-05
 2.386682312696E-04
 2.008614725382E-04
 .000245684498376
 .000181881013441
 .00024024024024
 1.756946696937E-04
 2.813852813855E-04
 .000161540449729
 8.16536020904E-05
 1.894014912879E-04
 5.161867563684E-05
 5.60795881515E-05
 .000469625306095
 .000377248278044
 3.816022806345E-04
 1.6045001510084E-04
 .00006435006435
 1.04214431614E-05

 1.66666666666667
 3.33333333333333
 14.0833333333347
 11.7777777777811
 21.0869565217391
 10
 13
 9.61538461538186
 24.0769230769247
 15
 3.5714285714284
 15.4210526315819
 2.33333333333295
 2.2
 22.7142857142948
 17.451612903238
 30.2352941176471
 13.3529411764675
 1.66666666666667
 1

 .000032880315651
 6.63592023624E-05
 .000169056663816
 .000156225589752
 .000245684498376
 .000181881013441
 .00024024024024
 1.756946696937E-04
 2.813852813855E-04
 .000161540449729
 8.16536020904E-05
 1.894014912879E-04
 4.30155630307E-05
 2.24318352606E-05
 .00030190198249
 .000255555285127
 3.816022806345E-04
 1.6045001510084E-04
 .0000429000429
 1.04214431614E-05

 1.66666666666667
 3.33333333333333
 17
 14
 21.0869565217391
 10
 13
 9.61538461538186
 24.0769230769247
 15
 3.5714285714284
 15.4210526315819
 2.59999999999954
 1
 27
 21
 30.2352941176471
 13.3529411764675
 1.5
 1

 .000032880315651
 6.63592023624E-05
 0
 0
 .000245684498376
 .000181881013441
 .00024024024024
 1.756946696937E-04
 2.813852813855E-04
 .000161540449729
 8.16536020904E-05
 1.894014912879E-04
 4.30155630307E-05
 2.24318352606E-05
 0
 0
 2.24471929785E-05
 1.887647236484E-05
 .000032175032175
 1.04214431614E-05

 1.66666666666667
 3.33333333333333
 3.33333333333333
 3.33333333333333
 21.0869565217391
 10
 13
 9.61538461538186
 24.0769230769247
 15
 3.5714285714284
 15.4210526315819
 2.59999999999954
 1
 1
 1
 2
 1
 1.66666666666667
 1

 .000032880315651
 6.63592023624E-05
 0
 0
 .000245684498376
 .000181881013441
 .00024024024024
 1.756946696937E-04
 2.813852813855E-04
 .000161540449729
 8.16536020904E-05
 1.894014912879E-04
 4.30155630307E-05
 2.24318352606E-05
 0
 0
 2.24471929785E-05
 1.887647236484E-05
 .000032175032175
 1.04214431614E-05

 1.66666666666667
 3.33333333333333
 3.33333333333333
 3.33333333333333
 21.0869565217391
 10
 13
 9.61538461538186
 24.0769230769247
 15
 3.5714285714284
 15.4210526315819
 2.59999999999954
 1
 1
 1
 2
 1
 1.66666666666667
 1

 .000032880315651
 6.63592023624E-05
 0
 0
 .000245684498376
 .000181881013441
 .00024024024024
 1.756946696937E-04
 2.813852813855E-04
 .000161540449729
 8.16536020904E-05
 1.894014912879E-04
 4.30155630307E-05
 2.24318352606E-05
 0
 0
 2.24471929785E-05
 1.887647236484E-05
 .000032175032175
 1.04214431614E-05

 1.66666666666667
 3.33333333333333
 3.33333333333333
 3.33333333333333
 21.0869565217391
 10
 13
 9.61538461538186
 24.0769230769247
 15
 3.5714285714284
 15.4210526315819
 2.59999999999954
 1
 1
 1
 2
 1
 1.66666666666667
 1

 .000010960105217
 2.21197341208E-05
 0
 0
 .000010681934712
 0
 .00004004004004
 2.70299491837E-05
 1.08225108225E-05
 0
 4.66592011945E-05
 1.99369990829E-05
 2.58093378184E-05
 1.12159176303E-05
 0
 0
 2.24471929785E-05
 9.43823618242E-06
 .000010725010725

 1
 2
 2
 2
 1
 1
 3
 2
 1
 1
 4
 2
 3
 1
 1
 1
 2
 1
 1

 .000021920210434
 4.42394682416E-05
 0
 0
 .000235002563664
 .000181881013441
 .0002002002002
 .00014866472051
 .000270562770563
 .000161540449729
 3.49944008959E-05
 .000169464492205
 1.72062252123E-05
 1.12159176303E-05
 0
 0
 0
 9.43823618242E-06
 .00002145002145
 1.04214431614E-05

 2
 4
 4
 4
 22
 10
 15
 11
 25
 15
 3
 17
 2
 1
 1
 1
 1
 1
 2
 1

 0
 0
 .000169056663816
 .000156225589752
 0
 0
 0
 0
 0
 0
 0
 0
 0
 0
 .00030190198249
 .000255555285127
 .000359155087656
 .000141573542736
 .000010725010725

 0
 0
 17
 14
 14
 14
 14
 14
 14
 14
 14
 14
 14
 14
 27
 21
 32
 15
 1

 0
 0
 .000169056663816
 .000156225589752
 0
 0
 0
 0
 0
 0
 0
 0
 0
 0
 .00030190198249
 .000255555285127
 .000359155087656
 .000141573542736
 .000010725010725

 0
 0
 17
 14
 14
 14
 14
 14
 14
 14
 14
 14
 14
 14
 27
 21
 32
 15
 1

 0
 0
 .000169056663816
 .000156225589752
 0
 0
 0
 0
 0
 0
 0
 0
 0
 0
 .00030190198249
 .000255555285127
 .000359155087656
 .000141573542736
 .000010725010725

 0
 0
 17
 14
 14
 14
 14
 14
 14
 14
 14
 14
 14
 14
 27
 21
 32
 15
 1

 0
 0
 .000169056663816
 .000156225589752
 0
 0
 0
 0
 0
 0
 0
 0
 0
 0
 .00030190198249
 .000255555285127
 .000359155087656
 .000141573542736
 .000010725010725

 0
 0
 17
 14
 14
 14
 14
 14
 14
 14
 14
 14
 14
 14
 27
 21
 32
 15
 1

 0
 0
 6.96115674536E-05
 4.46358827862E-05
 0
 0
 0
 0
 0
 0
 0
 0
 8.60311260614E-06
 3.36477528909E-05
 .000167723323605
 .000121692992917
 0
 0
 .00002145002145

 0
 0
 7
 4
 4
 4
 4
 4
 4
 4
 4
 4
 1
 3
 15
 10
 10
 10
 2

 0
 0
 6.96115674536E-05
 4.46358827862E-05
 0
 0
 0
 0
 0
 0
 0
 0
 8.60311260614E-06
 3.36477528909E-05
 .000167723323605
 .000121692992917
 0
 0
 .00002145002145

 0
 0
 7
 4
 4
 4
 4
 4
 4
 4
 4
 4
 1
 3
 15
 10
 10
 10
 2

 0
 0
 6.96115674536E-05
 4.46358827862E-05
 0
 0
 0
 0
 0
 0
 0
 0
 8.60311260614E-06
 3.36477528909E-05
 .000167723323605
 .000121692992917
 0
 0
 .00002145002145

 0
 0
 7
 4
 4
 4
 4
 4
 4
 4
 4
 4
 1
 3
 15
 10
 10
 10
 2

 0
 0
 6.96115674536E-05
 4.46358827862E-05
 0
 0
 0
 0
 0
 0
 0
 0
 8.60311260614E-06
 3.36477528909E-05
 .000167723323605
 .000121692992917
 0
 0
 .00002145002145

 0
 0
 7
 4
 4
 4
 4
 4
 4
 4
 4
 4
 1
 3
 15
 10
 10
 10
 2

 0
 0
 6.96115674536E-05
 4.46358827862E-05
 0
 0
 0
 0
 0
 0
 0
 0
 8.60311260614E-06
 3.36477528909E-05
 .000167723323605
 .000121692992917
 0
 0
 .00002145002145

 0
 0
 7
 4
 4
 4
 4
 4
 4
 4
 4
 4
 1
 3
 15
 10
 10
 10
 2

 .000274002630425
 .000132718404725
 9.94450963623E-06
 0
 .000021363869424
 0
 0
 0
 7.57575757576E-05
 .000118462996468
 0
 0
 2.58093378184E-05
 2.24318352606E-05
 .000122997103977
 .000170370190084
 0
 0
 7.50750750751E-05
 .000156321647422

 25
 12
 1
 1
 2
 2
 2
 2
 7
 11
 11
 11
 3
 2
 11
 14
 14
 14
 7
 15

 .000274002630425
 .000132718404725
 9.94450963623E-06
 0
 .000021363869424
 0
 0
 0
 7.57575757576E-05
 .000118462996468
 0
 0
 2.58093378184E-05
 2.24318352606E-05
 .000122997103977
 .000170370190084
 0
 0
 7.50750750751E-05
 .000156321647422

 25
 12
 1
 1
 2
 2
 2
 2
 7
 11
 11
 11
 3
 2
 11
 14
 14
 14
 7
 15

 .000274002630425
 .000132718404725
 9.94450963623E-06
 0
 .000021363869424
 0
 0
 0
 7.57575757576E-05
 .000118462996468
 0
 0
 2.58093378184E-05
 2.24318352606E-05
 .000122997103977
 .000170370190084
 0
 0
 7.50750750751E-05
 .000156321647422

 25
 12
 1
 1
 2
 2
 2
 2
 7
 11
 11
 11
 3
 2
 11
 14
 14
 14
 7
 15

 .000274002630425
 .000132718404725
 9.94450963623E-06
 0
 .000021363869424
 0
 0
 0
 7.57575757576E-05
 .000118462996468
 0
 0
 2.58093378184E-05
 2.24318352606E-05
 .000122997103977
 .000170370190084
 0
 0
 7.50750750751E-05
 .000156321647422

 25
 12
 1
 1
 2
 2
 2
 2
 7
 11
 11
 11
 3
 2
 11
 14
 14
 14
 7
 15

 .000274002630425
 .000132718404725
 9.94450963623E-06
 0
 .000021363869424
 0
 0
 0
 7.57575757576E-05
 .000118462996468
 0
 0
 2.58093378184E-05
 2.24318352606E-05
 .000122997103977
 .000170370190084
 0
 0
 7.50750750751E-05
 .000156321647422

 25
 12
 1
 1
 2
 2
 2
 2
 7
 11
 11
 11
 3
 2
 11
 14
 14
 14
 7
 15

 .000274002630425
 .000132718404725
 9.94450963623E-06
 0
 .000021363869424
 0
 0
 0
 7.57575757576E-05
 .000118462996468
 0
 0
 2.58093378184E-05
 2.24318352606E-05
 .000122997103977
 .000170370190084
 0
 0
 7.50750750751E-05
 .000156321647422

 25
 12
 1
 1
 2
 2
 2
 2
 7
 11
 11
 11
 3
 2
 11
 14
 14
 14
 7
 15

 .00010960105217
 3.31796011812E-05
 8.95005867261E-05
 7.81127948758E-05
 .000149547085968
 1.81881013441E-05
 .00016016016016
 6.75748729592E-05
 .00004329004329
 7.53855432066E-05
 2.33296005972E-05
 4.98424977072E-05
 0
 0
 3.35446647211E-05
 0
 0
 0
 0
 3.12643294843E-05

 10
 3
 9
 7
 14
 1
 12
 5
 4
 5.28571428571618
 2
 5
 5
 5
 3
 3
 3
 3
 3
 3

 .00010960105217
 3.31796011812E-05
 8.95005867261E-05
 7.81127948758E-05
 .000149547085968
 1.81881013441E-05
 .00016016016016
 6.75748729592E-05
 .00004329004329
 7.53855432066E-05
 2.33296005972E-05
 4.98424977072E-05
 0
 0
 3.35446647211E-05
 0
 0
 0
 0
 3.12643294843E-05

 10
 3
 9
 7
 14
 1
 12
 5
 4
 5.28571428571618
 2
 5
 5
 5
 3
 3
 3
 3
 3
 3

 .00010960105217
 3.31796011812E-05
 8.95005867261E-05
 7.81127948758E-05
 .000149547085968
 1.81881013441E-05
 .00016016016016
 6.75748729592E-05
 0
 1.07693633152E-05
 2.33296005972E-05
 4.98424977072E-05
 0
 0
 3.35446647211E-05
 0
 0
 0
 0
 3.12643294843E-05

 10
 3
 9
 7
 14
 1
 12
 5
 5
 1
 2
 5
 5
 5
 3
 3
 3
 3
 3
 3

 .00010960105217
 3.31796011812E-05
 8.95005867261E-05
 7.81127948758E-05
 .000149547085968
 1.81881013441E-05
 .00016016016016
 6.75748729592E-05
 0
 1.07693633152E-05
 2.33296005972E-05
 4.98424977072E-05
 0
 0
 3.35446647211E-05
 0
 0
 0
 0
 3.12643294843E-05

 10
 3
 9
 7
 14
 1
 12
 5
 5
 1
 2
 5
 5
 5
 3
 3
 3
 3
 3
 3

 .00010960105217
 3.31796011812E-05
 8.95005867261E-05
 7.81127948758E-05
 .000149547085968
 1.81881013441E-05
 .00016016016016
 6.75748729592E-05
 0
 1.07693633152E-05
 2.33296005972E-05
 4.98424977072E-05
 0
 0
 3.35446647211E-05
 0
 0
 0
 0
 3.12643294843E-05

 10
 3
 9
 7
 14
 1
 12
 5
 5
 1
 2
 5
 5
 5
 3
 3
 3
 3
 3
 3

 .00010960105217
 3.31796011812E-05
 8.95005867261E-05
 7.81127948758E-05
 .000149547085968
 1.81881013441E-05
 .00016016016016
 6.75748729592E-05
 0
 1.07693633152E-05
 2.33296005972E-05
 4.98424977072E-05
 0
 0
 3.35446647211E-05
 0
 0
 0
 0
 3.12643294843E-05

 10
 3
 9
 7
 14
 1
 12
 5
 5
 1
 2
 5
 5
 5
 3
 3
 3
 3
 3
 3

 0
 0
 0
 0
 0
 0
 0
 0
 .00004329004329
 6.46161798914E-05

 0
 0
 0
 0
 0
 0
 0
 0
 4
 6

 0
 0
 0
 0
 0
 0
 0
 0
 .00004329004329
 6.46161798914E-05

 0
 0
 0
 0
 0
 0
 0
 0
 4
 6

 0
 0
 0
 0
 0
 0
 0
 0
 .00004329004329
 6.46161798914E-05

 0
 0
 0
 0
 0
 0
 0
 0
 4
 6

 0
 0
 0
 0
 0
 0
 0
 0
 .00004329004329
 6.46161798914E-05

 0
 0
 0
 0
 0
 0
 0
 0
 4
 6

 .980567733450184
 .990388975524861
 .986664412578029
 .986832414578216
 .987790548624388
 .987886724504795
 .989322655989312
 .98968807438643
 .985746753247166
 .983964418023676
 .992266237402135
 .992114916862639
 .997143766614876
 .997061429581043
 .993257522391056
 .994511646019323
 .995510561404497
 .995752793717927
 .992385242385213
 .992871732877614

 1671.14977589504
 1369.45269576109
 1583.74279609328
 1437.01610240408
 1583.86531203704
 971.04109362048
 1402.45025295127
 1400.94253642662
 1876.69287353273
 1739.16406361133
 1292.95236583803
 1515.4023913587
 2074.18438376315
 1761.57369765036
 2046.03687943288
 2036.98771459684
 2250.25687163161
 2460.71073534121
 1255.55594942169
 1244.11063061551

 .000142481367821
 7.74190694228E-05
 5.96670578174E-05
 6.69538241793E-05
 .000149547085968
 .000218257216129
 2.66933600267E-05
 6.75748729592E-05
 .00021645021645
 .000118462996468
 0
 9.96849954145E-06
 6.88249008491E-05
 0
 6.70893294422E-05
 .000097354394334
 2.24471929785E-05
 .000556855934763
 5.36250536251E-05
 6.25286589687E-05

 13
 7
 6
 6
 14
 12
 2
 5
 20
 11
 11
 1
 8
 8
 6
 8
 2
 59
 5
 6

 .000142481367821
 7.74190694228E-05
 5.96670578174E-05
 6.69538241793E-05
 .000149547085968
 .000218257216129
 2.66933600267E-05
 6.75748729592E-05
 .00021645021645
 .000118462996468
 0
 9.96849954145E-06
 6.88249008491E-05
 0
 6.70893294422E-05
 .000097354394334
 2.24471929785E-05
 .000556855934763
 5.36250536251E-05
 6.25286589687E-05

 13
 7
 6
 6
 14
 12
 2
 5
 20
 11
 11
 1
 8
 8
 6
 8
 2
 59
 5
 6

 .000142481367821
 7.74190694228E-05
 5.96670578174E-05
 6.69538241793E-05
 .000149547085968
 .000218257216129
 2.66933600267E-05
 6.75748729592E-05
 .00021645021645
 .000118462996468
 0
 9.96849954145E-06
 6.88249008491E-05
 0
 6.70893294422E-05
 .000097354394334
 2.24471929785E-05
 .000556855934763
 5.36250536251E-05
 6.25286589687E-05

 13
 7
 6
 6
 14
 12
 2
 5
 20
 11
 11
 1
 8
 8
 6
 8
 2
 59
 5
 6

 .000142481367821
 7.74190694228E-05
 5.96670578174E-05
 6.69538241793E-05
 .000149547085968
 .000218257216129
 2.66933600267E-05
 6.75748729592E-05
 .00021645021645
 .000118462996468
 0
 9.96849954145E-06
 6.88249008491E-05
 0
 6.70893294422E-05
 .000097354394334
 2.24471929785E-05
 .000556855934763
 5.36250536251E-05
 6.25286589687E-05

 13
 7
 6
 6
 14
 12
 2
 5
 20
 11
 11
 1
 8
 8
 6
 8
 2
 59
 5
 6

 .000142481367821
 7.74190694228E-05
 5.96670578174E-05
 6.69538241793E-05
 .000149547085968
 .000218257216129
 2.66933600267E-05
 6.75748729592E-05
 .00021645021645
 .000118462996468
 0
 9.96849954145E-06
 6.88249008491E-05
 0
 6.70893294422E-05
 .000097354394334
 2.24471929785E-05
 .000556855934763
 5.36250536251E-05
 6.25286589687E-05

 13
 7
 6
 6
 14
 12
 2
 5
 20
 11
 11
 1
 8
 8
 6
 8
 2
 59
 5
 6

 .000142481367821
 7.74190694228E-05
 5.96670578174E-05
 6.69538241793E-05
 .000149547085968
 .000218257216129
 2.66933600267E-05
 6.75748729592E-05
 .00021645021645
 .000118462996468
 0
 9.96849954145E-06
 6.88249008491E-05
 0
 6.70893294422E-05
 .000097354394334
 2.24471929785E-05
 .000556855934763
 5.36250536251E-05
 6.25286589687E-05

 13
 7
 6
 6
 14
 12
 2
 5
 20
 11
 11
 1
 8
 8
 6
 8
 2
 59
 5
 6

 7.15037264357561E-02
 .156154263025782
 .212384892300958
 .215948400919415
 .222397880704134
 .230570560739055
 .219686353019789
 .233376581252045
 .269123376623392
 .246349185836037
 .180162840612246
 .183011683081477
 .204521795985825
 .220415213270812
 .300068207484952
 .312130357533988
 .255729646007831
 .240165357897879
 .207979407979429
 .204562507816166

 1308.53218884069
 2956.2560379632
 2521.93861497349
 2282.23222405928
 3628.98001921234
 2278.28705529717
 3024.17326852986
 3070.66967801632
 4070.99227892345
 3865.45088524593
 2267.93376497239
 2407.57780924788
 2615.80646111125
 2078.51842051748
 3761.07542107668
 3715.34465281324
 4630.26688610813
 3892.47771751919
 1795.90594059397
 1872.75373172377

 .000635686102587
 .000486634150658
 .000208834702361
 .00025665632602
 .00100410186293
 .00105490987796
 .00146813480147
 .00122986268786
 .00141774891775
 .000893857155165
 .000513251213139
 .000777542964233
 .00179805053468
 .00200764925582
 .000167723323605
 .00020687808796
 0
 0
 .00040755040755
 .000354329067489

 58
 44
 21
 23
 94
 58
 110
 91
 131
 83
 44
 78
 209
 179
 15
 17
 17
 17
 38
 34

 .000635686102587
 .000486634150658
 .000208834702361
 .00025665632602
 .00100410186293
 .00105490987796
 .00146813480147
 .00122986268786
 .00141774891775
 .000893857155165
 .000513251213139
 .000777542964233
 .00179805053468
 .00200764925582
 .000167723323605
 .00020687808796
 0
 0
 .00040755040755
 .000354329067489

 58
 44
 21
 23
 94
 58
 110
 91
 131
 83
 44
 78
 209
 179
 15
 17
 17
 17
 38
 34

 .000635686102587
 .000486634150658
 .000208834702361
 .00025665632602
 .00100410186293
 .00105490987796
 .00146813480147
 .00122986268786
 .00141774891775
 .000893857155165
 .000513251213139
 .000777542964233
 .00179805053468
 .00200764925582
 .000167723323605
 .00020687808796
 0
 0
 .00040755040755
 .000354329067489

 58
 44
 21
 23
 94
 58
 110
 91
 131
 83
 44
 78
 209
 179
 15
 17
 17
 17
 38
 34

 .000635686102587
 .000486634150658
 .000208834702361
 .00025665632602
 .00100410186293
 .00105490987796
 .00146813480147
 .00122986268786
 .00141774891775
 .000893857155165
 .000513251213139
 .000777542964233
 .00179805053468
 .00200764925582
 .000167723323605
 .00020687808796
 0
 0
 .00040755040755
 .000354329067489

 58
 44
 21
 23
 94
 58
 110
 91
 131
 83
 44
 78
 209
 179
 15
 17
 17
 17
 38
 34

 .000635686102587
 .000486634150658
 .000208834702361
 .00025665632602
 .00100410186293
 .00105490987796
 .00146813480147
 .00122986268786
 .00141774891775
 .000893857155165
 .000513251213139
 .000777542964233
 .00179805053468
 .00200764925582
 .000167723323605
 .00020687808796
 0
 0
 .00040755040755
 .000354329067489

 58
 44
 21
 23
 94
 58
 110
 91
 131
 83
 44
 78
 209
 179
 15
 17
 17
 17
 38
 34

 .000964489259097
 .00056405322008
 9.94450963623E-06
 5.57948534827E-05
 .000833190907537
 .0012367908914
 .001001001001
 .00113525786572
 .000822510822511
 .000840010338589
 .000466592011945
 .000488456477531
 .000103237351274
 .000112159176303
 .000011181554907
 0
 0
 0
 .0003861003861
 .000416857726458

 88
 51
 1
 5
 78
 68
 75
 84
 76
 78
 40
 49
 12
 10
 1
 1
 1
 1
 36
 40

 .000964489259097
 .00056405322008
 9.94450963623E-06
 5.57948534827E-05
 .000833190907537
 .0012367908914
 .001001001001
 .00113525786572
 .000822510822511
 .000840010338589
 .000466592011945
 .000488456477531
 .000103237351274
 .000112159176303
 .000011181554907
 0
 0
 0
 .0003861003861
 .000416857726458

 88
 51
 1
 5
 78
 68
 75
 84
 76
 78
 40
 49
 12
 10
 1
 1
 1
 1
 36
 40

 .000964489259097
 .00056405322008
 9.94450963623E-06
 5.57948534827E-05
 .000833190907537
 .0012367908914
 .001001001001
 .00113525786572
 .000822510822511
 .000840010338589
 .000466592011945
 .000488456477531
 .000103237351274
 .000112159176303
 .000011181554907
 0
 0
 0
 .0003861003861
 .000416857726458

 88
 51
 1
 5
 78
 68
 75
 84
 76
 78
 40
 49
 12
 10
 1
 1
 1
 1
 36
 40

 .000964489259097
 .00056405322008
 9.94450963623E-06
 5.57948534827E-05
 .000833190907537
 .0012367908914
 .001001001001
 .00113525786572
 .000822510822511
 .000840010338589
 .000466592011945
 .000488456477531
 .000103237351274
 .000112159176303
 .000011181554907
 0
 0
 0
 .0003861003861
 .000416857726458

 88
 51
 1
 5
 78
 68
 75
 84
 76
 78
 40
 49
 12
 10
 1
 1
 1
 1
 36
 40

 .000964489259097
 .00056405322008
 9.94450963623E-06
 5.57948534827E-05
 .000833190907537
 .0012367908914
 .001001001001
 .00113525786572
 .000822510822511
 .000840010338589
 .000466592011945
 .000488456477531
 .000103237351274
 .000112159176303
 .000011181554907
 0
 0
 0
 .0003861003861
 .000416857726458

 88
 51
 1
 5
 78
 68
 75
 84
 76
 78
 40
 49
 12
 10
 1
 1
 1
 1
 36
 40

 .0022577816747
 .00283132596746
 .00197895741761
 .00189702501841
 .0118996752692
 .00796638838872
 .0112245578912
 .0114066385555
 .00779220779221
 .00577237873697
 .00545912653975
 .00579169823358
 .00454244345604
 .00502473109837
 .00242639741482
 .00219047387251
 .000213248333296
 .000141573542736
 .0031317031317
 .00297011130101

 206
 256
 199
 170
 1114
 438
 841
 844
 720
 536
 468
 581
 528
 448
 217
 180
 19
 15
 292
 285

 .0022577816747
 .00283132596746
 .00197895741761
 .00189702501841
 .0118996752692
 .00796638838872
 .0112245578912
 .0114066385555
 .00779220779221
 .00577237873697
 .00545912653975
 .00579169823358
 .00454244345604
 .00502473109837
 .00242639741482
 .00219047387251
 .000213248333296
 .000141573542736
 .0031317031317
 .00297011130101

 206
 256
 199
 170
 1114
 438
 841
 844
 720
 536
 468
 581
 528
 448
 217
 180
 19
 15
 292
 285

 .0022577816747
 .00283132596746
 .00197895741761
 .00189702501841
 .0118996752692
 .00796638838872
 .0112245578912
 .0114066385555
 .00779220779221
 .00577237873697
 .00545912653975
 .00579169823358
 .00454244345604
 .00502473109837
 .00242639741482
 .00219047387251
 .000213248333296
 .000141573542736
 .0031317031317
 .00297011130101

 206
 256
 199
 170
 1114
 438
 841
 844
 720
 536
 468
 581
 528
 448
 217
 180
 19
 15
 292
 285

 .0022577816747
 .00283132596746
 .00197895741761
 .00189702501841
 .0118996752692
 .00796638838872
 .0112245578912
 .0114066385555
 .00779220779221
 .00577237873697
 .00545912653975
 .00579169823358
 .00454244345604
 .00502473109837
 .00242639741482
 .00219047387251
 .000213248333296
 .000141573542736
 .0031317031317
 .00297011130101

 206
 256
 199
 170
 1114
 438
 841
 844
 720
 536
 468
 581
 528
 448
 217
 180
 19
 15
 292
 285

 .0022577816747
 .00283132596746
 .00197895741761
 .00189702501841
 .0118996752692
 .00796638838872
 .0112245578912
 .0114066385555
 .00779220779221
 .00577237873697
 .00545912653975
 .00579169823358
 .00454244345604
 .00502473109837
 .00242639741482
 .00219047387251
 .000213248333296
 .000141573542736
 .0031317031317
 .00297011130101

 206
 256
 199
 170
 1114
 438
 841
 844
 720
 536
 468
 581
 528
 448
 217
 180
 19
 15
 292
 285

 .0442130644454
 .09376555293806
 .033015771992311
 .034749034749048
 .11275850282009
 .11811353012854
 .11791791791797
 .12202670558977
 .11494588744584
 .11037520461784
 .07589119074287
 .07034770126399
 .06370604884848
 .06213618367191
 .027819708608688
 .025032248643094
 1.42315203483908E-02
 1.96598459680298E-02
 .04101244101242
 .04121680770356

 1982.07982151658
 4466.87261146549
 2053.50060240996
 1983.28901734144
 6217.28704054282
 3892.92793347736
 4950.14793435188
 5077.58588990966
 6093.19207230986
 5824.19133574069
 3926.40116815176
 4124.14524585355
 4687.06860229554
 3436.47581227456
 1735.71302250833
 1381.14778804033
 826.137223974961
 1459.5232837257
 2211.44089958072
 2336.58988622019

 .00191801841298
 .00383777386996
 .000815449790171
 .000769968978061
 .00372799521449
 .00327385824194
 .00545879212546
 .00558168450643
 .00732683982684
 .00674162143534
 .00370940649496
 .00336935284501
 .00477472749641
 .00487892416918
 .000726801068957
 .000608464964587
 3.36707894678E-05
 8.49441256418E-05
 .00166237666238
 .0015944808037

 175
 347
 82
 69
 349
 180
 409
 413
 677
 626
 318
 338
 555
 435
 65
 50
 3
 9
 155
 153

 .00191801841298
 .00383777386996
 .000815449790171
 .000769968978061
 .00372799521449
 .00327385824194
 .00545879212546
 .00558168450643
 .00732683982684
 .00674162143534
 .00370940649496
 .00336935284501
 .00477472749641
 .00487892416918
 .000726801068957
 .000608464964587
 3.36707894678E-05
 8.49441256418E-05
 .00166237666238
 .0015944808037

 175
 347
 82
 69
 349
 180
 409
 413
 677
 626
 318
 338
 555
 435
 65
 50
 3
 9
 155
 153

 .00191801841298
 .00383777386996
 .000815449790171
 .000769968978061
 .00372799521449
 .00327385824194
 .00545879212546
 .00558168450643
 .00732683982684
 .00674162143534
 .00370940649496
 .00336935284501
 .00477472749641
 .00487892416918
 .000726801068957
 .000608464964587
 3.36707894678E-05
 8.49441256418E-05
 .00166237666238
 .0015944808037

 175
 347
 82
 69
 349
 180
 409
 413
 677
 626
 318
 338
 555
 435
 65
 50
 3
 9
 155
 153

 .00191801841298
 .00383777386996
 .000815449790171
 .000769968978061
 .00372799521449
 .00327385824194
 .00545879212546
 .00558168450643
 .00732683982684
 .00674162143534
 .00370940649496
 .00336935284501
 .00477472749641
 .00487892416918
 .000726801068957
 .000608464964587
 3.36707894678E-05
 8.49441256418E-05
 .00166237666238
 .0015944808037

 175
 347
 82
 69
 349
 180
 409
 413
 677
 626
 318
 338
 555
 435
 65
 50
 3
 9
 155
 153

 .0294607628233
 .0657177300729
 .0249706636966
 .0268484834959
 .0847291061357
 .0896309634237
 .0857657657658
 .088806898043
 .085303030303
 .0814271560265
 .0576707726764
 .0524143705889
 .0500098935795
 .0482172298927
 .0228998244496
 .0201523596271
 .0110889133314
 .0161299456358
 .0302016302016
 .0307432573263

 2688
 5942
 2511
 2406
 7932
 4928
 6426
 6571
 7882
 7561
 4944
 5258
 5813
 4299
 2048
 1656
 988
 1709
 2816
 2950

 .0294607628233
 .0657177300729
 .0249706636966
 .0268484834959
 .0847291061357
 .0896309634237
 .0857657657658
 .088806898043
 .085303030303
 .0814271560265
 .0576707726764
 .0524143705889
 .0500098935795
 .0482172298927
 .0228998244496
 .0201523596271
 .0110889133314
 .0161299456358
 .0302016302016
 .0307432573263

 2688
 5942
 2511
 2406
 7932
 4928
 6426
 6571
 7882
 7561
 4944
 5258
 5813
 4299
 2048
 1656
 988
 1709
 2816
 2950

 .0294607628233
 .0657177300729
 .0249706636966
 .0268484834959
 .0847291061357
 .0896309634237
 .0857657657658
 .088806898043
 .085303030303
 .0814271560265
 .0576707726764
 .0524143705889
 .0500098935795
 .0482172298927
 .0228998244496
 .0201523596271
 .0110889133314
 .0161299456358
 .0302016302016
 .0307432573263

 2688
 5942
 2511
 2406
 7932
 4928
 6426
 6571
 7882
 7561
 4944
 5258
 5813
 4299
 2048
 1656
 988
 1709
 2816
 2950

 .0294607628233
 .0657177300729
 .0249706636966
 .0268484834959
 .0847291061357
 .0896309634237
 .0857657657658
 .088806898043
 .085303030303
 .0814271560265
 .0576707726764
 .0524143705889
 .0500098935795
 .0482172298927
 .0228998244496
 .0201523596271
 .0110889133314
 .0161299456358
 .0302016302016
 .0307432573263

 2688
 5942
 2511
 2406
 7932
 4928
 6426
 6571
 7882
 7561
 4944
 5258
 5813
 4299
 2048
 1656
 988
 1709
 2816
 2950

 .00822007891276
 .0137916542243
 .00707054635136
 .00689624389046
 .0125940010255
 .0119495825831
 .0182849516183
 .0199075575738
 .0105844155844
 .0105324373223
 .0111865434864
 .0109852864947
 .00608240061254
 .00615753877904
 .00385763644292
 .00373597488257
 .00299670026263
 .00325619148294
 .00740025740026
 .00731585309934

 750
 1247
 711
 618
 1179
 657
 1370
 1473
 978
 978
 959
 1102
 707
 549
 345
 307
 267
 345
 690
 702

 .00822007891276
 .0137916542243
 .00707054635136
 .00689624389046
 .0125940010255
 .0119495825831
 .0182849516183
 .0199075575738
 .0105844155844
 .0105324373223
 .0111865434864
 .0109852864947
 .00608240061254
 .00615753877904
 .00385763644292
 .00373597488257
 .00299670026263
 .00325619148294
 .00740025740026
 .00731585309934

 750
 1247
 711
 618
 1179
 657
 1370
 1473
 978
 978
 959
 1102
 707
 549
 345
 307
 267
 345
 690
 702

 .00822007891276
 .0137916542243
 .00707054635136
 .00689624389046
 .0125940010255
 .0119495825831
 .0182849516183
 .0199075575738
 .0105844155844
 .0105324373223
 .0111865434864
 .0109852864947
 .00608240061254
 .00615753877904
 .00385763644292
 .00373597488257
 .00299670026263
 .00325619148294
 .00740025740026
 .00731585309934

 750
 1247
 711
 618
 1179
 657
 1370
 1473
 978
 978
 959
 1102
 707
 549
 345
 307
 267
 345
 690
 702

 .00822007891276
 .0137916542243
 .00707054635136
 .00689624389046
 .0125940010255
 .0119495825831
 .0182849516183
 .0199075575738
 .0105844155844
 .0105324373223
 .0111865434864
 .0109852864947
 .00608240061254
 .00615753877904
 .00385763644292
 .00373597488257
 .00299670026263
 .00325619148294
 .00740025740026
 .00731585309934

 750
 1247
 711
 618
 1179
 657
 1370
 1473
 978
 978
 959
 1102
 707
 549
 345
 307
 267
 345
 690
 702

 .00461420429636
 .0104183947709
 .00015911215418
 .000234338384627
 .0117074004444
 .0132591258798
 .00840840840841
 .00773056546654
 .0117316017316
 .0116739898337
 .00332446808511
 .00357869133538
 .00283902716003
 .00288249083099
 .000335446647211
 .000535449168837
 .000112235964893
 .000188764723648
 .00174817674818
 .00156321647422

 421
 942
 16
 21
 1096
 729
 630
 572
 1084
 1084
 285
 359
 330
 257
 30
 44
 10
 20
 163
 150

 .00461420429636
 .0104183947709
 .00015911215418
 .000234338384627
 .0117074004444
 .0132591258798
 .00840840840841
 .00773056546654
 .0117316017316
 .0116739898337
 .00332446808511
 .00357869133538
 .00283902716003
 .00288249083099
 .000335446647211
 .000535449168837
 .000112235964893
 .000188764723648
 .00174817674818
 .00156321647422

 421
 942
 16
 21
 1096
 729
 630
 572
 1084
 1084
 285
 359
 330
 257
 30
 44
 10
 20
 163
 150

 .00461420429636
 .0104183947709
 .00015911215418
 .000234338384627
 .0117074004444
 .0132591258798
 .00840840840841
 .00773056546654
 .0117316017316
 .0116739898337
 .00332446808511
 .00357869133538
 .00283902716003
 .00288249083099
 .000335446647211
 .000535449168837
 .000112235964893
 .000188764723648
 .00174817674818
 .00156321647422

 421
 942
 16
 21
 1096
 729
 630
 572
 1084
 1084
 285
 359
 330
 257
 30
 44
 10
 20
 163
 150

 .00461420429636
 .0104183947709
 .00015911215418
 .000234338384627
 .0117074004444
 .0132591258798
 .00840840840841
 .00773056546654
 .0117316017316
 .0116739898337
 .00332446808511
 .00357869133538
 .00283902716003
 .00288249083099
 .000335446647211
 .000535449168837
 .000112235964893
 .000188764723648
 .00174817674818
 .00156321647422

 421
 942
 16
 21
 1096
 729
 630
 572
 1084
 1084
 285
 359
 330
 257
 30
 44
 10
 20
 163
 150

 .001150811047786
 2.2893924815056E-03
 8.73127946060409E-02
 8.59240743633031E-02
 .006163476328828
 5.9475091395214E-03
 .006299632966303
 .005000540598979
 .003419913419913
 .002821573188591
 1.00433930571062E-02
 1.21416324414868E-02
 2.83472560372324E-02
 3.45674581366332E-02
 8.29783189650042E-02
 8.84829751503185E-02
 .163033962603021
 .126925400181185
 .055083655083686
 5.24511234315915E-02

 60.5047619047728
 140.951690821289
 3952.9252847379
 3504.56597402617
 272.431542461009
 154.902140672794
 205.588983050854
 185.399999999998
 107.310126582278
 93.7938931297467
 523.478513356622
 771.064039408739
 2571.90743550884
 2511.95003244677
 3385.6120468938
 3405.60596891631
 6456.19854054612
 5486.90452111903
 2112.81230529658
 2136.54500297966

 .001150811047786
 2.2893924815056E-03
 8.73127946060409E-02
 8.59240743633031E-02
 .006163476328828
 5.9475091395214E-03
 .006299632966303
 .005000540598979
 .003419913419913
 .002821573188591
 1.00433930571062E-02
 1.21416324414868E-02
 2.83472560372324E-02
 3.45674581366332E-02
 8.29783189650042E-02
 8.84829751503185E-02
 .163033962603021
 .126925400181185
 .055083655083686
 5.24511234315915E-02

 60.5047619047728
 140.951690821289
 3952.9252847379
 3504.56597402617
 272.431542461009
 154.902140672794
 205.588983050854
 185.399999999998
 107.310126582278
 93.7938931297467
 523.478513356622
 771.064039408739
 2571.90743550884
 2511.95003244677
 3385.6120468938
 3405.60596891631
 6456.19854054612
 5486.90452111903
 2112.81230529658
 2136.54500297966

 .001150811047786
 .002189853677962
 7.98444678692362E-02
 .0772312361907
 .006131430524692
 .005874756734145
 .00580580580581
 .004635636285
 .003419913419913
 .002821573188591
 .00950681224337
 .01147374297221
 2.70567891463123E-02
 3.33673549501909E-02
 7.34963604038422E-02
 7.76157908827835E-02
 .143168196817
 .106378360012065
 .04730802230805
 .0457709783651

 60.5047619047728
 147.090909090936
 4265.09440777172
 3828.5139430722
 273.846689895474
 156.795665634686
 221.170114942498
 198.860058309014
 107.310126582278
 93.7938931297467
 551.809815950983
 814.023457862563
 2689.48585055687
 2599.76100840357
 3741.96942035616
 3781.58513640497
 7113.5841956715
 6138.80729305344
 2360.53253230629
 2369.91803278614

 .000350723366944
 .00185805766615
 .0499114938642
 .0512085165264
 .00324730815245
 .00312835343119
 .00339005672339
 .00324359390204
 .000919913419913
 .000613853708969
 .0075821201941
 .00943020056621
 .0249318203326
 .0311129555065
 .0504847204052
 .0555528512668
 .0472737884128
 .037252718212
 .029954954955
 .0293259410563

 32
 168
 5019
 4589
 304
 172
 254
 240
 85
 57
 650
 946
 2898
 2774
 4515
 4565
 4212
 3947
 2793
 2814

 .000350723366944
 .00185805766615
 .0499114938642
 .0512085165264
 .00324730815245
 .00312835343119
 .00339005672339
 .00324359390204
 .000919913419913
 .000613853708969
 .0075821201941
 .00943020056621
 .0249318203326
 .0311129555065
 .0504847204052
 .0555528512668
 .0472737884128
 .037252718212
 .029954954955
 .0293259410563

 32
 168
 5019
 4589
 304
 172
 254
 240
 85
 57
 650
 946
 2898
 2774
 4515
 4565
 4212
 3947
 2793
 2814

 .000800087680842
 .000331796011812
 2.99329740050362E-02
 .0260227196643
 .002884122372242
 .002746403302955
 .00241574908242
 .00139204238296
 .0025
 .002207719479622
 .00192469204927
 .002043542406
 2.1249688137123E-03
 2.2543994436909E-03
 2.30116399986422E-02
 2.20629396159835E-02
 .0958944084042
 6.91256418000648E-02
 .01735306735305
 .0164450373088

 73
 30
 3008.00066445183
 2332
 239.896296296306
 139.476821192024
 175.09944751382
 103
 115.519480519476
 104.024390243887
 165
 205
 243.032388663961
 195.089552238807
 2046.03498542273
 1809.00441257584
 8544
 7320.00109229929
 1614.00494437577
 1578

 .000800087680842
 .000331796011812
 .0299230294954
 .0260227196643
 .00271321141685
 .00263727469489
 .00237570904238
 .00139204238296
 .00123376623377
 .000969242698372
 .00192469204927
 .002043542406
 .0021077625885
 .0022207516908
 .0229445506692
 .0220386010174
 .0958944084042
 .0691067653277
 .0173316173316
 .0164450373088

 73
 30
 3009
 2332
 254
 145
 178
 103
 114
 90
 165
 205
 245
 198
 2052
 1811
 8544
 7322
 1616
 1578

 0
 0
 9.94450963623E-06
 0
 .000170910955392
 .000109128608065
 .00004004004004
 0
 .00126623376623
 .00123847678125
 0
 0
 1.72062252123E-05
 3.36477528909E-05
 6.70893294422E-05
 2.43385985835E-05
 0
 1.88764723648E-05
 .00002145002145

 0
 0
 1
 1
 16
 6
 3
 3
 117
 115
 115
 115
 2
 3
 6
 2
 2
 2
 2

 0
 9.95388035436E-05
 7.4683267368047E-03
 8.6928381726031E-03
 .000032045804136
 7.27524053764E-05
 .000493827160493
 .000364904313979
 0
 0
 5.365808137362E-04
 6.3798397065245E-04
 1.29046689092014E-03
 1.2001031864423E-03
 .009470777006255
 .010867184267535
 1.98433185930421E-02
 2.05187254605731E-02
 .007764907764911
 6.6488807370072E-03

 0
 5.88888888888889
 615.500665778936
 626.460847240018
 1.66666666666667
 2
 22.4054054054138
 14.4074074074102
 14.4074074074102
 14.4074074074102
 21.521739130432
 34.4687500000107
 106.67999999995
 70.4766355140003
 624.157024793448
 720.281075027977
 1720.50678733037
 2114.71297148108
 606.483425414363
 540.034482758607

 0
 2.21197341208E-05
 .007020823803174
 .008079094784293
 .000021363869424
 3.63762026882E-05
 .00036036036036
 .000229754568061
 0
 0
 .000221631205674
 .00020933849037
 .00106678596316
 .000942137080945
 .008531526394065
 .010124857010738
 .0195739522773
 .0202355783751
 .007110682110685
 6.1278085789343E-03

 0
 2
 652.220963172829
 670.165745856357
 2
 2
 27
 17
 17
 17
 19
 21
 124
 84
 683.623853211035
 768.617788461526
 1744
 2144
 657.027149321271
 582.030612244904

 0
 2.21197341208E-05
 .007020823803174
 .008079094784293
 .000021363869424
 3.63762026882E-05
 .00036036036036
 .000229754568061
 0
 0
 .000221631205674
 .00020933849037
 .00106678596316
 .000942137080945
 .008531526394065
 .010124857010738
 .0195739522773
 .0202355783751
 .007110682110685
 6.1278085789343E-03

 0
 2
 652.220963172829
 670.165745856357
 2
 2
 27
 17
 17
 17
 19
 21
 124
 84
 683.623853211035
 768.617788461526
 1744
 2144
 657.027149321271
 582.030612244904

 0
 7.74190694228E-05
 .000417669404722
 .000591425446917
 0
 3.63762026882E-05
 .000133466800133
 .000135149745918
 0
 0
 .000291620007465
 .000418676980741
 .000215077815154
 .000246750187867
 .00093925061219
 .000742327256797
 8.97887719141E-05
 .000235955904561
 .000632775632776
 .00050022927175

 0
 7
 42
 53
 53
 2
 10
 10
 10
 10
 25
 42
 25
 22
 84
 61
 8
 25
 59
 48

 0
 7.74190694228E-05
 .000417669404722
 .000591425446917
 0
 3.63762026882E-05
 .000133466800133
 .000135149745918
 0
 0
 .000291620007465
 .000418676980741
 .000215077815154
 .000246750187867
 .00093925061219
 .000742327256797
 8.97887719141E-05
 .000235955904561
 .000632775632776
 .00050022927175

 0
 7
 42
 53
 53
 2
 10
 10
 10
 10
 25
 42
 25
 22
 84
 61
 8
 25
 59
 48

 0
 0
 2.98335289087E-05
 2.23179413931E-05
 .000010681934712
 0
 0
 0
 0
 0
 2.33296005972E-05
 9.96849954145E-06
 8.60311260614E-06
 1.12159176303E-05
 0
 0
 .000179577543828
 4.71911809121E-05
 .00002145002145
 2.08428863229E-05

 0
 0
 3
 2
 1
 1
 1
 1
 1
 1
 2
 1
 1
 1
 1
 1
 16
 5
 2
 2

 0
 0
 2.98335289087E-05
 2.23179413931E-05
 .000010681934712
 0
 0
 0
 0
 0
 2.33296005972E-05
 9.96849954145E-06
 8.60311260614E-06
 1.12159176303E-05
 0
 0
 .000179577543828
 4.71911809121E-05
 .00002145002145
 2.08428863229E-05

 0
 0
 3
 2
 1
 1
 1
 1
 1
 1
 2
 1
 1
 1
 1
 1
 16
 5
 2
 2

 0
 0
 0
 0
 0
 0
 0
 0
 0
 0
 0
 2.99054986243E-05
 0
 0
 .000011181554907
 0
 2.24471929785E-05
 2.83147085473E-05
 .000010725010725
 3.12643294843E-05

 0
 0
 0
 0
 0
 0
 0
 0
 0
 0
 0
 3
 3
 3
 1
 1
 2
 3
 1
 3

 0
 0
 0
 0
 0
 0
 0
 0
 0
 0
 0
 2.99054986243E-05
 0
 0
 .000011181554907
 0
 2.24471929785E-05
 2.83147085473E-05
 .000010725010725
 3.12643294843E-05

 0
 0
 0
 0
 0
 0
 0
 0
 0
 0
 0
 3
 3
 3
 1
 1
 2
 3
 1
 3

 0
 0
 0
 0
 0
 0
 0
 0
 0
 0
 0
 2.99054986243E-05
 0
 0
 .000011181554907
 0
 2.24471929785E-05
 2.83147085473E-05
 .000010725010725
 3.12643294843E-05

 0
 0
 0
 0
 0
 0
 0
 0
 0
 0
 0
 3
 3
 3
 1
 1
 2
 3
 1
 3

 .000021920210434
 7.74190694228E-05
 0
 0
 8.54554776961E-05
 5.45643040323E-05
 6.67334000667E-05
 5.40598983674E-05
 5.41125541126E-05
 .000107693633152
 1.16648002986E-05
 0
 4.30155630307E-05
 7.85114234121E-05
 0
 0
 0
 0
 .000010725010725
 2.08428863229E-05

 2
 7
 7
 7
 8
 3
 5
 4
 5
 10
 1
 1
 5
 7
 7
 7
 7
 7
 1
 2

 .000021920210434

 2

 .000021920210434

 2

 .000021920210434

 2

 .000021920210434

 2

 0
 7.74190694228E-05
 0
 0
 8.54554776961E-05
 5.45643040323E-05
 6.67334000667E-05
 5.40598983674E-05
 5.41125541126E-05
 .000107693633152
 1.16648002986E-05
 0
 4.30155630307E-05
 7.85114234121E-05
 0
 0
 0
 0
 .000010725010725
 2.08428863229E-05

 0
 7
 7
 7
 8
 3
 5
 4
 5
 10
 1
 1
 5
 7
 7
 7
 7
 7
 1
 2

 0
 7.74190694228E-05
 0
 0
 8.54554776961E-05
 5.45643040323E-05
 6.67334000667E-05
 5.40598983674E-05
 5.41125541126E-05
 .000107693633152
 1.16648002986E-05
 0
 4.30155630307E-05
 7.85114234121E-05
 0
 0
 0
 0
 .000010725010725
 2.08428863229E-05

 0
 7
 7
 7
 8
 3
 5
 4
 5
 10
 1
 1
 5
 7
 7
 7
 7
 7
 1
 2

 0
 7.74190694228E-05
 0
 0
 8.54554776961E-05
 5.45643040323E-05
 6.67334000667E-05
 5.40598983674E-05
 5.41125541126E-05
 .000107693633152
 1.16648002986E-05
 0
 4.30155630307E-05
 7.85114234121E-05
 0
 0
 0
 0
 .000010725010725
 2.08428863229E-05

 0
 7
 7
 7
 8
 3
 5
 4
 5
 10
 1
 1
 5
 7
 7
 7
 7
 7
 1
 2

 0
 7.74190694228E-05
 0
 0
 8.54554776961E-05
 5.45643040323E-05
 6.67334000667E-05
 5.40598983674E-05
 5.41125541126E-05
 .000107693633152
 1.16648002986E-05
 0
 4.30155630307E-05
 7.85114234121E-05
 0
 0
 0
 0
 .000010725010725
 2.08428863229E-05

 0
 7
 7
 7
 8
 3
 5
 4
 5
 10
 1
 1
 5
 7
 7
 7
 7
 7
 1
 2

 .00010960105217
 .000154838138846
 9.94450963623E-06
 0
 .000277730302512
 .000181881013441
 .000373707040374
 .000351389339388
 .000465367965368
 .000527698802447
 .000093318402389
 6.97794967901E-05
 .000404346292489
 .0003701252818
 0
 0
 0
 1.88764723648E-05
 0
 1.04214431614E-05

 10
 14
 1
 1
 26
 10
 28
 26
 43
 49
 8
 7
 47
 33
 33
 33
 33
 2
 2
 1

 .00010960105217
 .000154838138846
 9.94450963623E-06
 0
 .000277730302512
 .000181881013441
 .000373707040374
 .000351389339388
 .000465367965368
 .000527698802447
 .000093318402389
 6.97794967901E-05
 .000404346292489
 .0003701252818
 0
 0
 0
 1.88764723648E-05
 0
 1.04214431614E-05

 10
 14
 1
 1
 26
 10
 28
 26
 43
 49
 8
 7
 47
 33
 33
 33
 33
 2
 2
 1

 .00010960105217
 .000154838138846
 9.94450963623E-06
 0
 .000277730302512
 .000181881013441
 .000373707040374
 .000351389339388
 .000465367965368
 .000527698802447
 .000093318402389
 6.97794967901E-05
 .000404346292489
 .0003701252818
 0
 0
 0
 1.88764723648E-05
 0
 1.04214431614E-05

 10
 14
 1
 1
 26
 10
 28
 26
 43
 49
 8
 7
 47
 33
 33
 33
 33
 2
 2
 1

 .00010960105217
 .000154838138846
 9.94450963623E-06
 0
 .000277730302512
 .000181881013441
 .000373707040374
 .000351389339388
 .000465367965368
 .000527698802447
 .000093318402389
 6.97794967901E-05
 .000404346292489
 .0003701252818
 0
 0
 0
 1.88764723648E-05
 0
 1.04214431614E-05

 10
 14
 1
 1
 26
 10
 28
 26
 43
 49
 8
 7
 47
 33
 33
 33
 33
 2
 2
 1

 .00010960105217
 .000154838138846
 9.94450963623E-06
 0
 .000277730302512
 .000181881013441
 .000373707040374
 .000351389339388
 .000465367965368
 .000527698802447
 .000093318402389
 6.97794967901E-05
 .000404346292489
 .0003701252818
 0
 0
 0
 1.88764723648E-05
 0
 1.04214431614E-05

 10
 14
 1
 1
 26
 10
 28
 26
 43
 49
 8
 7
 47
 33
 33
 33
 33
 2
 2
 1

 .000372643577378
 .000353915745933
 .019600628493
 .0205771419644
 .00123910442659
 .00101853367527
 .001001001001
 .00129743756082
 .000335497835498
 .000247695356251
 .00104983202688
 .00102675545277
 .0149522097095
 .0184614004195
 .0830342267396
 .0862681626792
 .0149161597342
 .00626698882513
 .00735735735736
 .00727416732669

 34
 32
 1971
 1844
 116
 56
 75
 96
 31
 23
 90
 103
 1738
 1646
 7426
 7089
 1329
 664
 686
 698

 .000372643577378
 .000353915745933
 .019600628493
 .0205771419644
 .00123910442659
 .00101853367527
 .001001001001
 .00129743756082
 .000335497835498
 .000247695356251
 .00104983202688
 .00102675545277
 .0149522097095
 .0184614004195
 .0830342267396
 .0862681626792
 .0149161597342
 .00626698882513
 .00735735735736
 .00727416732669

 34
 32
 1971
 1844
 116
 56
 75
 96
 31
 23
 90
 103
 1738
 1646
 7426
 7089
 1329
 664
 686
 698

 .000372643577378
 .000353915745933
 .019600628493
 .0205771419644
 .00123910442659
 .00101853367527
 .001001001001
 .00129743756082
 .000335497835498
 .000247695356251
 .00104983202688
 .00102675545277
 .0149522097095
 .0184614004195
 .0830342267396
 .0862681626792
 .0149161597342
 .00626698882513
 .00735735735736
 .00727416732669

 34
 32
 1971
 1844
 116
 56
 75
 96
 31
 23
 90
 103
 1738
 1646
 7426
 7089
 1329
 664
 686
 698

 .000372643577378
 .000353915745933
 .019600628493
 .0205771419644
 .00123910442659
 .00101853367527
 .001001001001
 .00129743756082
 .000335497835498
 .000247695356251
 .00104983202688
 .00102675545277
 .0149522097095
 .0184614004195
 .0830342267396
 .0862681626792
 .0149161597342
 .00626698882513
 .00735735735736
 .00727416732669

 34
 32
 1971
 1844
 116
 56
 75
 96
 31
 23
 90
 103
 1738
 1646
 7426
 7089
 1329
 664
 686
 698

 .000372643577378
 .000353915745933
 .019600628493
 .0205771419644
 .00123910442659
 .00101853367527
 .001001001001
 .00129743756082
 .000335497835498
 .000247695356251
 .00104983202688
 .00102675545277
 .0149522097095
 .0184614004195
 .0830342267396
 .0862681626792
 .0149161597342
 .00626698882513
 .00735735735736
 .00727416732669

 34
 32
 1971
 1844
 116
 56
 75
 96
 31
 23
 90
 103
 1738
 1646
 7426
 7089
 1329
 664
 686
 698

 .000241122314774
 .00028755654357
 .000556892539629
 .000401722945076
 .000630234148009
 .000491078736291
 .000467133800467
 .000567628932858
 .00104978354978
 .000689239252175
 .000384938409854
 .000219306989912
 .000559202319399
 .00038134119943
 .000458443751188
 .000535449168837
 .00075198096478
 .000528541226216
 .000675675675676
 .000531493601234

 22
 26
 56
 36
 59
 27
 35
 42
 97
 64
 33
 22
 65
 34
 41
 44
 67
 56
 63
 51

 .000241122314774
 .00028755654357
 .000556892539629
 .000401722945076
 .000630234148009
 .000491078736291
 .000467133800467
 .000567628932858
 .00104978354978
 .000689239252175
 .000384938409854
 .000219306989912
 .000559202319399
 .00038134119943
 .000458443751188
 .000535449168837
 .00075198096478
 .000528541226216
 .000675675675676
 .000531493601234

 22
 26
 56
 36
 59
 27
 35
 42
 97
 64
 33
 22
 65
 34
 41
 44
 67
 56
 63
 51

 .000241122314774
 .00028755654357
 .000556892539629
 .000401722945076
 .000630234148009
 .000491078736291
 .000467133800467
 .000567628932858
 .00104978354978
 .000689239252175
 .000384938409854
 .000219306989912
 .000559202319399
 .00038134119943
 .000458443751188
 .000535449168837
 .00075198096478
 .000528541226216
 .000675675675676
 .000531493601234

 22
 26
 56
 36
 59
 27
 35
 42
 97
 64
 33
 22
 65
 34
 41
 44
 67
 56
 63
 51

 .000241122314774
 .00028755654357
 .000556892539629
 .000401722945076
 .000630234148009
 .000491078736291
 .000467133800467
 .000567628932858
 .00104978354978
 .000689239252175
 .000384938409854
 .000219306989912
 .000559202319399
 .00038134119943
 .000458443751188
 .000535449168837
 .00075198096478
 .000528541226216
 .000675675675676
 .000531493601234

 22
 26
 56
 36
 59
 27
 35
 42
 97
 64
 33
 22
 65
 34
 41
 44
 67
 56
 63
 51

 .000241122314774
 .00028755654357
 .000556892539629
 .000401722945076
 .000630234148009
 .000491078736291
 .000467133800467
 .000567628932858
 .00104978354978
 .000689239252175
 .000384938409854
 .000219306989912
 .000559202319399
 .00038134119943
 .000458443751188
 .000535449168837
 .00075198096478
 .000528541226216
 .000675675675676
 .000531493601234

 22
 26
 56
 36
 59
 27
 35
 42
 97
 64
 33
 22
 65
 34
 41
 44
 67
 56
 63
 51

 .000657606313021
 .000940088700134
 9.94450963623E-06
 1.11589706965E-05
 .0032045804136
 .00305560102581
 .00204204204204
 .00262190507082
 .0029329004329
 .00238002929267
 .000221631205674
 .000279117987161
 .00145392603044
 .00142442153905
 6.70893294422E-05
 .000048677197167
 2.24471929785E-05
 .000066067653277
 9.65250965251E-05
 .000104214431614

 60
 85
 1
 1
 300
 168
 153
 194
 271
 221
 19
 28
 169
 127
 6
 4
 2
 7
 9
 10

 .000657606313021
 .000940088700134
 9.94450963623E-06
 1.11589706965E-05
 .0032045804136
 .00305560102581
 .00204204204204
 .00262190507082
 .0029329004329
 .00238002929267
 .000221631205674
 .000279117987161
 .00145392603044
 .00142442153905
 6.70893294422E-05
 .000048677197167
 2.24471929785E-05
 .000066067653277
 9.65250965251E-05
 .000104214431614

 60
 85
 1
 1
 300
 168
 153
 194
 271
 221
 19
 28
 169
 127
 6
 4
 2
 7
 9
 10

 .000657606313021
 .000940088700134
 9.94450963623E-06
 1.11589706965E-05
 .0032045804136
 .00305560102581
 .00204204204204
 .00262190507082
 .0029329004329
 .00238002929267
 .000221631205674
 .000279117987161
 .00145392603044
 .00142442153905
 6.70893294422E-05
 .000048677197167
 2.24471929785E-05
 .000066067653277
 9.65250965251E-05
 .000104214431614

 60
 85
 1
 1
 300
 168
 153
 194
 271
 221
 19
 28
 169
 127
 6
 4
 2
 7
 9
 10

 .000657606313021
 .000940088700134
 9.94450963623E-06
 1.11589706965E-05
 .0032045804136
 .00305560102581
 .00204204204204
 .00262190507082
 .0029329004329
 .00238002929267
 .000221631205674
 .000279117987161
 .00145392603044
 .00142442153905
 6.70893294422E-05
 .000048677197167
 2.24471929785E-05
 .000066067653277
 9.65250965251E-05
 .000104214431614

 60
 85
 1
 1
 300
 168
 153
 194
 271
 221
 19
 28
 169
 127
 6
 4
 2
 7
 9
 10

 .000657606313021
 .000940088700134
 9.94450963623E-06
 1.11589706965E-05
 .0032045804136
 .00305560102581
 .00204204204204
 .00262190507082
 .0029329004329
 .00238002929267
 .000221631205674
 .000279117987161
 .00145392603044
 .00142442153905
 6.70893294422E-05
 .000048677197167
 2.24471929785E-05
 .000066067653277
 9.65250965251E-05
 .000104214431614

 60
 85
 1
 1
 300
 168
 153
 194
 271
 221
 19
 28
 169
 127
 6
 4
 2
 7
 9
 10

 .000504164839982
 .000376035480054
 .000179001173452
 .000145066619055
 .00315117074004
 .00331023444463
 .00257590924258
 .00352740836847
 .000530303030303
 .000333850262772
 .00153975363942
 .00150524343076
 .00291645517348
 .00348815038302
 .000257175762862
 .000194708788668
 0
 0
 .0002145002145
 .000229271749552

 46
 34
 18
 13
 295
 182
 193
 261
 49
 31
 132
 151
 339
 311
 23
 16
 16
 16
 20
 22

 .000504164839982
 .000376035480054
 .000179001173452
 .000145066619055
 .00315117074004
 .00331023444463
 .00257590924258
 .00352740836847
 .000530303030303
 .000333850262772
 .00153975363942
 .00150524343076
 .00291645517348
 .00348815038302
 .000257175762862
 .000194708788668
 0
 0
 .0002145002145
 .000229271749552

 46
 34
 18
 13
 295
 182
 193
 261
 49
 31
 132
 151
 339
 311
 23
 16
 16
 16
 20
 22

 .000504164839982
 .000376035480054
 .000179001173452
 .000145066619055
 .00315117074004
 .00331023444463
 .00257590924258
 .00352740836847
 .000530303030303
 .000333850262772
 .00153975363942
 .00150524343076
 .00291645517348
 .00348815038302
 .000257175762862
 .000194708788668
 0
 0
 .0002145002145
 .000229271749552

 46
 34
 18
 13
 295
 182
 193
 261
 49
 31
 132
 151
 339
 311
 23
 16
 16
 16
 20
 22

 .000504164839982
 .000376035480054
 .000179001173452
 .000145066619055
 .00315117074004
 .00331023444463
 .00257590924258
 .00352740836847
 .000530303030303
 .000333850262772
 .00153975363942
 .00150524343076
 .00291645517348
 .00348815038302
 .000257175762862
 .000194708788668
 0
 0
 .0002145002145
 .000229271749552

 46
 34
 18
 13
 295
 182
 193
 261
 49
 31
 132
 151
 339
 311
 23
 16
 16
 16
 20
 22

 .000504164839982
 .000376035480054
 .000179001173452
 .000145066619055
 .00315117074004
 .00331023444463
 .00257590924258
 .00352740836847
 .000530303030303
 .000333850262772
 .00153975363942
 .00150524343076
 .00291645517348
 .00348815038302
 .000257175762862
 .000194708788668
 0
 0
 .0002145002145
 .000229271749552

 46
 34
 18
 13
 295
 182
 193
 261
 49
 31
 132
 151
 339
 311
 23
 16
 16
 16
 20
 22

 .000591845681719
 .00163686032494
 .000228723721633
 .000100430736269
 .00271321141685
 .00250995798549
 .00286953620287
 .00339225862255
 .00386363636364
 .0036723528905
 .00159807764091
 .00154511742892
 .00309712053821
 .00317410468938
 .000368991311932
 .000292063183002
 0
 0
 .000139425139425
 .000250114635875

 54
 148
 23
 9
 254
 138
 215
 251
 357
 341
 137
 155
 360
 283
 33
 24
 24
 24
 13
 24

 .000591845681719
 .00163686032494
 .000228723721633
 .000100430736269
 .00271321141685
 .00250995798549
 .00286953620287
 .00339225862255
 .00386363636364
 .0036723528905
 .00159807764091
 .00154511742892
 .00309712053821
 .00317410468938
 .000368991311932
 .000292063183002
 0
 0
 .000139425139425
 .000250114635875

 54
 148
 23
 9
 254
 138
 215
 251
 357
 341
 137
 155
 360
 283
 33
 24
 24
 24
 13
 24

 .000591845681719
 .00163686032494
 .000228723721633
 .000100430736269
 .00271321141685
 .00250995798549
 .00286953620287
 .00339225862255
 .00386363636364
 .0036723528905
 .00159807764091
 .00154511742892
 .00309712053821
 .00317410468938
 .000368991311932
 .000292063183002
 0
 0
 .000139425139425
 .000250114635875

 54
 148
 23
 9
 254
 138
 215
 251
 357
 341
 137
 155
 360
 283
 33
 24
 24
 24
 13
 24

 .000591845681719
 .00163686032494
 .000228723721633
 .000100430736269
 .00271321141685
 .00250995798549
 .00286953620287
 .00339225862255
 .00386363636364
 .0036723528905
 .00159807764091
 .00154511742892
 .00309712053821
 .00317410468938
 .000368991311932
 .000292063183002
 0
 0
 .000139425139425
 .000250114635875

 54
 148
 23
 9
 254
 138
 215
 251
 357
 341
 137
 155
 360
 283
 33
 24
 24
 24
 13
 24

 .000591845681719
 .00163686032494
 .000228723721633
 .000100430736269
 .00271321141685
 .00250995798549
 .00286953620287
 .00339225862255
 .00386363636364
 .0036723528905
 .00159807764091
 .00154511742892
 .00309712053821
 .00317410468938
 .000368991311932
 .000292063183002
 0
 0
 .000139425139425
 .000250114635875

 54
 148
 23
 9
 254
 138
 215
 251
 357
 341
 137
 155
 360
 283
 33
 24
 24
 24
 13
 24

 4.8443665059201E-03
 1.13695433380864E-02
 4.04642097098551E-02
 4.39663445443782E-02
 .017828149034352
 2.14619595859943E-02
 1.90990990991204E-02
 2.12049951346472E-02
 1.30627705627676E-02
 1.25355388989372E-02
 1.94685516984118E-02
 2.02659595677786E-02
 2.67728864303361E-02
 2.62115995020146E-02
 5.84348059441322E-02
 6.74179180762762E-02
 2.61509798200093E-02
 3.81871035940745E-02
 4.84984984985052E-02
 5.07211638668684E-02

 168.968325791905
 536.19066147863
 1703.93831408233
 1642.63553299521
 737.275614140395
 571.510169491241
 681.371767994585
 743.275334608218
 556.608947804534
 613.467353952055
 780.454763331392
 949.861780619931
 1540.6908740365
 1166.14249037223
 2593.52544967562
 2777.09241877315
 1073.19914163121
 1445.59614434002
 2126.19371959288
 2279.59174029181

 .000021920210434
 1.10598670604E-05
 4.97225481811E-05
 2.23179413931E-05
 .00021363869424
 .000127316709409
 5.33867200534E-05
 1.35149745918E-05
 .00038961038961
 .000172309813044
 0
 9.96849954145E-06
 .00022368092776
 .000246750187867
 5.59077745351E-05
 1.21692992917E-05
 0
 9.43823618242E-06
 8.58000858001E-05
 3.12643294843E-05

 2
 1
 5
 2
 20
 7
 4
 1
 36
 16
 16
 1
 26
 22
 5
 1
 1
 1
 8
 3

 .000021920210434
 1.10598670604E-05
 4.97225481811E-05
 2.23179413931E-05
 .00021363869424
 .000127316709409
 5.33867200534E-05
 1.35149745918E-05
 .00038961038961
 .000172309813044
 0
 9.96849954145E-06
 .00022368092776
 .000246750187867
 5.59077745351E-05
 1.21692992917E-05
 0
 9.43823618242E-06
 8.58000858001E-05
 3.12643294843E-05

 2
 1
 5
 2
 20
 7
 4
 1
 36
 16
 16
 1
 26
 22
 5
 1
 1
 1
 8
 3

 .000021920210434
 1.10598670604E-05
 4.97225481811E-05
 2.23179413931E-05
 .00021363869424
 .000127316709409
 5.33867200534E-05
 1.35149745918E-05
 .00038961038961
 .000172309813044
 0
 9.96849954145E-06
 .00022368092776
 .000246750187867
 5.59077745351E-05
 1.21692992917E-05
 0
 9.43823618242E-06
 8.58000858001E-05
 3.12643294843E-05

 2
 1
 5
 2
 20
 7
 4
 1
 36
 16
 16
 1
 26
 22
 5
 1
 1
 1
 8
 3

 .000021920210434
 1.10598670604E-05
 4.97225481811E-05
 2.23179413931E-05
 .00021363869424
 .000127316709409
 5.33867200534E-05
 1.35149745918E-05
 .00038961038961
 .000172309813044
 0
 9.96849954145E-06
 .00022368092776
 .000246750187867
 5.59077745351E-05
 1.21692992917E-05
 0
 9.43823618242E-06
 8.58000858001E-05
 3.12643294843E-05

 2
 1
 5
 2
 20
 7
 4
 1
 36
 16
 16
 1
 26
 22
 5
 1
 1
 1
 8
 3

 4.8224462954861E-03
 .011358483471026
 .040414487161674
 4.39440266029851E-02
 .017614510340112
 2.13346428765853E-02
 .019045712379067
 2.11914801600554E-02
 1.26731601731576E-02
 1.23632290858932E-02
 1.94685516984118E-02
 2.02559910682371E-02
 2.65492055025761E-02
 2.59648493141476E-02
 5.83788981695971E-02
 6.74057487769845E-02
 2.61509798200093E-02
 3.81776653578921E-02
 4.84126984127051E-02
 5.06898995373841E-02

 169.727272727322
 536.711781889028
 1706.02854330733
 1643.46876587128
 745.975136446513
 574.878942881229
 683.270497547477
 743.748724489981
 572.614005123875
 621.794425087285
 780.454763331392
 950.328740157637
 1553.45236552223
 1177.01555075592
 2596.00440528726
 2777.59360895527
 1073.19914163121
 1445.95327564888
 2129.94771820979
 2280.99588815794

 .002575624726
 .0078193260117
 .0157819367927
 .018568527239
 .0100410186293
 .0129681162583
 .00742075408742
 .00875770353552
 .00814935064935
 .00864779874214
 .0102533594625
 .0110351289924
 .00794927604807
 .00780627867069
 .0201044357228
 .0222089712074
 .0147702529799
 .0154409543944
 .0162912912913
 .0172058026596

 235
 707
 1587
 1664
 940
 713
 556
 648
 753
 803
 879
 1107
 924
 696
 1798
 1825
 1316
 1636
 1519
 1651

 .002575624726
 .0078193260117
 .0157819367927
 .018568527239
 .0100410186293
 .0129681162583
 .00742075408742
 .00875770353552
 .00814935064935
 .00864779874214
 .0102533594625
 .0110351289924
 .00794927604807
 .00780627867069
 .0201044357228
 .0222089712074
 .0147702529799
 .0154409543944
 .0162912912913
 .0172058026596

 235
 707
 1587
 1664
 940
 713
 556
 648
 753
 803
 879
 1107
 924
 696
 1798
 1825
 1316
 1636
 1519
 1651

 .002575624726
 .0078193260117
 .0157819367927
 .018568527239
 .0100410186293
 .0129681162583
 .00742075408742
 .00875770353552
 .00814935064935
 .00864779874214
 .0102533594625
 .0110351289924
 .00794927604807
 .00780627867069
 .0201044357228
 .0222089712074
 .0147702529799
 .0154409543944
 .0162912912913
 .0172058026596

 235
 707
 1587
 1664
 940
 713
 556
 648
 753
 803
 879
 1107
 924
 696
 1798
 1825
 1316
 1636
 1519
 1651

 .00107409031127
 .00112810644016
 .000109389605999
 2.23179413931E-05
 .00117501281832
 .000782088357796
 .000467133800467
 .000256784517245
 .00116883116883
 .000958473335056
 6.99888017917E-05
 .000109653494956
 .00060221788243
 .000414988952321
 .000402535976653
 .000705819358921
 0
 0
 .0000429000429
 2.08428863229E-05

 98
 102
 11
 2
 110
 43
 35
 19
 108
 89
 6
 11
 70
 37
 36
 58
 58
 58
 4
 2

 .00107409031127
 .00112810644016
 .000109389605999
 2.23179413931E-05
 .00117501281832
 .000782088357796
 .000467133800467
 .000256784517245
 .00116883116883
 .000958473335056
 6.99888017917E-05
 .000109653494956
 .00060221788243
 .000414988952321
 .000402535976653
 .000705819358921
 0
 0
 .0000429000429
 2.08428863229E-05

 98
 102
 11
 2
 110
 43
 35
 19
 108
 89
 6
 11
 70
 37
 36
 58
 58
 58
 4
 2

 .00107409031127
 .00112810644016
 .000109389605999
 2.23179413931E-05
 .00117501281832
 .000782088357796
 .000467133800467
 .000256784517245
 .00116883116883
 .000958473335056
 6.99888017917E-05
 .000109653494956
 .00060221788243
 .000414988952321
 .000402535976653
 .000705819358921
 0
 0
 .0000429000429
 2.08428863229E-05

 98
 102
 11
 2
 110
 43
 35
 19
 108
 89
 6
 11
 70
 37
 36
 58
 58
 58
 4
 2

 .001096010521697
 .002289392481502
 .0240358797908
 .02490682259473
 .006195522132964
 .00736618104436
 .0108775442109
 1.16904530219837E-02
 .00327922077922
 .00266003273886
 .009086879432627
 .009041429084091
 .017713808856073
 .017395888244597
 .03702212829721
 .04338355197511
 .01136950324362
 .02268951978258
 .03157443157443
 .03302555337868

 98.0199999999973
 197.241545893718
 1825.48986346739
 1658.36917562777
 576.01379310345
 405
 815
 861.009248554919
 303
 247
 680.211810012837
 777.804851157678
 1910.75910636241
 1443.04384268213
 3115.16188462709
 3378.17026647955
 758.832181638677
 1319.61730449296
 2481.48437499981
 2640.44335752651

 .000010960105217
 .000055299335302
 .0034308558245
 .00377173209543
 .000021363869424
 0
 0
 2.70299491837E-05
 0
 0
 .000618234415827
 .000697794967901
 .000662439670673
 .000628091387297
 .00112933704561
 .00116825273201
 .0016723158769
 .00779598308668
 .00271342771343
 .00303263995998

 1
 5
 345
 338
 2
 2
 2
 2
 2
 2
 53
 70
 77
 56
 101
 96
 149
 826
 253
 291

 .000010960105217
 .000055299335302
 .0034308558245
 .00377173209543
 .000021363869424
 0
 0
 2.70299491837E-05
 0
 0
 .000618234415827
 .000697794967901
 .000662439670673
 .000628091387297
 .00112933704561
 .00116825273201
 .0016723158769
 .00779598308668
 .00271342771343
 .00303263995998

 1
 5
 345
 338
 2
 2
 2
 2
 2
 2
 53
 70
 77
 56
 101
 96
 149
 826
 253
 291

 .00108505041648
 .0022340931462
 .0206050239663
 .0211350904993
 .00617415826354
 .00736618104436
 .0108775442109
 .0116634230728
 .00327922077922
 .00266003273886
 .0084686450168
 .00834363411619
 .0170513691854
 .0167677968573
 .0358927912516
 .0422152992431
 .00969718736672
 .0148935366959
 .028861003861
 .0299929134187

 99
 202
 2072
 1894
 578
 405
 815
 863
 303
 247
 726
 837
 1982
 1495
 3210
 3469
 864
 1578
 2691
 2878

 .00108505041648
 .0022340931462
 .0206050239663
 .0211350904993
 .00617415826354
 .00736618104436
 .0108775442109
 .0116634230728
 .00327922077922
 .00266003273886
 .0084686450168
 .00834363411619
 .0170513691854
 .0167677968573
 .0358927912516
 .0422152992431
 .00969718736672
 .0148935366959
 .028861003861
 .0299929134187

 99
 202
 2072
 1894
 578
 405
 815
 863
 303
 247
 726
 837
 1982
 1495
 3210
 3469
 864
 1578
 2691
 2878

 7.67207365191E-05
 .000121658537664
 0
 0
 .000192274824816
 .000163692912097
 .00028028028028
 .000459509136123
 7.57575757576E-05
 9.69242698372E-05
 1.16648002986E-05
 1.99369990829E-05
 8.60311260614E-06
 2.24318352606E-05
 3.35446647211E-05
 2.43385985835E-05
 0
 0
 6.43500643501E-05
 5.21072158072E-05

 7
 11
 11
 11
 18
 9
 21
 34
 7
 9
 1
 2
 1
 2
 3
 2
 2
 2
 6
 5

 7.67207365191E-05
 .000121658537664
 0
 0
 .000192274824816
 .000163692912097
 .00028028028028
 .000459509136123
 7.57575757576E-05
 9.69242698372E-05
 1.16648002986E-05
 1.99369990829E-05
 8.60311260614E-06
 2.24318352606E-05
 3.35446647211E-05
 2.43385985835E-05
 0
 0
 6.43500643501E-05
 5.21072158072E-05

 7
 11
 11
 11
 18
 9
 21
 34
 7
 9
 1
 2
 1
 2
 3
 2
 2
 2
 6
 5

 7.67207365191E-05
 .000121658537664
 0
 0
 .000192274824816
 .000163692912097
 .00028028028028
 .000459509136123
 7.57575757576E-05
 9.69242698372E-05
 1.16648002986E-05
 1.99369990829E-05
 8.60311260614E-06
 2.24318352606E-05
 3.35446647211E-05
 2.43385985835E-05
 0
 0
 6.43500643501E-05
 5.21072158072E-05

 7
 11
 11
 11
 18
 9
 21
 34
 7
 9
 1
 2
 1
 2
 3
 2
 2
 2
 6
 5

 0
 0
 .000487280972175
 .000446358827862
 .000010681934712
 5.45643040323E-05
 0
 2.70299491837E-05
 0
 0
 4.66592011945E-05
 4.98424977072E-05
 .000275299603397
 .000325261611279
 .000816253508213
 .00108306763697
 1.12235964893E-05
 4.71911809121E-05
 .000439725439725
 .000385593396974

 0
 0
 49
 40
 1
 3
 3
 2
 2
 2
 4
 5
 32
 29
 73
 89
 1
 5
 41
 37

 0
 0
 .000487280972175
 .000446358827862
 .000010681934712
 5.45643040323E-05
 0
 2.70299491837E-05
 0
 0
 4.66592011945E-05
 4.98424977072E-05
 .000275299603397
 .000325261611279
 .000816253508213
 .00108306763697
 1.12235964893E-05
 4.71911809121E-05
 .000439725439725
 .000385593396974

 0
 0
 49
 40
 1
 3
 3
 2
 2
 2
 4
 5
 32
 29
 73
 89
 1
 5
 41
 37

 0
 0
 .000487280972175
 .000446358827862
 .000010681934712
 5.45643040323E-05
 0
 2.70299491837E-05
 0
 0
 4.66592011945E-05
 4.98424977072E-05
 .000275299603397
 .000325261611279
 .000816253508213
 .00108306763697
 1.12235964893E-05
 4.71911809121E-05
 .000439725439725
 .000385593396974

 0
 0
 49
 40
 1
 3
 3
 2
 2
 2
 4
 5
 32
 29
 73
 89
 1
 5
 41
 37

 .00261946514687
 .0043575876218
 .000706060184172
 .000524471622738
 .00440095710135
 .00534730179517
 .00351017684351
 .00310844415612
 .0119588744589
 .00857241319893
 .00241461366181
 .00217313290004
 .00241747464233
 .00214224026739
 .00269475473259
 .00209311947818
 .000628521403399
 .00128360012081
 .00134062634063
 .00136520905415

 239
 394
 71
 47
 412
 294
 263
 230
 1105
 796
 207
 218
 281
 191
 241
 172
 56
 136
 125
 131

 .00261946514687
 .0043575876218
 .000706060184172
 .000524471622738
 .00440095710135
 .00534730179517
 .00351017684351
 .00310844415612
 .0119588744589
 .00857241319893
 .00241461366181
 .00217313290004
 .00241747464233
 .00214224026739
 .00269475473259
 .00209311947818
 .000628521403399
 .00128360012081
 .00134062634063
 .00136520905415

 239
 394
 71
 47
 412
 294
 263
 230
 1105
 796
 207
 218
 281
 191
 241
 172
 56
 136
 125
 131

 .00261946514687
 .0043575876218
 .000706060184172
 .000524471622738
 .00440095710135
 .00534730179517
 .00351017684351
 .00310844415612
 .0119588744589
 .00857241319893
 .00241461366181
 .00217313290004
 .00241747464233
 .00214224026739
 .00269475473259
 .00209311947818
 .000628521403399
 .00128360012081
 .00134062634063
 .00136520905415

 239
 394
 71
 47
 412
 294
 263
 230
 1105
 796
 207
 218
 281
 191
 241
 172
 56
 136
 125
 131

 .00261946514687
 .0043575876218
 .000706060184172
 .000524471622738
 .00440095710135
 .00534730179517
 .00351017684351
 .00310844415612
 .0119588744589
 .00857241319893
 .00241461366181
 .00217313290004
 .00241747464233
 .00214224026739
 .00269475473259
 .00209311947818
 .000628521403399
 .00128360012081
 .00134062634063
 .00136520905415

 239
 394
 71
 47
 412
 294
 263
 230
 1105
 796
 207
 218
 281
 191
 241
 172
 56
 136
 125
 131

 .00261946514687
 .0043575876218
 .000706060184172
 .000524471622738
 .00440095710135
 .00534730179517
 .00351017684351
 .00310844415612
 .0119588744589
 .00857241319893
 .00241461366181
 .00217313290004
 .00241747464233
 .00214224026739
 .00269475473259
 .00209311947818
 .000628521403399
 .00128360012081
 .00134062634063
 .00136520905415

 239
 394
 71
 47
 412
 294
 263
 230
 1105
 796
 207
 218
 281
 191
 241
 172
 56
 136
 125
 131

 .000032880315651
 7.74190694228E-05
 .000825394299807
 .00112705604035
 .000032045804136
 5.45643040323E-05
 9.34267600934E-05
 .000108119796735
 9.74025974026E-05
 2.15387266305E-05
 .000104983202688
 .000119621994497
 .000412949405095
 .000527148128624
 .00173314101059
 .00227565896756
 .00329973736784
 .00129303835699
 .000482625482625
 .000416857726458

 3
 7
 83
 101
 3
 3
 7
 8
 9
 2
 9
 12
 48
 47
 155
 187
 294
 137
 45
 40

 .000032880315651
 7.74190694228E-05
 .000825394299807
 .00112705604035
 .000032045804136
 5.45643040323E-05
 9.34267600934E-05
 .000108119796735
 9.74025974026E-05
 2.15387266305E-05
 .000104983202688
 .000119621994497
 .000412949405095
 .000527148128624
 .00173314101059
 .00227565896756
 .00329973736784
 .00129303835699
 .000482625482625
 .000416857726458

 3
 7
 83
 101
 3
 3
 7
 8
 9
 2
 9
 12
 48
 47
 155
 187
 294
 137
 45
 40

 .000032880315651
 7.74190694228E-05
 .000825394299807
 .00112705604035
 .000032045804136
 5.45643040323E-05
 9.34267600934E-05
 .000108119796735
 9.74025974026E-05
 2.15387266305E-05
 .000104983202688
 .000119621994497
 .000412949405095
 .000527148128624
 .00173314101059
 .00227565896756
 .00329973736784
 .00129303835699
 .000482625482625
 .000416857726458

 3
 7
 83
 101
 3
 3
 7
 8
 9
 2
 9
 12
 48
 47
 155
 187
 294
 137
 45
 40

 .000032880315651
 7.74190694228E-05
 .000825394299807
 .00112705604035
 .000032045804136
 5.45643040323E-05
 9.34267600934E-05
 .000108119796735
 9.74025974026E-05
 2.15387266305E-05
 .000104983202688
 .000119621994497
 .000412949405095
 .000527148128624
 .00173314101059
 .00227565896756
 .00329973736784
 .00129303835699
 .000482625482625
 .000416857726458

 3
 7
 83
 101
 3
 3
 7
 8
 9
 2
 9
 12
 48
 47
 155
 187
 294
 137
 45
 40

 .000032880315651
 7.74190694228E-05
 .000825394299807
 .00112705604035
 .000032045804136
 5.45643040323E-05
 9.34267600934E-05
 .000108119796735
 9.74025974026E-05
 2.15387266305E-05
 .000104983202688
 .000119621994497
 .000412949405095
 .000527148128624
 .00173314101059
 .00227565896756
 .00329973736784
 .00129303835699
 .000482625482625
 .000416857726458

 3
 7
 83
 101
 3
 3
 7
 8
 9
 2
 9
 12
 48
 47
 155
 187
 294
 137
 45
 40

 .007255589653658
 2.69528960262074E-02
 1.26394717476505E-02
 1.24645702680424E-02
 .037247906340754
 .036303450282819
 .039679679679724
 .045545464374553
 .083841991342023
 .075374773843345
 .050870194102291
 5.44379759958659E-02
 4.07529444152723E-02
 .047319956482266
 1.54976351011362E-02
 .013532260812424
 4.0180475431568E-03
 7.7771066143181E-03
 3.31295581295562E-02
 .031722872983392

 271.220543806617
 1040.71522363569
 568.667977969932
 517.028648164718
 1312.33352451907
 683.602204409036
 1270.96300033714
 1383.30682492602
 4071.80650574459
 3531.26746678135
 1595.44347626619
 1993.59586156363
 1996.85138273134
 1764.34771272863
 562.715728715759
 431.798561151114
 232.564245810024
 495.63834951461
 1382.90806086107
 1371.13272010496

 .000120561157387
 6.63592023624E-05
 .000328168817996
 .000212020443234
 .000672961886857
 .000927593168549
 .000734067400734
 .000743323602552
 .000963203463203
 .000915395881795
 .000641564016424
 .00054826747478
 .000137649801698
 8.97273410424E-05
 8.94524392562E-05
 .000219047387251
 .000426496666592
 .000113258834189
 8.58000858001E-05
 .000114635874776

 11
 6
 33
 19
 63
 51
 55
 55
 89
 85
 55
 55
 16
 8
 8
 18
 38
 12
 8
 11

 .000120561157387
 6.63592023624E-05
 .000328168817996
 .000212020443234
 .000672961886857
 .000927593168549
 .000734067400734
 .000743323602552
 .000963203463203
 .000915395881795
 .000641564016424
 .00054826747478
 .000137649801698
 8.97273410424E-05
 8.94524392562E-05
 .000219047387251
 .000426496666592
 .000113258834189
 8.58000858001E-05
 .000114635874776

 11
 6
 33
 19
 63
 51
 55
 55
 89
 85
 55
 55
 16
 8
 8
 18
 38
 12
 8
 11

 .000120561157387
 6.63592023624E-05
 .000328168817996
 .000212020443234
 .000672961886857
 .000927593168549
 .000734067400734
 .000743323602552
 .000963203463203
 .000915395881795
 .000641564016424
 .00054826747478
 .000137649801698
 8.97273410424E-05
 8.94524392562E-05
 .000219047387251
 .000426496666592
 .000113258834189
 8.58000858001E-05
 .000114635874776

 11
 6
 33
 19
 63
 51
 55
 55
 89
 85
 55
 55
 16
 8
 8
 18
 38
 12
 8
 11

 .000120561157387
 6.63592023624E-05
 .000328168817996
 .000212020443234
 .000672961886857
 .000927593168549
 .000734067400734
 .000743323602552
 .000963203463203
 .000915395881795
 .000641564016424
 .00054826747478
 .000137649801698
 8.97273410424E-05
 8.94524392562E-05
 .000219047387251
 .000426496666592
 .000113258834189
 8.58000858001E-05
 .000114635874776

 11
 6
 33
 19
 63
 51
 55
 55
 89
 85
 55
 55
 16
 8
 8
 18
 38
 12
 8
 11

 .000252082419991
 .000508753884778
 .000904950376897
 .000747651036668
 .00350367458554
 .00314654153253
 .00321654988322
 .00428424694562
 .00427489177489
 .00408158869648
 .00740714818962
 .00723713066709
 .00621144730163
 .00723426687154
 .00122997103977
 .00097354394334
 2.24471929785E-05
 2.83147085473E-05
 .00108322608323
 .000958772770853

 23
 46
 91
 67
 328
 173
 241
 317
 395
 379
 635
 726
 722
 645
 110
 80
 2
 3
 101
 92

 .000252082419991
 .000508753884778
 .000904950376897
 .000747651036668
 .00350367458554
 .00314654153253
 .00321654988322
 .00428424694562
 .00427489177489
 .00408158869648
 .00740714818962
 .00723713066709
 .00621144730163
 .00723426687154
 .00122997103977
 .00097354394334
 2.24471929785E-05
 2.83147085473E-05
 .00108322608323
 .000958772770853

 23
 46
 91
 67
 328
 173
 241
 317
 395
 379
 635
 726
 722
 645
 110
 80
 2
 3
 101
 92

 .000252082419991
 .000508753884778
 .000904950376897
 .000747651036668
 .00350367458554
 .00314654153253
 .00321654988322
 .00428424694562
 .00427489177489
 .00408158869648
 .00740714818962
 .00723713066709
 .00621144730163
 .00723426687154
 .00122997103977
 .00097354394334
 2.24471929785E-05
 2.83147085473E-05
 .00108322608323
 .000958772770853

 23
 46
 91
 67
 328
 173
 241
 317
 395
 379
 635
 726
 722
 645
 110
 80
 2
 3
 101
 92

 .000252082419991
 .000508753884778
 .000904950376897
 .000747651036668
 .00350367458554
 .00314654153253
 .00321654988322
 .00428424694562
 .00427489177489
 .00408158869648
 .00740714818962
 .00723713066709
 .00621144730163
 .00723426687154
 .00122997103977
 .00097354394334
 2.24471929785E-05
 2.83147085473E-05
 .00108322608323
 .000958772770853

 23
 46
 91
 67
 328
 173
 241
 317
 395
 379
 635
 726
 722
 645
 110
 80
 2
 3
 101
 92

 .000460324419114
 .00134930378137
 .000109389605999
 .000178543531145
 .000993419928217
 .00136410760081
 .001001001001
 .000810898475511
 .00159090909091
 .00106616696821
 .000781541620007
 .000637983970653
 .000464568080732
 .000863625657533
 .000100633994163
 .000182539489376
 .000112235964893
 .000066067653277
 .000697125697126
 .000448122055942

 42
 122
 11
 16
 93
 75
 75
 60
 147
 99
 67
 64
 54
 77
 9
 15
 10
 7
 65
 43

 .000460324419114
 .00134930378137
 .000109389605999
 .000178543531145
 .000993419928217
 .00136410760081
 .001001001001
 .000810898475511
 .00159090909091
 .00106616696821
 .000781541620007
 .000637983970653
 .000464568080732
 .000863625657533
 .000100633994163
 .000182539489376
 .000112235964893
 .000066067653277
 .000697125697126
 .000448122055942

 42
 122
 11
 16
 93
 75
 75
 60
 147
 99
 67
 64
 54
 77
 9
 15
 10
 7
 65
 43

 .000460324419114
 .00134930378137
 .000109389605999
 .000178543531145
 .000993419928217
 .00136410760081
 .001001001001
 .000810898475511
 .00159090909091
 .00106616696821
 .000781541620007
 .000637983970653
 .000464568080732
 .000863625657533
 .000100633994163
 .000182539489376
 .000112235964893
 .000066067653277
 .000697125697126
 .000448122055942

 42
 122
 11
 16
 93
 75
 75
 60
 147
 99
 67
 64
 54
 77
 9
 15
 10
 7
 65
 43

 .000460324419114
 .00134930378137
 .000109389605999
 .000178543531145
 .000993419928217
 .00136410760081
 .001001001001
 .000810898475511
 .00159090909091
 .00106616696821
 .000781541620007
 .000637983970653
 .000464568080732
 .000863625657533
 .000100633994163
 .000182539489376
 .000112235964893
 .000066067653277
 .000697125697126
 .000448122055942

 42
 122
 11
 16
 93
 75
 75
 60
 147
 99
 67
 64
 54
 77
 9
 15
 10
 7
 65
 43

 .000416483998246
 .000475574283597
 2.98335289087E-05
 7.81127948758E-05
 .002670483678
 .00252814608683
 .00284284284284
 .00317601902908
 .00294372294372
 .00272464891876
 .00137644643524
 .00126599944176
 .00154856026911
 .00206372884398
 .000245994207955
 .000146031591501
 1.12235964893E-05
 1.88764723648E-05
 .00019305019305
 .000198007420068

 38
 43
 3
 7
 250
 139
 213
 235
 272
 253
 118
 127
 180
 184
 22
 12
 1
 2
 18
 19

 .000416483998246
 .000475574283597
 2.98335289087E-05
 7.81127948758E-05
 .002670483678
 .00252814608683
 .00284284284284
 .00317601902908
 .00294372294372
 .00272464891876
 .00137644643524
 .00126599944176
 .00154856026911
 .00206372884398
 .000245994207955
 .000146031591501
 1.12235964893E-05
 1.88764723648E-05
 .00019305019305
 .000198007420068

 38
 43
 3
 7
 250
 139
 213
 235
 272
 253
 118
 127
 180
 184
 22
 12
 1
 2
 18
 19

 .000416483998246
 .000475574283597
 2.98335289087E-05
 7.81127948758E-05
 .002670483678
 .00252814608683
 .00284284284284
 .00317601902908
 .00294372294372
 .00272464891876
 .00137644643524
 .00126599944176
 .00154856026911
 .00206372884398
 .000245994207955
 .000146031591501
 1.12235964893E-05
 1.88764723648E-05
 .00019305019305
 .000198007420068

 38
 43
 3
 7
 250
 139
 213
 235
 272
 253
 118
 127
 180
 184
 22
 12
 1
 2
 18
 19

 .000416483998246
 .000475574283597
 2.98335289087E-05
 7.81127948758E-05
 .002670483678
 .00252814608683
 .00284284284284
 .00317601902908
 .00294372294372
 .00272464891876
 .00137644643524
 .00126599944176
 .00154856026911
 .00206372884398
 .000245994207955
 .000146031591501
 1.12235964893E-05
 1.88764723648E-05
 .00019305019305
 .000198007420068

 38
 43
 3
 7
 250
 139
 213
 235
 272
 253
 118
 127
 180
 184
 22
 12
 1
 2
 18
 19

 .00424156071898
 .0140792107679
 .00359991248832
 .0035597116522
 .0205734062553
 .0180971608374
 .0244511177845
 .0270569791329
 .0584956709957
 .0509498578444
 .0259075214632
 .0260277523027
 .024398427351
 .0283426238518
 .00815135352722
 .00573173996641
 .000246919122764
 .00179326487466
 .0133848133848
 .0134019759056

 387
 1273
 362
 319
 1926
 995
 1832
 2002
 5405
 4731
 2221
 2611
 2836
 2527
 729
 471
 22
 190
 1248
 1286

 .00424156071898
 .0140792107679
 .00359991248832
 .0035597116522
 .0205734062553
 .0180971608374
 .0244511177845
 .0270569791329
 .0584956709957
 .0509498578444
 .0259075214632
 .0260277523027
 .024398427351
 .0283426238518
 .00815135352722
 .00573173996641
 .000246919122764
 .00179326487466
 .0133848133848
 .0134019759056

 387
 1273
 362
 319
 1926
 995
 1832
 2002
 5405
 4731
 2221
 2611
 2836
 2527
 729
 471
 22
 190
 1248
 1286

 .00424156071898
 .0140792107679
 .00359991248832
 .0035597116522
 .0205734062553
 .0180971608374
 .0244511177845
 .0270569791329
 .0584956709957
 .0509498578444
 .0259075214632
 .0260277523027
 .024398427351
 .0283426238518
 .00815135352722
 .00573173996641
 .000246919122764
 .00179326487466
 .0133848133848
 .0134019759056

 387
 1273
 362
 319
 1926
 995
 1832
 2002
 5405
 4731
 2221
 2611
 2836
 2527
 729
 471
 22
 190
 1248
 1286

 .00424156071898
 .0140792107679
 .00359991248832
 .0035597116522
 .0205734062553
 .0180971608374
 .0244511177845
 .0270569791329
 .0584956709957
 .0509498578444
 .0259075214632
 .0260277523027
 .024398427351
 .0283426238518
 .00815135352722
 .00573173996641
 .000246919122764
 .00179326487466
 .0133848133848
 .0134019759056

 387
 1273
 362
 319
 1926
 995
 1832
 2002
 5405
 4731
 2221
 2611
 2836
 2527
 729
 471
 22
 190
 1248
 1286

 .00176457693994
 .0104736941062
 .00758766085244
 .00765505389783
 .00883396000684
 .0102399010567
 .00743410076743
 .00947399718889
 .0155735930736
 .0156371155337
 .0147559723778
 .0187009051398
 .00797508538589
 .00870355208111
 .00542305412991
 .00609681894517
 .00319872499944
 .00575732407128
 .0176211926212
 .0165075659677

 161
 947
 763
 686
 827
 563
 557
 701
 1439
 1452
 1265
 1876
 927
 776
 485
 501
 285
 610
 1643
 1584

 .00176457693994
 .0104736941062
 .00758766085244
 .00765505389783
 .00883396000684
 .0102399010567
 .00743410076743
 .00947399718889
 .0155735930736
 .0156371155337
 .0147559723778
 .0187009051398
 .00797508538589
 .00870355208111
 .00542305412991
 .00609681894517
 .00319872499944
 .00575732407128
 .0176211926212
 .0165075659677

 161
 947
 763
 686
 827
 563
 557
 701
 1439
 1452
 1265
 1876
 927
 776
 485
 501
 285
 610
 1643
 1584

 .00176457693994
 .0104736941062
 .00758766085244
 .00765505389783
 .00883396000684
 .0102399010567
 .00743410076743
 .00947399718889
 .0155735930736
 .0156371155337
 .0147559723778
 .0187009051398
 .00797508538589
 .00870355208111
 .00542305412991
 .00609681894517
 .00319872499944
 .00575732407128
 .0176211926212
 .0165075659677

 161
 947
 763
 686
 827
 563
 557
 701
 1439
 1452
 1265
 1876
 927
 776
 485
 501
 285
 610
 1643
 1584

 .00176457693994
 .0104736941062
 .00758766085244
 .00765505389783
 .00883396000684
 .0102399010567
 .00743410076743
 .00947399718889
 .0155735930736
 .0156371155337
 .0147559723778
 .0187009051398
 .00797508538589
 .00870355208111
 .00542305412991
 .00609681894517
 .00319872499944
 .00575732407128
 .0176211926212
 .0165075659677

 161
 947
 763
 686
 827
 563
 557
 701
 1439
 1452
 1265
 1876
 927
 776
 485
 501
 285
 610
 1643
 1584

 0
 0
 7.95560770898E-05
 3.34769120896E-05
 0
 0
 0
 0
 0
 0
 0
 1.99369990829E-05
 1.72062252123E-05
 2.24318352606E-05
 .000257175762862
 .000182539489376
 0
 0
 6.43500643501E-05
 .000093792988453

 0
 0
 8
 3
 3
 3
 3
 3
 3
 3
 3
 2
 2
 2
 23
 15
 15
 15
 6
 9

 0
 0
 7.95560770898E-05
 3.34769120896E-05
 0
 0
 0
 0
 0
 0
 0
 1.99369990829E-05
 1.72062252123E-05
 2.24318352606E-05
 .000257175762862
 .000182539489376
 0
 0
 6.43500643501E-05
 .000093792988453

 0
 0
 8
 3
 3
 3
 3
 3
 3
 3
 3
 2
 2
 2
 23
 15
 15
 15
 6
 9

 0
 0
 7.95560770898E-05
 3.34769120896E-05
 0
 0
 0
 0
 0
 0
 0
 1.99369990829E-05
 1.72062252123E-05
 2.24318352606E-05
 .000257175762862
 .000182539489376
 0
 0
 6.43500643501E-05
 .000093792988453

 0
 0
 8
 3
 3
 3
 3
 3
 3
 3
 3
 2
 2
 2
 23
 15
 15
 15
 6
 9

 0
 0
 7.95560770898E-05
 3.34769120896E-05
 0
 0
 0
 0
 0
 0
 0
 1.99369990829E-05
 1.72062252123E-05
 2.24318352606E-05
 .000257175762862
 .000182539489376
 0
 0
 6.43500643501E-05
 .000093792988453

 0
 0
 8
 3
 3
 3
 3
 3
 3
 3
 3
 2
 2
 2
 23
 15
 15
 15
 6
 9

 .005030688294609
 .009633144209602
 8.7412239702474E-03
 8.6035664070362E-03
 .01892838830966
 2.24623051599333E-02
 9.9699699699734E-03
 .010771434749704
 2.24567099567156E-02
 2.10433359179742E-02
 .010031728256809
 .011712986961206
 .012113182549444
 1.28646575219563E-02
 .021490948531305
 .020602623700901
 .00046016745606
 .000877755964965
 .015218790218795
 1.38188336320833E-02

 347.535947712408
 568.359357061041
 441.616609783834
 387.632944228303
 1284.97855530444
 923.905263157904
 380.068273092401
 403.000000000025
 1103.3845783133
 1109.92528147392
 535.7558139535
 754.714042553288
 742.464488636433
 629.359197907483
 947.967741935534
 834.875959834562
 20.6097560975616
 50.4193548387047
 736.756166314248
 678.182503770725

 .000021920210434
 2.21197341208E-05
 0
 0
 .000010681934712
 5.45643040323E-05
 2.66933600267E-05
 8.10898475511E-05
 5.41125541126E-05
 .000301542172827
 0
 0
 0
 1.12159176303E-05
 0
 0
 0
 0
 .000032175032175
 3.12643294843E-05

 2
 2
 2
 2
 1
 3
 2
 6
 5
 28
 28
 28
 28
 1
 1
 1
 1
 1
 3
 3

 .000021920210434
 2.21197341208E-05
 0
 0
 .000010681934712
 5.45643040323E-05
 2.66933600267E-05
 8.10898475511E-05
 5.41125541126E-05
 .000301542172827
 0
 0
 0
 1.12159176303E-05
 0
 0
 0
 0
 .000032175032175
 3.12643294843E-05

 2
 2
 2
 2
 1
 3
 2
 6
 5
 28
 28
 28
 28
 1
 1
 1
 1
 1
 3
 3

 .000021920210434
 2.21197341208E-05
 0
 0
 .000010681934712
 5.45643040323E-05
 2.66933600267E-05
 8.10898475511E-05
 5.41125541126E-05
 .000301542172827
 0
 0
 0
 1.12159176303E-05
 0
 0
 0
 0
 .000032175032175
 3.12643294843E-05

 2
 2
 2
 2
 1
 3
 2
 6
 5
 28
 28
 28
 28
 1
 1
 1
 1
 1
 3
 3

 .000021920210434
 2.21197341208E-05
 0
 0
 .000010681934712
 5.45643040323E-05
 2.66933600267E-05
 8.10898475511E-05
 5.41125541126E-05
 .000301542172827
 0
 0
 0
 1.12159176303E-05
 0
 0
 0
 0
 .000032175032175
 3.12643294843E-05

 2
 2
 2
 2
 1
 3
 2
 6
 5
 28
 28
 28
 28
 1
 1
 1
 1
 1
 3
 3

 .000646646207804
 .00207925500735
 .00374908013286
 .00373825518334
 .00291616817638
 .00303741292446
 .00399065732399
 .00417612714888
 .00784632034632
 .00594468855001
 .00227463605823
 .00248215638582
 .00780302313377
 .00866990432822
 .0101975780752
 .0104047508944
 .000246919122764
 .00031146179402
 .00523380523381
 .00480428529743

 59
 188
 377
 335
 273
 167
 299
 309
 725
 552
 195
 249
 907
 773
 912
 855
 22
 33
 488
 461

 .000646646207804
 .00207925500735
 .00374908013286
 .00373825518334
 .00291616817638
 .00303741292446
 .00399065732399
 .00417612714888
 .00784632034632
 .00594468855001
 .00227463605823
 .00248215638582
 .00780302313377
 .00866990432822
 .0101975780752
 .0104047508944
 .000246919122764
 .00031146179402
 .00523380523381
 .00480428529743

 59
 188
 377
 335
 273
 167
 299
 309
 725
 552
 195
 249
 907
 773
 912
 855
 22
 33
 488
 461

 .000646646207804
 .00207925500735
 .00374908013286
 .00373825518334
 .00291616817638
 .00303741292446
 .00399065732399
 .00417612714888
 .00784632034632
 .00594468855001
 .00227463605823
 .00248215638582
 .00780302313377
 .00866990432822
 .0101975780752
 .0104047508944
 .000246919122764
 .00031146179402
 .00523380523381
 .00480428529743

 59
 188
 377
 335
 273
 167
 299
 309
 725
 552
 195
 249
 907
 773
 912
 855
 22
 33
 488
 461

 .000646646207804
 .00207925500735
 .00374908013286
 .00373825518334
 .00291616817638
 .00303741292446
 .00399065732399
 .00417612714888
 .00784632034632
 .00594468855001
 .00227463605823
 .00248215638582
 .00780302313377
 .00866990432822
 .0101975780752
 .0104047508944
 .000246919122764
 .00031146179402
 .00523380523381
 .00480428529743

 59
 188
 377
 335
 273
 167
 299
 309
 725
 552
 195
 249
 907
 773
 912
 855
 22
 33
 488
 461

 .00432924156072
 .00749858986695
 .00493247677957
 .00482067534091
 .0158519911126
 .019188446918
 .00588588588589
 .00641961293113
 .0143722943723
 .0147001809253
 .0075821201941
 .00905139758364
 .00405206603749
 .00394800300587
 .0111256471325
 .010051841215
 .000213248333296
 .000566294170945
 .00963105963106
 .00862895493768

 395
 678
 496
 432
 1484
 1055
 441
 475
 1328
 1365
 650
 908
 471
 352
 995
 826
 19
 60
 898
 828

 .00432924156072
 .00749858986695
 .00493247677957
 .00482067534091
 .0158519911126
 .019188446918
 .00588588588589
 .00641961293113
 .0143722943723
 .0147001809253
 .0075821201941
 .00905139758364
 .00405206603749
 .00394800300587
 .0111256471325
 .010051841215
 .000213248333296
 .000566294170945
 .00963105963106
 .00862895493768

 395
 678
 496
 432
 1484
 1055
 441
 475
 1328
 1365
 650
 908
 471
 352
 995
 826
 19
 60
 898
 828

 .00432924156072
 .00749858986695
 .00493247677957
 .00482067534091
 .0158519911126
 .019188446918
 .00588588588589
 .00641961293113
 .0143722943723
 .0147001809253
 .0075821201941
 .00905139758364
 .00405206603749
 .00394800300587
 .0111256471325
 .010051841215
 .000213248333296
 .000566294170945
 .00963105963106
 .00862895493768

 395
 678
 496
 432
 1484
 1055
 441
 475
 1328
 1365
 650
 908
 471
 352
 995
 826
 19
 60
 898
 828

 .00432924156072
 .00749858986695
 .00493247677957
 .00482067534091
 .0158519911126
 .019188446918
 .00588588588589
 .00641961293113
 .0143722943723
 .0147001809253
 .0075821201941
 .00905139758364
 .00405206603749
 .00394800300587
 .0111256471325
 .010051841215
 .000213248333296
 .000566294170945
 .00963105963106
 .00862895493768

 395
 678
 496
 432
 1484
 1055
 441
 475
 1328
 1365
 650
 908
 471
 352
 995
 826
 19
 60
 898
 828

 .000032880315651
 3.31796011812E-05
 5.96670578174E-05
 4.46358827862E-05
 .000149547085968
 .000181881013441
 6.67334000667E-05
 9.46048221429E-05
 .000183982683983
 9.69242698372E-05
 .000174972004479
 .000179432991746
 .000258093378184
 .000235534270236
 .000167723323605
 .000146031591501
 0
 0
 .00032175032175
 .000354329067489

 3
 3
 6
 4
 14
 10
 5
 7
 17
 9
 15
 18
 30
 21
 15
 12
 12
 12
 30
 34

 .000032880315651
 3.31796011812E-05
 5.96670578174E-05
 4.46358827862E-05
 .000149547085968
 .000181881013441
 6.67334000667E-05
 9.46048221429E-05
 .000183982683983
 9.69242698372E-05
 .000174972004479
 .000179432991746
 .000258093378184
 .000235534270236
 .000167723323605
 .000146031591501
 0
 0
 .00032175032175
 .000354329067489

 3
 3
 6
 4
 14
 10
 5
 7
 17
 9
 15
 18
 30
 21
 15
 12
 12
 12
 30
 34

 .000032880315651
 3.31796011812E-05
 5.96670578174E-05
 4.46358827862E-05
 .000149547085968
 .000181881013441
 6.67334000667E-05
 9.46048221429E-05
 .000183982683983
 9.69242698372E-05
 .000174972004479
 .000179432991746
 .000258093378184
 .000235534270236
 .000167723323605
 .000146031591501
 0
 0
 .00032175032175
 .000354329067489

 3
 3
 6
 4
 14
 10
 5
 7
 17
 9
 15
 18
 30
 21
 15
 12
 12
 12
 30
 34

 .000032880315651
 3.31796011812E-05
 5.96670578174E-05
 4.46358827862E-05
 .000149547085968
 .000181881013441
 6.67334000667E-05
 9.46048221429E-05
 .000183982683983
 9.69242698372E-05
 .000174972004479
 .000179432991746
 .000258093378184
 .000235534270236
 .000167723323605
 .000146031591501
 0
 0
 .00032175032175
 .000354329067489

 3
 3
 6
 4
 14
 10
 5
 7
 17
 9
 15
 18
 30
 21
 15
 12
 12
 12
 30
 34

 0
 0
 .00589709421428
 .00514428549111
 0
 0
 2.66933600267E-05
 1.35149745918E-05
 0
 0
 0
 .000109653494956
 .000129046689092
 .000123375093933
 .00262766540315
 .00295713972789
 .0280028732407
 .0371300211416
 .000793650793651
 .000687815248656

 0
 0
 593
 461
 461
 461
 2
 1
 1
 1
 1
 11
 15
 11
 235
 243
 2495
 3934
 74
 66

 0
 0
 .00589709421428
 .00514428549111
 0
 0
 2.66933600267E-05
 1.35149745918E-05
 0
 0
 0
 .000109653494956
 .000129046689092
 .000123375093933
 .00262766540315
 .00295713972789
 .0280028732407
 .0371300211416
 .000793650793651
 .000687815248656

 0
 0
 593
 461
 461
 461
 2
 1
 1
 1
 1
 11
 15
 11
 235
 243
 2495
 3934
 74
 66

 0
 0
 .00589709421428
 .00514428549111
 0
 0
 2.66933600267E-05
 1.35149745918E-05
 0
 0
 0
 .000109653494956
 .000129046689092
 .000123375093933
 .00262766540315
 .00295713972789
 .0280028732407
 .0371300211416
 .000793650793651
 .000687815248656

 0
 0
 593
 461
 461
 461
 2
 1
 1
 1
 1
 11
 15
 11
 235
 243
 2495
 3934
 74
 66

 0
 0
 .00589709421428
 .00514428549111
 0
 0
 2.66933600267E-05
 1.35149745918E-05
 0
 0
 0
 .000109653494956
 .000129046689092
 .000123375093933
 .00262766540315
 .00295713972789
 .0280028732407
 .0371300211416
 .000793650793651
 .000687815248656

 0
 0
 593
 461
 461
 461
 2
 1
 1
 1
 1
 11
 15
 11
 235
 243
 2495
 3934
 74
 66

 0
 0
 .00589709421428
 .00514428549111
 0
 0
 2.66933600267E-05
 1.35149745918E-05
 0
 0
 0
 .000109653494956
 .000129046689092
 .000123375093933
 .00262766540315
 .00295713972789
 .0280028732407
 .0371300211416
 .000793650793651
 .000687815248656

 0
 0
 593
 461
 461
 461
 2
 1
 1
 1
 1
 11
 15
 11
 235
 243
 2495
 3934
 74
 66

 0
 0
 0
 0
 0
 0
 0
 1.35149745918E-05
 7.57575757576E-05
 .000140001723098
 0
 0
 0
 0
 0
 0
 0
 9.43823618242E-06

 0
 0
 0
 0
 0
 0
 0
 1
 7
 13
 13
 13
 13
 13
 13
 13
 13
 1

 0
 0
 0
 0
 0
 0
 0
 1.35149745918E-05
 7.57575757576E-05
 .000140001723098
 0
 0
 0
 0
 0
 0
 0
 9.43823618242E-06

 0
 0
 0
 0
 0
 0
 0
 1
 7
 13
 13
 13
 13
 13
 13
 13
 13
 1

 0
 0
 0
 0
 0
 0
 0
 1.35149745918E-05
 7.57575757576E-05
 .000140001723098
 0
 0
 0
 0
 0
 0
 0
 9.43823618242E-06

 0
 0
 0
 0
 0
 0
 0
 1
 7
 13
 13
 13
 13
 13
 13
 13
 13
 1

 0
 0
 0
 0
 0
 0
 0
 1.35149745918E-05
 7.57575757576E-05
 .000140001723098
 0
 0
 0
 0
 0
 0
 0
 9.43823618242E-06

 0
 0
 0
 0
 0
 0
 0
 1
 7
 13
 13
 13
 13
 13
 13
 13
 13
 1

 0
 0
 0
 0
 0
 0
 0
 1.35149745918E-05
 7.57575757576E-05
 .000140001723098
 0
 0
 0
 0
 0
 0
 0
 9.43823618242E-06

 0
 0
 0
 0
 0
 0
 0
 1
 7
 13
 13
 13
 13
 13
 13
 13
 13
 1

 .155677334502429
 .13907782828452
 9.44728415441913E-02
 9.32555181110672E-02
 5.54071953511802E-02
 5.08903075607898E-02
 3.74240907574354E-02
 4.05043788517913E-02
 5.40151515151552E-02
 7.28116653743168E-02
 7.45147443075779E-02
 6.75365843933044E-02
 .084026600824196
 6.74861763815405E-02
 8.33137656122611E-02
 6.50449047143323E-02
 6.68926350759783E-02
 7.30802627604746E-02
 .119305019305087
 .123869273417006

 1052.24922557075
 923.294075546805
 781.409894737237
 773.740217781938
 440.320223635726
 233.112223016407
 279.039229671941
 322.067734401379
 589.780605089372
 613.495193018611
 459.651847213843
 528.810774907172
 706.047199754141
 465.456373608279
 735.416588377537
 561.142937324171
 531.488255033455
 1440.63528348184
 776.605717368328
 845.861181221768

 3.56751424813522E-02
 4.51574372076228E-02
 .016507885996164
 .015711830740761
 .018084515467442
 1.64966079191005E-02
 9.0090090090147E-03
 .010798464698883
 2.18073593073686E-02
 2.47695356250314E-02
 .01663400522582
 .01360700187408
 1.26551786436361E-02
 .011114974371631
 .014871468026377
 .012802102854907
 4.8149228939003E-03
 4.4359710057308E-03
 .021814671814706
 .02391721205551

 1123.18801843281
 1451.29635072246
 797.680722892076
 679.005681818727
 541.85056113402
 291.597574421147
 236.407407407428
 278.924906132593
 1090.95136476459
 1058.34260869525
 501.10098176723
 467.621245421253
 658.200543847788
 506.8526740667
 860.461654135598
 738.832699619617
 202.034965034978
 234.165957446798
 1241.27335299942
 1365.23267973789

 3.56751424813522E-02
 4.51574372076228E-02
 .016507885996164
 .015711830740761
 .018084515467442
 1.64966079191005E-02
 9.0090090090147E-03
 .010798464698883
 2.18073593073686E-02
 2.47695356250314E-02
 .01663400522582
 .01360700187408
 1.26551786436361E-02
 .011114974371631
 .014871468026377
 .012802102854907
 4.8149228939003E-03
 4.4359710057308E-03
 .021814671814706
 .02391721205551

 1123.18801843281
 1451.29635072246
 797.680722892076
 679.005681818727
 541.85056113402
 291.597574421147
 236.407407407428
 278.924906132593
 1090.95136476459
 1058.34260869525
 501.10098176723
 467.621245421253
 658.200543847788
 506.8526740667
 860.461654135598
 738.832699619617
 202.034965034978
 234.165957446798
 1241.27335299942
 1365.23267973789

 .0163853572994
 .0220644347855
 .0106704588397
 .0101100274511
 .00778713040506
 .0069296666121
 .00433767100434
 .00521678019245
 .0153787878788
 .0155617299905
 .00858529301978
 .00680848518681
 .00800949783632
 .00767168765913
 .0117294510975
 .0105994596831
 .00233450806977
 .00278427967381
 .0166666666667
 .0179978323398

 1495
 1995
 1073
 906
 729
 381
 325
 386
 1421
 1445
 736
 683
 931
 684
 1049
 871
 208
 295
 1554
 1727

 .0163853572994
 .0220644347855
 .0106704588397
 .0101100274511
 .00778713040506
 .0069296666121
 .00433767100434
 .00521678019245
 .0153787878788
 .0155617299905
 .00858529301978
 .00680848518681
 .00800949783632
 .00767168765913
 .0117294510975
 .0105994596831
 .00233450806977
 .00278427967381
 .0166666666667
 .0179978323398

 1495
 1995
 1073
 906
 729
 381
 325
 386
 1421
 1445
 736
 683
 931
 684
 1049
 871
 208
 295
 1554
 1727

 .0163853572994
 .0220644347855
 .0106704588397
 .0101100274511
 .00778713040506
 .0069296666121
 .00433767100434
 .00521678019245
 .0153787878788
 .0155617299905
 .00858529301978
 .00680848518681
 .00800949783632
 .00767168765913
 .0117294510975
 .0105994596831
 .00233450806977
 .00278427967381
 .0166666666667
 .0179978323398

 1495
 1995
 1073
 906
 729
 381
 325
 386
 1421
 1445
 736
 683
 931
 684
 1049
 871
 208
 295
 1554
 1727

 6.57606313021E-05
 3.31796011812E-05
 0
 0
 0
 0
 0
 0
 0
 1.07693633152E-05

 6
 3
 3
 3
 3
 3
 3
 3
 3
 1

 6.57606313021E-05
 3.31796011812E-05
 0
 0
 0
 0
 0
 0
 0
 1.07693633152E-05

 6
 3
 3
 3
 3
 3
 3
 3
 3
 1

 6.57606313021E-05
 3.31796011812E-05
 0
 0
 0
 0
 0
 0
 0
 1.07693633152E-05

 6
 3
 3
 3
 3
 3
 3
 3
 3
 1

 .00503068829461
 .00891425285068
 .000885061357624
 .000892717655723
 .00590710989574
 .00562012331533
 .00281614948282
 .00324359390204
 .00435064935065
 .00590161109675
 .00351110488988
 .00319988835281
 .00190128788596
 .00162630805639
 .00082743506312
 .000425925475211
 .000134683157871
 .000217079432196
 .00136207636208
 .00162574513319

 459
 806
 89
 80
 553
 309
 211
 240
 402
 548
 301
 321
 221
 145
 74
 35
 12
 23
 127
 156

 .00503068829461
 .00891425285068
 .000885061357624
 .000892717655723
 .00590710989574
 .00562012331533
 .00281614948282
 .00324359390204
 .00435064935065
 .00590161109675
 .00351110488988
 .00319988835281
 .00190128788596
 .00162630805639
 .00082743506312
 .000425925475211
 .000134683157871
 .000217079432196
 .00136207636208
 .00162574513319

 459
 806
 89
 80
 553
 309
 211
 240
 402
 548
 301
 321
 221
 145
 74
 35
 12
 23
 127
 156

 .00503068829461
 .00891425285068
 .000885061357624
 .000892717655723
 .00590710989574
 .00562012331533
 .00281614948282
 .00324359390204
 .00435064935065
 .00590161109675
 .00351110488988
 .00319988835281
 .00190128788596
 .00162630805639
 .00082743506312
 .000425925475211
 .000134683157871
 .000217079432196
 .00136207636208
 .00162574513319

 459
 806
 89
 80
 553
 309
 211
 240
 402
 548
 301
 321
 221
 145
 74
 35
 12
 23
 127
 156

 .00223586146427
 .00161474059082
 .0038982477774
 .00392795768518
 .00212570500769
 .00240082937742
 .000854187520854
 .00151367715429
 .000800865800866
 .00144309468424
 .00293952967525
 .00222297539774
 .00164319450777
 .00104308033962
 .00219158476178
 .00159417820722
 .00233450806977
 .00141573542736
 .00323895323895
 .00364750510651

 204
 146
 392
 352
 199
 132
 64
 112
 74
 134
 252
 223
 191
 93
 196
 131
 208
 150
 302
 350

 .00223586146427
 .00161474059082
 .0038982477774
 .00392795768518
 .00212570500769
 .00240082937742
 .000854187520854
 .00151367715429
 .000800865800866
 .00144309468424
 .00293952967525
 .00222297539774
 .00164319450777
 .00104308033962
 .00219158476178
 .00159417820722
 .00233450806977
 .00141573542736
 .00323895323895
 .00364750510651

 204
 146
 392
 352
 199
 132
 64
 112
 74
 134
 252
 223
 191
 93
 196
 131
 208
 150
 302
 350

 .00223586146427
 .00161474059082
 .0038982477774
 .00392795768518
 .00212570500769
 .00240082937742
 .000854187520854
 .00151367715429
 .000800865800866
 .00144309468424
 .00293952967525
 .00222297539774
 .00164319450777
 .00104308033962
 .00219158476178
 .00159417820722
 .00233450806977
 .00141573542736
 .00323895323895
 .00364750510651

 204
 146
 392
 352
 199
 132
 64
 112
 74
 134
 252
 223
 191
 93
 196
 131
 208
 150
 302
 350

 1.19245944761191E-02
 1.25308293794416E-02
 .00105411802144
 .000781127948758
 .002157750811832
 1.4914243102182E-03
 .000974307640974
 .000824413450103
 1.1904761904725E-03
 1.8307917635957E-03
 .00159807764091
 .00137565293672
 .00109259530098
 .000773898316491
 .000122997103977
 .000182539489376
 1.12235964893E-05
 1.88764723648E-05
 .000546975546976
 .00064612947601

 1074.09007352942
 1125.02824360106
 106
 70
 200.009900990103
 78.0975609756123
 73
 61
 108.01818181818
 164.105882352951
 137
 138
 127
 69
 11
 15
 1
 2
 49.0392156862763
 62

 7.67207365191E-05
 4.42394682416E-05
 0
 0
 .000010681934712
 3.63762026882E-05
 0
 0
 1.08225108225E-05
 3.23080899457E-05
 0
 0
 0
 0
 0
 0
 0
 0
 .000010725010725

 7
 4
 4
 4
 1
 2
 2
 2
 1
 3
 3
 3
 3
 3
 3
 3
 3
 3
 1

 7.67207365191E-05
 4.42394682416E-05
 0
 0
 .000010681934712
 3.63762026882E-05
 0
 0
 1.08225108225E-05
 3.23080899457E-05
 0
 0
 0
 0
 0
 0
 0
 0
 .000010725010725

 7
 4
 4
 4
 1
 2
 2
 2
 1
 3
 3
 3
 3
 3
 3
 3
 3
 3
 1

 .0118478737396
 .0124865899112
 .00105411802144
 .000781127948758
 .00214706887712
 .00145504810753
 .000974307640974
 .000824413450103
 .00117965367965
 .00179848367365
 .00159807764091
 .00137565293672
 .00109259530098
 .000773898316491
 .000122997103977
 .000182539489376
 1.12235964893E-05
 1.88764723648E-05
 .000536250536251
 .00064612947601

 1081
 1129
 106
 70
 201
 80
 73
 61
 109
 167
 137
 138
 127
 69
 11
 15
 1
 2
 50
 62

 .0118478737396
 .0124865899112
 .00105411802144
 .000781127948758
 .00214706887712
 .00145504810753
 .000974307640974
 .000824413450103
 .00117965367965
 .00179848367365
 .00159807764091
 .00137565293672
 .00109259530098
 .000773898316491
 .000122997103977
 .000182539489376
 1.12235964893E-05
 1.88764723648E-05
 .000536250536251
 .00064612947601

 1081
 1129
 106
 70
 201
 80
 73
 61
 109
 167
 137
 138
 127
 69
 11
 15
 1
 2
 50
 62

 .000032880315651
 0
 0
 0
 .00010681934712
 5.45643040323E-05
 2.66933600267E-05
 0
 8.65800865801E-05
 2.15387266305E-05
 0
 0
 8.60311260614E-06

 3
 3
 3
 3
 10
 3
 2
 2
 8
 2
 2
 2
 1

 .000032880315651
 0
 0
 0
 .00010681934712
 5.45643040323E-05
 2.66933600267E-05
 0
 8.65800865801E-05
 2.15387266305E-05
 0
 0
 8.60311260614E-06

 3
 3
 3
 3
 10
 3
 2
 2
 8
 2
 2
 2
 1

 .000032880315651
 0
 0
 0
 .00010681934712
 5.45643040323E-05
 2.66933600267E-05
 0
 8.65800865801E-05
 2.15387266305E-05
 0
 0
 8.60311260614E-06

 3
 3
 3
 3
 10
 3
 2
 2
 8
 2
 2
 2
 1

 9.24375274002957E-02
 7.12808432042548E-02
 4.36464527933954E-02
 4.15894837860232E-02
 1.82554264228292E-02
 1.77515869118407E-02
 1.16916916916956E-02
 .010825494648072
 1.72186147186131E-02
 2.60403204962442E-02
 3.03634751772992E-02
 2.79417042146876E-02
 .03818921685868
 2.91277380858911E-02
 3.43273735645501E-02
 2.71740453184614E-02
 3.68246200812617E-02
 3.32886590153937E-02
 6.04783354783346E-02
 6.17157864021008E-02

 1162.34526914964
 737.959193173088
 276.574846206481
 264.098470619754
 134.730836746706
 85.059426229508
 72.2328767123845
 58.9725343321129
 127.343180389659
 209.957816377122
 151.747598924306
 161.781305743831
 356.494931290874
 234.325375433153
 237.17263843644
 189.927899686468
 238.024687595266
 254.002551743648
 308.714843057299
 318.455589327934

 9.23388864533426E-02
 .071203424134832
 4.36464527933954E-02
 4.15894837860232E-02
 1.82340625534052E-02
 1.76970226078084E-02
 1.16916916916956E-02
 1.08119796734802E-02
 1.71212121212105E-02
 2.59649349530375E-02
 .030340145576702
 2.79417042146876E-02
 .03818921685868
 2.91277380858911E-02
 3.43273735645501E-02
 2.71740453184614E-02
 3.67909492917939E-02
 3.32697825430289E-02
 6.04783354783346E-02
 6.17053649589394E-02

 1163.57922848761
 738.757067412326
 276.574846206481
 264.098470619754
 134.886350322273
 85.312435765673
 72.2328767123845
 59.0450000000278
 128.016434892508
 210.547075902066
 151.862745098027
 161.781305743831
 356.494931290874
 234.325375433153
 237.17263843644
 189.927899686468
 238.239780353895
 254.14553191485
 308.714843057299
 318.509204526267

 .000569925471284
 .000597232821262
 .005658425983018
 .00696319771464
 3.845496496321E-04
 3.819501282262E-04
 4.004004004001E-04
 3.108444156125E-04
 .000346320346321
 .00043077453261
 .000501586412841
 .00054826747478
 .001307673116137
 .001615092138768
 .00824080596647
 .00652274442037
 .00417517789401
 .00514383871942
 .00629558129558
 .0061903372379

 30.6538461538462
 32.3333333333378
 431.49560632695
 395.080128205004
 24.7222222222176
 17.3809523809528
 20.3999999999964
 17.7826086956546
 16.5624999999984
 20
 29.9767441860478
 39.9454545454607
 111.578947368496
 102.680555555575
 439.279511533325
 290.701492537305
 186.435483870974
 279.444036697326
 295.381601362879
 297.215488215484

 .000569925471284
 .000597232821262
 .005658425983018
 .00696319771464
 3.845496496321E-04
 3.819501282262E-04
 4.004004004001E-04
 3.108444156125E-04
 .000346320346321
 .00043077453261
 .000501586412841
 .00054826747478
 .001307673116137
 .001615092138768
 .00824080596647
 .00652274442037
 .00417517789401
 .00514383871942
 .00629558129558
 .0061903372379

 30.6538461538462
 32.3333333333378
 431.49560632695
 395.080128205004
 24.7222222222176
 17.3809523809528
 20.3999999999964
 17.7826086956546
 16.5624999999984
 20
 29.9767441860478
 39.9454545454607
 111.578947368496
 102.680555555575
 439.279511533325
 290.701492537305
 186.435483870974
 279.444036697326
 295.381601362879
 297.215488215484

 .000569925471284
 .000597232821262
 .005658425983018
 .00696319771464
 3.845496496321E-04
 3.819501282262E-04
 4.004004004001E-04
 3.108444156125E-04
 .000346320346321
 .00043077453261
 .000501586412841
 .00054826747478
 .001307673116137
 .001615092138768
 .00824080596647
 .00652274442037
 .00417517789401
 .00514383871942
 .00629558129558
 .0061903372379

 30.6538461538462
 32.3333333333378
 431.49560632695
 395.080128205004
 24.7222222222176
 17.3809523809528
 20.3999999999964
 17.7826086956546
 16.5624999999984
 20
 29.9767441860478
 39.9454545454607
 111.578947368496
 102.680555555575
 439.279511533325
 290.701492537305
 186.435483870974
 279.444036697326
 295.381601362879
 297.215488215484

 2.4002630425221E-03
 1.4488425849108E-03
 .001103840569625
 1.0824201575692E-03
 4.27277388481E-05
 9.09405067205E-05
 0
 .000108119796735
 9.74025974026E-05
 1.184629964676E-04
 6.99888017917E-05
 7.97479963316E-05
 8.60311260614E-06
 6.72955057818E-05
 7.938903983991E-04
 5.841263660039E-04
 .000190801140317
 6.606765327694E-05
 1.072501072501E-04
 1.771645337441E-04

 207.328767123277
 127.061068702288
 103.216216216221
 85.6391752577592
 4
 5
 5
 8
 4.55555555555601
 6.63636363636402
 4.33333333333429
 8
 1
 3.33333333333333
 67.1126760563346
 40.5416666666733
 17
 3.85714285714286
 3.80000000000112
 8.05882352940828

 6.57606313021E-05
 2.21197341208E-05
 1.98890192725E-05
 5.57948534827E-05
 0
 0
 0
 0
 .00004329004329
 3.23080899457E-05
 1.16648002986E-05
 0
 0
 0
 2.23631098141E-05
 3.65078978752E-05
 0
 9.43823618242E-06
 .00002145002145
 1.04214431614E-05

 6
 2
 2
 5
 5
 5
 5
 5
 4
 3
 1
 1
 1
 1
 2
 3
 3
 1
 2
 1

 6.57606313021E-05
 2.21197341208E-05
 1.98890192725E-05
 5.57948534827E-05
 0
 0
 0
 0
 .00004329004329
 3.23080899457E-05
 1.16648002986E-05
 0
 0
 0
 2.23631098141E-05
 3.65078978752E-05
 0
 9.43823618242E-06
 .00002145002145
 1.04214431614E-05

 6
 2
 2
 5
 5
 5
 5
 5
 4
 3
 1
 1
 1
 1
 2
 3
 3
 1
 2
 1

 .00233450241122
 .00142672285079
 1.98890192725E-05
 1.11589706965E-05
 4.27277388481E-05
 9.09405067205E-05
 0
 .000108119796735
 5.41125541126E-05
 8.61549065219E-05
 5.83240014931E-05
 7.97479963316E-05
 0
 4.48636705212E-05
 0
 1.21692992917E-05
 0
 9.43823618242E-06
 5.36250536251E-05
 6.25286589687E-05

 213
 129
 2
 1
 4
 5
 5
 8
 5
 8
 5
 8
 8
 4
 4
 1
 1
 1
 5
 6

 .00233450241122
 .00142672285079
 1.98890192725E-05
 1.11589706965E-05
 4.27277388481E-05
 9.09405067205E-05
 0
 .000108119796735
 5.41125541126E-05
 8.61549065219E-05
 5.83240014931E-05
 7.97479963316E-05
 0
 4.48636705212E-05
 0
 1.21692992917E-05
 0
 9.43823618242E-06
 5.36250536251E-05
 6.25286589687E-05

 213
 129
 2
 1
 4
 5
 5
 8
 5
 8
 5
 8
 8
 4
 4
 1
 1
 1
 5
 6

 0
 0
 .00106406253108
 .00101546633339
 0
 0
 0
 0
 0
 0
 0
 0
 8.60311260614E-06
 2.24318352606E-05
 .000771527288585
 .000535449168837
 .000190801140317
 4.71911809121E-05
 .000032175032175
 .000104214431614

 0
 0
 107
 91
 91
 91
 91
 91
 91
 91
 91
 91
 1
 2
 69
 44
 17
 5
 3
 10

 0
 0
 .00106406253108
 .00101546633339
 0
 0
 0
 0
 0
 0
 0
 0
 8.60311260614E-06
 2.24318352606E-05
 .000771527288585
 .000535449168837
 .000190801140317
 4.71911809121E-05
 .000032175032175
 .000104214431614

 0
 0
 107
 91
 91
 91
 91
 91
 91
 91
 91
 91
 1
 2
 69
 44
 17
 5
 3
 10

 1.205611573871E-04
 2.543769423888E-04
 7.95560770898E-05
 2.23179413931E-05
 1.281832165441E-04
 1.273167094087E-04
 1.868535201867E-04
 1.621796951025E-04
 .00004329004329
 1.184629964672E-04
 2.332960059729E-04
 2.2927548945335E-04
 6.88249008491E-05
 7.85114234121E-05
 6.70893294422E-05
 2.43385985834E-05
 1.12235964893E-05
 2.83147085473E-05
 .0002145002145
 .000093792988453

 7.54545454545575
 13.2608695652131
 4.24999999999937
 2
 4.50000000000117
 3.57142857142857
 7
 7.16666666667252
 2.5
 9.18181818181818
 14.9000000000021
 17.6086956521739
 4.99999999999855
 7
 3
 1
 1
 3
 7.7
 5.00000000000107

 .000010960105217
 0
 0
 0
 .000032045804136
 0
 0
 1.35149745918E-05
 0
 0
 0
 9.96849954145E-06
 0
 0
 0
 1.21692992917E-05
 0
 0
 .0000429000429

 1
 1
 1
 1
 3
 3
 3
 1
 1
 1
 1
 1
 1
 1
 1
 1
 1
 1
 4

 .000010960105217
 0
 0
 0
 .000032045804136
 0
 0
 1.35149745918E-05
 0
 0
 0
 9.96849954145E-06
 0
 0
 0
 1.21692992917E-05
 0
 0
 .0000429000429

 1
 1
 1
 1
 3
 3
 3
 1
 1
 1
 1
 1
 1
 1
 1
 1
 1
 1
 4

 9.86409469531E-05
 .000176957872966
 4.97225481811E-05
 2.23179413931E-05
 6.40916082721E-05
 7.27524053764E-05
 5.33867200534E-05
 2.70299491837E-05
 3.24675324675E-05
 .000107693633152
 .000198301605077
 .000199369990829
 5.16186756368E-05
 0
 3.35446647211E-05
 1.21692992917E-05
 1.12235964893E-05
 2.83147085473E-05
 .0000429000429
 6.25286589687E-05

 9
 16
 5
 2
 6
 4
 4
 2
 3
 10
 17
 20
 6
 6
 3
 1
 1
 3
 4
 6

 9.86409469531E-05
 .000176957872966
 4.97225481811E-05
 2.23179413931E-05
 6.40916082721E-05
 7.27524053764E-05
 5.33867200534E-05
 2.70299491837E-05
 3.24675324675E-05
 .000107693633152
 .000198301605077
 .000199369990829
 5.16186756368E-05
 0
 3.35446647211E-05
 1.21692992917E-05
 1.12235964893E-05
 2.83147085473E-05
 .0000429000429
 6.25286589687E-05

 9
 16
 5
 2
 6
 4
 4
 2
 3
 10
 17
 20
 6
 6
 3
 1
 1
 3
 4
 6

 .000010960105217
 7.74190694228E-05
 2.98335289087E-05
 0
 .000032045804136
 5.45643040323E-05
 1.33466800133E-05
 0
 1.08225108225E-05
 1.07693633152E-05
 3.49944008959E-05
 1.99369990829E-05
 1.72062252123E-05
 7.85114234121E-05
 3.35446647211E-05
 0
 0
 0
 .000117975117975
 3.12643294843E-05

 1
 7
 3
 3
 3
 3
 1
 1
 1
 1
 3
 2
 2
 7
 3
 3
 3
 3
 11
 3

 .000010960105217
 7.74190694228E-05
 2.98335289087E-05
 0
 .000032045804136
 5.45643040323E-05
 1.33466800133E-05
 0
 1.08225108225E-05
 1.07693633152E-05
 3.49944008959E-05
 1.99369990829E-05
 1.72062252123E-05
 7.85114234121E-05
 3.35446647211E-05
 0
 0
 0
 .000117975117975
 3.12643294843E-05

 1
 7
 3
 3
 3
 3
 1
 1
 1
 1
 3
 2
 2
 7
 3
 3
 3
 3
 11
 3

 0
 0
 0
 0
 0
 0
 .00012012012012
 .000121634771327
 0
 0
 0
 0
 0
 0
 0
 0
 0
 0
 .000010725010725

 0
 0
 0
 0
 0
 0
 9
 9
 9
 9
 9
 9
 9
 9
 9
 9
 9
 9
 1

 0
 0
 0
 0
 0
 0
 .00012012012012
 .000121634771327
 0
 0
 0
 0
 0
 0
 0
 0
 0
 0
 .000010725010725

 0
 0
 0
 0
 0
 0
 9
 9
 9
 9
 9
 9
 9
 9
 9
 9
 9
 9
 1

 .000723366944323
 4.645144165364E-04
 4.1170269894031E-03
 3.9279576851865E-03
 .000117501281832
 5.45643040323E-05
 2.002002002003E-04
 2.027246188775E-04
 .000021645021645
 5.38468165762E-05
 7.348824188132E-04
 8.4732246102345E-04
 6.7964589588514E-04
 .000672955057818
 .004193083090131
 .005098936403238
 2.0090237715738E-03
 .00168944427665
 .00438652938653
 4.3353203551634E-03

 64.0303030303044
 40.0476190476182
 370.120772946897
 301.99431818185
 11
 3
 13.1333333333368
 9.9333333333335
 1
 5
 59.1269841269877
 83.0235294117649
 45.8354430379799
 43.3333333333333
 319.645333333294
 358.198090692078
 139.23463687147
 134.240223463692
 360.070904645503
 360.18750000001

 .000712406839106
 4.645144165364E-04
 .004067304441222
 .00391679871449
 .000117501281832
 5.45643040323E-05
 2.002002002003E-04
 1.892096442857E-04
 .000021645021645
 5.38468165762E-05
 7.348824188132E-04
 8.4732246102345E-04
 .000671042783279
 .000672955057818
 .004181901535224
 .005098936403238
 .001930458596149
 .001576185442461
 .00436507936508
 .004324898912002

 65
 40.0476190476182
 374.584352078268
 302.85185185188
 11
 3
 13.1333333333368
 10.5714285714271
 1
 5
 59.1269841269877
 83.0235294117649
 46.4102564102616
 43.3333333333333
 320.497326203168
 358.198090692078
 144.616279069736
 143.023952095828
 361.830466830491
 361.053012048198

 .000712406839106
 4.645144165364E-04
 .004067304441222
 .00391679871449
 .000117501281832
 5.45643040323E-05
 2.002002002003E-04
 1.892096442857E-04
 .000021645021645
 5.38468165762E-05
 7.348824188132E-04
 8.4732246102345E-04
 .000671042783279
 .000672955057818
 .004181901535224
 .005098936403238
 .001930458596149
 .001576185442461
 .00436507936508
 .004324898912002

 65
 40.0476190476182
 374.584352078268
 302.85185185188
 11
 3
 13.1333333333368
 10.5714285714271
 1
 5
 59.1269841269877
 83.0235294117649
 46.4102564102616
 43.3333333333333
 320.497326203168
 358.198090692078
 144.616279069736
 143.023952095828
 361.830466830491
 361.053012048198

 .000010960105217
 0
 4.97225481811E-05
 1.11589706965E-05
 0
 0
 0
 1.35149745918E-05
 0
 0
 0
 0
 8.60311260614E-06
 0
 .000011181554907
 0
 7.85651754248E-05
 .000113258834189
 .00002145002145
 1.04214431614E-05

 1
 1
 5
 1
 1
 1
 1
 1
 1
 1
 1
 1
 1
 1
 1
 1
 7
 12
 2
 1

 .000010960105217
 0
 4.97225481811E-05
 1.11589706965E-05
 0
 0
 0
 1.35149745918E-05
 0
 0
 0
 0
 8.60311260614E-06
 0
 .000011181554907
 0
 7.85651754248E-05
 .000113258834189
 .00002145002145
 1.04214431614E-05

 1
 1
 5
 1
 1
 1
 1
 1
 1
 1
 1
 1
 1
 1
 1
 1
 7
 12
 2
 1

 9.973695747472E-04
 .001249764977825
 1.1635076274384E-03
 .000948512509206
 6.302341480081E-04
 7.457121551087E-04
 .000173506840173
 .000202724618878
 1.2445887445882E-03
 .002380029292671
 .001283128032848
 .001186251445433
 2.7529960339678E-04
 2.916138583883E-04
 2.683573177681E-04
 2.920631830024E-04
 .003468091315178
 .002803156146181
 .001791076791077
 .001771645337446

 34.7582417582361
 42.9115044247683
 42.6068376068334
 26.9058823529394
 24.9999999999967
 14.9024390243909
 8.07692307691958
 6.33333333334007
 68.3913043478237
 99.4796380090616
 36.2727272727229
 36.7478991596652
 15.1250000000054
 12.0769230769233
 10.3333333333302
 7.50000000000411
 108.792880258894
 106.037037037047
 47.1197604790496
 46.3764705882316

 .000854888206926
 .001061747237798
 .00097456194435
 .000703015153882
 .000555460605024
 .0006183954457
 .000133466800133
 1.621796951025E-04
 .001093073593073
 .002035409666583
 .000816536020903
 .000697794967902
 2.580933781845E-04
 .000280397940758
 .000245994207954
 .000219047387252
 .00283956991178
 .00229349139233
 .001158301158302
 .001146358747759

 39.4102564102564
 48.7499999999958
 49.0204081632648
 32.460317460318
 27.8846153846154
 17.2352941176455
 10
 7.50000000000462
 76.8811881188165
 113.613756613765
 43.2571428571452
 38.457142857142
 16.0666666666713
 12.5199999999994
 11.0909090909091
 9
 126.549407114619
 121.748971193424
 54.2962962962958
 55.0181818181819

 .000854888206926
 .001061747237798
 .00097456194435
 .000703015153882
 .000555460605024
 .0006183954457
 .000133466800133
 1.621796951025E-04
 .001093073593073
 .002035409666583
 .000816536020903
 .000697794967902
 2.580933781845E-04
 .000280397940758
 .000245994207954
 .000219047387252
 .00283956991178
 .00229349139233
 .001158301158302
 .001146358747759

 39.4102564102564
 48.7499999999958
 49.0204081632648
 32.460317460318
 27.8846153846154
 17.2352941176455
 10
 7.50000000000462
 76.8811881188165
 113.613756613765
 43.2571428571452
 38.457142857142
 16.0666666666713
 12.5199999999994
 11.0909090909091
 9
 126.549407114619
 121.748971193424
 54.2962962962958
 55.0181818181819

 5.48005260851E-05
 .000055299335302
 9.94450963623E-05
 .000122748677662
 .000032045804136
 7.27524053764E-05
 1.33466800133E-05
 2.70299491837E-05
 6.49350649351E-05
 .000172309813044
 .000128312803285
 .000089716495873
 8.60311260614E-06
 0
 0
 3.65078978752E-05
 .000269366315742
 .000113258834189
 .00019305019305
 .000270957522198

 5
 5
 10
 11
 3
 4
 1
 2
 6
 16
 11
 9
 1
 1
 1
 3
 24
 12
 18
 26

 5.48005260851E-05
 .000055299335302
 9.94450963623E-05
 .000122748677662
 .000032045804136
 7.27524053764E-05
 1.33466800133E-05
 2.70299491837E-05
 6.49350649351E-05
 .000172309813044
 .000128312803285
 .000089716495873
 8.60311260614E-06
 0
 0
 3.65078978752E-05
 .000269366315742
 .000113258834189
 .00019305019305
 .000270957522198

 5
 5
 10
 11
 3
 4
 1
 2
 6
 16
 11
 9
 1
 1
 1
 3
 24
 12
 18
 26

 8.76808417361E-05
 .000132718404725
 8.95005867261E-05
 .000122748677662
 4.27277388481E-05
 5.45643040323E-05
 2.66933600267E-05
 1.35149745918E-05
 8.65800865801E-05
 .000172309813044
 .00033827920866
 .000398739981658
 8.60311260614E-06
 1.12159176303E-05
 2.23631098141E-05
 3.65078978752E-05
 .000359155087656
 .000396405919662
 .000439725439725
 .000354329067489

 8
 12
 9
 11
 4
 3
 2
 1
 8
 16
 29
 40
 1
 1
 2
 3
 32
 42
 41
 34

 8.76808417361E-05
 .000132718404725
 8.95005867261E-05
 .000122748677662
 4.27277388481E-05
 5.45643040323E-05
 2.66933600267E-05
 1.35149745918E-05
 8.65800865801E-05
 .000172309813044
 .00033827920866
 .000398739981658
 8.60311260614E-06
 1.12159176303E-05
 2.23631098141E-05
 3.65078978752E-05
 .000359155087656
 .000396405919662
 .000439725439725
 .000354329067489

 8
 12
 9
 11
 4
 3
 2
 1
 8
 16
 29
 40
 1
 1
 2
 3
 32
 42
 41
 34

 .000131521262604
 7.74190694228E-05
 9.94450963623E-06
 0
 .000010681934712
 3.63762026882E-05
 2.66933600267E-05
 1.35149745918E-05
 .000021645021645
 3.23080899457E-05
 6.99888017917E-05
 4.98424977072E-05
 4.30155630307E-05
 .000100943258673
 0
 0
 0
 0
 9.65250965251E-05
 .000198007420068

 12
 7
 1
 1
 1
 2
 2
 1
 2
 3
 6
 5
 5
 9
 9
 9
 9
 9
 9
 19

 .000131521262604
 7.74190694228E-05
 9.94450963623E-06
 0
 .000010681934712
 3.63762026882E-05
 2.66933600267E-05
 1.35149745918E-05
 .000021645021645
 3.23080899457E-05
 6.99888017917E-05
 4.98424977072E-05
 4.30155630307E-05
 .000100943258673
 0
 0
 0
 0
 9.65250965251E-05
 .000198007420068

 12
 7
 1
 1
 1
 2
 2
 1
 2
 3
 6
 5
 5
 9
 9
 9
 9
 9
 9
 19

 .000131521262604
 7.74190694228E-05
 9.94450963623E-06
 0
 .000010681934712
 3.63762026882E-05
 2.66933600267E-05
 1.35149745918E-05
 .000021645021645
 3.23080899457E-05
 6.99888017917E-05
 4.98424977072E-05
 4.30155630307E-05
 .000100943258673
 0
 0
 0
 0
 9.65250965251E-05
 .000198007420068

 12
 7
 1
 1
 1
 2
 2
 1
 2
 3
 6
 5
 5
 9
 9
 9
 9
 9
 9
 19

 5.48005260851E-05
 4.42394682416E-05
 0
 0
 .000149547085968
 0
 0
 0
 .000021645021645
 3.23080899457E-05
 0
 9.96849954145E-06
 0
 0
 0
 0
 7.85651754248E-05

 5
 4
 4
 4
 14
 14
 14
 14
 2
 3
 3
 1
 1
 1
 1
 1
 7

 5.48005260851E-05
 4.42394682416E-05
 0
 0
 .000149547085968
 0
 0
 0
 .000021645021645
 3.23080899457E-05
 0
 9.96849954145E-06
 0
 0
 0
 0
 7.85651754248E-05

 5
 4
 4
 4
 14
 14
 14
 14
 2
 3
 3
 1
 1
 1
 1
 1
 7

 5.48005260851E-05
 4.42394682416E-05
 0
 0
 .000149547085968
 0
 0
 0
 .000021645021645
 3.23080899457E-05
 0
 9.96849954145E-06
 0
 0
 0
 0
 7.85651754248E-05

 5
 4
 4
 4
 14
 14
 14
 14
 2
 3
 3
 1
 1
 1
 1
 1
 7

 .001380973257343
 1.4156629837312E-03
 .001680622128524
 .001350235454281
 .001068193471201
 1.2004146887111E-03
 6.006006006004E-04
 .000432479186939
 5.627705627706E-04
 1.2384767812527E-03
 .001061496827175
 .001116471948642
 8.775174858265E-04
 7.290346459692E-04
 9.839768318181E-04
 7.544965560887E-04
 .002154930525938
 .002463379643613
 .002681252681255
 .002449039142939

 56.7142857143011
 51.2343749999876
 55.4378698224712
 37.8099173553833
 55.2800000000157
 35.9393939393922
 19.3111111111135
 11.4375000000023
 22.8076923076964
 55.6782608695618
 34.978021978025
 40.9107142857066
 40.5098039215505
 22.138461538462
 31.5227272727266
 24.9999999999972
 58.4895833333368
 83.3448275862165
 86.4400000000794
 69.0765957446523

 .000493204734765
 .000597232821262
 .000626504107083
 .00054678956413
 .000277730302512
 .000327385824194
 2.535869202534E-04
 2.297545680612E-04
 2.056277056276E-04
 .000398466442664
 .000349944008959
 .000388771482117
 .000275299603397
 3.140456936481E-04
 .000424899086467
 2.312166865435E-04
 .000931558508608
 .001094835397161
 .000847275847275
 .000823294009755

 23.4
 27.9259259259253
 32.8412698412689
 25.7346938775527
 13.0769230769231
 9
 10.1578947368413
 9.94117647058752
 11.6315789473652
 20.7837837837811
 16.0666666666659
 23.2051282051316
 16.5625000000007
 17.4999999999967
 19.0526315789496
 10.7894736842129
 48.0602409638669
 82.8965517241691
 40.5696202531646
 41.3291139240532

 .000493204734765
 .000597232821262
 .000626504107083
 .00054678956413
 .000277730302512
 .000327385824194
 2.535869202534E-04
 2.297545680612E-04
 2.056277056276E-04
 .000398466442664
 .000349944008959
 .000388771482117
 .000275299603397
 3.140456936481E-04
 .000424899086467
 2.312166865435E-04
 .000931558508608
 .001094835397161
 .000847275847275
 .000823294009755

 23.4
 27.9259259259253
 32.8412698412689
 25.7346938775527
 13.0769230769231
 9
 10.1578947368413
 9.94117647058752
 11.6315789473652
 20.7837837837811
 16.0666666666659
 23.2051282051316
 16.5625000000007
 17.4999999999967
 19.0526315789496
 10.7894736842129
 48.0602409638669
 82.8965517241691
 40.5696202531646
 41.3291139240532

 .000032880315651
 3.31796011812E-05
 .00023866823127
 .000189702501841
 .000021363869424
 1.81881013441E-05
 0
 1.35149745918E-05
 .000021645021645
 3.23080899457E-05
 .000116648002986
 .00013955899358
 9.46342386675E-05
 7.85114234121E-05
 5.59077745351E-05
 8.51850950422E-05
 .000325484298189
 .000415282392027
 .00045045045045
 .000604443703364

 3
 3
 24
 17
 2
 1
 1
 1
 2
 3
 10
 14
 11
 7
 5
 7
 29
 44
 42
 58

 .000032880315651
 3.31796011812E-05
 .00023866823127
 .000189702501841
 .000021363869424
 1.81881013441E-05
 0
 1.35149745918E-05
 .000021645021645
 3.23080899457E-05
 .000116648002986
 .00013955899358
 9.46342386675E-05
 7.85114234121E-05
 5.59077745351E-05
 8.51850950422E-05
 .000325484298189
 .000415282392027
 .00045045045045
 .000604443703364

 3
 3
 24
 17
 2
 1
 1
 1
 2
 3
 10
 14
 11
 7
 5
 7
 29
 44
 42
 58

 .000854888206927
 .000785250561288
 .000815449790171
 .00061374338831
 .000769099299265
 .000854840763173
 .000347013680347
 .000189209644286
 .000335497835498
 .000807702248643
 .00059490481523
 .000588141472945
 .000507583643762
 .000336477528909
 .000503169970816
 .000438094774503
 .000897887719141
 .000953261854425
 .00138352638353
 .00102130142982

 78
 71
 82
 55
 72
 47
 26
 14
 31
 75
 51
 59
 59
 30
 45
 36
 80
 101
 129
 98

 .000854888206927
 .000785250561288
 .000815449790171
 .00061374338831
 .000769099299265
 .000854840763173
 .000347013680347
 .000189209644286
 .000335497835498
 .000807702248643
 .00059490481523
 .000588141472945
 .000507583643762
 .000336477528909
 .000503169970816
 .000438094774503
 .000897887719141
 .000953261854425
 .00138352638353
 .00102130142982

 78
 71
 82
 55
 72
 47
 26
 14
 31
 75
 51
 59
 59
 30
 45
 36
 80
 101
 129
 98

 5.48005260851E-05
 4.42394682416E-05
 .000188945683088
 .000189702501841
 .000032045804136
 1.81881013441E-05
 .00004004004004
 4.05449237755E-05
 5.41125541126E-05
 7.53855432067E-05
 .000186636804778
 .000109653494956
 .000146252914304
 4.48636705212E-05
 8.94524392562E-05
 8.51850950422E-05
 .000134683157871
 .000122697070371
 .000289575289575
 .000375171953812

 5
 4
 19
 17
 3
 1
 3
 3
 5
 7
 16
 11
 17
 4
 8
 7
 12
 13
 27
 36

 5.48005260851E-05
 4.42394682416E-05
 .000188945683088
 .000189702501841
 .000032045804136
 1.81881013441E-05
 .00004004004004
 4.05449237755E-05
 5.41125541126E-05
 7.53855432067E-05
 .000186636804778
 .000109653494956
 .000146252914304
 4.48636705212E-05
 8.94524392562E-05
 8.51850950422E-05
 .000134683157871
 .000122697070371
 .000289575289575
 .000375171953812

 5
 4
 19
 17
 3
 1
 3
 3
 5
 7
 16
 11
 17
 4
 8
 7
 12
 13
 27
 36

 5.48005260851E-05
 4.42394682416E-05
 .000188945683088
 .000189702501841
 .000032045804136
 1.81881013441E-05
 .00004004004004
 4.05449237755E-05
 5.41125541126E-05
 7.53855432067E-05
 .000186636804778
 .000109653494956
 .000146252914304
 4.48636705212E-05
 8.94524392562E-05
 8.51850950422E-05
 .000134683157871
 .000122697070371
 .000289575289575
 .000375171953812

 5
 4
 19
 17
 3
 1
 3
 3
 5
 7
 16
 11
 17
 4
 8
 7
 12
 13
 27
 36

 1.7097764138532E-03
 1.4930820531534E-03
 8.4528331908036E-04
 5.467895641301E-04
 4.486412579043E-04
 3.819501282265E-04
 1.868535201868E-04
 2.567845172452E-04
 4.437229437231E-04
 8.292409752744E-04
 7.232176185141E-04
 5.5823597432145E-04
 4.3875874291344E-04
 4.822844581026E-04
 .000335446647211
 1.460315915008E-04
 6.846393858446E-04
 7.0786771368212E-04
 7.400257400251E-04
 8.232940097536E-04

 43.6025641025689
 38.362962962959
 27.0941176470581
 25.1224489795864
 10.1904761904729
 4.90476190476305
 4.42857142857273
 5.42105263158237
 13.0000000000009
 30.9740259740144
 16.419354838709
 15.8928571428634
 18.3333333333409
 12.7209302325538
 16.0666666666784
 4.33333333333516
 39.7868852458855
 36.7333333333296
 25.5507246376786
 28.7721518987394

 .000460324419114
 3.649756129932E-04
 .000576781558902
 4.128819157716E-04
 1.175012818322E-04
 1.818810134414E-04
 .00004004004004
 1.351497459187E-04
 1.839826839825E-04
 1.938485396747E-04
 .000268290406868
 1.594959926632E-04
 1.4625291430414E-04
 1.682387644546E-04
 .000100633994163
 6.08464964587E-05
 .000538732631484
 .000443597100574
 4.611754611751E-04
 .000573179373879

 38.1904761904762
 20.8787878787879
 31.7931034482705
 31.4864864864857
 5.90909090909021
 5.20000000000176
 3
 6.80000000000178
 15.1176470588235
 13.0000000000052
 11.5217391304336
 8
 15.1176470588221
 11.5333333333342
 9
 5
 48
 47
 31.2790697674366
 36.2363636363764

 .000460324419114
 3.649756129932E-04
 .000576781558902
 4.128819157716E-04
 1.175012818322E-04
 1.818810134414E-04
 .00004004004004
 1.351497459187E-04
 1.839826839825E-04
 1.938485396747E-04
 .000268290406868
 1.594959926632E-04
 1.4625291430414E-04
 1.682387644546E-04
 .000100633994163
 6.08464964587E-05
 .000538732631484
 .000443597100574
 4.611754611751E-04
 .000573179373879

 38.1904761904762
 20.8787878787879
 31.7931034482705
 31.4864864864857
 5.90909090909021
 5.20000000000176
 3
 6.80000000000178
 15.1176470588235
 13.0000000000052
 11.5217391304336
 8
 15.1176470588221
 11.5333333333342
 9
 5
 48
 47
 31.2790697674366
 36.2363636363764

 .00043840420868
 .000475574283597
 9.94450963623E-06
 0
 .000138865151256
 9.09405067205E-05
 8.00800800801E-05
 4.05449237755E-05
 .000162337662338
 .000484621349186
 .000093318402389
 6.97794967901E-05
 .000060221788243
 5.60795881515E-05
 0
 0
 1.12235964893E-05
 9.43823618242E-06
 .000032175032175
 2.08428863229E-05

 40
 43
 1
 1
 13
 5
 6
 3
 15
 45
 8
 7
 7
 5
 5
 5
 1
 1
 3
 2

 .00043840420868
 .000475574283597
 9.94450963623E-06
 0
 .000138865151256
 9.09405067205E-05
 8.00800800801E-05
 4.05449237755E-05
 .000162337662338
 .000484621349186
 .000093318402389
 6.97794967901E-05
 .000060221788243
 5.60795881515E-05
 0
 0
 1.12235964893E-05
 9.43823618242E-06
 .000032175032175
 2.08428863229E-05

 40
 43
 1
 1
 13
 5
 6
 3
 15
 45
 8
 7
 7
 5
 5
 5
 1
 1
 3
 2

 6.57606313021E-05
 2.21197341208E-05
 9.94450963623E-06
 1.11589706965E-05
 0
 1.81881013441E-05
 0
 1.35149745918E-05
 1.08225108225E-05
 1.07693633152E-05
 3.49944008959E-05
 9.96849954145E-06
 0
 0
 0
 1.21692992917E-05
 0
 0
 0
 2.08428863229E-05

 6
 2
 1
 1
 1
 1
 1
 1
 1
 1
 3
 1
 1
 1
 1
 1
 1
 1
 1
 2

 6.57606313021E-05
 2.21197341208E-05
 9.94450963623E-06
 1.11589706965E-05
 0
 1.81881013441E-05
 0
 1.35149745918E-05
 1.08225108225E-05
 1.07693633152E-05
 3.49944008959E-05
 9.96849954145E-06
 0
 0
 0
 1.21692992917E-05
 0
 0
 0
 2.08428863229E-05

 6
 2
 1
 1
 1
 1
 1
 1
 1
 1
 3
 1
 1
 1
 1
 1
 1
 1
 1
 2

 9.86409469531E-05
 6.63592023624E-05
 3.97780385449E-05
 4.46358827862E-05
 5.34096735601E-05
 0
 1.33466800133E-05
 0
 1.08225108225E-05
 2.15387266305E-05
 2.33296005972E-05
 5.98109972487E-05
 1.72062252123E-05
 5.60795881515E-05
 .000011181554907
 1.21692992917E-05
 1.12235964893E-05
 3.77529447297E-05
 .000032175032175
 4.16857726458E-05

 9
 6
 4
 4
 5
 5
 1
 1
 1
 2
 2
 6
 2
 5
 1
 1
 1
 4
 3
 4

 9.86409469531E-05
 6.63592023624E-05
 3.97780385449E-05
 4.46358827862E-05
 5.34096735601E-05
 0
 1.33466800133E-05
 0
 1.08225108225E-05
 2.15387266305E-05
 2.33296005972E-05
 5.98109972487E-05
 1.72062252123E-05
 5.60795881515E-05
 .000011181554907
 1.21692992917E-05
 1.12235964893E-05
 3.77529447297E-05
 .000032175032175
 4.16857726458E-05

 9
 6
 4
 4
 5
 5
 1
 1
 1
 2
 2
 6
 2
 5
 1
 1
 1
 4
 3
 4

 .000646646207804
 .00056405322008
 .000208834702361
 7.81127948758E-05
 .000138865151256
 9.09405067205E-05
 5.33867200534E-05
 6.75748729592E-05
 7.57575757576E-05
 .000118462996468
 .000303284807764
 .000259180988078
 .000215077815154
 .000201886517345
 .000223631098141
 6.08464964587E-05
 .000123459561382
 .000217079432196
 .0002145002145
 .000166743090583

 59
 51
 21
 7
 13
 5
 4
 5
 7
 11
 26
 26
 25
 18
 20
 5
 11
 23
 20
 16

 .000646646207804
 .00056405322008
 .000208834702361
 7.81127948758E-05
 .000138865151256
 9.09405067205E-05
 5.33867200534E-05
 6.75748729592E-05
 7.57575757576E-05
 .000118462996468
 .000303284807764
 .000259180988078
 .000215077815154
 .000201886517345
 .000223631098141
 6.08464964587E-05
 .000123459561382
 .000217079432196
 .0002145002145
 .000166743090583

 59
 51
 21
 7
 13
 5
 4
 5
 7
 11
 26
 26
 25
 18
 20
 5
 11
 23
 20
 16

 .00486628671635
 .00541933485959
 .002167903100693
 .001484143102641
 .00207229533413
 .002691838998925
 .000800800800801
 7.298086279598E-04
 .003365800865801
 .005373912294305
 .002647909667789
 .00271143187527
 .002976676961722
 .001962785585303
 .001856138114567
 .001070898337674
 1.1111360524356E-03
 .001142026578077
 .0035178035178
 .003647505106507

 344.635135135085
 398.616326530596
 176.853211009154
 99.0150375939836
 166.319587628878
 127.635135135134
 54.3000000000034
 52.0370370370405
 229.816720257205
 363.29659318634
 198.982378854659
 233.242647058833
 294.531791907487
 154.382857142883
 86.9036144578305
 50.5681818181898
 87.7272727272642
 107.809917355389
 283.512195121951
 280.691428571391

 .00062472599737
 .00056405322008
 .000228723721633
 .000223179413931
 .00016022902068
 .000200069114785
 .00004004004004
 1.35149745918E-05
 .000519480519481
 .000872318428535
 .000174972004479
 .00020933849037
 .000240887152972
 .000123375093933
 .000726801068957
 .000328571080877
 6.73415789356E-05
 .000066067653277
 .0002574002574
 .000406436283297

 57
 51
 23
 20
 15
 11
 3
 1
 48
 81
 15
 21
 28
 11
 65
 27
 6
 7
 24
 39

 .00062472599737
 .00056405322008
 .000228723721633
 .000223179413931
 .00016022902068
 .000200069114785
 .00004004004004
 1.35149745918E-05
 .000519480519481
 .000872318428535
 .000174972004479
 .00020933849037
 .000240887152972
 .000123375093933
 .000726801068957
 .000328571080877
 6.73415789356E-05
 .000066067653277
 .0002574002574
 .000406436283297

 57
 51
 23
 20
 15
 11
 3
 1
 48
 81
 15
 21
 28
 11
 65
 27
 6
 7
 24
 39

 .00424156071898
 .00485528163951
 .00193917937906
 .00126096368871
 .00191206631345
 .00249176988414
 .000760760760761
 .000716293653368
 .00284632034632
 .00450159386577
 .00247293766331
 .0025020933849
 .00273578980875
 .00183941049137
 .00112933704561
 .000742327256797
 .0010437944735
 .0010759589248
 .0032604032604
 .00324106882321

 387
 439
 195
 113
 179
 137
 57
 53
 263
 418
 212
 251
 318
 164
 101
 61
 93
 114
 304
 311

 .00424156071898
 .00485528163951
 .00193917937906
 .00126096368871
 .00191206631345
 .00249176988414
 .000760760760761
 .000716293653368
 .00284632034632
 .00450159386577
 .00247293766331
 .0025020933849
 .00273578980875
 .00183941049137
 .00112933704561
 .000742327256797
 .0010437944735
 .0010759589248
 .0032604032604
 .00324106882321

 387
 439
 195
 113
 179
 137
 57
 53
 263
 418
 212
 251
 318
 164
 101
 61
 93
 114
 304
 311

 3.5839544059671E-03
 2.7649667651008E-03
 2.14801408142116E-03
 1.8635481063245E-03
 1.4634250555451E-03
 8.912169658613E-04
 1.0410410410417E-03
 8.784733484694E-04
 1.9913419913456E-03
 1.1415525114152E-03
 2.7528928704696E-03
 2.04354240599715E-03
 1.9098909985632E-03
 1.0094325867267E-03
 1.2523341495872E-03
 7.301579575052E-04
 6.285214033981E-04
 1.20809423134992E-03
 1.4157014157011E-03
 .001490266372088

 90.9816513761669
 69.5039999999971
 86.2777777776736
 66.2215568862587
 40.3868613138817
 12.5510204081621
 18.2564102564042
 14.6923076923061
 78.4456521739863
 28.4528301886738
 87.8305084745344
 58.9609756097494
 68.4954954955038
 31.488888888895
 53.5178571428563
 30.1000000000055
 22.0714285714262
 51.3125000000097
 48.0757575757714
 51.0699300699137

 .00219202104341
 .001647920192
 .00141212036834
 .001249804718015
 .000833190907537
 .000472890634947
 .000587253920587
 .000459509136122
 .00145021645022
 .000656931162229
 .00199468085106
 .001315841939471
 .001187229539647
 .000684170975448
 8.833428376552E-04
 5.597877674207E-04
 .000460167456059
 .000868317728783
 .001093951093951
 .001104672975114

 100.249999999999
 75.2550335570468
 113.605633802751
 84.5714285714286
 53.7692307692419
 15.7692307692259
 24.2272727272754
 19.8823529411836
 99.9701492537797
 39.4262295082074
 114.157894736816
 79.6363636363678
 82.0434782608883
 42.9672131147681
 67.9113924050608
 37.0869565217438
 26.9512195122049
 61.8478260869599
 59.6470588235407
 64.7924528301742

 .00219202104341
 .001647920192
 .00141212036834
 .001249804718015
 .000833190907537
 .000472890634947
 .000587253920587
 .000459509136122
 .00145021645022
 .000656931162229
 .00199468085106
 .001315841939471
 .001187229539647
 .000684170975448
 8.833428376552E-04
 5.597877674207E-04
 .000460167456059
 .000868317728783
 .001093951093951
 .001104672975114

 100.249999999999
 75.2550335570468
 113.605633802751
 84.5714285714286
 53.7692307692419
 15.7692307692259
 24.2272727272754
 19.8823529411836
 99.9701492537797
 39.4262295082074
 114.157894736816
 79.6363636363678
 82.0434782608883
 42.9672131147681
 67.9113924050608
 37.0869565217438
 26.9512195122049
 61.8478260869599
 59.6470588235407
 64.7924528301742

 .000021920210434
 0
 9.94450963623E-06
 0
 .000010681934712
 0
 0
 1.35149745918E-05
 0
 0
 1.16648002986E-05
 9.96849954145E-06
 6.88249008491E-05
 6.72955057818E-05
 0
 0
 1.12235964893E-05
 9.43823618242E-06
 .0000429000429
 4.16857726458E-05

 2
 2
 1
 1
 1
 1
 1
 1
 1
 1
 1
 1
 8
 6
 6
 6
 1
 1
 4
 4

 .000021920210434
 0
 9.94450963623E-06
 0
 .000010681934712
 0
 0
 1.35149745918E-05
 0
 0
 1.16648002986E-05
 9.96849954145E-06
 6.88249008491E-05
 6.72955057818E-05
 0
 0
 1.12235964893E-05
 9.43823618242E-06
 .0000429000429
 4.16857726458E-05

 2
 2
 1
 1
 1
 1
 1
 1
 1
 1
 1
 1
 8
 6
 6
 6
 1
 1
 4
 4

 .00105217010083
 .000851609763651
 2.98335289087E-05
 0
 .000277730302512
 .000181881013441
 .000146813480147
 8.10898475511E-05
 .000313852813853
 .000183079176359
 .000116648002986
 .000149527493122
 2.58093378184E-05
 2.24318352606E-05
 0
 0
 0
 0
 5.36250536251E-05
 5.21072158072E-05

 96
 77
 3
 3
 26
 10
 11
 6
 29
 17
 10
 15
 3
 2
 2
 2
 2
 2
 5
 5

 .00105217010083
 .000851609763651
 2.98335289087E-05
 0
 .000277730302512
 .000181881013441
 .000146813480147
 8.10898475511E-05
 .000313852813853
 .000183079176359
 .000116648002986
 .000149527493122
 2.58093378184E-05
 2.24318352606E-05
 0
 0
 0
 0
 5.36250536251E-05
 5.21072158072E-05

 96
 77
 3
 3
 26
 10
 11
 6
 29
 17
 10
 15
 3
 2
 2
 2
 2
 2
 5
 5

 .00021920210434
 9.95388035436E-05
 .000268501760178
 .000212020443234
 6.40916082721E-05
 3.63762026882E-05
 .000106773440107
 .000108119796735
 .00012987012987
 .000161540449729
 .000291620007465
 .000229275489453
 .000524789868975
 .000112159176303
 .000257175762862
 7.30157957505E-05
 3.36707894678E-05
 5.66294170945E-05
 .00010725010725
 .000135478761099

 20
 9
 27
 19
 6
 2
 8
 8
 12
 15
 25
 23
 61
 10
 23
 6
 3
 6
 10
 13

 .00021920210434
 9.95388035436E-05
 .000268501760178
 .000212020443234
 6.40916082721E-05
 3.63762026882E-05
 .000106773440107
 .000108119796735
 .00012987012987
 .000161540449729
 .000291620007465
 .000229275489453
 .000524789868975
 .000112159176303
 .000257175762862
 7.30157957505E-05
 3.36707894678E-05
 5.66294170945E-05
 .00010725010725
 .000135478761099

 20
 9
 27
 19
 6
 2
 8
 8
 12
 15
 25
 23
 61
 10
 23
 6
 3
 6
 10
 13

 9.86409469531E-05
 .000132718404725
 .000417669404722
 .000390563974379
 .0002670483678
 .000181881013441
 .000173506840174
 .000175694669694
 7.57575757576E-05
 9.69242698372E-05
 .000233296005972
 .000279117987161
 7.74280134553E-05
 8.97273410424E-05
 .00011181554907
 .000097354394334
 .000123459561382
 .00027370884929
 .000117975117975
 .000156321647422

 9
 12
 42
 35
 25
 10
 13
 13
 7
 9
 20
 28
 9
 8
 10
 8
 11
 29
 11
 15

 9.86409469531E-05
 .000132718404725
 .000417669404722
 .000390563974379
 .0002670483678
 .000181881013441
 .000173506840174
 .000175694669694
 7.57575757576E-05
 9.69242698372E-05
 .000233296005972
 .000279117987161
 7.74280134553E-05
 8.97273410424E-05
 .00011181554907
 .000097354394334
 .000123459561382
 .00027370884929
 .000117975117975
 .000156321647422

 9
 12
 42
 35
 25
 10
 13
 13
 7
 9
 20
 28
 9
 8
 10
 8
 11
 29
 11
 15

 0
 3.31796011812E-05
 9.94450963623E-06
 1.11589706965E-05
 .000010681934712
 1.81881013441E-05
 2.66933600267E-05
 4.05449237755E-05
 .000021645021645
 .000043077453261
 .000104983202688
 5.98109972487E-05
 2.58093378184E-05
 3.36477528909E-05

 0
 3
 1
 1
 1
 1
 2
 3
 2
 4
 9
 6
 3
 3

 0
 3.31796011812E-05
 9.94450963623E-06
 1.11589706965E-05
 .000010681934712
 1.81881013441E-05
 2.66933600267E-05
 4.05449237755E-05
 .000021645021645
 .000043077453261
 .000104983202688
 5.98109972487E-05
 2.58093378184E-05
 3.36477528909E-05

 0
 3
 1
 1
 1
 1
 2
 3
 2
 4
 9
 6
 3
 3

 6.8062253397682E-03
 3.5502173263836E-03
 7.10037988026523E-03
 7.1975360992695E-03
 2.9375320458061E-03
 2.4917698841431E-03
 2.3890557223933E-03
 2.0002162395974E-03
 1.2337662337675E-03
 2.0138709399452E-03
 4.5492721164576E-03
 3.85780932254315E-03
 3.28638901555068E-03
 3.1292410188506E-03
 .0050987890376
 .00292063183002
 .008080989472268
 3.3977650256677E-03
 .00487987987988
 5.5650506482176E-03

 593.48631239936
 303.355140186904
 622.591036414551
 584.079069767429
 240.883636363661
 121.83211678831
 177.011173184363
 140.216216216223
 108.12280701755
 185.010695187167
 344.189743589667
 355.956072351434
 372.094240837706
 275.028673835123
 356.25
 165.633333333359
 591.5527777778
 301.066666666616
 398.006593406621
 483.992509363319

 6.8062253397682E-03
 3.5502173263836E-03
 7.10037988026523E-03
 7.1975360992695E-03
 2.9375320458061E-03
 2.4917698841431E-03
 2.3890557223933E-03
 2.0002162395974E-03
 1.2337662337675E-03
 2.0138709399452E-03
 4.5492721164576E-03
 3.85780932254315E-03
 3.28638901555068E-03
 3.1292410188506E-03
 .0050987890376
 .00292063183002
 .008080989472268
 3.3977650256677E-03
 .00487987987988
 5.5650506482176E-03

 593.48631239936
 303.355140186904
 622.591036414551
 584.079069767429
 240.883636363661
 121.83211678831
 177.011173184363
 140.216216216223
 108.12280701755
 185.010695187167
 344.189743589667
 355.956072351434
 372.094240837706
 275.028673835123
 356.25
 165.633333333359
 591.5527777778
 301.066666666616
 398.006593406621
 483.992509363319

 6.7075843928151E-03
 3.4949179910816E-03
 6.62304341772623E-03
 6.8516080076765E-03
 2.7986668945501E-03
 2.3644531747341E-03
 .00237570904238
 2.0002162395974E-03
 .001222943722945
 2.0138709399452E-03
 .004374300111978
 3.7780613262116E-03
 3.25197656512614E-03
 3.1292410188506E-03
 .0044614404079
 .0023608440626
 .00744124447238
 3.1334944125597E-03
 .00468682968683
 5.3774646713116E-03

 602.081699346404
 308.101265822779
 664.003003003003
 612.003257328994
 252.190839694658
 128.015384615385
 178
 140.216216216223
 109.070796460181
 185.010695187167
 357.431999999957
 363.337730870717
 376.005291005293
 275.028673835123
 399
 194
 637.509803921602
 324.096385542162
 413.659038901614
 500.248062015509

 9.86409469531E-05
 4.42394682416E-05
 0
 0
 .000138865151256
 .000127316709409
 1.33466800133E-05
 0
 0
 0
 .000163307204181
 6.97794967901E-05
 8.60311260614E-06

 9
 4
 4
 4
 13
 7
 1
 1
 1
 1
 14
 7
 1

 0
 1.10598670604E-05
 .000477336462539
 .000345928091593
 0
 0
 0
 0
 1.08225108225E-05
 0
 1.16648002986E-05
 9.96849954145E-06
 2.58093378184E-05
 0
 .0006373486297
 .00055978776742
 .000639744999888
 .000264270613108
 .00019305019305
 .000187585976906

 0
 1
 48
 31
 31
 31
 31
 31
 1
 1
 1
 1
 3
 3
 57
 46
 57
 28
 18
 18

 .000186321788689
 .000232257208268
 .000785616261262
 .00058026647622
 .000352503845496
 .000163692912097
 .000266933600267
 .000310844415612
 3.24675324675E-05
 7.53855432067E-05
 .000478256812243
 .000338928984409
 .00026669649079
 .000213102434976
 .000659711739514
 .00020687808796
 .000044894385957
 3.77529447297E-05
 .000782925782926
 .000812872566593

 17
 21
 79
 52
 33
 9
 20
 23
 3
 7
 41
 34
 31
 19
 59
 17
 4
 4
 73
 78

 .000186321788689
 .000232257208268
 .000785616261262
 .00058026647622
 .000352503845496
 .000163692912097
 .000266933600267
 .000310844415612
 3.24675324675E-05
 7.53855432067E-05
 .000478256812243
 .000338928984409
 .00026669649079
 .000213102434976
 .000659711739514
 .00020687808796
 .000044894385957
 3.77529447297E-05
 .000782925782926
 .000812872566593

 17
 21
 79
 52
 33
 9
 20
 23
 3
 7
 41
 34
 31
 19
 59
 17
 4
 4
 73
 78

 .000186321788689
 .000232257208268
 .000785616261262
 .00058026647622
 .000352503845496
 .000163692912097
 .000266933600267
 .000310844415612
 3.24675324675E-05
 7.53855432067E-05
 .000478256812243
 .000338928984409
 .00026669649079
 .000213102434976
 .000659711739514
 .00020687808796
 .000044894385957
 3.77529447297E-05
 .000782925782926
 .000812872566593

 17
 21
 79
 52
 33
 9
 20
 23
 3
 7
 41
 34
 31
 19
 59
 17
 4
 4
 73
 78

 2.4550635686091E-03
 9.843281683758E-04
 6.5633763599093E-04
 4.909947106486E-04
 .001175012818321
 5.638311416677E-04
 3.870537203866E-04
 .000175694669694
 2.922077922081E-04
 3.661583527178E-04
 1.2714632325496E-03
 .001116471948643
 1.0237704001313E-03
 5.832277167752E-04
 6.485301846073E-04
 4.989412709614E-04
 2.805899122318E-04
 1.0382059800652E-04
 .001501501501502
 1.8341739964148E-03

 159.732142857148
 54.4606741572842
 29.3030303030285
 21.1363636363631
 32.636363636375
 11.7096774193551
 6.655172413789
 3.46153846153859
 13.7407407407447
 15.9999999999989
 46.6513761467654
 48.0357142856938
 52.7142857142737
 18.5769230769255
 23.8275862069025
 13.9268292682927
 14.1200000000053
 4.09090909091033
 57.4857142857203
 77.7613636363631

 1.096010521701E-04
 6.63592023624E-05
 3.977803854493E-05
 3.34769120896E-05
 .000598188343873
 2.000691147854E-04
 1.334668001335E-04
 6.75748729592E-05
 1.08225108225E-05
 4.30774532609E-05
 .000303284807765
 .000299054986244
 3.183151664273E-04
 1.682387644542E-04
 1.900864334191E-04
 1.582008907927E-04
 2.24471929785E-05
 1.88764723648E-05
 .000139425139425
 1.146358747759E-04

 8.20000000000073
 4.33333333333333
 2.50000000000013
 1.66666666666766
 39.5714285714393
 5.90909090909255
 5.2
 2.5999999999997
 1
 2.50000000000116
 14.2307692307662
 15
 33.2162162162148
 9.13333333333
 13.4705882352881
 11.1538461538494
 2
 2
 8.38461538461538
 7.72727272727217

 1.096010521701E-04
 6.63592023624E-05
 3.977803854493E-05
 3.34769120896E-05
 .000598188343873
 2.000691147854E-04
 1.334668001335E-04
 6.75748729592E-05
 1.08225108225E-05
 4.30774532609E-05
 .000303284807765
 .000299054986244
 3.183151664273E-04
 1.682387644542E-04
 1.900864334191E-04
 1.582008907927E-04
 2.24471929785E-05
 1.88764723648E-05
 .000139425139425
 1.146358747759E-04

 8.20000000000073
 4.33333333333333
 2.50000000000013
 1.66666666666766
 39.5714285714393
 5.90909090909255
 5.2
 2.5999999999997
 1
 2.50000000000116
 14.2307692307662
 15
 33.2162162162148
 9.13333333333
 13.4705882352881
 11.1538461538494
 2
 2
 8.38461538461538
 7.72727272727217

 .00204953967558
 .000752070960107
 .000318224308359
 .000301292208807
 .000309776106648
 .00030919772285
 .000133466800133
 5.40598983674E-05
 9.74025974026E-05
 7.53855432067E-05
 .000174972004479
 .000129590494039
 .00060221788243
 .000302829776018
 .000368991311932
 .000231216686543
 .000202024736807
 1.88764723648E-05
 .000782925782926
 .000979615657176

 187
 68
 32
 27
 29
 17
 10
 4
 9
 7
 15
 13
 70
 27
 33
 19
 18
 2
 73
 94

 .00204953967558
 .000752070960107
 .000318224308359
 .000301292208807
 .000309776106648
 .00030919772285
 .000133466800133
 5.40598983674E-05
 9.74025974026E-05
 7.53855432067E-05
 .000174972004479
 .000129590494039
 .00060221788243
 .000302829776018
 .000368991311932
 .000231216686543
 .000202024736807
 1.88764723648E-05
 .000782925782926
 .000979615657176

 187
 68
 32
 27
 29
 17
 10
 4
 9
 7
 15
 13
 70
 27
 33
 19
 18
 2
 73
 94

 .000295922840859
 1.658980059064E-04
 .000298335289087
 .000156225589752
 .0002670483678
 5.45643040323E-05
 1.201201201201E-04
 5.40598983674E-05
 .000183982683983
 2.476953562502E-04
 7.932064203056E-04
 .00068782646836
 .000103237351274
 .000112159176303
 8.94524392562E-05
 1.095236936257E-04
 5.61179824463E-05
 6.606765327692E-05
 .000579150579151
 7.399224644629E-04

 27
 13.1333333333354
 30
 14
 21.32
 3
 4.55555555555537
 4
 17
 21.0869565217413
 66.0294117647069
 69
 12
 10
 8
 7.22222222222506
 5
 5.28571428571407
 48.3333333333379
 67.1126760563379

 .000295922840859
 .000154838138846
 .000298335289087
 .000156225589752
 .000245684498376
 5.45643040323E-05
 5.33867200534E-05
 5.40598983674E-05
 .000183982683983
 .000236925992935
 .000781541620007
 .00068782646836
 .000103237351274
 .000112159176303
 8.94524392562E-05
 .000097354394334
 5.61179824463E-05
 5.66294170945E-05
 .000546975546976
 .00071907957814

 27
 14
 30
 14
 23
 3
 4
 4
 17
 22
 67
 69
 12
 10
 8
 8
 5
 6
 51
 69

 0
 1.10598670604E-05
 0
 0
 .000021363869424
 0
 6.67334000667E-05
 0
 0
 1.07693633152E-05
 1.16648002986E-05
 0
 0
 0
 0
 1.21692992917E-05
 0
 9.43823618242E-06
 .000032175032175
 2.08428863229E-05

 0
 1
 1
 1
 2
 2
 5
 5
 5
 1
 1
 1
 1
 1
 1
 1
 1
 1
 3
 2

 .044432266549806
 3.14874415209574E-02
 4.50486286520353E-03
 .004965741959955
 4.7107332079991E-03
 5.0744802750034E-03
 .002802802802804
 2.8516596388789E-03
 4.7510822510751E-03
 7.6570173171289E-03
 6.1240201567796E-03
 5.7318872363322E-03
 1.27154004318833E-02
 .009802712008885
 .003600460680058
 .003017986224356
 5.7240342095233E-03
 4.50203865900904E-03
 .01214071214072
 1.27975322022569E-02

 2061.27577701107
 1324.23357920614
 152.97350993376
 147.633707865132
 228.469387755164
 136.942652329795
 93.7714285714567
 81.4360189573273
 195.405466970414
 331.967651195517
 202.379047619094
 216.979130434759
 607.692828146225
 363.100686498834
 132.217391304335
 146.153225806519
 222.262745098029
 200.375262054495
 333.515901060119
 348.159609120454

 .00974353353792
 .00896955218598
 .00204856898506
 .00218715825652
 .00327935395659
 .00336479874866
 .00174841508175
 .00147313223051
 .00277056277056
 .00463082622555
 .00332446808511
 .00283105386977
 .00578129167133
 .00434056012293
 .00216922165196
 .00227565896756
 .00285079350827
 .00207641196013
 .00523380523381
 .0050543999333

 889
 811
 206
 196
 307
 185
 131
 109
 256
 430
 285
 284
 672
 387
 194
 187
 254
 220
 488
 485

 .00974353353792
 .00896955218598
 .00204856898506
 .00218715825652
 .00327935395659
 .00336479874866
 .00174841508175
 .00147313223051
 .00277056277056
 .00463082622555
 .00332446808511
 .00283105386977
 .00578129167133
 .00434056012293
 .00216922165196
 .00227565896756
 .00285079350827
 .00207641196013
 .00523380523381
 .0050543999333

 889
 811
 206
 196
 307
 185
 131
 109
 256
 430
 285
 284
 672
 387
 194
 187
 254
 220
 488
 485

 .0298662867164
 .0193547673557
 .000795560770898
 .000636061329703
 .000747735429841
 .0010730979793
 .000547213880547
 .000905503297654
 .00148268398268
 .00239079865598
 .00153975363942
 .0019239204115
 .00569526054527
 .0045536625579
 .000201267988326
 7.30157957505E-05
 .000179577543828
 .000141573542736
 .00152295152295
 .00180290966693

 2725
 1750
 80
 57
 70
 59
 41
 67
 137
 222
 132
 193
 662
 406
 18
 6
 16
 15
 142
 173

 .0298662867164
 .0193547673557
 .000795560770898
 .000636061329703
 .000747735429841
 .0010730979793
 .000547213880547
 .000905503297654
 .00148268398268
 .00239079865598
 .00153975363942
 .0019239204115
 .00569526054527
 .0045536625579
 .000201267988326
 7.30157957505E-05
 .000179577543828
 .000141573542736
 .00152295152295
 .00180290966693

 2725
 1750
 80
 57
 70
 59
 41
 67
 137
 222
 132
 193
 662
 406
 18
 6
 16
 15
 142
 173

 .000383603682595
 .000298616410631
 .000139223134907
 .000312451179503
 .000277730302512
 .000236445317473
 .000146813480147
 6.75748729592E-05
 5.41125541126E-05
 8.61549065219E-05
 .000151642403882
 .000109653494956
 .000335521391639
 .000134591011564
 .000190086433419
 7.30157957505E-05
 .000101012368403
 .000132135306554
 .000160875160875
 .000270957522198

 35
 27
 14
 28
 26
 13
 11
 5
 5
 8
 13
 11
 39
 12
 17
 6
 9
 14
 15
 26

 .000383603682595
 .000298616410631
 .000139223134907
 .000312451179503
 .000277730302512
 .000236445317473
 .000146813480147
 6.75748729592E-05
 5.41125541126E-05
 8.61549065219E-05
 .000151642403882
 .000109653494956
 .000335521391639
 .000134591011564
 .000190086433419
 7.30157957505E-05
 .000101012368403
 .000132135306554
 .000160875160875
 .000270957522198

 35
 27
 14
 28
 26
 13
 11
 5
 5
 8
 13
 11
 39
 12
 17
 6
 9
 14
 15
 26

 .004120999561598
 .002477410221532
 .00141212036834
 .00169616354587
 .000352503845496
 3.637620268822E-04
 .00036036036036
 .000378419288572
 .000411255411255
 .000441543895925
 1.0148376259786E-03
 .000817416962399
 6.968521210973E-04
 .000504716293364
 .00052553308063
 .000316401781585
 2.4804148241293E-03
 2.04809725158242E-03
 .00209137709138
 .001823752553256

 368.085106382984
 214.223214285719
 142
 152
 27.4242424242424
 16.4000000000009
 27
 28
 38
 41
 85.0229885057475
 82
 77.0987654320964
 45
 47
 26
 219.009049773753
 215.00921658986
 176.025641025663
 154.382857142865

 .004120999561598
 .002477410221532
 .00141212036834
 .00169616354587
 .000331139976072
 3.637620268822E-04
 .00036036036036
 .000378419288572
 .000411255411255
 .000441543895925
 1.0148376259786E-03
 .000817416962399
 .000679645895885
 .000504716293364
 .00052553308063
 .000316401781585
 .00246919122764
 2.04809725158242E-03
 .00198412698413
 .00170911667848

 368.085106382984
 214.223214285719
 142
 152
 29.0645161290323
 16.4000000000009
 27
 28
 38
 41
 85.0229885057475
 82
 79
 45
 47
 26
 220
 215.00921658986
 185
 164

 0
 0
 0
 0
 .000021363869424
 0
 0
 0
 0
 0
 0
 0
 1.72062252123E-05
 0
 0
 0
 1.12235964893E-05
 0
 .00010725010725
 .000114635874776

 0
 0
 0
 0
 2
 2
 2
 2
 2
 2
 2
 2
 2
 2
 2
 2
 1
 1
 10
 11

 .000317843051293
 .000376035480054
 9.94450963623E-05
 .000133907648359
 5.34096735601E-05
 3.63762026882E-05
 0
 2.70299491837E-05
 3.24675324675E-05
 .000107693633152
 5.83240014931E-05
 2.99054986243E-05
 .000206474702547
 .000269182023127
 .000514351525723
 .00027989388371
 .000112235964893
 9.43823618242E-05
 .00312097812098
 .00382466964025

 29
 34
 10
 12
 5
 2
 2
 2
 3
 10
 5
 3
 24
 24
 46
 23
 10
 10
 291
 367

 .000317843051293
 .000376035480054
 9.94450963623E-05
 .000133907648359
 5.34096735601E-05
 3.63762026882E-05
 0
 2.70299491837E-05
 3.24675324675E-05
 .000107693633152
 5.83240014931E-05
 2.99054986243E-05
 .000206474702547
 .000269182023127
 .000514351525723
 .00027989388371
 .000112235964893
 9.43823618242E-05
 .00312097812098
 .00382466964025

 29
 34
 10
 12
 5
 2
 2
 2
 3
 10
 5
 3
 24
 24
 46
 23
 10
 10
 291
 367

 0
 1.10598670604E-05
 9.94450963623E-06
 0
 0
 0
 0
 0
 0
 0
 3.49944008959E-05
 1.99369990829E-05
 0
 0
 0
 0
 0
 9.43823618242E-06
 .000010725010725
 2.08428863229E-05

 0
 1
 1
 1
 1
 1
 1
 1
 1
 1
 3
 2
 2
 2
 2
 2
 2
 1
 1
 2

 0
 1.10598670604E-05
 9.94450963623E-06
 0
 0
 0
 0
 0
 0
 0
 3.49944008959E-05
 1.99369990829E-05
 0
 0
 0
 0
 0
 9.43823618242E-06
 .000010725010725
 2.08428863229E-05

 0
 1
 1
 1
 1
 1
 1
 1
 1
 1
 3
 2
 2
 2
 2
 2
 2
 1
 1
 2

 1.096010521701E-04
 .000055299335302
 9.94450963623E-06
 3.34769120896E-05
 .000032045804136
 7.27524053764E-05
 .00008008008008
 9.46048221429E-05
 .00004329004329
 2.15387266304E-05
 6.99888017917E-05
 6.97794967901E-05
 1.720622521228E-05
 0
 2.23631098141E-05
 2.43385985835E-05
 4.48943859571E-05
 8.49441256418E-05
 .00002145002145
 4.16857726458E-05

 5.8000000000011
 3.4
 1
 3
 3
 4
 4.333333333335
 3.57142857142887
 4
 1
 4.33333333333429
 3.57142857142898
 1
 1
 2
 2
 2.49999999999889
 4.55555555555542
 2
 4

 7.67207365191E-05
 4.42394682416E-05
 9.94450963623E-06
 3.34769120896E-05
 .000032045804136
 7.27524053764E-05
 6.67334000667E-05
 4.05449237755E-05
 .00004329004329
 1.07693633152E-05
 5.83240014931E-05
 3.98739981658E-05
 8.60311260614E-06
 0
 2.23631098141E-05
 2.43385985835E-05
 3.36707894678E-05
 4.71911809121E-05
 .00002145002145
 4.16857726458E-05

 7
 4
 1
 3
 3
 4
 5
 3
 4
 1
 5
 4
 1
 1
 2
 2
 3
 5
 2
 4

 7.67207365191E-05
 4.42394682416E-05
 9.94450963623E-06
 3.34769120896E-05
 .000032045804136
 7.27524053764E-05
 6.67334000667E-05
 4.05449237755E-05
 .00004329004329
 1.07693633152E-05
 5.83240014931E-05
 3.98739981658E-05
 8.60311260614E-06
 0
 2.23631098141E-05
 2.43385985835E-05
 3.36707894678E-05
 4.71911809121E-05
 .00002145002145
 4.16857726458E-05

 7
 4
 1
 3
 3
 4
 5
 3
 4
 1
 5
 4
 1
 1
 2
 2
 3
 5
 2
 4

 .000032880315651
 1.10598670604E-05
 0
 0
 0
 0
 1.33466800133E-05
 5.40598983674E-05
 0
 1.07693633152E-05
 1.16648002986E-05
 2.99054986243E-05
 8.60311260614E-06
 0
 0
 0
 1.12235964893E-05
 3.77529447297E-05

 3
 1
 1
 1
 1
 1
 1
 4
 4
 1
 1
 3
 1
 1
 1
 1
 1
 4

 .000032880315651
 1.10598670604E-05
 0
 0
 0
 0
 1.33466800133E-05
 5.40598983674E-05
 0
 1.07693633152E-05
 1.16648002986E-05
 2.99054986243E-05
 8.60311260614E-06
 0
 0
 0
 1.12235964893E-05
 3.77529447297E-05

 3
 1
 1
 1
 1
 1
 1
 4
 4
 1
 1
 3
 1
 1
 1
 1
 1
 4

 1.48619026742591E-02
 .013824833825496
 3.3314107281309E-03
 2.9124913517973E-03
 .000566142539737
 5.092668376352E-04
 3.336670003339E-04
 5.000540598979E-04
 9.090909090906E-04
 1.1092444214695E-03
 1.9713512504668E-03
 1.72455042067085E-03
 1.64319450777328E-03
 1.2337509393336E-03
 2.1133138774302E-03
 1.4603159150094E-03
 1.6947630698788E-03
 1.6988825128376E-03
 2.4667524667511E-03
 2.3552461544877E-03

 519.163716814227
 461.764799999909
 125.346268656684
 122.754789272044
 40.2830188679346
 24.2142857142871
 19.4800000000106
 25.3243243243221
 36.3095238095285
 52.3592233009771
 125.75739644971
 111.832369942189
 87.6492146596848
 54.5090909090877
 85.3492063492203
 60.0500000000006
 75.8344370860928
 120.93333333333
 100.408695652157
 109.35398230087

 .00266330556773
 .00243317075329
 .00044750293363
 .000189702501841
 0
 0
 1.33466800133E-05
 0
 .000021645021645
 3.23080899457E-05
 3.49944008959E-05
 .000129590494039
 .000060221788243
 1.12159176303E-05
 5.59077745351E-05
 1.21692992917E-05
 .000549956227974
 .000302023557838
 .000740025740026
 .000604443703364

 243
 220
 45
 17
 17
 17
 1
 1
 2
 3
 3
 13
 7
 1
 5
 1
 49
 32
 69
 58

 .00266330556773
 .00243317075329
 .00044750293363
 .000189702501841
 0
 0
 1.33466800133E-05
 0
 .000021645021645
 3.23080899457E-05
 3.49944008959E-05
 .000129590494039
 .000060221788243
 1.12159176303E-05
 5.59077745351E-05
 1.21692992917E-05
 .000549956227974
 .000302023557838
 .000740025740026
 .000604443703364

 243
 220
 45
 17
 17
 17
 1
 1
 2
 3
 3
 13
 7
 1
 5
 1
 49
 32
 69
 58

 .000010960105217
 6.63592023624E-05
 7.95560770898E-05
 2.23179413931E-05
 .000032045804136
 1.81881013441E-05
 1.33466800133E-05
 0
 .00004329004329
 6.46161798914E-05
 8.16536020903E-05
 3.98739981658E-05
 8.60311260614E-06
 0
 5.59077745351E-05
 1.21692992917E-05
 3.36707894678E-05
 1.88764723648E-05
 7.50750750751E-05
 6.25286589687E-05

 1
 6
 8
 2
 3
 1
 1
 1
 4
 6
 7
 4
 1
 1
 5
 1
 3
 2
 7
 6

 .000010960105217
 6.63592023624E-05
 7.95560770898E-05
 2.23179413931E-05
 .000032045804136
 1.81881013441E-05
 1.33466800133E-05
 0
 .00004329004329
 6.46161798914E-05
 8.16536020903E-05
 3.98739981658E-05
 8.60311260614E-06
 0
 5.59077745351E-05
 1.21692992917E-05
 3.36707894678E-05
 1.88764723648E-05
 7.50750750751E-05
 6.25286589687E-05

 1
 6
 8
 2
 3
 1
 1
 1
 4
 6
 7
 4
 1
 1
 5
 1
 3
 2
 7
 6

 .00464708461201
 .00470044350067
 .00110384056962
 .000825763831544
 .000032045804136
 1.81881013441E-05
 1.33466800133E-05
 8.10898475511E-05
 .00021645021645
 .000183079176359
 .000151642403882
 6.97794967901E-05
 .000851708148008
 .000504716293364
 .00093925061219
 .000851850950422
 .000044894385957
 1.88764723648E-05
 .00019305019305
 .000177164533745

 424
 425
 111
 74
 3
 1
 1
 6
 20
 17
 13
 7
 99
 45
 84
 70
 4
 2
 18
 17

 .00464708461201
 .00470044350067
 .00110384056962
 .000825763831544
 .000032045804136
 1.81881013441E-05
 1.33466800133E-05
 8.10898475511E-05
 .00021645021645
 .000183079176359
 .000151642403882
 6.97794967901E-05
 .000851708148008
 .000504716293364
 .00093925061219
 .000851850950422
 .000044894385957
 1.88764723648E-05
 .00019305019305
 .000177164533745

 424
 425
 111
 74
 3
 1
 1
 6
 20
 17
 13
 7
 99
 45
 84
 70
 4
 2
 18
 17

 .007474791758
 6.5916807679924E-03
 .00165078859961
 1.8189122235365E-03
 .000502050931465
 .000472890634947
 .000293626960294
 .000405449237755
 6.060606060606E-04
 7.969328853277E-04
 .0016913960433
 1.3955899358029E-03
 7.2266145891614E-04
 7.178187283393E-04
 .00106224771617
 .000584126366004
 .00106624166648
 .00135910601027
 .0014586014586
 .00151110925841

 682
 584.120805369122
 166
 161.012269938654
 45.0425531914913
 26
 22
 30
 46.8928571428536
 68.2432432432447
 145
 136.057142857143
 82.02380952381
 62.0312500000001
 95
 48
 95
 144
 132.058823529412
 145

 .007474791758
 6.5916807679924E-03
 .00165078859961
 1.8189122235365E-03
 .000502050931465
 .000472890634947
 .000293626960294
 .000405449237755
 6.060606060606E-04
 7.969328853277E-04
 .0016913960433
 1.3955899358029E-03
 7.2266145891614E-04
 7.178187283393E-04
 .00106224771617
 .000584126366004
 .00106624166648
 .00135910601027
 .0014586014586
 .00151110925841

 682
 584.120805369122
 166
 161.012269938654
 45.0425531914913
 26
 22
 30
 46.8928571428536
 68.2432432432447
 145
 136.057142857143
 82.02380952381
 62.0312500000001
 95
 48
 95
 144
 132.058823529412
 145

 6.57606313021E-05
 3.31796011812E-05
 0
 0
 0
 0
 0
 1.35149745918E-05
 .000021645021645
 3.23080899457E-05
 1.16648002986E-05
 7.97479963316E-05

 6
 3
 3
 3
 3
 3
 3
 1
 2
 3
 1
 8

 6.57606313021E-05
 3.31796011812E-05
 0
 0
 0
 0
 0
 1.35149745918E-05
 .000021645021645
 3.23080899457E-05
 1.16648002986E-05
 7.97479963316E-05

 6
 3
 3
 3
 3
 3
 3
 1
 2
 3
 1
 8

 0
 0
 4.97225481811E-05
 5.57948534827E-05
 0
 0
 0
 0
 0
 0
 0
 9.96849954145E-06

 0
 0
 5
 5
 5
 5
 5
 5
 5
 5
 5
 1

 0
 0
 4.97225481811E-05
 5.57948534827E-05
 0
 0
 0
 0
 0
 0
 0
 9.96849954145E-06

 0
 0
 5
 5
 5
 5
 5
 5
 5
 5
 5
 1

 .00217010083296
 .00247741022153
 .003062908967957
 .003358850179664
 .000395231584344
 .000563831141667
 4.804804804801E-04
 5.270840090822E-04
 .000411255411255
 .00067846988886
 .002029675251958
 .0028609593684
 .004181112726585
 .004587310310789
 1.5318730222622E-03
 .001740209798716
 .002716110350399
 .00628586529749
 .00758258258258
 .00727416732669

 99
 114.285714285696
 267.142857142851
 255.152823920262
 20.1351351351351
 15.9032258064539
 25.9999999999958
 30.2820512820544
 21.5789473684211
 39.1269841269754
 88.9425287356204
 152.282229965108
 421.041152263371
 351.699266503669
 125.525547445251
 126.13286713283
 190.950413223094
 610.525525525536
 631.526166902428
 596.667621776471

 .00108505041648
 .0010617472378
 .00284412975596
 .00307987591225
 .000138865151256
 .000327385824194
 .0004004004004
 .000459509136123
 .000281385281385
 .000506160075816
 .00116648002986
 .00178436141792
 .00388000378537
 .00423961686425
 .00146478369282
 .00163068610509
 .00239062605221
 .0060121564482
 .00715358215358
 .00670098795281

 99
 96
 286
 276
 13
 18
 30
 34
 26
 47
 100
 179
 451
 378
 131
 134
 213
 637
 667
 643

 .00108505041648
 .0010617472378
 .00284412975596
 .00307987591225
 .000138865151256
 .000327385824194
 .0004004004004
 .000459509136123
 .000281385281385
 .000506160075816
 .00116648002986
 .00178436141792
 .00388000378537
 .00423961686425
 .00146478369282
 .00163068610509
 .00239062605221
 .0060121564482
 .00715358215358
 .00670098795281

 99
 96
 286
 276
 13
 18
 30
 34
 26
 47
 100
 179
 451
 378
 131
 134
 213
 637
 667
 643

 .00108505041648
 .00141566298373
 .000218779211997
 .000278974267414
 .000256366433088
 .000236445317473
 8.00800800801E-05
 6.75748729592E-05
 .00012987012987
 .000172309813044
 .000863195222098
 .00107659795048
 .000301108941215
 .000347693446539
 6.70893294422E-05
 .000109523693626
 .000325484298189
 .00027370884929
 .000429000429
 .00057317937388

 99
 128
 22
 25
 24
 13
 6
 5
 12
 16
 74
 108
 35
 31
 6
 9
 29
 29
 40
 55

 .00108505041648
 .00141566298373
 .000218779211997

 99
 128
 22

 0
 0
 0
 .000278974267414
 .000256366433088
 .000236445317473
 8.00800800801E-05
 6.75748729592E-05
 .00012987012987
 .000172309813044
 .000863195222098
 .00107659795048
 .000301108941215
 .000347693446539
 6.70893294422E-05
 .000109523693626
 .000325484298189
 .00027370884929
 .000429000429
 .00057317937388

 0
 0
 0
 25
 24
 13
 6
 5
 12
 16
 74
 108
 35
 31
 6
 9
 29
 29
 40
 55

 3.9456378781231E-03
 2.8534457015786E-03
 4.3358062013969E-03
 3.1691476778224E-03
 1.3245599042891E-03
 1.3641076008041E-03
 .001214547881214
 8.108984755109E-04
 1.0606060606065E-03
 1.7984836736467E-03
 3.0795072788312E-03
 2.41237688903345E-03
 3.1401361012443E-03
 1.9964333381932E-03
 2.0238614381711E-03
 1.5333317107604E-03
 3.0303710521033E-03
 1.39685895499922E-03
 8.2153582153561E-03
 8.1808328817399E-03

 182.966666666664
 117.775193798402
 188.536697247699
 123.035211267685
 72.8225806451709
 45.2666666666267
 35.2637362637369
 24.2333333333369
 39.4489795918436
 70.077844311405
 117.060606060511
 107.02479338847
 143.03287671241
 72.1910112359662
 65.7182320441968
 43.746031746042
 112.874074074115
 70.1216216215937
 316.577023498727
 346.839490445933

 5.041648399821E-04
 3.649756129932E-04
 1.0441735118044E-03
 6.806972124886E-04
 9.61374124081E-05
 1.818810134411E-04
 .00024024024024
 8.10898475511E-05
 .000194805194805
 3.446196260877E-04
 4.315976110492E-04
 .000309023485785
 .000628027220248
 3.476934465392E-04
 6.373486297001E-04
 6.571421617547E-04
 5.162854385063E-04
 3.209000302028E-04
 .001072501072501
 .000917086998207

 34.1304347826033
 20.8787878787879
 93.6857142857138
 55.2950819672134
 9
 8.20000000000044
 18
 6
 18
 26.5625000000017
 33.2162162162204
 31
 46.9178082191985
 24.0322580645153
 51.3157894736808
 52.0370370370413
 44.0434782608668
 30.2352941176537
 61.5200000000116
 61.8181818181874

 5.041648399821E-04
 3.649756129932E-04
 1.0441735118044E-03
 6.806972124886E-04
 9.61374124081E-05
 1.818810134411E-04
 .00024024024024
 8.10898475511E-05
 .000194805194805
 3.446196260877E-04
 4.315976110492E-04
 .000309023485785
 .000628027220248
 3.476934465392E-04
 6.373486297001E-04
 6.571421617547E-04
 5.162854385063E-04
 3.209000302028E-04
 .001072501072501
 .000917086998207

 34.1304347826033
 20.8787878787879
 93.6857142857138
 55.2950819672134
 9
 8.20000000000044
 18
 6
 18
 26.5625000000017
 33.2162162162204
 31
 46.9178082191985
 24.0322580645153
 51.3157894736808
 52.0370370370413
 44.0434782608668
 30.2352941176537
 61.5200000000116
 61.8181818181874

 .003441473038141
 2.4884700885854E-03
 3.2916326895925E-03
 2.4884504653338E-03
 .001228422491881
 .001182226587363
 .000974307640974
 7.298086279598E-04
 8.658008658015E-04
 .001453864047559
 .002647909667782
 2.10335340324845E-03
 2.5121088809963E-03
 .001648739891654
 .001386512808471
 8.761895490057E-04
 .002514085613597
 1.07595892479642E-03
 7.1428571428551E-03
 7.2637458835329E-03

 204.770700636926
 131.986666666638
 218.625377643498
 141.565022421577
 77.8173913043594
 50.9692307692046
 39.5205479452034
 26.2592592592635
 44.2750000000001
 80.3925925926157
 130.726872246618
 118.194312796243
 167.061643835672
 82.3469387755216
 72.338709677415
 37.5277777777886
 127.008928571472
 82.0175438596241
 354.873873873918
 382.824964132041

 .003430512932924
 .002477410221525
 .00327174367032
 .002410337670458
 .001217740557169
 .001182226587363
 .000974307640974
 .000716293653368
 .000854978354979
 .001453864047559
 .002542926465094
 .002093384903707
 .002494902655784
 .001648739891654
 .001375331253564
 .000864020249714
 .002514085613597
 .001066520688614
 .00708923208923
 .00724290299721

 205.421725239599
 132.571428571401
 219.942249240118
 145.925925925971
 78.4912280701865
 50.9692307692046
 39.5205479452034
 26.7358490566064
 44.8227848101257
 80.3925925926157
 135.752293577931
 118.752380952415
 168.200000000056
 82.3469387755216
 72.9186991869862
 38.0422535211357
 127.008928571472
 82.7345132743107
 357.520423600653
 383.920863309399

 .000010960105217
 1.10598670604E-05
 1.98890192725E-05
 7.81127948758E-05
 .000010681934712
 0
 0
 1.35149745918E-05
 1.08225108225E-05
 0
 .000104983202688
 9.96849954145E-06
 1.72062252123E-05
 0
 .000011181554907
 1.21692992917E-05
 0
 9.43823618242E-06
 5.36250536251E-05
 2.08428863229E-05

 1
 1
 2
 7
 1
 1
 1
 1
 1
 1
 9
 1
 2
 2
 1
 1
 1
 1
 5
 2

 .000591845681718
 3.539157459322E-04
 4.3755842399363E-04
 3.905639743791E-04
 .000117501281832
 2.000691147855E-04
 5.33867200533E-05
 .000081089847551
 .000162337662338
 .000376927716034
 2.099664053751E-04
 2.093384903704E-04
 3.0282956373653E-03
 4.149889523216E-04
 1.229971039771E-04
 2.43385985834E-05
 .000426496666592
 .000339776502567
 .000933075933076
 .001135937304598

 19.9259259259259
 11.062499999999
 17.3181818181792
 18.8857142857104
 9.18181818181818
 4.45454545454855
 1.50000000000094
 3.00000000000247
 11.5333333333378
 19.9142857142798
 6
 7.09523809523812
 334.346590909098
 18.8378378378453
 7.72727272726807
 1
 30.8421052631616
 24.7222222222168
 43.9655172413836
 58.9082568807212

 .00010960105217
 .000121658537664
 6.961156745363E-05
 2.23179413931E-05
 0
 3.63762026882E-05
 1.33466800133E-05
 1.35149745918E-05
 0
 0
 6.99888017917E-05
 5.98109972487E-05
 3.44124504246E-05
 6.72955057818E-05

 10
 11
 5.28571428571449
 2
 2
 2
 1
 1
 1
 1
 6
 6
 4
 6

 .00010960105217
 .000121658537664
 6.961156745363E-05
 2.23179413931E-05
 0
 3.63762026882E-05
 1.33466800133E-05
 1.35149745918E-05
 0
 0
 6.99888017917E-05
 5.98109972487E-05
 3.44124504246E-05
 6.72955057818E-05

 10
 11
 5.28571428571449
 2
 2
 2
 1
 1
 1
 1
 6
 6
 4
 6

 .000263042525208
 .000143778271785
 .000139223134907
 .000100430736269
 .00010681934712
 5.45643040323E-05
 1.33466800133E-05
 1.35149745918E-05
 .000021645021645
 .000118462996468
 6.99888017917E-05
 6.97794967901E-05
 .00295086762391
 .000280397940758
 2.23631098141E-05
 1.21692992917E-05
 .000044894385957
 .000066067653277
 .000514800514801
 .000406436283297

 24
 13
 14
 9
 10
 3
 1
 1
 2
 11
 6
 7
 343
 25
 2
 1
 4
 7
 48
 39

 .000263042525208
 .000143778271785
 .000139223134907
 .000100430736269
 .00010681934712
 5.45643040323E-05
 1.33466800133E-05
 1.35149745918E-05
 .000021645021645
 .000118462996468
 6.99888017917E-05
 6.97794967901E-05
 .00295086762391
 .000280397940758
 2.23631098141E-05
 1.21692992917E-05
 .000044894385957
 .000066067653277
 .000514800514801
 .000406436283297

 24
 13
 14
 9
 10
 3
 1
 1
 2
 11
 6
 7
 343
 25
 2
 1
 4
 7
 48
 39

 .00021920210434
 8.84789364832E-05
 .000228723721633
 .000267815296717
 .000010681934712
 .000109128608065
 2.66933600267E-05
 5.40598983674E-05
 .000140692640693
 .000258464719566
 6.99888017917E-05
 7.97479963316E-05
 4.30155630307E-05
 6.72955057818E-05
 .000100633994163
 1.21692992917E-05
 .000381602280635
 .00027370884929
 .000418275418275
 .000729501021301

 20
 8
 23
 24
 1
 6
 2
 4
 13
 24
 6
 8
 5
 6
 9
 1
 34
 29
 39
 70

 .00021920210434
 8.84789364832E-05
 .000228723721633
 .000267815296717
 .000010681934712
 .000109128608065
 2.66933600267E-05
 5.40598983674E-05
 .000140692640693
 .000258464719566
 6.99888017917E-05
 7.97479963316E-05
 4.30155630307E-05
 6.72955057818E-05
 .000100633994163
 1.21692992917E-05
 .000381602280635
 .00027370884929
 .000418275418275
 .000729501021301

 20
 8
 23
 24
 1
 6
 2
 4
 13
 24
 6
 8
 5
 6
 9
 1
 34
 29
 39
 70

 .000131521262604
 8.84789364832E-05
 1.889456830884E-04
 1.004307362688E-04
 .000010681934712
 0
 0
 1.35149745918E-05
 1.08225108225E-05
 4.30774532609E-05
 2.449608062707E-04
 1.096534949559E-04
 1.462529143044E-04
 .000112159176303
 2.236310981402E-04
 1.825394893757E-04
 4.48943859571E-05
 2.831470854726E-05
 .00027885027885
 7.29501021301E-05

 10.1666666666667
 6.25
 7.84210526315681
 3.00000000000199
 1
 1
 1
 1
 1
 2.50000000000116
 11.6666666666635
 4.09090909090868
 4.0588235294108
 4.2
 10.4999999999928
 7.53333333332782
 2.49999999999889
 1
 20.6923076923077
 4.14285714285656

 .000010960105217
 1.10598670604E-05
 1.093896059986E-04
 4.46358827861E-05
 0
 0
 0
 0
 1.08225108225E-05
 1.07693633152E-05
 6.99888017917E-05
 5.98109972487E-05
 .000060221788243
 1.12159176303E-05
 4.47262196281E-05
 2.43385985835E-05
 1.12235964893E-05
 1.887647236484E-05
 .000032175032175
 2.08428863229E-05

 1
 1
 7.72727272727098
 2.50000000000112
 2.50000000000112
 2.50000000000112
 2.50000000000112
 2.50000000000112
 1
 1
 3.33333333333429
 3.33333333333333
 3.57142857142905
 1
 2.50000000000112
 2
 1
 1
 3
 2

 .000010960105217
 1.10598670604E-05
 8.95005867261E-05
 3.34769120896E-05
 0
 0
 0
 0
 1.08225108225E-05
 1.07693633152E-05
 4.66592011945E-05
 3.98739981658E-05
 3.44124504246E-05
 1.12159176303E-05
 3.35446647211E-05
 2.43385985835E-05
 1.12235964893E-05
 9.43823618242E-06
 .000032175032175
 2.08428863229E-05

 1
 1
 9
 3
 3
 3
 3
 3
 1
 1
 4
 4
 4
 1
 3
 2
 1
 1
 3
 2

 0
 0
 1.98890192725E-05
 1.11589706965E-05
 0
 0
 0
 0
 0
 0
 2.33296005972E-05
 1.99369990829E-05
 2.58093378184E-05
 0
 .000011181554907
 0
 0
 9.43823618242E-06

 0
 0
 2
 1
 1
 1
 1
 1
 1
 1
 2
 2
 3
 3
 1
 1
 1
 1

 .000120561157387
 7.74190694228E-05
 7.95560770898E-05
 4.46358827862E-05
 .000010681934712
 0
 0
 1.35149745918E-05
 0
 3.23080899457E-05
 .000174972004479
 4.98424977072E-05
 1.72062252123E-05
 6.72955057818E-05
 0
 3.65078978752E-05
 3.36707894678E-05
 9.43823618242E-06
 .000246675246675
 5.21072158072E-05

 11
 7
 8
 4
 1
 1
 1
 1
 1
 3
 15
 5
 2
 6
 6
 3
 3
 1
 23
 5

 .000120561157387
 7.74190694228E-05
 7.95560770898E-05
 4.46358827862E-05
 .000010681934712
 0
 0
 1.35149745918E-05
 0
 3.23080899457E-05
 .000174972004479
 4.98424977072E-05
 1.72062252123E-05
 6.72955057818E-05
 0
 3.65078978752E-05
 3.36707894678E-05
 9.43823618242E-06
 .000246675246675
 5.21072158072E-05

 11
 7
 8
 4
 1
 1
 1
 1
 1
 3
 15
 5
 2
 6
 6
 3
 3
 1
 23
 5

 0
 0
 0
 1.11589706965E-05
 0
 0
 0
 0
 0
 0
 0
 0
 1.72062252123E-05
 1.12159176303E-05
 .000156541768698
 .000121692992917

 0
 0
 0
 1
 1
 1
 1
 1
 1
 1
 1
 1
 2
 1
 14
 10

 0
 0
 0
 1.11589706965E-05
 0
 0
 0
 0
 0
 0
 0
 0
 1.72062252123E-05
 1.12159176303E-05
 .000156541768698
 .000121692992917

 0
 0
 0
 1
 1
 1
 1
 1
 1
 1
 1
 1
 2
 1
 14
 10

 0
 0
 0
 0
 0
 0
 0
 0
 0
 0
 0
 0
 5.16186756368E-05
 2.24318352606E-05
 2.23631098141E-05

 0
 0
 0
 0
 0
 0
 0
 0
 0
 0
 0
 0
 6
 2
 2

 0
 0
 0
 0
 0
 0
 0
 0
 0
 0
 0
 0
 5.16186756368E-05
 2.24318352606E-05
 2.23631098141E-05

 0
 0
 0
 0
 0
 0
 0
 0
 0
 0
 0
 0
 6
 2
 2

 5.48005260851E-05
 2.21197341208E-05
 0
 0
 6.40916082721E-05
 7.27524053764E-05
 2.66933600267E-05
 .000108119796735
 0
 0
 4.66592011945E-05
 1.99369990829E-05
 8.60311260614E-06
 0
 0
 0
 0
 0
 .0001287001287
 7.29501021301E-05

 5
 2
 2
 2
 6
 4
 2
 8
 8
 8
 4
 2
 1
 1
 1
 1
 1
 1
 12
 7

 5.48005260851E-05
 2.21197341208E-05
 0
 0
 6.40916082721E-05
 7.27524053764E-05
 2.66933600267E-05
 .000108119796735
 0
 0
 4.66592011945E-05
 1.99369990829E-05
 8.60311260614E-06
 0
 0
 0
 0
 0
 .0001287001287
 7.29501021301E-05

 5
 2
 2
 2
 6
 4
 2
 8
 8
 8
 4
 2
 1
 1
 1
 1
 1
 1
 12
 7

 5.48005260851E-05
 2.21197341208E-05
 0
 0
 6.40916082721E-05
 7.27524053764E-05
 2.66933600267E-05
 .000108119796735
 0
 0
 4.66592011945E-05
 1.99369990829E-05
 8.60311260614E-06
 0
 0
 0
 0
 0
 .0001287001287
 7.29501021301E-05

 5
 2
 2
 2
 6
 4
 2
 8
 8
 8
 4
 2
 1
 1
 1
 1
 1
 1
 12
 7

 0
 0
 6.96115674536E-05
 1.11589706965E-05
 0
 0
 0
 0
 0
 0
 0
 0
 0
 0
 .000201267988326
 .000255555285127
 5.61179824463E-05
 1.88764723648E-05
 .000010725010725
 1.04214431614E-05

 0
 0
 7
 1
 1
 1
 1
 1
 1
 1
 1
 1
 1
 1
 18
 21
 5
 2
 1
 1

 0
 0
 6.96115674536E-05
 1.11589706965E-05
 0
 0
 0
 0
 0
 0
 0
 0
 0
 0
 .000201267988326
 .000255555285127
 5.61179824463E-05
 1.88764723648E-05
 .000010725010725
 1.04214431614E-05

 0
 0
 7
 1
 1
 1
 1
 1
 1
 1
 1
 1
 1
 1
 18
 21
 5
 2
 1
 1

 0
 0
 6.96115674536E-05
 1.11589706965E-05
 0
 0
 0
 0
 0
 0
 0
 0
 0
 0
 .000201267988326
 .000255555285127
 5.61179824463E-05
 1.88764723648E-05
 .000010725010725
 1.04214431614E-05

 0
 0
 7
 1
 1
 1
 1
 1
 1
 1
 1
 1
 1
 1
 18
 21
 5
 2
 1
 1

 8.76808417361E-05
 .000055299335302

 8
 5

 8.76808417361E-05
 .000055299335302

 8
 5

 8.76808417361E-05
 .000055299335302

 8
 5

 8.76808417361E-05
 .000055299335302

 8
 5

 .000010960105217
 2.21197341208E-05
 0
 0
 .000021363869424
 5.45643040323E-05
 0
 1.35149745918E-05
 9.74025974026E-05
 7.53855432067E-05
 2.33296005972E-05
 0
 0
 0
 0
 0
 3.36707894678E-05
 1.88764723648E-05
 0
 1.04214431614E-05

 1
 2
 2
 2
 2
 3
 3
 1
 9
 7
 2
 2
 2
 2
 2
 2
 3
 2
 2
 1

 .000010960105217
 2.21197341208E-05
 0
 0
 .000021363869424
 5.45643040323E-05
 0
 1.35149745918E-05
 9.74025974026E-05
 7.53855432067E-05
 2.33296005972E-05
 0
 0
 0
 0
 0
 3.36707894678E-05
 1.88764723648E-05
 0
 1.04214431614E-05

 1
 2
 2
 2
 2
 3
 3
 1
 9
 7
 2
 2
 2
 2
 2
 2
 3
 2
 2
 1

 .000010960105217
 2.21197341208E-05
 0
 0
 .000021363869424
 5.45643040323E-05
 0
 1.35149745918E-05
 9.74025974026E-05
 7.53855432067E-05
 2.33296005972E-05
 0
 0
 0
 0
 0
 3.36707894678E-05
 1.88764723648E-05
 0
 1.04214431614E-05

 1
 2
 2
 2
 2
 3
 3
 1
 9
 7
 2
 2
 2
 2
 2
 2
 3
 2
 2
 1

 .000010960105217
 2.21197341208E-05
 0
 0
 .000021363869424
 5.45643040323E-05
 0
 1.35149745918E-05
 9.74025974026E-05
 7.53855432067E-05
 2.33296005972E-05
 0
 0
 0
 0
 0
 3.36707894678E-05
 1.88764723648E-05
 0
 1.04214431614E-05

 1
 2
 2
 2
 2
 3
 3
 1
 9
 7
 2
 2
 2
 2
 2
 2
 3
 2
 2
 1

 .00133713283648
 .00249952995565
 .0018397342827
 .00196397884259
 .000491368996753
 .000381950128226
 .000373707040374
 .000486539085307
 .00146103896104
 .00158309640734
 .00158641284061
 .00142549543443
 .00454244345604
 .00536120862728
 .00219158476178
 .00172804049943
 .000168353947339
 .000198202959831
 .0016731016731
 .00205302430281

 122
 226
 185
 176
 46
 21
 28
 36
 135
 147
 136
 143
 528
 478
 196
 142
 15
 21
 156
 197

 .00133713283648
 .00249952995565
 .0018397342827
 .00196397884259
 .000491368996753
 .000381950128226
 .000373707040374
 .000486539085307
 .00146103896104
 .00158309640734
 .00158641284061
 .00142549543443
 .00454244345604
 .00536120862728
 .00219158476178
 .00172804049943
 .000168353947339
 .000198202959831
 .0016731016731
 .00205302430281

 122
 226
 185
 176
 46
 21
 28
 36
 135
 147
 136
 143
 528
 478
 196
 142
 15
 21
 156
 197

 .00133713283648
 .00249952995565
 .0018397342827
 .00196397884259
 .000491368996753
 .000381950128226
 .000373707040374
 .000486539085307
 .00146103896104
 .00158309640734
 .00158641284061
 .00142549543443
 .00454244345604
 .00536120862728
 .00219158476178
 .00172804049943
 .000168353947339
 .000198202959831
 .0016731016731
 .00205302430281

 122
 226
 185
 176
 46
 21
 28
 36
 135
 147
 136
 143
 528
 478
 196
 142
 15
 21
 156
 197

 .00133713283648
 .00249952995565
 .0018397342827
 .00196397884259
 .000491368996753
 .000381950128226
 .000373707040374
 .000486539085307
 .00146103896104
 .00158309640734
 .00158641284061
 .00142549543443
 .00454244345604
 .00536120862728
 .00219158476178
 .00172804049943
 .000168353947339
 .000198202959831
 .0016731016731
 .00205302430281

 122
 226
 185
 176
 46
 21
 28
 36
 135
 147
 136
 143
 528
 478
 196
 142
 15
 21
 156
 197

 .00133713283648
 .00249952995565
 .0018397342827
 .00196397884259
 .000491368996753
 .000381950128226
 .000373707040374
 .000486539085307
 .00146103896104
 .00158309640734
 .00158641284061
 .00142549543443
 .00454244345604
 .00536120862728
 .00219158476178
 .00172804049943
 .000168353947339
 .000198202959831
 .0016731016731
 .00205302430281

 122
 226
 185
 176
 46
 21
 28
 36
 135
 147
 136
 143
 528
 478
 196
 142
 15
 21
 156
 197

 .00486628671635
 .00432440802062
 .00129278625271
 .00169616354587
 .00303366945821
 .00210981975592
 .00224224224224
 .00329765380041
 .00268398268398
 .00415697423968
 .00174972004479
 .00162486542526
 .00288204272306
 .00215345618502
 .00119642637505
 .000717988658213
 .000269366315742
 .000254832376925
 .00106177606178
 .00135478761099

 444
 391
 130
 152
 284
 116
 168
 244
 248
 386
 150
 163
 335
 192
 107
 59
 24
 27
 99
 130

 .00486628671635
 .00432440802062
 .00129278625271
 .00169616354587
 .00303366945821
 .00210981975592
 .00224224224224
 .00329765380041
 .00268398268398
 .00415697423968
 .00174972004479
 .00162486542526
 .00288204272306
 .00215345618502
 .00119642637505
 .000717988658213
 .000269366315742
 .000254832376925
 .00106177606178
 .00135478761099

 444
 391
 130
 152
 284
 116
 168
 244
 248
 386
 150
 163
 335
 192
 107
 59
 24
 27
 99
 130

 .00486628671635
 .00432440802062
 .00129278625271
 .00169616354587
 .00303366945821
 .00210981975592
 .00224224224224
 .00329765380041
 .00268398268398
 .00415697423968
 .00174972004479
 .00162486542526
 .00288204272306
 .00215345618502
 .00119642637505
 .000717988658213
 .000269366315742
 .000254832376925
 .00106177606178
 .00135478761099

 444
 391
 130
 152
 284
 116
 168
 244
 248
 386
 150
 163
 335
 192
 107
 59
 24
 27
 99
 130

 .00486628671635
 .00432440802062
 .00129278625271
 .00169616354587
 .00303366945821
 .00210981975592
 .00224224224224
 .00329765380041
 .00268398268398
 .00415697423968
 .00174972004479
 .00162486542526
 .00288204272306
 .00215345618502
 .00119642637505
 .000717988658213
 .000269366315742
 .000254832376925
 .00106177606178
 .00135478761099

 444
 391
 130
 152
 284
 116
 168
 244
 248
 386
 150
 163
 335
 192
 107
 59
 24
 27
 99
 130

 .00486628671635
 .00432440802062
 .00129278625271
 .00169616354587
 .00303366945821
 .00210981975592
 .00224224224224
 .00329765380041
 .00268398268398
 .00415697423968
 .00174972004479
 .00162486542526
 .00288204272306
 .00215345618502
 .00119642637505
 .000717988658213
 .000269366315742
 .000254832376925
 .00106177606178
 .00135478761099

 444
 391
 130
 152
 284
 116
 168
 244
 248
 386
 150
 163
 335
 192
 107
 59
 24
 27
 99
 130

 .000164401578255
 .000176957872966
 0
 0
 .000021363869424
 1.81881013441E-05
 0
 4.05449237755E-05
 1.08225108225E-05
 8.61549065219E-05
 1.16648002986E-05

 15
 16
 16
 16
 2
 1
 1
 3
 1
 8
 1

 .000164401578255
 .000176957872966
 0
 0
 .000021363869424
 1.81881013441E-05
 0
 4.05449237755E-05
 1.08225108225E-05
 8.61549065219E-05
 1.16648002986E-05

 15
 16
 16
 16
 2
 1
 1
 3
 1
 8
 1

 .000164401578255
 .000176957872966
 0
 0
 .000021363869424
 1.81881013441E-05
 0
 4.05449237755E-05
 1.08225108225E-05
 8.61549065219E-05
 1.16648002986E-05

 15
 16
 16
 16
 2
 1
 1
 3
 1
 8
 1

 .000164401578255
 .000176957872966
 0
 0
 .000021363869424
 1.81881013441E-05
 0
 4.05449237755E-05
 1.08225108225E-05
 8.61549065219E-05
 1.16648002986E-05

 15
 16
 16
 16
 2
 1
 1
 3
 1
 8
 1

 .000164401578255
 .000176957872966

 15
 16

 0
 0
 0
 0
 .000021363869424
 1.81881013441E-05
 0
 4.05449237755E-05
 1.08225108225E-05
 8.61549065219E-05
 1.16648002986E-05

 0
 0
 0
 0
 2
 1
 1
 3
 1
 8
 1

 .000010960105217
 4.42394682416E-05
 0
 0
 .000224320628952
 .000291009621506
 6.67334000667E-05
 9.46048221429E-05
 .00025974025974
 .000236925992935
 2.33296005972E-05
 0
 8.60311260614E-06
 1.12159176303E-05

 1
 4
 4
 4
 21
 16
 5
 7
 24
 22
 2
 2
 1
 1

 .000010960105217
 4.42394682416E-05
 0
 0
 .000224320628952
 .000291009621506
 6.67334000667E-05
 9.46048221429E-05
 .00025974025974
 .000236925992935
 2.33296005972E-05
 0
 8.60311260614E-06
 1.12159176303E-05

 1
 4
 4
 4
 21
 16
 5
 7
 24
 22
 2
 2
 1
 1

 .000010960105217
 4.42394682416E-05
 0
 0
 .000224320628952
 .000291009621506
 6.67334000667E-05
 9.46048221429E-05
 .00025974025974
 .000236925992935
 2.33296005972E-05
 0
 8.60311260614E-06
 1.12159176303E-05

 1
 4
 4
 4
 21
 16
 5
 7
 24
 22
 2
 2
 1
 1

 .000010960105217
 4.42394682416E-05
 0
 0
 .000224320628952
 .000291009621506
 6.67334000667E-05
 9.46048221429E-05
 .00025974025974
 .000236925992935
 2.33296005972E-05
 0
 8.60311260614E-06
 1.12159176303E-05

 1
 4
 4
 4
 21
 16
 5
 7
 24
 22
 2
 2
 1
 1

 .000010960105217
 4.42394682416E-05
 0
 0
 .000224320628952
 .000291009621506
 6.67334000667E-05
 9.46048221429E-05
 .00025974025974
 .000236925992935
 2.33296005972E-05
 0
 8.60311260614E-06
 1.12159176303E-05

 1
 4
 4
 4
 21
 16
 5
 7
 24
 22
 2
 2
 1
 1

 2.11858833844791E-02
 1.55944125551652E-02
 .031185982219222
 .032294061195823
 .01529653050757
 1.38411451228525E-02
 1.40407073740444E-02
 1.49610768732009E-02
 .010573593073591
 1.59386577065643E-02
 2.41461366181629E-02
 2.29375174448468E-02
 2.57491160301737E-02
 1.97175831940881E-02
 .030726912884504
 2.26227273833209E-02
 2.48153718377353E-02
 3.49025974025941E-02
 .034277134277166
 3.48284630455954E-02

 659.443869632729
 513.761702127687
 1541.52806122487
 1545.17346233635
 735.416201116835
 381.862023653098
 504.317490494357
 572.943992773736
 473.305015353007
 699.204054054285
 862.650241546369
 1062.10299869538
 1321.16906114259
 810.277588168639
 1294.4650655021
 954.439483592841
 1039.90818634119
 2744.11681990219
 1357.70713391779
 1489.85457809772

 .01023673827269
 8.7925943130216E-03
 2.33298196066062E-02
 2.48956636240165E-02
 .010681934711981
 9.4578126989281E-03
 9.1691691691737E-03
 1.07308898259578E-02
 7.1212121212075E-03
 1.02739726027502E-02
 .01500093318405
 1.54312372901315E-02
 .014900591033833
 1.27188505927819E-02
 .021982936947207
 1.67449558254052E-02
 .01863117017216
 .03146707943219
 .02358429858433
 2.47821918379629E-02

 643.50963597438
 616.187421383625
 1927.68883205458
 1918.45316001804
 922.977999999848
 483.319230769213
 622.179039301331
 714.340050377929
 613.541033434658
 909.062893081813
 1089.80559875615
 1357.31136950854
 1537.47459584289
 998.777777777862
 1651.92166836223
 1196.98982558138
 1309.71204819286
 3029.35692861402
 1781.54297407919
 1930.14802354945

 .00183033757124
 .00108386697192
 .00229718172597
 .00187470707702
 .000395231584345
 .000327385824194
 .000427093760427
 .000554113958266
 .000238095238095
 .000236925992935
 .00124813363195
 .00100681845369
 .00082589881019
 .000773898316491
 .0019120458891
 .00113174483413
 .00223349570136
 .00151011778919
 .00245602745603
 .00258451790404

 167
 98
 231
 168
 37
 18
 32
 41
 22
 22
 107
 101
 96
 69
 171
 93
 199
 160
 229
 248

 .00183033757124
 .00108386697192
 .00229718172597
 .00187470707702
 .000395231584345
 .000327385824194
 .000427093760427
 .000554113958266
 .000238095238095
 .000236925992935
 .00124813363195
 .00100681845369
 .00082589881019
 .000773898316491
 .0019120458891
 .00113174483413
 .00223349570136
 .00151011778919
 .00245602745603
 .00258451790404

 167
 98
 231
 168
 37
 18
 32
 41
 22
 22
 107
 101
 96
 69
 171
 93
 199
 160
 229
 248

 .00183033757124
 .00108386697192
 .00229718172597
 .00187470707702
 .000395231584345
 .000327385824194
 .000427093760427
 .000554113958266
 .000238095238095
 .000236925992935
 .00124813363195
 .00100681845369
 .00082589881019
 .000773898316491
 .0019120458891
 .00113174483413
 .00223349570136
 .00151011778919
 .00245602745603
 .00258451790404

 167
 98
 231
 168
 37
 18
 32
 41
 22
 22
 107
 101
 96
 69
 171
 93
 199
 160
 229
 248

 .00010960105217
 4.42394682416E-05
 9.94450963623E-06
 1.11589706965E-05
 .000032045804136
 1.81881013441E-05
 2.66933600267E-05
 1.35149745918E-05
 1.08225108225E-05
 1.07693633152E-05
 0
 9.96849954145E-06
 .000060221788243
 3.36477528909E-05
 .000011181554907
 3.65078978752E-05
 0
 0
 .0000429000429
 2.08428863229E-05

 10
 4
 1
 1
 3
 1
 2
 1
 1
 1
 1
 1
 7
 3
 1
 3
 3
 3
 4
 2

 .00010960105217
 4.42394682416E-05
 9.94450963623E-06
 1.11589706965E-05
 .000032045804136
 1.81881013441E-05
 2.66933600267E-05
 1.35149745918E-05
 1.08225108225E-05
 1.07693633152E-05
 0
 9.96849954145E-06
 .000060221788243
 3.36477528909E-05
 .000011181554907
 3.65078978752E-05
 0
 0
 .0000429000429
 2.08428863229E-05

 10
 4
 1
 1
 3
 1
 2
 1
 1
 1
 1
 1
 7
 3
 1
 3
 3
 3
 4
 2

 .00010960105217
 4.42394682416E-05
 9.94450963623E-06
 1.11589706965E-05
 .000032045804136
 1.81881013441E-05
 2.66933600267E-05
 1.35149745918E-05
 1.08225108225E-05
 1.07693633152E-05
 0
 9.96849954145E-06
 .000060221788243
 3.36477528909E-05
 .000011181554907
 3.65078978752E-05
 0
 0
 .0000429000429
 2.08428863229E-05

 10
 4
 1
 1
 3
 1
 2
 1
 1
 1
 1
 1
 7
 3
 1
 3
 3
 3
 4
 2

 .00829679964928
 .00766448787286
 .021022693371
 .0230097975763
 .0102546573235
 .00911223877339
 .00871538204872
 .0101632608931
 .00687229437229
 .0100262772465
 .0137527995521
 .0144144503369
 .0140144704354
 .0119113045234
 .0200597095032
 .0155767030934
 .0163976744708
 .029956961643
 .0210853710854
 .0221768310476

 757
 693
 2114
 2062
 960
 501
 653
 752
 635
 931
 1179
 1446
 1629
 1062
 1794
 1280
 1461
 3174
 1966
 2128

 .00829679964928
 .00766448787286
 .021022693371
 .0230097975763
 .0102546573235
 .00911223877339
 .00871538204872
 .0101632608931
 .00687229437229
 .0100262772465
 .0137527995521
 .0144144503369
 .0140144704354
 .0119113045234
 .0200597095032
 .0155767030934
 .0163976744708
 .029956961643
 .0210853710854
 .0221768310476

 757
 693
 2114
 2062
 960
 501
 653
 752
 635
 931
 1179
 1446
 1629
 1062
 1794
 1280
 1461
 3174
 1966
 2128

 .00829679964928
 .00766448787286
 .021022693371
 .0230097975763
 .0102546573235
 .00911223877339
 .00871538204872
 .0101632608931
 .00687229437229
 .0100262772465
 .0137527995521
 .0144144503369
 .0140144704354
 .0119113045234
 .0200597095032
 .0155767030934
 .0163976744708
 .029956961643
 .0210853710854
 .0221768310476

 757
 693
 2114
 2062
 960
 501
 653
 752
 635
 931
 1179
 1446
 1629
 1062
 1794
 1280
 1461
 3174
 1966
 2128

 1.09491451117891E-02
 6.8018182421436E-03
 7.85616261261573E-03
 7.3983975718065E-03
 .004614595795589
 4.3833324239244E-03
 4.8715382048707E-03
 4.2301870472431E-03
 3.4523809523835E-03
 5.6646851038141E-03
 9.1452034341129E-03
 7.5062801547153E-03
 1.08485249963407E-02
 6.9987326013062E-03
 .008743975937297
 5.8777715579157E-03
 6.1842016655753E-03
 3.4355179704041E-03
 .010692835692836
 1.00462712076325E-02

 674.341341341345
 381.357723577227
 394.77721518996
 289.084464555075
 301.245370370363
 162.950207468856
 282.479452054781
 214.258785942459
 184.040752351131
 318.585551330792
 490.045918367262
 455.220451527275
 1024.07057890552
 467.714743589705
 395.795396419508
 263.447204968922
 227.068965517231
 131.505494505527
 422.887662988873
 403.736514522776

 .009074967119681
 .005419334859594
 .0052705901072
 4.4970651907065E-03
 .003845496496324
 3.5830559647841E-03
 4.2976309642967E-03
 3.5544383176511E-03
 2.5865800865825E-03
 4.3508227793552E-03
 7.1621873833472E-03
 5.8016667331272E-03
 9.7731359205723E-03
 6.0565955203606E-03
 .005926224100727
 4.1862389563617E-03
 3.5915508765593E-03
 1.8027031108448E-03
 .00644573144573
 6.0757013631216E-03

 802.408212560394
 470.408163265303
 530
 401.00496277916
 356.022222222224
 195.010152284263
 318.024844720496
 251.273764258546
 237.008368200839
 402.004950495053
 610.013029315963
 572.085910652929
 1132.00704225352
 536.014814814814
 528.003773584908
 342.005813953492
 318.006249999995
 187.041884816761
 593.053244592349
 567.21955403087

 .009074967119681
 .005419334859594
 .0052705901072
 4.4970651907065E-03
 .003845496496324
 3.5830559647841E-03
 4.2976309642967E-03
 3.5544383176511E-03
 2.5865800865825E-03
 4.3508227793552E-03
 7.1621873833472E-03
 5.8016667331272E-03
 9.7731359205723E-03
 6.0565955203606E-03
 .005926224100727
 4.1862389563617E-03
 3.5915508765593E-03
 1.8027031108448E-03
 .00644573144573
 6.0757013631216E-03

 802.408212560394
 470.408163265303
 530
 401.00496277916
 356.022222222224
 195.010152284263
 318.024844720496
 251.273764258546
 237.008368200839
 402.004950495053
 610.013029315963
 572.085910652929
 1132.00704225352
 536.014814814814
 528.003773584908
 342.005813953492
 318.006249999995
 187.041884816761
 593.053244592349
 567.21955403087

 .009074967119681
 .005419334859594
 .0052705901072
 4.4970651907065E-03
 .003845496496324
 3.5830559647841E-03
 4.2976309642967E-03
 3.5544383176511E-03
 2.5865800865825E-03
 4.3508227793552E-03
 7.1621873833472E-03
 5.8016667331272E-03
 9.7731359205723E-03
 6.0565955203606E-03
 .005926224100727
 4.1862389563617E-03
 3.5915508765593E-03
 1.8027031108448E-03
 .00644573144573
 6.0757013631216E-03

 802.408212560394
 470.408163265303
 530
 401.00496277916
 356.022222222224
 195.010152284263
 318.024844720496
 251.273764258546
 237.008368200839
 402.004950495053
 610.013029315963
 572.085910652929
 1132.00704225352
 536.014814814814
 528.003773584908
 342.005813953492
 318.006249999995
 187.041884816761
 593.053244592349
 567.21955403087

 .00043840420868
 .000353915745933
 1.58117703215623E-03
 .00170732251657
 .000138865151256
 5.45643040323E-05
 .000106773440107
 .000108119796735
 .000151515151515
 .000258464719566
 4.199328107506E-04
 3.887714821169E-04
 2.2368092776014E-04
 2.131024349756E-04
 .00176668567531
 .00110740623555
 .000852993333184
 .000396405919662
 .00131917631918
 1.1672016340843E-03

 40
 32
 157.012578616349
 153
 9.61538461538462
 3
 8
 8
 14
 24
 34.0555555555578
 35.2051282051298
 24.0769230769251
 15.4210526315782
 158
 91
 76
 42
 119.065040650414
 106.160714285723

 .00043840420868
 .000353915745933
 .00157123252252
 .00170732251657
 .000117501281832
 5.45643040323E-05
 .000106773440107
 .000108119796735
 .000151515151515
 .000258464719566
 .000408268010452
 .000368834483034
 .000215077815154
 .000190670599715
 .00176668567531
 .00110740623555
 .000852993333184
 .000396405919662
 .00129772629773
 .0011359373046

 40
 32
 158
 153
 11
 3
 8
 8
 14
 24
 35
 37
 25
 17
 158
 91
 76
 42
 121
 109

 .00043840420868
 .000353915745933
 .00157123252252
 .00170732251657
 .000117501281832
 5.45643040323E-05
 .000106773440107
 .000108119796735
 .000151515151515
 .000258464719566
 .000408268010452
 .000368834483034
 .000215077815154
 .000190670599715
 .00176668567531
 .00110740623555
 .000852993333184
 .000396405919662
 .00129772629773
 .0011359373046

 40
 32
 158
 153
 11
 3
 8
 8
 14
 24
 35
 37
 25
 17
 158
 91
 76
 42
 121
 109

 0
 0
 9.94450963623E-06
 0
 .000021363869424
 0
 0
 0
 0
 0
 1.16648002986E-05
 1.99369990829E-05
 8.60311260614E-06
 2.24318352606E-05
 0
 0
 0
 0
 .00002145002145
 3.12643294843E-05

 0
 0
 1
 1
 2
 2
 2
 2
 2
 2
 1
 2
 1
 2
 2
 2
 2
 2
 2
 3

 0
 0
 9.94450963623E-06
 0
 .000021363869424
 0
 0
 0
 0
 0
 1.16648002986E-05
 1.99369990829E-05
 8.60311260614E-06
 2.24318352606E-05
 0
 0
 0
 0
 .00002145002145
 3.12643294843E-05

 0
 0
 1
 1
 2
 2
 2
 2
 2
 2
 1
 2
 1
 2
 2
 2
 2
 2
 2
 3

 .000865848312144
 .000453454549476
 .000725949203445
 .000836922802241
 .000405913519057
 .00040013822957
 .000186853520187
 .000229754568061
 .000270562770563
 .000409235805979
 .00067655841732
 .000498424977072
 .000464568080732
 .0003701252818
 .000659711739514
 .000401586876628
 .00147029114009
 .000915508909695
 .00233805233805
 .00217808162074

 79
 41
 73
 75
 38
 22
 14
 17
 25
 38
 58
 50
 54
 33
 59
 33
 131
 97
 218
 209

 .000865848312144
 .000453454549476
 .000725949203445
 .000836922802241
 .000405913519057
 .00040013822957
 .000186853520187
 .000229754568061
 .000270562770563
 .000409235805979
 .00067655841732
 .000498424977072
 .000464568080732
 .0003701252818
 .000659711739514
 .000401586876628
 .00147029114009
 .000915508909695
 .00233805233805
 .00217808162074

 79
 41
 73
 75
 38
 22
 14
 17
 25
 38
 58
 50
 54
 33
 59
 33
 131
 97
 218
 209

 .000865848312144
 .000453454549476
 .000725949203445
 .000836922802241
 .000405913519057
 .00040013822957
 .000186853520187
 .000229754568061
 .000270562770563
 .000409235805979
 .00067655841732
 .000498424977072
 .000464568080732
 .0003701252818
 .000659711739514
 .000401586876628
 .00147029114009
 .000915508909695
 .00233805233805
 .00217808162074

 79
 41
 73
 75
 38
 22
 14
 17
 25
 38
 58
 50
 54
 33
 59
 33
 131
 97
 218
 209

 5.699254712841E-04
 5.751130871406E-04
 2.784462698145E-04
 .000357087062289
 .000224320628952
 .000345573925538
 .00028028028028
 .000337874364796
 .000443722943723
 6.461617989139E-04
 8.865248226951E-04
 8.174169623992E-04
 3.8714006727628E-04
 .00035890936417
 .000391354421746
 .000182539489376
 .000269366315742
 3.209000302023E-04
 .000589875589876
 6.252865896866E-04

 27.5384615384578
 25.5769230769205
 24.2857142857112
 32
 19.0952380952381
 19
 21
 25
 30.317073170737
 52.4333333333356
 66.5526315789473
 72.609756097567
 41.1333333333332
 32
 35
 15
 17.3333333333333
 28.5294117647025
 55
 49.0333333333383

 .000504164839982
 .000530873618899
 .000258557250542
 .000357087062289
 .00021363869424
 .000345573925538
 .00028028028028
 .000337874364796
 4.112554112555E-04
 6.353924355987E-04
 8.398656215006E-04
 .000767574464692
 3.7853695467014E-04
 .00035890936417
 .000391354421746
 .000182539489376
 .000224471929785
 .000292585321655
 .000589875589876
 5.731793738794E-04

 30.3478260869565
 27.3749999999979
 26
 32
 20
 19
 21
 25
 32.4736842105294
 53.3050847457624
 70.0277777777794
 77
 42.0454545454545
 32
 35
 15
 20
 31
 55
 53.0363636363677

 .000504164839982
 .000530873618899
 .000258557250542
 .000357087062289
 .00021363869424
 .000345573925538
 .00028028028028
 .000337874364796
 4.112554112555E-04
 6.353924355987E-04
 8.398656215006E-04
 .000767574464692
 3.7853695467014E-04
 .00035890936417
 .000391354421746
 .000182539489376
 .000224471929785
 .000292585321655
 .000589875589876
 5.731793738794E-04

 30.3478260869565
 27.3749999999979
 26
 32
 20
 19
 21
 25
 32.4736842105294
 53.3050847457624
 70.0277777777794
 77
 42.0454545454545
 32
 35
 15
 20
 31
 55
 53.0363636363677

 6.57606313021E-05
 4.42394682416E-05
 1.98890192725E-05
 0
 .000010681934712
 0
 0
 0
 3.24675324675E-05
 1.07693633152E-05
 4.66592011945E-05
 4.98424977072E-05
 8.60311260614E-06
 0
 0
 0
 .000044894385957
 2.83147085473E-05
 0
 5.21072158072E-05

 6
 4
 2
 2
 1
 1
 1
 1
 3
 1
 4
 5
 1
 1
 1
 1
 4
 3
 3
 5

 6.57606313021E-05
 4.42394682416E-05
 1.98890192725E-05
 0
 .000010681934712
 0
 0
 0
 3.24675324675E-05
 1.07693633152E-05
 4.66592011945E-05
 4.98424977072E-05
 8.60311260614E-06
 0
 0
 0
 .000044894385957
 2.83147085473E-05
 0
 5.21072158072E-05

 6
 4
 2
 2
 1
 1
 1
 1
 3
 1
 4
 5
 1
 1
 1
 1
 4
 3
 3
 5

 1.9289785181941E-03
 2.4774102215332E-03
 3.4308558245031E-03
 3.4815988573181E-03
 3.5250384549651E-03
 3.8013131809192E-03
 .002082082082085
 2.2434857822484E-03
 2.6406926406939E-03
 .001927716033428
 2.7412280701778E-03
 2.9207703656492E-03
 1.29907000352744E-03
 .001256182774594
 1.7666856753106E-03
 .001983595784555
 4.7363577184709E-03
 5.8045152521903E-03
 4.5903045903022E-03
 4.9293426153674E-03

 61.6704545454623
 93.7321428572109
 103.515942028951
 108.993589743552
 104.945454545481
 60.2822966507347
 57.038461538481
 55.987951807263
 92.1803278688634
 88.8100558659338
 123.902127659595
 130.201365187811
 36.8013245033056
 32.2142857142734
 46.253164556959
 40.4969325153392
 261.255924170631
 373.764227642383
 125.158878504645
 145.909090909063

 .000043840420868
 9.95388035436E-05
 0
 0
 .00010681934712
 7.27524053764E-05
 6.67334000667E-05
 9.46048221429E-05
 8.65800865801E-05
 5.38468165762E-05
 1.16648002986E-05
 3.98739981658E-05
 8.60311260614E-06
 0
 .000011181554907
 1.21692992917E-05
 0
 0
 .0000429000429
 3.12643294843E-05

 4
 9
 9
 9
 10
 4
 5
 7
 8
 5
 1
 4
 1
 1
 1
 1
 1
 1
 4
 3

 .000043840420868
 9.95388035436E-05
 0
 0
 .00010681934712
 7.27524053764E-05
 6.67334000667E-05
 9.46048221429E-05
 8.65800865801E-05
 5.38468165762E-05
 1.16648002986E-05
 3.98739981658E-05
 8.60311260614E-06
 0
 .000011181554907
 1.21692992917E-05
 0
 0
 .0000429000429
 3.12643294843E-05

 4
 9
 9
 9
 10
 4
 5
 7
 8
 5
 1
 4
 1
 1
 1
 1
 1
 1
 4
 3

 .000043840420868
 9.95388035436E-05
 0
 0
 .00010681934712
 7.27524053764E-05
 6.67334000667E-05
 9.46048221429E-05
 8.65800865801E-05
 5.38468165762E-05
 1.16648002986E-05
 3.98739981658E-05
 8.60311260614E-06
 0
 .000011181554907
 1.21692992917E-05
 0
 0
 .0000429000429
 3.12643294843E-05

 4
 9
 9
 9
 10
 4
 5
 7
 8
 5
 1
 4
 1
 1
 1
 1
 1
 1
 4
 3

 .000043840420868
 9.95388035436E-05
 0
 0
 .00010681934712
 7.27524053764E-05
 6.67334000667E-05
 9.46048221429E-05
 8.65800865801E-05
 5.38468165762E-05
 1.16648002986E-05
 3.98739981658E-05
 8.60311260614E-06
 0
 .000011181554907
 1.21692992917E-05
 0
 0
 .0000429000429
 3.12643294843E-05

 4
 9
 9
 9
 10
 4
 5
 7
 8
 5
 1
 4
 1
 1
 1
 1
 1
 1
 4
 3

 .000043840420868
 9.95388035436E-05
 0
 0
 .00010681934712
 7.27524053764E-05
 6.67334000667E-05
 9.46048221429E-05
 8.65800865801E-05
 5.38468165762E-05
 1.16648002986E-05
 3.98739981658E-05
 8.60311260614E-06
 0
 .000011181554907
 1.21692992917E-05
 0
 0
 .0000429000429
 3.12643294843E-05

 4
 9
 9
 9
 10
 4
 5
 7
 8
 5
 1
 4
 1
 1
 1
 1
 1
 1
 4
 3

 5.48005260851E-05
 .000055299335302
 .000228723721634
 1.562255897512E-04
 7.47735429841E-05
 .000236445317473
 .00012012012012
 .00021623959347
 5.41125541126E-05
 3.23080899457E-05
 6.99888017917E-05
 .000089716495873
 4.30155630307E-05
 6.72955057818E-05
 1.229971039773E-04
 1.338622922092E-04
 2.581427192531E-04
 .000320900030202
 .000375375375375
 .000333486181166

 5
 5
 11.5217391304342
 8.2857142857088
 5.28571428571524
 13
 9
 16
 5
 3
 4.33333333333429
 9
 5
 6
 5.54545454545484
 5.54545454545475
 12.5652173913049
 17.5294117647015
 17.6285714285714
 17.5624999999944

 5.48005260851E-05
 .000055299335302
 .000109389605999
 .000111589706965
 6.40916082721E-05
 .000236445317473
 .00012012012012
 .00021623959347
 5.41125541126E-05
 3.23080899457E-05
 5.83240014931E-05
 .000089716495873
 4.30155630307E-05
 6.72955057818E-05
 5.59077745351E-05
 7.30157957505E-05
 8.97887719141E-05
 .000132135306554
 .000203775203775
 .00021885030639

 5
 5
 11
 10
 6
 13
 9
 16
 5
 3
 5
 9
 5
 6
 5
 6
 8
 14
 19
 21

 5.48005260851E-05
 .000055299335302
 .000109389605999
 .000111589706965
 6.40916082721E-05
 .000236445317473
 .00012012012012
 .00021623959347
 5.41125541126E-05
 3.23080899457E-05
 5.83240014931E-05
 .000089716495873
 4.30155630307E-05
 6.72955057818E-05
 5.59077745351E-05
 7.30157957505E-05
 8.97887719141E-05
 .000132135306554
 .000203775203775
 .00021885030639

 5
 5
 11
 10
 6
 13
 9
 16
 5
 3
 5
 9
 5
 6
 5
 6
 8
 14
 19
 21

 5.48005260851E-05
 .000055299335302
 .000109389605999
 .000111589706965
 6.40916082721E-05
 .000236445317473
 .00012012012012
 .00021623959347
 5.41125541126E-05
 3.23080899457E-05
 5.83240014931E-05
 .000089716495873
 4.30155630307E-05
 6.72955057818E-05
 5.59077745351E-05
 7.30157957505E-05
 8.97887719141E-05
 .000132135306554
 .000203775203775
 .00021885030639

 5
 5
 11
 10
 6
 13
 9
 16
 5
 3
 5
 9
 5
 6
 5
 6
 8
 14
 19
 21

 5.48005260851E-05
 .000055299335302
 .000109389605999
 .000111589706965
 6.40916082721E-05
 .000236445317473
 .00012012012012
 .00021623959347
 5.41125541126E-05
 3.23080899457E-05
 5.83240014931E-05
 .000089716495873
 4.30155630307E-05
 6.72955057818E-05
 5.59077745351E-05
 7.30157957505E-05
 8.97887719141E-05
 .000132135306554
 .000203775203775
 .00021885030639

 5
 5
 11
 10
 6
 13
 9
 16
 5
 3
 5
 9
 5
 6
 5
 6
 8
 14
 19
 21

 0
 0
 .000119334115635
 4.46358827862E-05
 .000010681934712
 0
 0
 0
 0
 0
 1.16648002986E-05
 0
 0
 0
 6.70893294422E-05
 6.08464964587E-05
 .000168353947339
 .000188764723648
 .0001716001716
 .000114635874776

 0
 0
 12
 4
 1
 1
 1
 1
 1
 1
 1
 1
 1
 1
 6
 5
 15
 20
 16
 11

 0
 0
 .000119334115635
 4.46358827862E-05
 .000010681934712
 0
 0
 0
 0
 0
 1.16648002986E-05
 0
 0
 0
 6.70893294422E-05
 6.08464964587E-05
 .000168353947339
 .000188764723648
 .0001716001716
 .000114635874776

 0
 0
 12
 4
 1
 1
 1
 1
 1
 1
 1
 1
 1
 1
 6
 5
 15
 20
 16
 11

 0
 0
 .000119334115635
 4.46358827862E-05
 .000010681934712
 0
 0
 0
 0
 0
 1.16648002986E-05
 0
 0
 0
 6.70893294422E-05
 6.08464964587E-05
 .000168353947339
 .000188764723648
 .0001716001716
 .000114635874776

 0
 0
 12
 4
 1
 1
 1
 1
 1
 1
 1
 1
 1
 1
 6
 5
 15
 20
 16
 11

 0
 0
 .000119334115635
 4.46358827862E-05
 .000010681934712
 0
 0
 0
 0
 0
 1.16648002986E-05
 0
 0
 0
 6.70893294422E-05
 6.08464964587E-05
 .000168353947339
 .000188764723648
 .0001716001716
 .000114635874776

 0
 0
 12
 4
 1
 1
 1
 1
 1
 1
 1
 1
 1
 1
 6
 5
 15
 20
 16
 11

 .000175361683472
 .00026543680945
 .001322619781622
 .001082420157565
 .001506152794393
 .001618741019626
 .000440440440441
 .000445994161531
 .000487012987013
 2.476953562502E-04
 2.682904068683E-04
 3.887714821166E-04
 .000524789868975
 3.476934465395E-04
 .000760345733678
 .000876189549006
 .000673415789356
 .000726744186046
 .001555126555127
 .001802909666933

 11.125
 12
 91.1654135338742
 67.6804123711247
 87.3829787233701
 56.910112359561
 18.3333333333333
 16.8787878787865
 24.3777777777825
 12.0434782608662
 13.2608695652191
 26.2820512820519
 31.491803278682
 22.6129032258084
 37.5588235294024
 40.6944444444519
 38.5333333333207
 52.844155844171
 74.6551724137903
 92.3526011560842

 .000142481367821
 .000132718404725
 .00106406253108
 .000881558685027
 .000384549649633
 .000381950128226
 .000293626960294
 .000256784517245
 .000313852813853
 .000150771086413
 .000186636804778
 .000309023485785
 .000215077815154
 5.60795881515E-05
 .000503169970816
 .000596295665296
 .000516285438506
 .00058517064331
 .000911625911626
 .0011359373046

 13
 12
 107
 79
 36
 21
 22
 19
 29
 14
 16
 31
 25
 5
 45
 49
 46
 62
 85
 109

 .000142481367821
 .000132718404725
 .00106406253108
 .000881558685027
 .000384549649633
 .000381950128226
 .000293626960294
 .000256784517245
 .000313852813853
 .000150771086413
 .000186636804778
 .000309023485785
 .000215077815154
 5.60795881515E-05
 .000503169970816
 .000596295665296
 .000516285438506
 .00058517064331
 .000911625911626
 .0011359373046

 13
 12
 107
 79
 36
 21
 22
 19
 29
 14
 16
 31
 25
 5
 45
 49
 46
 62
 85
 109

 .000142481367821
 .000132718404725
 .00106406253108
 .000881558685027
 .000384549649633
 .000381950128226
 .000293626960294
 .000256784517245
 .000313852813853
 .000150771086413
 .000186636804778
 .000309023485785
 .000215077815154
 5.60795881515E-05
 .000503169970816
 .000596295665296
 .000516285438506
 .00058517064331
 .000911625911626
 .0011359373046

 13
 12
 107
 79
 36
 21
 22
 19
 29
 14
 16
 31
 25
 5
 45
 49
 46
 62
 85
 109

 .000142481367821
 .000132718404725
 .00106406253108
 .000881558685027
 .000384549649633
 .000381950128226
 .000293626960294
 .000256784517245
 .000313852813853
 .000150771086413
 .000186636804778
 .000309023485785
 .000215077815154
 5.60795881515E-05
 .000503169970816
 .000596295665296
 .000516285438506
 .00058517064331
 .000911625911626
 .0011359373046

 13
 12
 107
 79
 36
 21
 22
 19
 29
 14
 16
 31
 25
 5
 45
 49
 46
 62
 85
 109

 .000032880315651
 .000132718404725
 .000258557250542
 .000200861472538
 .00112160314476
 .0012367908914
 .000146813480147
 .000189209644286
 .00017316017316
 9.69242698372E-05
 8.16536020903E-05
 7.97479963316E-05
 .000309712053821
 .000291613858388
 .000257175762862
 .00027989388371
 .00015713035085
 .000141573542736
 .000643500643501
 .000666972362333

 3
 12
 26
 18
 105
 68
 11
 14
 16
 9
 7
 8
 36
 26
 23
 23
 14
 15
 60
 64

 .000032880315651
 .000132718404725
 .000258557250542
 .000200861472538
 .00112160314476
 .0012367908914
 .000146813480147
 .000189209644286
 .00017316017316
 9.69242698372E-05
 8.16536020903E-05
 7.97479963316E-05
 .000309712053821
 .000291613858388
 .000257175762862
 .00027989388371
 .00015713035085
 .000141573542736
 .000643500643501
 .000666972362333

 3
 12
 26
 18
 105
 68
 11
 14
 16
 9
 7
 8
 36
 26
 23
 23
 14
 15
 60
 64

 .000032880315651
 .000132718404725
 .000258557250542
 .000200861472538
 .00112160314476
 .0012367908914
 .000146813480147
 .000189209644286
 .00017316017316
 9.69242698372E-05
 8.16536020903E-05
 7.97479963316E-05
 .000309712053821
 .000291613858388
 .000257175762862
 .00027989388371
 .00015713035085
 .000141573542736
 .000643500643501
 .000666972362333

 3
 12
 26
 18
 105
 68
 11
 14
 16
 9
 7
 8
 36
 26
 23
 23
 14
 15
 60
 64

 .000032880315651
 .000132718404725
 .000258557250542
 .000200861472538
 .00112160314476
 .0012367908914
 .000146813480147
 .000189209644286
 .00017316017316
 9.69242698372E-05
 8.16536020903E-05
 7.97479963316E-05
 .000309712053821
 .000291613858388
 .000257175762862
 .00027989388371
 .00015713035085
 .000141573542736
 .000643500643501
 .000666972362333

 3
 12
 26
 18
 105
 68
 11
 14
 16
 9
 7
 8
 36
 26
 23
 23
 14
 15
 60
 64

 .000043840420868
 .000176957872966
 .000198890192725
 .000178543531145
 .000138865151256
 .000181881013441
 .00004004004004
 1.35149745918E-05
 8.65800865801E-05
 .000043077453261
 .00025662560657
 .000269149487619
 8.60311260614E-05
 6.72955057818E-05
 0
 7.30157957505E-05
 7.85651754248E-05
 .000207641196013
 .000182325182325
 .000135478761099

 4
 16
 20
 16
 13
 10
 3
 1
 8
 4
 22
 27
 10
 6
 6
 6
 7
 22
 17
 13

 .000043840420868
 .000176957872966
 .000198890192725
 .000178543531145
 .000138865151256
 .000181881013441
 .00004004004004
 1.35149745918E-05
 8.65800865801E-05
 .000043077453261
 .00025662560657
 .000269149487619
 8.60311260614E-05
 6.72955057818E-05
 0
 7.30157957505E-05
 7.85651754248E-05
 .000207641196013
 .000182325182325
 .000135478761099

 4
 16
 20
 16
 13
 10
 3
 1
 8
 4
 22
 27
 10
 6
 6
 6
 7
 22
 17
 13

 .000043840420868
 .000176957872966
 .000198890192725
 .000178543531145
 .000138865151256
 .000181881013441
 .00004004004004
 1.35149745918E-05
 8.65800865801E-05
 .000043077453261
 .00025662560657
 .000269149487619
 8.60311260614E-05
 6.72955057818E-05
 0
 7.30157957505E-05
 7.85651754248E-05
 .000207641196013
 .000182325182325
 .000135478761099

 4
 16
 20
 16
 13
 10
 3
 1
 8
 4
 22
 27
 10
 6
 6
 6
 7
 22
 17
 13

 .000043840420868
 .000176957872966
 .000198890192725
 .000178543531145
 .000138865151256
 .000181881013441
 .00004004004004
 1.35149745918E-05
 8.65800865801E-05
 .000043077453261
 .00025662560657
 .000269149487619
 8.60311260614E-05
 6.72955057818E-05
 0
 7.30157957505E-05
 7.85651754248E-05
 .000207641196013
 .000182325182325
 .000135478761099

 4
 16
 20
 16
 13
 10
 3
 1
 8
 4
 22
 27
 10
 6
 6
 6
 7
 22
 17
 13

 .000043840420868
 .000176957872966
 .000198890192725
 .000178543531145
 .000138865151256
 .000181881013441
 .00004004004004
 1.35149745918E-05
 8.65800865801E-05
 .000043077453261
 .00025662560657
 .000269149487619
 8.60311260614E-05
 6.72955057818E-05
 0
 7.30157957505E-05
 7.85651754248E-05
 .000207641196013
 .000182325182325
 .000135478761099

 4
 16
 20
 16
 13
 10
 3
 1
 8
 4
 22
 27
 10
 6
 6
 6
 7
 22
 17
 13

 .001611135466901
 1.8691175332112E-03
 1.5811770321598E-03
 .001941660901195
 .0016877456845
 1.6187410196264E-03
 .001401401401404
 1.4596172559209E-03
 1.9264069264081E-03
 1.5400189540797E-03
 2.0996640537534E-03
 2.0834164041676E-03
 6.366303328542E-04
 7.738983164909E-04
 8.497981729342E-04
 8.396816511307E-04
 .003726234034437
 4.5303533675645E-03
 .0023166023166
 .002553253574555

 72.2380952380949
 120.384615384655
 143.805031446541
 155.149425287375
 139.265822784831
 81.2696629213518
 77.8761904761875
 78.0555555555959
 119.337078651686
 108.874125874119
 157.200000000012
 173.612440191419
 47.4324324324327
 41.0869565217184
 61.684210526318
 51.6376811594084
 324.096385542175
 468.150000000006
 193.333333333333
 213.359183673439

 .001611135466901
 1.8691175332112E-03
 1.5811770321598E-03
 .001941660901195
 .0016877456845
 1.6187410196264E-03
 .001401401401404
 1.4596172559209E-03
 1.9264069264081E-03
 1.5400189540797E-03
 2.0996640537534E-03
 2.0834164041676E-03
 6.366303328542E-04
 7.738983164909E-04
 8.497981729342E-04
 8.396816511307E-04
 .003726234034437
 4.5303533675645E-03
 .0023166023166
 .002553253574555

 72.2380952380949
 120.384615384655
 143.805031446541
 155.149425287375
 139.265822784831
 81.2696629213518
 77.8761904761875
 78.0555555555959
 119.337078651686
 108.874125874119
 157.200000000012
 173.612440191419
 47.4324324324327
 41.0869565217184
 61.684210526318
 51.6376811594084
 324.096385542175
 468.150000000006
 193.333333333333
 213.359183673439

 .000668566418238
 .000221197341208
 0
 0
 .00010681934712
 3.63762026882E-05
 .000213546880214
 .000135149745918
 .000292207792208
 .000129232359783
 6.99888017917E-05
 .000109653494956
 9.46342386675E-05
 .000168238764455
 0
 1.21692992917E-05

 61
 20
 20
 20
 10
 2
 16
 10
 27
 12
 6
 11
 11
 15
 15
 1

 .000668566418238
 .000221197341208
 0
 0
 .00010681934712
 3.63762026882E-05
 .000213546880214
 .000135149745918
 .000292207792208
 .000129232359783
 6.99888017917E-05
 .000109653494956
 9.46342386675E-05
 .000168238764455
 0
 1.21692992917E-05

 61
 20
 20
 20
 10
 2
 16
 10
 27
 12
 6
 11
 11
 15
 15
 1

 .000668566418238
 .000221197341208
 0
 0
 .00010681934712
 3.63762026882E-05
 .000213546880214
 .000135149745918
 .000292207792208
 .000129232359783
 6.99888017917E-05
 .000109653494956
 9.46342386675E-05
 .000168238764455
 0
 1.21692992917E-05

 61
 20
 20
 20
 10
 2
 16
 10
 27
 12
 6
 11
 11
 15
 15
 1

 .000942569048663
 1.6479201920032E-03
 1.5811770321598E-03
 .001941660901195
 .00158092633738
 1.5823648169382E-03
 .00118785452119
 1.3244675100029E-03
 1.6341991342001E-03
 1.4107865942967E-03
 2.0296752519617E-03
 1.9737629092116E-03
 5.419960941867E-04
 6.056595520359E-04
 8.497981729342E-04
 .000827512351839
 .003726234034437
 4.5303533675645E-03
 .0023166023166
 .002553253574555

 80.2093023255844
 133.8590604027
 143.805031446541
 155.149425287375
 148
 83.0919540229903
 89
 85.0000000000104
 135.847682119208
 117.748091603053
 162.413793103455
 182.646464646481
 53.7936507936499
 48.333333333332
 61.684210526318
 52.3823529411614
 324.096385542175
 468.150000000006
 193.333333333333
 213.359183673439

 .000032880315651
 1.6479201920032E-03
 1.5811770321598E-03
 .001941660901195
 .00158092633738
 1.5823648169382E-03
 .00118785452119
 1.3244675100029E-03
 1.6341991342001E-03
 1.4107865942967E-03
 2.0296752519617E-03
 1.9737629092116E-03
 5.419960941867E-04
 6.056595520359E-04
 8.497981729342E-04
 .000827512351839
 .003726234034437
 4.5303533675645E-03
 .0023166023166
 .002553253574555

 3
 133.8590604027
 143.805031446541
 155.149425287375
 148
 83.0919540229903
 89
 85.0000000000104
 135.847682119208
 117.748091603053
 162.413793103455
 182.646464646481
 53.7936507936499
 48.333333333332
 61.684210526318
 52.3823529411614
 324.096385542175
 468.150000000006
 193.333333333333
 213.359183673439

 .000032880315651
 1.6479201920032E-03
 1.5811770321598E-03
 .001941660901195
 .00158092633738
 1.5823648169382E-03
 .00118785452119
 1.3244675100029E-03
 1.6341991342001E-03
 1.4107865942967E-03
 2.0296752519617E-03
 1.9737629092116E-03
 5.419960941867E-04
 6.056595520359E-04
 8.497981729342E-04
 .000827512351839
 .003726234034437
 4.5303533675645E-03
 .0023166023166
 .002553253574555

 3
 133.8590604027
 143.805031446541
 155.149425287375
 148
 83.0919540229903
 89
 85.0000000000104
 135.847682119208
 117.748091603053
 162.413793103455
 182.646464646481
 53.7936507936499
 48.333333333332
 61.684210526318
 52.3823529411614
 324.096385542175
 468.150000000006
 193.333333333333
 213.359183673439

 .000909688733012

 83

 .000909688733012

 83

 0
 1.10598670604E-05
 2.98335289087E-05
 8.92717655723E-05
 .000010681934712
 7.27524053764E-05
 0
 0
 0
 0
 2.33296005972E-05
 1.99369990829E-05
 0
 0
 0
 1.21692992917E-05
 0
 0
 6.43500643501E-05
 3.12643294843E-05

 0
 1
 3
 8
 1
 4
 4
 4
 4
 4
 2
 2
 2
 2
 2
 1
 1
 1
 6
 3

 0
 1.10598670604E-05
 2.98335289087E-05
 8.92717655723E-05
 .000010681934712
 7.27524053764E-05
 0
 0
 0
 0
 2.33296005972E-05
 1.99369990829E-05
 0
 0
 0
 1.21692992917E-05
 0
 0
 6.43500643501E-05
 3.12643294843E-05

 0
 1
 3
 8
 1
 4
 4
 4
 4
 4
 2
 2
 2
 2
 2
 1
 1
 1
 6
 3

 0
 1.10598670604E-05
 2.98335289087E-05
 8.92717655723E-05
 .000010681934712
 7.27524053764E-05
 0
 0
 0
 0
 2.33296005972E-05
 1.99369990829E-05
 0
 0
 0
 1.21692992917E-05
 0
 0
 6.43500643501E-05
 3.12643294843E-05

 0
 1
 3
 8
 1
 4
 4
 4
 4
 4
 2
 2
 2
 2
 2
 1
 1
 1
 6
 3

 0
 1.10598670604E-05
 2.98335289087E-05
 8.92717655723E-05
 .000010681934712
 7.27524053764E-05
 0
 0
 0
 0
 2.33296005972E-05
 1.99369990829E-05
 0
 0
 0
 1.21692992917E-05
 0
 0
 6.43500643501E-05
 3.12643294843E-05

 0
 1
 3
 8
 1
 4
 4
 4
 4
 4
 2
 2
 2
 2
 2
 1
 1
 1
 6
 3

 0
 1.10598670604E-05
 2.98335289087E-05
 8.92717655723E-05
 .000010681934712
 7.27524053764E-05
 0
 0
 0
 0
 2.33296005972E-05
 1.99369990829E-05
 0
 0
 0
 1.21692992917E-05
 0
 0
 6.43500643501E-05
 3.12643294843E-05

 0
 1
 3
 8
 1
 4
 4
 4
 4
 4
 2
 2
 2
 2
 2
 1
 1
 1
 6
 3

 0
 0
 6.96115674536E-05
 3.34769120896E-05
 0
 0
 1.33466800133E-05
 1.35149745918E-05
 0
 1.07693633152E-05
 1.16648002986E-05
 2.99054986243E-05
 0
 0
 2.23631098141E-05
 3.65078978752E-05
 0
 1.88764723648E-05
 5.36250536251E-05
 4.16857726458E-05

 0
 0
 7
 3
 3
 3
 1
 1
 1
 1
 1
 3
 3
 3
 2
 3
 3
 2
 5
 4

 0
 0
 6.96115674536E-05
 3.34769120896E-05
 0
 0
 1.33466800133E-05
 1.35149745918E-05
 0
 1.07693633152E-05
 1.16648002986E-05
 2.99054986243E-05
 0
 0
 2.23631098141E-05
 3.65078978752E-05
 0
 1.88764723648E-05
 5.36250536251E-05
 4.16857726458E-05

 0
 0
 7
 3
 3
 3
 1
 1
 1
 1
 1
 3
 3
 3
 2
 3
 3
 2
 5
 4

 0
 0
 6.96115674536E-05
 3.34769120896E-05
 0
 0
 1.33466800133E-05
 1.35149745918E-05
 0
 1.07693633152E-05
 1.16648002986E-05
 2.99054986243E-05
 0
 0
 2.23631098141E-05
 3.65078978752E-05
 0
 1.88764723648E-05
 5.36250536251E-05
 4.16857726458E-05

 0
 0
 7
 3
 3
 3
 1
 1
 1
 1
 1
 3
 3
 3
 2
 3
 3
 2
 5
 4

 0
 0
 6.96115674536E-05
 3.34769120896E-05
 0
 0
 1.33466800133E-05
 1.35149745918E-05
 0
 1.07693633152E-05
 1.16648002986E-05
 2.99054986243E-05
 0
 0
 2.23631098141E-05
 3.65078978752E-05
 0
 1.88764723648E-05
 5.36250536251E-05
 4.16857726458E-05

 0
 0
 7
 3
 3
 3
 1
 1
 1
 1
 1
 3
 3
 3
 2
 3
 3
 2
 5
 4

 0
 0
 6.96115674536E-05
 3.34769120896E-05
 0
 0
 1.33466800133E-05
 1.35149745918E-05
 0
 1.07693633152E-05
 1.16648002986E-05
 2.99054986243E-05
 0
 0
 2.23631098141E-05
 3.65078978752E-05
 0
 1.88764723648E-05
 5.36250536251E-05
 4.16857726458E-05

 0
 0
 7
 3
 3
 3
 1
 1
 1
 1
 1
 3
 3
 3
 2
 3
 3
 2
 5
 4

 .000010960105217
 2.21197341208E-05
 9.94450963623E-06
 4.46358827862E-05
 .00021363869424
 .000327385824194
 .000133466800133
 2.70299491837E-05
 .00021645021645
 .000269234082881
 6.99888017917E-05
 2.99054986243E-05
 0
 1.12159176303E-05
 .000011181554907
 1.21692992917E-05
 0
 0
 .00010725010725
 .000093792988453

 1
 2
 1
 4
 20
 18
 10
 2
 20
 25
 6
 3
 3
 1
 1
 1
 1
 1
 10
 9

 .000010960105217
 2.21197341208E-05
 9.94450963623E-06
 4.46358827862E-05
 .00021363869424
 .000327385824194
 .000133466800133
 2.70299491837E-05
 .00021645021645
 .000269234082881
 6.99888017917E-05
 2.99054986243E-05
 0
 1.12159176303E-05
 .000011181554907
 1.21692992917E-05
 0
 0
 .00010725010725
 .000093792988453

 1
 2
 1
 4
 20
 18
 10
 2
 20
 25
 6
 3
 3
 1
 1
 1
 1
 1
 10
 9

 .000010960105217
 2.21197341208E-05
 9.94450963623E-06
 4.46358827862E-05
 .00021363869424
 .000327385824194
 .000133466800133
 2.70299491837E-05
 .00021645021645
 .000269234082881
 6.99888017917E-05
 2.99054986243E-05
 0
 1.12159176303E-05
 .000011181554907
 1.21692992917E-05
 0
 0
 .00010725010725
 .000093792988453

 1
 2
 1
 4
 20
 18
 10
 2
 20
 25
 6
 3
 3
 1
 1
 1
 1
 1
 10
 9

 .000010960105217
 2.21197341208E-05
 9.94450963623E-06
 4.46358827862E-05
 .00021363869424
 .000327385824194
 .000133466800133
 2.70299491837E-05
 .00021645021645
 .000269234082881
 6.99888017917E-05
 2.99054986243E-05
 0
 1.12159176303E-05
 .000011181554907
 1.21692992917E-05
 0
 0
 .00010725010725
 .000093792988453

 1
 2
 1
 4
 20
 18
 10
 2
 20
 25
 6
 3
 3
 1
 1
 1
 1
 1
 10
 9

 .000010960105217
 2.21197341208E-05
 9.94450963623E-06
 4.46358827862E-05
 .00021363869424
 .000327385824194
 .000133466800133
 2.70299491837E-05
 .00021645021645
 .000269234082881
 6.99888017917E-05
 2.99054986243E-05
 0
 1.12159176303E-05
 .000011181554907
 1.21692992917E-05
 0
 0
 .00010725010725
 .000093792988453

 1
 2
 1
 4
 20
 18
 10
 2
 20
 25
 6
 3
 3
 1
 1
 1
 1
 1
 10
 9

 .000010960105217
 2.21197341208E-05
 9.94450963623E-06
 4.46358827862E-05
 .00021363869424
 .000327385824194
 .000133466800133
 2.70299491837E-05
 .00021645021645
 .000269234082881
 6.99888017917E-05
 2.99054986243E-05
 0
 1.12159176303E-05
 .000011181554907
 1.21692992917E-05
 0
 0
 .00010725010725
 .000093792988453

 1
 2
 1
 4
 20
 18
 10
 2
 20
 25
 6
 3
 3
 1
 1
 1
 1
 1
 10
 9

 .000591845681719
 .000508753884778
 .000208834702361
 .00029013323811
 .000459323192617
 .000618395445699
 .000520520520521
 .00036490431398
 .00116883116883
 .00114155251142
 .000828200821202
 .000787511463774
 .000232284040366
 .000347693446539
 .000279538872676
 .000304232482294
 .000258142719253
 .000103820598007
 .000600600600601
 .000625286589687

 54
 46
 21
 26
 43
 34
 39
 27
 108
 106
 71
 79
 27
 31
 25
 25
 23
 11
 56
 60

 .000591845681719
 .000508753884778
 .000208834702361
 .00029013323811
 .000459323192617
 .000618395445699
 .000520520520521
 .00036490431398
 .00116883116883
 .00114155251142
 .000828200821202
 .000787511463774
 .000232284040366
 .000347693446539
 .000279538872676
 .000304232482294
 .000258142719253
 .000103820598007
 .000600600600601
 .000625286589687

 54
 46
 21
 26
 43
 34
 39
 27
 108
 106
 71
 79
 27
 31
 25
 25
 23
 11
 56
 60

 .000591845681719
 .000508753884778
 .000208834702361
 .00029013323811
 .000459323192617
 .000618395445699
 .000520520520521
 .00036490431398
 .00116883116883
 .00114155251142
 .000828200821202
 .000787511463774
 .000232284040366
 .000347693446539
 .000279538872676
 .000304232482294
 .000258142719253
 .000103820598007
 .000600600600601
 .000625286589687

 54
 46
 21
 26
 43
 34
 39
 27
 108
 106
 71
 79
 27
 31
 25
 25
 23
 11
 56
 60

 .000591845681719
 .000508753884778
 .000208834702361
 .00029013323811
 .000459323192617
 .000618395445699
 .000520520520521
 .00036490431398
 .00116883116883
 .00114155251142
 .000828200821202
 .000787511463774
 .000232284040366
 .000347693446539
 .000279538872676
 .000304232482294
 .000258142719253
 .000103820598007
 .000600600600601
 .000625286589687

 54
 46
 21
 26
 43
 34
 39
 27
 108
 106
 71
 79
 27
 31
 25
 25
 23
 11
 56
 60

 .000591845681719
 .000508753884778
 .000208834702361
 .00029013323811
 .000459323192617
 .000618395445699
 .000520520520521
 .00036490431398
 .00116883116883
 .00114155251142
 .000828200821202
 .000787511463774
 .000232284040366
 .000347693446539
 .000279538872676
 .000304232482294
 .000258142719253
 .000103820598007
 .000600600600601
 .000625286589687

 54
 46
 21
 26
 43
 34
 39
 27
 108
 106
 71
 79
 27
 31
 25
 25
 23
 11
 56
 60

 .000591845681719
 .000508753884778
 .000208834702361
 .00029013323811
 .000459323192617
 .000618395445699
 .000520520520521
 .00036490431398
 .00116883116883
 .00114155251142
 .000828200821202
 .000787511463774
 .000232284040366
 .000347693446539
 .000279538872676
 .000304232482294
 .000258142719253
 .000103820598007
 .000600600600601
 .000625286589687

 54
 46
 21
 26
 43
 34
 39
 27
 108
 106
 71
 79
 27
 31
 25
 25
 23
 11
 56
 60

 8.71328364752453E-02
 7.16679385513924E-02
 2.77949044332225E-02
 2.44381458254095E-02
 5.96479234319508E-02
 5.53645804914056E-02
 7.08041374708272E-02
 6.78992323494264E-02
 5.85714285714569E-02
 6.13530628068917E-02
 4.77323628219064E-02
 4.59149088879853E-02
 6.22779321558782E-02
 .060969728238343
 2.15021300861948E-02
 2.16126755421454E-02
 2.42766392062648E-02
 2.93623527634803E-02
 5.23916773916832E-02
 4.63128934094464E-02

 3889.52025157097
 1508.90833333373
 844.952057244632
 708.190867579455
 1057.39040114645
 517.345597897236
 1311.4750235624
 1337.81608280267
 1236.89911308195
 1173.84044233783
 1103.92375366507
 1305.07859314028
 1544.93562646809
 1085.3259749816
 745.387935517189
 631.931306306299
 451.861766065603
 828.974284795651
 1402.09477993839
 1271.23537353701

 .000010960105217
 2.21197341208E-05
 0
 0
 4.27277388481E-05
 .000109128608065
 .0002002002002
 .000108119796735
 6.49350649351E-05
 5.38468165762E-05
 0
 0
 0
 0
 .000011181554907
 1.21692992917E-05
 0
 9.43823618242E-06

 1
 2
 2
 2
 4
 6
 15
 8
 6
 5
 5
 5
 5
 5
 1
 1
 1
 1

 .000010960105217
 2.21197341208E-05
 0
 0
 4.27277388481E-05
 .000109128608065
 .0002002002002
 .000108119796735
 6.49350649351E-05
 5.38468165762E-05
 0
 0
 0
 0
 .000011181554907
 1.21692992917E-05
 0
 9.43823618242E-06

 1
 2
 2
 2
 4
 6
 15
 8
 6
 5
 5
 5
 5
 5
 1
 1
 1
 1

 .000010960105217
 2.21197341208E-05
 0
 0
 4.27277388481E-05
 .000109128608065
 .0002002002002
 .000108119796735
 6.49350649351E-05
 5.38468165762E-05
 0
 0
 0
 0
 .000011181554907
 1.21692992917E-05
 0
 9.43823618242E-06

 1
 2
 2
 2
 4
 6
 15
 8
 6
 5
 5
 5
 5
 5
 1
 1
 1
 1

 .000010960105217
 2.21197341208E-05
 0
 0
 4.27277388481E-05
 .000109128608065
 .0002002002002
 .000108119796735
 6.49350649351E-05
 5.38468165762E-05
 0
 0
 0
 0
 .000011181554907
 1.21692992917E-05
 0
 9.43823618242E-06

 1
 2
 2
 2
 4
 6
 15
 8
 6
 5
 5
 5
 5
 5
 1
 1
 1
 1

 .000010960105217
 2.21197341208E-05
 0
 0
 4.27277388481E-05
 .000109128608065
 .0002002002002
 .000108119796735
 6.49350649351E-05
 5.38468165762E-05
 0
 0
 0
 0
 .000011181554907
 1.21692992917E-05
 0
 9.43823618242E-06

 1
 2
 2
 2
 4
 6
 15
 8
 6
 5
 5
 5
 5
 5
 1
 1
 1
 1

 .000021920210434
 2.21197341208E-05
 0
 0
 4.27277388481E-05
 0
 9.34267600934E-05
 9.46048221429E-05
 1.08225108225E-05
 0
 2.33296005972E-05
 9.96849954145E-06
 0
 0
 0
 0
 0
 0
 5.36250536251E-05
 1.04214431614E-05

 2
 2
 2
 2
 4
 4
 7
 7
 1
 1
 2
 1
 1
 1
 1
 1
 1
 1
 5
 1

 .000021920210434
 2.21197341208E-05
 0
 0
 4.27277388481E-05
 0
 9.34267600934E-05
 9.46048221429E-05
 1.08225108225E-05
 0
 2.33296005972E-05
 9.96849954145E-06
 0
 0
 0
 0
 0
 0
 5.36250536251E-05
 1.04214431614E-05

 2
 2
 2
 2
 4
 4
 7
 7
 1
 1
 2
 1
 1
 1
 1
 1
 1
 1
 5
 1

 .000021920210434
 2.21197341208E-05
 0
 0
 4.27277388481E-05
 0
 9.34267600934E-05
 9.46048221429E-05
 1.08225108225E-05
 0
 2.33296005972E-05
 9.96849954145E-06
 0
 0
 0
 0
 0
 0
 5.36250536251E-05
 1.04214431614E-05

 2
 2
 2
 2
 4
 4
 7
 7
 1
 1
 2
 1
 1
 1
 1
 1
 1
 1
 5
 1

 .000021920210434
 2.21197341208E-05
 0
 0
 4.27277388481E-05
 0
 9.34267600934E-05
 9.46048221429E-05
 1.08225108225E-05
 0
 2.33296005972E-05
 9.96849954145E-06
 0
 0
 0
 0
 0
 0
 5.36250536251E-05
 1.04214431614E-05

 2
 2
 2
 2
 4
 4
 7
 7
 1
 1
 2
 1
 1
 1
 1
 1
 1
 1
 5
 1

 .000021920210434
 2.21197341208E-05
 0
 0
 4.27277388481E-05
 0
 9.34267600934E-05
 9.46048221429E-05
 1.08225108225E-05
 0
 2.33296005972E-05
 9.96849954145E-06
 0
 0
 0
 0
 0
 0
 5.36250536251E-05
 1.04214431614E-05

 2
 2
 2
 2
 4
 4
 7
 7
 1
 1
 2
 1
 1
 1
 1
 1
 1
 1
 5
 1

 5.98312143796371E-02
 3.27040268976316E-02
 8.7909465184233E-03
 4.3743165130414E-03
 1.73581439070582E-02
 1.70968152634636E-02
 2.05138471805578E-02
 1.92588387933663E-02
 .021461038961087
 2.40910657361652E-02
 .012667973124313
 1.18126719566491E-02
 4.85215550986114E-03
 .004239616864253
 2.8177518365742E-03
 .002701584442768
 7.6095984197141E-03
 3.96405919661052E-03
 5.7271557271561E-03
 4.9397640585221E-03

 5337.83714966114
 2189.11092323322
 838.821266968315
 359.04591836732
 994.158153846382
 539.706382978711
 1236.41834743063
 1126.18877193
 1327.3489662134
 1369.31738936028
 965.401473296559
 967.303797468669
 148.602836879419
 127.613756613747
 210.793650793704
 162.783783783763
 496.970501474912
 222.533333333396
 293.966292134868
 287.181434599096

 5.98312143796371E-02
 3.27040268976316E-02
 8.7909465184233E-03
 4.3743165130414E-03
 1.73581439070582E-02
 1.70968152634636E-02
 2.05138471805578E-02
 1.92588387933663E-02
 .021461038961087
 2.40910657361652E-02
 .012667973124313
 1.18126719566491E-02
 4.85215550986114E-03
 .004239616864253
 2.8177518365742E-03
 .002701584442768
 7.6095984197141E-03
 3.96405919661052E-03
 5.7271557271561E-03
 4.9397640585221E-03

 5337.83714966114
 2189.11092323322
 838.821266968315
 359.04591836732
 994.158153846382
 539.706382978711
 1236.41834743063
 1126.18877193
 1327.3489662134
 1369.31738936028
 965.401473296559
 967.303797468669
 148.602836879419
 127.613756613747
 210.793650793704
 162.783783783763
 496.970501474912
 222.533333333396
 293.966292134868
 287.181434599096

 5.98312143796371E-02
 3.26929670305712E-02
 8.7909465184233E-03
 4.3743165130414E-03
 1.73047342334982E-02
 1.70604390607754E-02
 2.01935268602378E-02
 1.89479943777543E-02
 2.14502164502645E-02
 .02408029637285
 .012574654721924
 1.17129869612346E-02
 4.85215550986114E-03
 .004239616864253
 2.8177518365742E-03
 .002701584442768
 7.6095984197141E-03
 3.96405919661052E-03
 5.7271557271561E-03
 4.9397640585221E-03

 5337.83714966114
 2189.85115020319
 838.821266968315
 359.04591836732
 997.218518518737
 540.852878464806
 1255.65036351671
 1144.2867332384
 1328.01816347183
 1369.92933810328
 972.506493506548
 975.451063830083
 148.602836879419
 127.613756613747
 210.793650793704
 162.783783783763
 496.970501474912
 222.533333333396
 293.966292134868
 287.181434599096

 .0591626479614
 .0279925235299
 .00856222279679
 .0041846140112
 .0133096906512
 .0126589185355
 .0183516850184
 .0170558979349
 .017380952381
 .0185340742655
 .0119330907055
 .0106463575103
 .00164319450777
 .00150293296246
 .00257175762862
 .00229999756614
 .00646479157781
 .00266158260344
 .00415057915058
 .00377256242444

 5398
 2531
 861
 375
 1246
 696
 1375
 1262
 1606
 1721
 1023
 1068
 191
 134
 230
 189
 576
 282
 387
 362

 .0591626479614
 .0279925235299
 .00856222279679
 .0041846140112
 .0133096906512
 .0126589185355
 .0183516850184
 .0170558979349
 .017380952381
 .0185340742655
 .0119330907055
 .0106463575103
 .00164319450777
 .00150293296246
 .00257175762862
 .00229999756614
 .00646479157781
 .00266158260344
 .00415057915058
 .00377256242444

 5398
 2531
 861
 375
 1246
 696
 1375
 1262
 1606
 1721
 1023
 1068
 191
 134
 230
 189
 576
 282
 387
 362

 .000186321788689
 .00251058982271
 9.94450963623E-05
 .000100430736269
 .0025743462656
 .00260089849221
 .00121454788121
 .00141907233214
 .00224025974026
 .00310157663479
 .000489921612542
 .000667889469277
 .0015399571565
 .00188427416189
 .000145360213791
 .00020687808796
 1.12235964893E-05
 4.71911809121E-05
 .000729300729301
 .000677393805494

 17
 227
 10
 9
 241
 143
 91
 105
 207
 288
 42
 67
 179
 168
 13
 17
 1
 5
 68
 65

 .000186321788689
 .00251058982271
 9.94450963623E-05
 .000100430736269
 .0025743462656
 .00260089849221
 .00121454788121
 .00141907233214
 .00224025974026
 .00310157663479
 .000489921612542
 .000667889469277
 .0015399571565
 .00188427416189
 .000145360213791
 .00020687808796
 1.12235964893E-05
 4.71911809121E-05
 .000729300729301
 .000677393805494

 17
 227
 10
 9
 241
 143
 91
 105
 207
 288
 42
 67
 179
 168
 13
 17
 1
 5
 68
 65

 .000010960105217
 1.10598670604E-05
 0
 0
 0
 0
 0
 0
 0
 0
 1.16648002986E-05
 1.99369990829E-05
 8.60311260614E-06
 0
 0
 0
 .000314260701699
 .000141573542736

 1
 1
 1
 1
 1
 1
 1
 1
 1
 1
 1
 2
 1
 1
 1
 1
 28
 15

 .000010960105217
 1.10598670604E-05
 0
 0
 0
 0
 0
 0
 0
 0
 1.16648002986E-05
 1.99369990829E-05
 8.60311260614E-06
 0
 0
 0
 .000314260701699
 .000141573542736

 1
 1
 1
 1
 1
 1
 1
 1
 1
 1
 1
 2
 1
 1
 1
 1
 28
 15

 .000186321788689
 .00028755654357
 0
 0
 9.61374124081E-05
 7.27524053764E-05
 2.66933600267E-05
 0
 .000108225108225
 .000043077453261
 0
 1.99369990829E-05
 .000903326823645
 .000347693446539
 .000011181554907
 0
 2.24471929785E-05
 0
 8.58000858001E-05
 6.25286589687E-05

 17
 26
 26
 26
 9
 4
 2
 2
 10
 4
 4
 2
 105
 31
 1
 1
 2
 2
 8
 6

 .000186321788689
 .00028755654357
 0
 0
 9.61374124081E-05
 7.27524053764E-05
 2.66933600267E-05
 0
 .000108225108225
 .000043077453261
 0
 1.99369990829E-05
 .000903326823645
 .000347693446539
 .000011181554907
 0
 2.24471929785E-05
 0
 8.58000858001E-05
 6.25286589687E-05

 17
 26
 26
 26
 9
 4
 2
 2
 10
 4
 4
 2
 105
 31
 1
 1
 2
 2
 8
 6

 .000021920210434
 .000132718404725
 3.97780385449E-05
 2.23179413931E-05
 .000448641257905
 .000545643040323
 .000106773440107
 5.40598983674E-05
 .000292207792208
 .000495390712501
 1.16648002986E-05
 2.99054986243E-05
 .000258093378184
 .000179454682085
 .000011181554907
 0
 5.61179824463E-05
 9.43823618242E-06
 .0003861003861
 .000177164533745

 2
 12
 4
 2
 42
 30
 8
 4
 27
 46
 1
 3
 30
 16
 1
 1
 5
 1
 36
 17

 .000021920210434
 .000132718404725
 3.97780385449E-05
 2.23179413931E-05
 .000448641257905
 .000545643040323
 .000106773440107
 5.40598983674E-05
 .000292207792208
 .000495390712501
 1.16648002986E-05
 2.99054986243E-05
 .000258093378184
 .000179454682085
 .000011181554907
 0
 5.61179824463E-05
 9.43823618242E-06
 .0003861003861
 .000177164533745

 2
 12
 4
 2
 42
 30
 8
 4
 27
 46
 1
 3
 30
 16
 1
 1
 5
 1
 36
 17

 .000164401578255
 .00131612418019
 0
 0
 .000491368996753
 .000418326330914
 8.00800800801E-05
 6.75748729592E-05
 .000995670995671
 .00129232359783
 1.16648002986E-05
 9.96849954145E-06

 15
 119
 119
 119
 46
 23
 6
 5
 92
 120
 1
 1

 .000164401578255
 .00131612418019
 0
 0
 .000491368996753
 .000418326330914
 8.00800800801E-05
 6.75748729592E-05
 .000995670995671
 .00129232359783
 1.16648002986E-05
 9.96849954145E-06

 15
 119
 119
 119
 46
 23
 6
 5
 92
 120
 1
 1

 .000043840420868
 .000221197341208
 0
 0
 6.40916082721E-05
 .000236445317473
 2.66933600267E-05
 2.70299491837E-05
 .000140692640693
 .000193848539674
 1.16648002986E-05
 0
 0
 0
 0
 0
 0
 0
 0
 1.04214431614E-05

 4
 20
 20
 20
 6
 13
 2
 2
 13
 18
 1
 1
 1
 1
 1
 1
 1
 1
 1
 1

 .000043840420868
 .000221197341208
 0
 0
 6.40916082721E-05
 .000236445317473
 2.66933600267E-05
 2.70299491837E-05
 .000140692640693
 .000193848539674
 1.16648002986E-05
 0
 0
 0
 0
 0
 0
 0
 0
 1.04214431614E-05

 4
 20
 20
 20
 6
 13
 2
 2
 13
 18
 1
 1
 1
 1
 1
 1
 1
 1
 1
 1

 5.48005260851E-05
 .000199077607087
 8.95005867261E-05
 6.69538241793E-05
 .00032045804136
 .000527454938979
 .000373707040374
 .000324359390204
 .00025974025974
 .000420005169294
 .000093318402389
 .000318991985326
 .000498980531156
 .000325261611279
 7.82708843492E-05
 .000097354394334
 .000740757368291
 .00110427363334
 .000375375375375
 .000239693192713

 5
 18
 9
 6
 30
 29
 28
 24
 24
 39
 8
 32
 58
 29
 7
 8
 66
 117
 35
 23

 5.48005260851E-05
 .000199077607087
 8.95005867261E-05
 6.69538241793E-05
 .00032045804136
 .000527454938979
 .000373707040374
 .000324359390204
 .00025974025974
 .000420005169294
 .000093318402389
 .000318991985326
 .000498980531156
 .000325261611279
 7.82708843492E-05
 .000097354394334
 .000740757368291
 .00110427363334
 .000375375375375
 .000239693192713

 5
 18
 9
 6
 30
 29
 28
 24
 24
 39
 8
 32
 58
 29
 7
 8
 66
 117
 35
 23

 0
 2.21197341208E-05
 0
 0
 0
 0
 1.33466800133E-05
 0
 3.24675324675E-05
 0
 1.16648002986E-05
 0
 0
 0
 0
 .000097354394334

 0
 2
 2
 2
 2
 2
 1
 1
 3
 3
 1
 1
 1
 1
 1
 8

 0
 2.21197341208E-05
 0
 0
 0
 0
 1.33466800133E-05
 0
 3.24675324675E-05
 0
 1.16648002986E-05
 0
 0
 0
 0
 .000097354394334

 0
 2
 2
 2
 2
 2
 1
 1
 3
 3
 1
 1
 1
 1
 1
 8

 0
 1.10598670604E-05
 0
 0
 .000021363869424
 3.63762026882E-05
 .00032032032032
 .000310844415612
 0
 1.07693633152E-05

 0
 1
 1
 1
 2
 2
 24
 23
 23
 1

 0
 1.10598670604E-05
 0
 0
 .000021363869424
 3.63762026882E-05
 .00032032032032
 .000310844415612
 0
 1.07693633152E-05

 0
 1
 1
 1
 2
 2
 24
 23
 23
 1

 0
 1.10598670604E-05
 0
 0
 .000021363869424
 3.63762026882E-05
 .00032032032032
 .000310844415612
 0
 1.07693633152E-05

 0
 1
 1
 1
 2
 2
 24
 23
 23
 1

 0
 0
 0
 0
 .000032045804136
 0
 0
 0
 1.08225108225E-05
 0
 .000093318402389
 9.96849954145E-05

 0
 0
 0
 0
 3
 3
 3
 3
 1
 1
 8
 10

 0
 0
 0
 0
 .000032045804136
 0
 0
 0
 1.08225108225E-05
 0
 .000093318402389
 9.96849954145E-05

 0
 0
 0
 0
 3
 3
 3
 3
 1
 1
 8
 10

 0
 0
 0
 0
 .000032045804136
 0
 0
 0
 1.08225108225E-05
 0
 .000093318402389
 9.96849954145E-05

 0
 0
 0
 0
 3
 3
 3
 3
 1
 1
 8
 10

 1.7755370451561E-03
 2.8092062333406E-03
 3.480578372681E-04
 5.356305934345E-04
 4.7107332080012E-03
 3.9104417889782E-03
 2.4557891224506E-03
 2.2299708076596E-03
 1.0930735930737E-03
 1.4000172309811E-03
 1.0498320268759E-03
 1.1065034491002E-03
 6.1942410764225E-03
 5.7089020738248E-03
 7.827088434921E-04
 6.693114610461E-04
 3.254842981893E-04
 2.170794321958E-04
 .00146932646933
 1.2088874067289E-03

 50.4567901234673
 128.38582677165
 20.82857142857
 24.2916666666681
 228.274376417327
 86.3860465115532
 71.054347826023
 79.1212121212874
 48.4653465346616
 68.1846153846265
 47.4888888888985
 57.2162162162196
 386.369444444516
 264.434184675852
 62.3142857142874
 42.2363636363726
 14.655172413789
 19.3478260869611
 85.8759124088242
 67.603448275851

 1.7755370451561E-03
 2.8092062333406E-03
 3.480578372681E-04
 5.356305934345E-04
 4.7107332080012E-03
 3.9104417889782E-03
 2.4557891224506E-03
 2.2299708076596E-03
 1.0930735930737E-03
 1.4000172309811E-03
 1.0498320268759E-03
 1.1065034491002E-03
 6.1942410764225E-03
 5.7089020738248E-03
 7.827088434921E-04
 6.693114610461E-04
 3.254842981893E-04
 2.170794321958E-04
 .00146932646933
 1.2088874067289E-03

 50.4567901234673
 128.38582677165
 20.82857142857
 24.2916666666681
 228.274376417327
 86.3860465115532
 71.054347826023
 79.1212121212874
 48.4653465346616
 68.1846153846265
 47.4888888888985
 57.2162162162196
 386.369444444516
 264.434184675852
 62.3142857142874
 42.2363636363726
 14.655172413789
 19.3478260869611
 85.8759124088242
 67.603448275851

 .000043840420868
 8.84789364832E-05
 0
 0
 0
 0
 .000173506840174
 8.10898475511E-05
 0
 1.07693633152E-05

 4
 8
 8
 8
 8
 8
 13
 6
 6
 1

 .000043840420868
 8.84789364832E-05
 0
 0
 0
 0
 .000173506840174
 8.10898475511E-05
 0
 1.07693633152E-05

 4
 8
 8
 8
 8
 8
 13
 6
 6
 1

 .000043840420868
 8.84789364832E-05
 0
 0
 0
 0
 .000173506840174
 8.10898475511E-05
 0
 1.07693633152E-05

 4
 8
 8
 8
 8
 8
 13
 6
 6
 1

 .000931608943446
 .000519813751839
 1.98890192725E-05
 1.11589706965E-05
 8.866005810971E-04
 .00092759316855
 6.806806806803E-04
 4.730241107147E-04
 3.138528138526E-04
 3.553889894027E-04
 3.616088092569E-04
 3.4889748395045E-04
 1.806653647294E-04
 4.48636705212E-05
 2.23631098141E-05
 1.21692992917E-05
 .000112235964893
 0
 .0000429000429
 .000093792988453

 48.6705882353074
 21.8510638297961
 2
 1
 39.8674698795256
 29.0392156862721
 18.098039215688
 15.7999999999982
 17.6896551724097
 17.2424242424187
 25.5806451612854
 33.057142857142
 15.85714285715
 2
 2
 1
 10
 10
 2.5
 9

 .000887768522578
 .000464514416537
 1.98890192725E-05
 1.11589706965E-05
 .000299094171936
 .000291009621506
 .000547213880547
 .000445994161531
 .000238095238095
 .000236925992935
 .000326614408361
 .000338928984409
 2.58093378184E-05
 2.24318352606E-05
 0
 1.21692992917E-05
 0
 0
 .000032175032175
 .000093792988453

 50.8765432098879
 24.0476190476281
 2
 1
 28
 16
 20.5121951219509
 16.6363636363618
 22
 22
 28
 34
 3
 2
 2
 1
 1
 1
 3
 9

 .000887768522578
 .000464514416537
 1.98890192725E-05
 1.11589706965E-05
 .000299094171936
 .000291009621506
 .000547213880547
 .000445994161531
 .000238095238095
 .000236925992935
 .000326614408361
 .000338928984409
 2.58093378184E-05
 2.24318352606E-05
 0
 1.21692992917E-05
 0
 0
 .000032175032175
 .000093792988453

 50.8765432098879
 24.0476190476281
 2
 1
 28
 16
 20.5121951219509
 16.6363636363618
 22
 22
 28
 34
 3
 2
 2
 1
 1
 1
 3
 9

 .000043840420868
 4.42394682416E-05
 0
 0
 .000534096735601
 .000636583547044
 .00012012012012
 2.70299491837E-05
 5.41125541126E-05
 9.69242698372E-05
 3.49944008959E-05
 9.96849954145E-06
 0
 0
 2.23631098141E-05

 4
 4
 4
 4
 50
 35
 9
 2
 5
 9
 3
 1
 1
 1
 2

 .000043840420868
 4.42394682416E-05
 0
 0
 .000534096735601
 .000636583547044
 .00012012012012
 2.70299491837E-05
 5.41125541126E-05
 9.69242698372E-05
 3.49944008959E-05
 9.96849954145E-06
 0
 0
 2.23631098141E-05

 4
 4
 4
 4
 50
 35
 9
 2
 5
 9
 3
 1
 1
 1
 2

 0
 1.10598670604E-05
 0
 0
 5.34096735601E-05
 0
 1.33466800133E-05
 0
 .000021645021645
 2.15387266305E-05
 0
 0
 .000154856026911
 2.24318352606E-05
 0
 0
 .000112235964893
 0
 .000010725010725

 0
 1
 1
 1
 5
 5
 1
 1
 2
 2
 2
 2
 18
 2
 2
 2
 10
 10
 1

 0
 1.10598670604E-05
 0
 0
 5.34096735601E-05
 0
 1.33466800133E-05
 0
 .000021645021645
 2.15387266305E-05
 0
 0
 .000154856026911
 2.24318352606E-05
 0
 0
 .000112235964893
 0
 .000010725010725

 0
 1
 1
 1
 5
 5
 1
 1
 2
 2
 2
 2
 18
 2
 2
 2
 10
 10
 1

 8.000876808421E-04
 .002189853677958
 3.281688179956E-04
 .000524471622738
 3.7173132797841E-03
 2.9646605190841E-03
 1.6016016015963E-03
 1.6758568493938E-03
 7.792207792211E-04
 1.0338588782632E-03
 .000688223217619
 7.5760596514975E-04
 .005944750810844
 .005641606568043
 .000749164178771
 6.571421617544E-04
 2.132483332963E-04
 2.170794321958E-04
 .001415701415705
 .001094251531953

 55.0821917808257
 159.181818181839
 21.9696969696977
 24.7872340425523
 279.48275862075
 104.852760736142
 99.8500000000017
 100.532258064559
 60.8611111111105
 86.395833333334
 59
 68.3421052631603
 402.010130246119
 267.56461232606
 65.0298507462713
 43.0000000000061
 17.1052631578931
 19.3478260869611
 89.0454545455109
 73.8761904761712

 .000043840420868
 8.84789364832E-05
 0
 0
 0
 0
 0
 0
 0
 0
 0
 9.96849954145E-06
 0
 0
 0
 0
 0

 4
 8
 8
 8
 8
 8
 8
 8
 8
 8
 8
 1
 1
 1
 1
 1
 1

 .000043840420868
 8.84789364832E-05
 0
 0
 0
 0
 0
 0
 0
 0
 0
 9.96849954145E-06
 0
 0
 0
 0
 0

 4
 8
 8
 8
 8
 8
 8
 8
 8
 8
 8
 1
 1
 1
 1
 1
 1

 7.562472599741E-04
 2.1013747414748E-03
 3.281688179956E-04
 .000524471622738
 3.7173132797841E-03
 2.9646605190841E-03
 1.6016016015963E-03
 1.6758568493938E-03
 7.792207792211E-04
 1.0338588782632E-03
 .000688223217619
 7.476374656083E-04
 .005944750810844
 .005641606568043
 .000749164178771
 6.571421617544E-04
 2.132483332963E-04
 2.170794321958E-04
 .001415701415705
 .001094251531953

 58.0434782608692
 165.547368421078
 21.9696969696977
 24.7872340425523
 279.48275862075
 104.852760736142
 99.8500000000017
 100.532258064559
 60.8611111111105
 86.395833333334
 59
 69.240000000003
 402.010130246119
 267.56461232606
 65.0298507462713
 43.0000000000061
 17.1052631578931
 19.3478260869611
 89.0454545455109
 73.8761904761712

 .000690486628672
 .00195759646969
 .000258557250542
 .0003236101502
 .00332208169544
 .00232807697204
 .00145478812145
 .0015001621797
 .000714285714286
 .000980012061687
 .000688223217619
 .000717731966984
 .00424133451483
 .00366760506511
 .000737982623864
 .000584126366004
 .000202024736807
 .000198202959831
 .00112612612613
 .000896244111885

 63
 177
 26
 29
 311
 128
 109
 111
 66
 91
 59
 72
 493
 327
 66
 48
 18
 21
 105
 86

 6.57606313021E-05
 .000121658537664
 6.96115674536E-05
 .000200861472538
 .000181592890104
 .000163692912097
 .000133466800133
 .000162179695102
 5.41125541126E-05
 2.15387266305E-05
 0
 2.99054986243E-05
 .00160017894474
 .00186184232663
 .000011181554907
 3.65078978752E-05
 0
 1.88764723648E-05
 .000289575289575
 .000198007420068

 6
 11
 7
 18
 17
 9
 10
 12
 5
 2
 2
 3
 186
 166
 1
 3
 3
 2
 27
 19

 0
 2.21197341208E-05
 0
 0
 5.34096735601E-05
 1.81881013441E-05
 1.33466800133E-05
 0
 1.08225108225E-05
 3.23080899457E-05
 0
 0
 .000103237351274
 .000112159176303

 0
 2
 2
 2
 5
 1
 1
 1
 1
 3
 3
 3
 12
 10

 0
 0
 0
 0
 .00016022902068
 .000454702533603
 0
 1.35149745918E-05
 0
 0
 0
 0
 0
 0
 0
 3.65078978752E-05
 1.12235964893E-05

 0
 0
 0
 0
 15
 25
 25
 1
 1
 1
 1
 1
 1
 1
 1
 3
 1

 0
 1.10598670604E-05
 0
 0
 .00010681934712
 1.81881013441E-05
 0
 0
 0
 0
 0
 0
 6.88249008491E-05
 2.24318352606E-05
 .000011181554907
 0
 0
 0
 .000010725010725
 2.08428863229E-05

 0
 1
 1
 1
 10
 1
 1
 1
 1
 1
 1
 1
 8
 2
 1
 1
 1
 1
 1
 2

 0
 1.10598670604E-05
 0
 0
 .00010681934712
 1.81881013441E-05
 0
 0
 0
 0
 0
 0
 6.88249008491E-05
 2.24318352606E-05
 .000011181554907
 0
 0
 0
 .000010725010725
 2.08428863229E-05

 0
 1
 1
 1
 10
 1
 1
 1
 1
 1
 1
 1
 8
 2
 1
 1
 1
 1
 1
 2

 0
 1.10598670604E-05
 0
 0
 .00010681934712
 1.81881013441E-05
 0
 0
 0
 0
 0
 0
 6.88249008491E-05
 2.24318352606E-05
 .000011181554907
 0
 0
 0
 .000010725010725
 2.08428863229E-05

 0
 1
 1
 1
 10
 1
 1
 1
 1
 1
 1
 1
 8
 2
 1
 1
 1
 1
 1
 2

 .006499342393683
 .005861729542008
 3.5601344497748E-03
 .003671301359163
 9.3146470688701E-03
 8.8394172532364E-03
 1.08108108108201E-02
 9.3793923667392E-03
 .006266233766235
 6.8708537951272E-03
 .006357316162742
 .005073966266594
 .01147655221659
 8.7259839163803E-03
 .003041382934706
 .002969309027191
 .00620664885856
 .006578450619147
 .011422136422135
 1.08487223310688E-02

 484.278246205749
 285.135849056601
 220.631284916245
 193.936170212754
 475.713302752291
 273.127572016383
 522.958024691268
 460.227665706128
 332.796200345325
 342.485893416855
 396.009174311914
 330.996070726839
 605.632683658034
 301.257069408631
 120.904411764701
 110.959016393449
 280.681735985558
 375.051649928386
 510.327699530524
 495.39000960617

 .006499342393683
 .005861729542008
 3.5601344497748E-03
 .003671301359163
 9.3146470688701E-03
 8.8394172532364E-03
 1.08108108108201E-02
 9.3793923667392E-03
 .006266233766235
 6.8708537951272E-03
 .006357316162742
 .005073966266594
 .01147655221659
 8.7259839163803E-03
 .003041382934706
 .002969309027191
 .00620664885856
 .006578450619147
 .011422136422135
 1.08487223310688E-02

 484.278246205749
 285.135849056601
 220.631284916245
 193.936170212754
 475.713302752291
 273.127572016383
 522.958024691268
 460.227665706128
 332.796200345325
 342.485893416855
 396.009174311914
 330.996070726839
 605.632683658034
 301.257069408631
 120.904411764701
 110.959016393449
 280.681735985558
 375.051649928386
 510.327699530524
 495.39000960617

 .00585269618588
 .0039373126735
 .00267507309215
 .00271162987926
 .00617415826354
 .00603844964624
 .00835502168836
 .00739269110174
 .00436147186147
 .00448005513914
 .00536580813736
 .0040173053152
 .00222820616499
 .00253479738445
 .00156541768698
 .00144814661572
 .00226716649083
 .00216135608577
 .00507293007293
 .00552336487557

 534
 356
 269
 243
 578
 332
 626
 547
 403
 416
 460
 403
 259
 226
 140
 119
 202
 229
 473
 530

 .00585269618588
 .0039373126735
 .00267507309215
 .00271162987926
 .00617415826354
 .00603844964624
 .00835502168836
 .00739269110174
 .00436147186147
 .00448005513914
 .00536580813736
 .0040173053152
 .00222820616499
 .00253479738445
 .00156541768698
 .00144814661572
 .00226716649083
 .00216135608577
 .00507293007293
 .00552336487557

 534
 356
 269
 243
 578
 332
 626
 547
 403
 416
 460
 403
 259
 226
 140
 119
 202
 229
 473
 530

 .00585269618588
 .0039373126735
 .00267507309215
 .00271162987926
 .00617415826354
 .00603844964624
 .00835502168836
 .00739269110174
 .00436147186147
 .00448005513914
 .00536580813736
 .0040173053152
 .00222820616499
 .00253479738445
 .00156541768698
 .00144814661572
 .00226716649083
 .00216135608577
 .00507293007293
 .00552336487557

 534
 356
 269
 243
 578
 332
 626
 547
 403
 416
 460
 403
 259
 226
 140
 119
 202
 229
 473
 530

 .000646646207803
 .001924416868508
 8.850613576248E-04
 .000959671479903
 3.1404888053301E-03
 2.8009676069964E-03
 2.4557891224601E-03
 1.9867012649992E-03
 .001904761904765
 2.3907986559872E-03
 .000991508025382
 .001056660951394
 .0092483460516
 6.1911865319303E-03
 .001475965247726
 .001521162411471
 .00393948236773
 .004417094533377
 .006349206349205
 5.3253574554988E-03

 34.2542372881356
 140.149425287316
 74.4382022471967
 55.302325581392
 274.619047619045
 146.207792207799
 172.391304347835
 137.340136054419
 172.04545454546
 204.72972972974
 49.7058823529516
 57.2452830188738
 689.146976744011
 332.06884057956
 100.651515151533
 103.304000000014
 325.962962962994
 446.517094017066
 540.152027027047
 459.493150684952

 .000460324419114
 .00171427939436
 .000805505280535
 .000736492065972
 .00303366945821
 .00272821520162
 .00237570904238
 .00191912639204
 .00188311688312
 .00229387438615
 .000699888017917
 .000677857968819
 .00708036167485
 .00449758296975
 .0012746972594
 .00137513081997
 .00379357561337
 .00431327393537
 .00605963105963
 .00504397849014

 42
 155
 81
 66
 284
 150
 178
 142
 174
 213
 60
 68
 823
 401
 114
 113
 338
 457
 565
 484

 .000460324419114
 .00171427939436
 .000805505280535
 .000736492065972
 .00303366945821
 .00272821520162
 .00237570904238
 .00191912639204
 .00188311688312
 .00229387438615
 .000699888017917
 .000677857968819
 .00708036167485
 .00449758296975
 .0012746972594
 .00137513081997
 .00379357561337
 .00431327393537
 .00605963105963
 .00504397849014

 42
 155
 81
 66
 284
 150
 178
 142
 174
 213
 60
 68
 823
 401
 114
 113
 338
 457
 565
 484

 .000175361683472
 .000210137474148
 7.95560770898E-05
 .000223179413931
 9.61374124081E-05
 7.27524053764E-05
 8.00800800801E-05
 6.75748729592E-05
 .000021645021645
 9.69242698372E-05
 .000291620007465
 .000378802982575
 .00216798437675
 .00168238764455
 .000190086433419
 .000146031591501
 .00014590675436
 .000103820598007
 .000246675246675
 .000239693192713

 16
 19
 8
 20
 9
 4
 6
 5
 2
 9
 25
 38
 252
 150
 17
 12
 13
 11
 23
 23

 .000175361683472
 .000210137474148
 7.95560770898E-05
 .000223179413931
 9.61374124081E-05
 7.27524053764E-05
 8.00800800801E-05
 6.75748729592E-05
 .000021645021645
 9.69242698372E-05
 .000291620007465
 .000378802982575
 .00216798437675
 .00168238764455
 .000190086433419
 .000146031591501
 .00014590675436
 .000103820598007
 .000246675246675
 .000239693192713

 16
 19
 8
 20
 9
 4
 6
 5
 2
 9
 25
 38
 252
 150
 17
 12
 13
 11
 23
 23

 .000010960105217
 0
 0
 0
 .000010681934712
 0
 0
 0
 0
 0
 0
 0
 0
 1.12159176303E-05
 .000011181554907
 0
 0
 0
 .0000429000429
 4.16857726458E-05

 1
 1
 1
 1
 1
 1
 1
 1
 1
 1
 1
 1
 1
 1
 1
 1
 1
 1
 4
 4

 .000010960105217
 0
 0
 0
 .000010681934712
 0
 0
 0
 0
 0
 0
 0
 0
 1.12159176303E-05
 .000011181554907
 0
 0
 0
 .0000429000429
 4.16857726458E-05

 1
 1
 1
 1
 1
 1
 1
 1
 1
 1
 1
 1
 1
 1
 1
 1
 1
 1
 4
 4

 .000591845681719
 .000298616410631
 0
 1.11589706965E-05
 .000010681934712
 0
 .0002002002002
 .00014866472051
 .00030303030303
 .00020461790299
 8.16536020903E-05
 9.96849954145E-06
 0
 0
 0
 .000048677197167
 0
 0
 .00002145002145
 2.08428863229E-05

 54
 27
 27
 1
 1
 1
 15
 11
 28
 19
 7
 1
 1
 1
 1
 4
 4
 4
 2
 2

 .000591845681719
 .000298616410631
 0
 1.11589706965E-05
 .000010681934712
 0
 .0002002002002
 .00014866472051
 .00030303030303
 .00020461790299
 8.16536020903E-05
 9.96849954145E-06
 0
 0
 0
 .000048677197167
 0
 0
 .00002145002145
 2.08428863229E-05

 54
 27
 27
 1
 1
 1
 15
 11
 28
 19
 7
 1
 1
 1
 1
 4
 4
 4
 2
 2

 .000591845681719
 .000298616410631
 0
 1.11589706965E-05
 .000010681934712
 0
 .0002002002002
 .00014866472051
 .00030303030303
 .00020461790299
 8.16536020903E-05
 9.96849954145E-06
 0
 0
 0
 .000048677197167
 0
 0
 .00002145002145
 2.08428863229E-05

 54
 27
 27
 1
 1
 1
 15
 11
 28
 19
 7
 1
 1
 1
 1
 4
 4
 4
 2
 2

 .000591845681719
 .000298616410631
 0
 1.11589706965E-05
 .000010681934712
 0
 .0002002002002
 .00014866472051
 .00030303030303
 .00020461790299
 8.16536020903E-05
 9.96849954145E-06
 0
 0
 0
 .000048677197167
 0
 0
 .00002145002145
 2.08428863229E-05

 54
 27
 27
 1
 1
 1
 15
 11
 28
 19
 7
 1
 1
 1
 1
 4
 4
 4
 2
 2

 .000591845681719
 .000298616410631
 0
 1.11589706965E-05
 .000010681934712
 0
 .0002002002002
 .00014866472051
 .00030303030303
 .00020461790299
 8.16536020903E-05
 9.96849954145E-06
 0
 0
 0
 .000048677197167
 0
 0
 .00002145002145
 2.08428863229E-05

 54
 27
 27
 1
 1
 1
 15
 11
 28
 19
 7
 1
 1
 1
 1
 4
 4
 4
 2
 2

 .000021920210434
 6.63592023624E-05
 0
 0
 .000010681934712
 5.45643040323E-05
 1.33466800133E-05
 1.35149745918E-05
 .000119047619048
 .000118462996468
 1.16648002986E-05

 2
 6
 6
 6
 1
 3
 1
 1
 11
 11
 1

 .000021920210434
 6.63592023624E-05
 0
 0
 .000010681934712
 5.45643040323E-05
 1.33466800133E-05
 1.35149745918E-05
 .000119047619048
 .000118462996468
 1.16648002986E-05

 2
 6
 6
 6
 1
 3
 1
 1
 11
 11
 1

 .000021920210434
 6.63592023624E-05
 0
 0
 .000010681934712
 5.45643040323E-05
 1.33466800133E-05
 1.35149745918E-05
 .000119047619048
 .000118462996468
 1.16648002986E-05

 2
 6
 6
 6
 1
 3
 1
 1
 11
 11
 1

 .000021920210434
 6.63592023624E-05
 0
 0
 .000010681934712
 5.45643040323E-05
 1.33466800133E-05
 1.35149745918E-05
 .000119047619048
 .000118462996468
 1.16648002986E-05

 2
 6
 6
 6
 1
 3
 1
 1
 11
 11
 1

 .000021920210434
 6.63592023624E-05
 0
 0
 .000010681934712
 5.45643040323E-05
 1.33466800133E-05
 1.35149745918E-05
 .000119047619048
 .000118462996468
 1.16648002986E-05

 2
 6
 6
 6
 1
 3
 1
 1
 11
 11
 1

 1.83800964489651E-02
 2.98284614618752E-02
 .015036098569939
 1.57676255941983E-02
 .027965305076085
 .025226896564221
 3.63163163162918E-02
 3.64498864742126E-02
 .029199134199113
 2.84311191522253E-02
 2.72839678984201E-02
 2.74532477371943E-02
 3.89118783176031E-02
 4.16222703260673E-02
 1.47708340321663E-02
 1.51264390196394E-02
 9.6186221912954E-03
 1.81874811235005E-02
 .033504933504937
 2.91175121930593E-02

 885.00715563604
 1156.83908045935
 1018.77116402099
 952.013446567107
 1441.27807486643
 660.476568132368
 1697.81293641996
 1773.65776789033
 1431.95478131893
 1286.41212121235
 1388.80504489032
 1702.40232389349
 2211.99270395754
 1476.48908649954
 1016.6411809234
 850.127916331644
 563.212368728047
 1152.96782563549
 1964.37323943634
 1785.77022190393

 1.83800964489651E-02
 2.98284614618752E-02
 .015036098569939
 1.57676255941983E-02
 .027965305076085
 .025226896564221
 3.63163163162918E-02
 3.64498864742126E-02
 .029199134199113
 2.84311191522253E-02
 2.72839678984201E-02
 2.74532477371943E-02
 3.89118783176031E-02
 4.16222703260673E-02
 1.47708340321663E-02
 1.51264390196394E-02
 9.6186221912954E-03
 1.81874811235005E-02
 .033504933504937
 2.91175121930593E-02

 885.00715563604
 1156.83908045935
 1018.77116402099
 952.013446567107
 1441.27807486643
 660.476568132368
 1697.81293641996
 1773.65776789033
 1431.95478131893
 1286.41212121235
 1388.80504489032
 1702.40232389349
 2211.99270395754
 1476.48908649954
 1016.6411809234
 850.127916331644
 563.212368728047
 1152.96782563549
 1964.37323943634
 1785.77022190393

 .001161771153
 .000442394682416
 .0006861711649
 .000747651036668
 .000715689625705
 .000563831141667
 .000920920920921
 .000851443399286
 .000411255411255
 .000387697079349
 .000898189622994
 .000588141472945
 .000292505828609
 .000336477528909
 .000391354421746
 .000352909679461
 .000179577543828
 .000103820598007
 .0012012012012
 .00102130142982

 106
 40
 69
 67
 67
 31
 69
 63
 38
 36
 77
 59
 34
 30
 35
 29
 16
 11
 112
 98

 .001161771153
 .000442394682416
 .0006861711649
 .000747651036668
 .000715689625705
 .000563831141667
 .000920920920921
 .000851443399286
 .000411255411255
 .000387697079349
 .000898189622994
 .000588141472945
 .000292505828609
 .000336477528909
 .000391354421746
 .000352909679461
 .000179577543828
 .000103820598007
 .0012012012012
 .00102130142982

 106
 40
 69
 67
 67
 31
 69
 63
 38
 36
 77
 59
 34
 30
 35
 29
 16
 11
 112
 98

 .001161771153
 .000442394682416
 .0006861711649
 .000747651036668
 .000715689625705
 .000563831141667
 .000920920920921
 .000851443399286
 .000411255411255
 .000387697079349
 .000898189622994
 .000588141472945
 .000292505828609
 .000336477528909
 .000391354421746
 .000352909679461
 .000179577543828
 .000103820598007
 .0012012012012
 .00102130142982

 106
 40
 69
 67
 67
 31
 69
 63
 38
 36
 77
 59
 34
 30
 35
 29
 16
 11
 112
 98

 .014182376150853
 2.27280268090972E-02
 .014131148193042
 .014930702791958
 .024130490514468
 .020698059329542
 3.08308308308041E-02
 3.13006811547174E-02
 .024999999999978
 2.37679848367306E-02
 2.31779581933091E-02
 2.37948084054864E-02
 .036459991224848
 .038739779495078
 1.42676640613503E-02
 1.46153284493854E-02
 9.4165974544889E-03
 1.80553458169462E-02
 .031241956241957
 2.71791237650313E-02

 1081.48686244257
 1344.01995133788
 1080.32160450386
 1001.97458893824
 1635.63302346181
 769.346221440955
 1948.65541125517
 2021.41278065647
 1618.81818181772
 1467.86905301329
 1598.20483140364
 1925.20025136198
 2345.72581406342
 1571.30110017367
 1051.45611285254
 879.016652789578
 574.985697258555
 1161.33768949276
 2098.98970133869
 1906.47776073606

 .012954844366551
 1.78727451695812E-02
 .0122516358718
 .0128439752717
 .020477268842956
 1.67512413378764E-02
 2.82816149482534E-02
 2.91923451183918E-02
 .020562770562745
 1.89002326182457E-02
 2.07400149308959E-02
 2.12927150205829E-02
 .025671688016743
 .024304893504873
 .0129146959176
 .012388346679
 7.7442815775878E-03
 .013638251283577
 .026297726297725
 2.30834966025843E-02

 1176.01522842641
 1610.01113861386
 1232
 1151
 1891.17631716227
 913.034744842553
 2111.01510146295
 2158.00092592593
 1896.00421052632
 1749.01025641026
 1772.01012373452
 2132.00374531836
 2970.03284182306
 2149.07475772956
 1155
 1018
 684.026086956519
 1431.06782006919
 2450.00081566069
 2209.00812641084

 .012954844366551
 1.78727451695812E-02
 .0122516358718
 .0128439752717
 .020477268842956
 1.67512413378764E-02
 2.82816149482534E-02
 2.91923451183918E-02
 .020562770562745
 1.89002326182457E-02
 2.07400149308959E-02
 2.12927150205829E-02
 .025671688016743
 .024304893504873
 .0129146959176
 .012388346679
 7.7442815775878E-03
 .013638251283577
 .026297726297725
 2.30834966025843E-02

 1176.01522842641
 1610.01113861386
 1232
 1151
 1891.17631716227
 913.034744842553
 2111.01510146295
 2158.00092592593
 1896.00421052632
 1749.01025641026
 1772.01012373452
 2132.00374531836
 2970.03284182306
 2149.07475772956
 1155
 1018
 684.026086956519
 1431.06782006919
 2450.00081566069
 2209.00812641084

 .000142481367821
 .000331796011812
 .000218779211997
 .000569107505524
 .000470005127329
 9.09405067205E-05
 2.66933600267E-05
 1.35149745918E-05
 .000162337662338
 8.61549065219E-05
 .000221631205674
 .00013955899358
 .000997961062312
 .00431812828767
 .000324265092304
 .000438094774503
 7.85651754248E-05
 9.43823618242E-06
 .000514800514801
 .000458543499104

 13
 30
 22
 51
 44
 5
 2
 1
 15
 8
 19
 14
 116
 385
 29
 36
 7
 1
 48
 44

 .000142481367821
 .000331796011812
 .000218779211997
 .000569107505524
 .000470005127329
 9.09405067205E-05
 2.66933600267E-05
 1.35149745918E-05
 .000162337662338
 8.61549065219E-05
 .000221631205674
 .00013955899358
 .000997961062312
 .00431812828767
 .000324265092304
 .000438094774503
 7.85651754248E-05
 9.43823618242E-06
 .000514800514801
 .000458543499104

 13
 30
 22
 51
 44
 5
 2
 1
 15
 8
 19
 14
 116
 385
 29
 36
 7
 1
 48
 44

 .00105217010083
 .0044128869571
 .00125300821416
 .00127212265941
 .00274525722099
 .00334661064731
 .00222889556223
 .00191912639204
 .00399350649351
 .00442620832256
 .00178471444569
 .00199369990829
 .00886120598433
 .00879327942216
 .000916887502376
 .00163068610509
 .00108868885946
 .00364315916641
 .00335692835693
 .00282421109675

 96
 399
 126
 114
 257
 184
 167
 142
 369
 411
 153
 200
 1030
 784
 82
 134
 97
 386
 313
 271

 .00105217010083
 .0044128869571
 .00125300821416
 .00127212265941
 .00274525722099
 .00334661064731
 .00222889556223
 .00191912639204
 .00399350649351
 .00442620832256
 .00178471444569
 .00199369990829
 .00886120598433
 .00879327942216
 .000916887502376
 .00163068610509
 .00108868885946
 .00364315916641
 .00335692835693
 .00282421109675

 96
 399
 126
 114
 257
 184
 167
 142
 369
 411
 153
 200
 1030
 784
 82
 134
 97
 386
 313
 271

 .000032880315651
 9.95388035436E-05
 .000278446269814
 .000133907648359
 .000416595453769
 .000491078736291
 .000186853520187
 .000162179695102
 .000281385281385
 .000355388989403
 .000408268010452
 .000358865983492
 .000455964968125
 .000302829776018
 2.23631098141E-05
 1.21692992917E-05
 .000224471929785
 .000641800060405
 .000847275847276
 .000604443703364

 3
 9
 28
 12
 39
 27
 14
 12
 26
 33
 35
 36
 53
 27
 2
 1
 20
 68
 79
 58

 .000032880315651
 9.95388035436E-05
 .000278446269814
 .000133907648359
 .000416595453769
 .000491078736291
 .000186853520187
 .000162179695102
 .000281385281385
 .000355388989403
 .000408268010452
 .000358865983492
 .000455964968125
 .000302829776018
 2.23631098141E-05
 1.21692992917E-05
 .000224471929785
 .000641800060405
 .000847275847276
 .000604443703364

 3
 9
 28
 12
 39
 27
 14
 12
 26
 33
 35
 36
 53
 27
 2
 1
 20
 68
 79
 58

 0
 1.10598670604E-05
 0
 0
 0
 0
 0
 0
 0
 0
 0
 0
 .00011184046388
 7.85114234121E-05

 0
 1
 1
 1
 1
 1
 1
 1
 1
 1
 1
 1
 13
 7

 0
 1.10598670604E-05
 0
 0
 0
 0
 0
 0
 0
 0
 0
 0
 .00011184046388
 7.85114234121E-05

 0
 1
 1
 1
 1
 1
 1
 1
 1
 1
 1
 1
 13
 7

 0
 0
 .000129278625271
 .000111589706965
 .000021363869424
 0
 .000106773440107
 1.35149745918E-05
 0
 0
 2.33296005972E-05
 9.96849954145E-06
 .000301108941215
 3.36477528909E-05
 8.94524392562E-05
 .000133862292209
 .000269366315742
 .000103820598007
 .000225225225225
 .000208428863229

 0
 0
 13
 10
 2
 2
 8
 1
 1
 1
 2
 1
 35
 3
 8
 11
 24
 11
 21
 20

 0
 0
 .000129278625271
 .000111589706965
 .000021363869424
 0
 .000106773440107
 1.35149745918E-05
 0
 0
 2.33296005972E-05
 9.96849954145E-06
 .000301108941215
 3.36477528909E-05
 8.94524392562E-05
 .000133862292209
 .000269366315742
 .000103820598007
 .000225225225225
 .000208428863229

 0
 0
 13
 10
 2
 2
 8
 1
 1
 1
 2
 1
 35
 3
 8
 11
 24
 11
 21
 20

 0
 0
 0
 0
 0
 1.81881013441E-05
 0
 0
 0
 0
 0
 0
 .000060221788243
 .000908489328054
 0
 1.21692992917E-05
 1.12235964893E-05
 1.88764723648E-05

 0
 0
 0
 0
 0
 1
 1
 1
 1
 1
 1
 1
 7
 81
 81
 1
 1
 2

 0
 0
 0
 0
 0
 1.81881013441E-05
 0
 0
 0
 0
 0
 0
 .000060221788243
 .000908489328054
 0
 1.21692992917E-05
 1.12235964893E-05
 1.88764723648E-05

 0
 0
 0
 0
 0
 1
 1
 1
 1
 1
 1
 1
 7
 81
 81
 1
 1
 2

 3.0359491451121E-03
 .006658039970362
 .000218779211997
 8.92717655723E-05
 .003119124935912
 .003965006093012
 4.5645645645667E-03
 4.2977619202092E-03
 .00378787878788
 4.2754372361457E-03
 .003207820082117
 .003070297858763
 2.15938126414614E-03
 2.5460133020803E-03
 .00011181554907
 .000158200890793
 2.24471929785E-05
 2.83147085473E-05
 .00106177606178
 .000917086998208

 265.259927797831
 592.083056478406
 22
 8
 253.020547945252
 181.669724770635
 332.146198830414
 308.157232704407
 350
 391.045340050377
 243.101818181767
 290.525974025969
 249.007968127492
 225.008810572688
 10
 13
 2
 3
 99
 88

 .00297018851381
 .00660274063506
 .000218779211997
 8.92717655723E-05
 .00289480430696
 .00360124406613
 .0044978311645
 .00423018704725
 .00378787878788
 .0042431291462
 .00300951847704
 .00298058136289
 .00215077815154
 .00253479738445
 .00011181554907
 .000158200890793
 2.24471929785E-05
 2.83147085473E-05
 .00106177606178
 .000917086998208

 271
 597
 22
 8
 271
 198
 337
 313
 350
 394
 258
 299
 250
 226
 10
 13
 2
 3
 99
 88

 .00297018851381
 .00660274063506
 .000218779211997
 8.92717655723E-05
 .00289480430696
 .00360124406613
 .0044978311645
 .00423018704725
 .00378787878788
 .0042431291462
 .00300951847704
 .00298058136289
 .00215077815154
 .00253479738445
 .00011181554907
 .000158200890793
 2.24471929785E-05
 2.83147085473E-05
 .00106177606178
 .000917086998208

 271
 597
 22
 8
 271
 198
 337
 313
 350
 394
 258
 299
 250
 226
 10
 13
 2
 3
 99
 88

 6.57606313021E-05
 .000055299335302
 0
 0
 .000224320628952
 .000363762026882
 6.67334000667E-05
 6.75748729592E-05
 0
 3.23080899457E-05
 .000198301605077
 .000089716495873
 8.60311260614E-06
 1.12159176303E-05

 6
 5
 5
 5
 21
 20
 5
 5
 5
 3
 17
 9
 1
 1

 6.57606313021E-05
 .000055299335302
 0
 0
 .000224320628952
 .000363762026882
 6.67334000667E-05
 6.75748729592E-05
 0
 3.23080899457E-05
 .000198301605077
 .000089716495873
 8.60311260614E-06
 1.12159176303E-05

 6
 5
 5
 5
 21
 20
 5
 5
 5
 3
 17
 9
 1
 1

 0
 .000055299335302
 4.97225481811E-05
 3.34769120896E-05
 .000128183216544
 .000109128608065
 8.00800800801E-05
 .000135149745918
 5.41125541126E-05
 .000150771086413
 .000174972004479
 .000408708481199
 .000817295697583
 .000650523222557
 5.59077745351E-05
 8.51850950422E-05
 .000516285438506
 .000405844155844
 .0001716001716
 .000166743090583

 0
 5
 5
 3
 12
 6
 6
 10
 5
 14
 15
 41
 95
 58
 5
 7
 46
 43
 16
 16

 0
 .000055299335302
 4.97225481811E-05
 3.34769120896E-05
 .000128183216544
 .000109128608065
 8.00800800801E-05
 .000135149745918
 5.41125541126E-05
 .000150771086413
 .000174972004479
 .000408708481199
 .000817295697583
 .000650523222557
 5.59077745351E-05
 8.51850950422E-05
 .000516285438506
 .000405844155844
 .0001716001716
 .000166743090583

 0
 5
 5
 3
 12
 6
 6
 10
 5
 14
 15
 41
 95
 58
 5
 7
 46
 43
 16
 16

 0
 .000055299335302
 4.97225481811E-05
 3.34769120896E-05
 .000128183216544
 .000109128608065
 8.00800800801E-05
 .000135149745918
 5.41125541126E-05
 .000150771086413
 .000174972004479
 .000408708481199
 .000817295697583
 .000650523222557
 5.59077745351E-05
 8.51850950422E-05
 .000516285438506
 .000405844155844
 .0001716001716
 .000166743090583

 0
 5
 5
 3
 12
 6
 6
 10
 5
 14
 15
 41
 95
 58
 5
 7
 46
 43
 16
 16

 0
 .000055299335302
 4.97225481811E-05
 3.34769120896E-05
 .000128183216544
 .000109128608065
 8.00800800801E-05
 .000135149745918
 5.41125541126E-05
 .000150771086413
 .000174972004479
 .000408708481199
 .000817295697583
 .000650523222557
 5.59077745351E-05
 8.51850950422E-05
 .000516285438506
 .000405844155844
 .0001716001716
 .000166743090583

 0
 5
 5
 3
 12
 6
 6
 10
 5
 14
 15
 41
 95
 58
 5
 7
 46
 43
 16
 16

 0
 .000055299335302
 4.97225481811E-05
 3.34769120896E-05
 .000128183216544
 .000109128608065
 8.00800800801E-05
 .000135149745918
 5.41125541126E-05
 .000150771086413
 .000174972004479
 .000408708481199
 .000817295697583
 .000650523222557
 5.59077745351E-05
 8.51850950422E-05
 .000516285438506
 .000405844155844
 .0001716001716
 .000166743090583

 0
 5
 5
 3
 12
 6
 6
 10
 5
 14
 15
 41
 95
 58
 5
 7
 46
 43
 16
 16

 0
 0
 9.94450963623E-06
 4.46358827862E-05
 5.34096735601E-05
 1.81881013441E-05
 .00004004004004
 1.35149745918E-05
 0
 1.07693633152E-05
 1.16648002986E-05
 9.96849954145E-06
 2.58093378184E-05
 2.24318352606E-05
 2.23631098141E-05
 0
 0
 0
 .00002145002145

 0
 0
 1
 4
 5
 1
 3
 1
 1
 1
 1
 1
 3
 2
 2
 2
 2
 2
 2

 0
 0
 9.94450963623E-06
 4.46358827862E-05
 5.34096735601E-05
 1.81881013441E-05
 .00004004004004
 1.35149745918E-05
 0
 1.07693633152E-05
 1.16648002986E-05
 9.96849954145E-06
 2.58093378184E-05
 2.24318352606E-05
 2.23631098141E-05
 0
 0
 0
 .00002145002145

 0
 0
 1
 4
 5
 1
 3
 1
 1
 1
 1
 1
 3
 2
 2
 2
 2
 2
 2

 0
 0
 9.94450963623E-06
 4.46358827862E-05
 5.34096735601E-05
 1.81881013441E-05
 .00004004004004
 1.35149745918E-05
 0
 1.07693633152E-05
 1.16648002986E-05
 9.96849954145E-06
 2.58093378184E-05
 2.24318352606E-05
 2.23631098141E-05
 0
 0
 0
 .00002145002145

 0
 0
 1
 4
 5
 1
 3
 1
 1
 1
 1
 1
 3
 2
 2
 2
 2
 2
 2

 0
 0
 9.94450963623E-06
 4.46358827862E-05
 5.34096735601E-05
 1.81881013441E-05
 .00004004004004
 1.35149745918E-05
 0
 1.07693633152E-05
 1.16648002986E-05
 9.96849954145E-06
 2.58093378184E-05
 2.24318352606E-05
 2.23631098141E-05
 0
 0
 0
 .00002145002145

 0
 0
 1
 4
 5
 1
 3
 1
 1
 1
 1
 1
 3
 2
 2
 2
 2
 2
 2

 0
 0
 9.94450963623E-06
 4.46358827862E-05
 5.34096735601E-05
 1.81881013441E-05
 .00004004004004
 1.35149745918E-05
 0
 1.07693633152E-05
 1.16648002986E-05
 9.96849954145E-06
 2.58093378184E-05
 2.24318352606E-05
 2.23631098141E-05
 0
 0
 0
 .00002145002145

 0
 0
 1
 4
 5
 1
 3
 1
 1
 1
 1
 1
 3
 2
 2
 2
 2
 2
 2

 0
 0
 0
 0
 .000010681934712
 0
 8.00800800801E-05
 6.75748729592E-05
 0
 2.15387266305E-05
 6.99888017917E-05
 2.99054986243E-05

 0
 0
 0
 0
 1
 1
 6
 5
 5
 2
 6
 3

 0
 0
 0
 0
 .000010681934712
 0
 8.00800800801E-05
 6.75748729592E-05
 0
 2.15387266305E-05
 6.99888017917E-05
 2.99054986243E-05

 0
 0
 0
 0
 1
 1
 6
 5
 5
 2
 6
 3

 0
 0
 0
 0
 .000010681934712
 0
 8.00800800801E-05
 6.75748729592E-05
 0
 2.15387266305E-05
 6.99888017917E-05
 2.99054986243E-05

 0
 0
 0
 0
 1
 1
 6
 5
 5
 2
 6
 3

 0
 0
 0
 0
 .000010681934712
 0
 8.00800800801E-05
 6.75748729592E-05
 0
 2.15387266305E-05
 6.99888017917E-05
 2.99054986243E-05

 0
 0
 0
 0
 1
 1
 6
 5
 5
 2
 6
 3

 0
 0
 0
 0
 .000010681934712
 0
 8.00800800801E-05
 6.75748729592E-05
 0
 2.15387266305E-05
 6.99888017917E-05
 2.99054986243E-05

 0
 0
 0
 0
 1
 1
 6
 5
 5
 2
 6
 3

 1.3590530469091E-03
 7.410110930472E-04
 4.3755842399392E-03
 4.3408396009641E-03
 1.3031960348651E-03
 8.366526618293E-04
 2.8428428428473E-03
 3.6895880635722E-03
 1.1904761904765E-03
 1.4646334108729E-03
 4.1293393057113E-03
 3.59862833446125E-03
 1.93570033638254E-03
 2.1534561850183E-03
 1.2635157044942E-03
 1.1195755348415E-03
 2.5702035960374E-03
 1.9820295983084E-03
 1.5122265122261E-03
 1.4173162699563E-03

 40.4516129032356
 18.2835820895445
 189.072727272768
 154.264781491019
 35.8032786885372
 16.2608695652104
 94.7558685446808
 117.871794871765
 47.5272727272586
 50.2205882352874
 127.853107344617
 118.008310249255
 64.6622222222213
 58.7395833333336
 34.911504424776
 31.543478260872
 75.5152838427595
 88.4476190476304
 51.7092198581692
 49.6911764706128

 1.3590530469091E-03
 7.410110930472E-04
 4.3755842399392E-03
 4.3408396009641E-03
 1.3031960348651E-03
 8.366526618293E-04
 2.8428428428473E-03
 3.6895880635722E-03
 1.1904761904765E-03
 1.4646334108729E-03
 4.1293393057113E-03
 3.59862833446125E-03
 1.93570033638254E-03
 2.1534561850183E-03
 1.2635157044942E-03
 1.1195755348415E-03
 2.5702035960374E-03
 1.9820295983084E-03
 1.5122265122261E-03
 1.4173162699563E-03

 40.4516129032356
 18.2835820895445
 189.072727272768
 154.264781491019
 35.8032786885372
 16.2608695652104
 94.7558685446808
 117.871794871765
 47.5272727272586
 50.2205882352874
 127.853107344617
 118.008310249255
 64.6622222222213
 58.7395833333336
 34.911504424776
 31.543478260872
 75.5152838427595
 88.4476190476304
 51.7092198581692
 49.6911764706128

 .000197281893906
 .000309676277691
 .000318224308359
 .000424040886469
 .000331139976072
 .000272821520162
 .000427093760427
 .000567628932858
 .000357142857143
 .000506160075816
 .00093318402389
 .000807448462857
 .000765677021947
 .000785114234121
 .000313083537397
 .000146031591501
 .000280589912231
 .000396405919662
 .00032175032175
 .000333486181166

 18
 28
 32
 38
 31
 15
 32
 42
 33
 47
 80
 81
 89
 70
 28
 12
 25
 42
 30
 32

 .000197281893906
 .000309676277691
 .000318224308359
 .000424040886469
 .000331139976072
 .000272821520162
 .000427093760427
 .000567628932858
 .000357142857143
 .000506160075816
 .00093318402389
 .000807448462857
 .000765677021947
 .000785114234121
 .000313083537397
 .000146031591501
 .000280589912231
 .000396405919662
 .00032175032175
 .000333486181166

 18
 28
 32
 38
 31
 15
 32
 42
 33
 47
 80
 81
 89
 70
 28
 12
 25
 42
 30
 32

 .000197281893906
 .000309676277691
 .000318224308359
 .000424040886469
 .000331139976072
 .000272821520162
 .000427093760427
 .000567628932858
 .000357142857143
 .000506160075816
 .00093318402389
 .000807448462857
 .000765677021947
 .000785114234121
 .000313083537397
 .000146031591501
 .000280589912231
 .000396405919662
 .00032175032175
 .000333486181166

 18
 28
 32
 38
 31
 15
 32
 42
 33
 47
 80
 81
 89
 70
 28
 12
 25
 42
 30
 32

 .000197281893906
 .000309676277691
 .000318224308359
 .000424040886469
 .000331139976072
 .000272821520162
 .000427093760427
 .000567628932858
 .000357142857143
 .000506160075816
 .00093318402389
 .000807448462857
 .000765677021947
 .000785114234121
 .000313083537397
 .000146031591501
 .000280589912231
 .000396405919662
 .00032175032175
 .000333486181166

 18
 28
 32
 38
 31
 15
 32
 42
 33
 47
 80
 81
 89
 70
 28
 12
 25
 42
 30
 32

 1.1617711530031E-03
 4.313348153562E-04
 4.0573599315802E-03
 3.9167987144951E-03
 9.720560587931E-04
 5.638311416673E-04
 2.4157490824203E-03
 3.1219591307142E-03
 8.333333333335E-04
 9.584733350569E-04
 3.1961552818213E-03
 2.79117987160425E-03
 1.17002331443554E-03
 1.3683419508973E-03
 9.504321670972E-04
 9.735439433405E-04
 2.2896136838064E-03
 1.5856236786464E-03
 1.1904761904761E-03
 1.0838300887903E-03

 44.2641509434041
 11.3076923076933
 201.392156862781
 166.851851851853
 37.4395604395755
 16.870967741926
 105.850828729352
 131.666666666652
 53.7532467532279
 51.9213483145946
 141.824817518236
 128.714285714218
 48.7352941176411
 52.2786885245939
 37.1882352941159
 34.4750000000013
 81.7058823528977
 100.05952380955
 57.576576576588
 55.1346153846435

 8.76808417361E-05
 .000132718404725
 .00245629388015
 .00217599928583
 .000587506409161
 .00040013822957
 .00177510844178
 .00228403070602
 .000681818181818
 .000710777978806
 .00219298245614
 .001804298417
 .000524789868975
 .00072903464597
 .000458443751188
 .000450264073795
 .00132438438573
 .00120809423135
 .000815100815101
 .000760765350786

 8
 12
 247
 195
 55
 22
 133
 169
 63
 66
 188
 181
 61
 65
 41
 37
 118
 128
 76
 73

 8.76808417361E-05
 .000132718404725
 .00245629388015
 .00217599928583
 .000587506409161
 .00040013822957
 .00177510844178
 .00228403070602
 .000681818181818
 .000710777978806
 .00219298245614
 .001804298417
 .000524789868975
 .00072903464597
 .000458443751188
 .000450264073795
 .00132438438573
 .00120809423135
 .000815100815101
 .000760765350786

 8
 12
 247
 195
 55
 22
 133
 169
 63
 66
 188
 181
 61
 65
 41
 37
 118
 128
 76
 73

 8.76808417361E-05
 .000132718404725
 .00245629388015
 .00217599928583
 .000587506409161
 .00040013822957
 .00177510844178
 .00228403070602
 .000681818181818
 .000710777978806
 .00219298245614
 .001804298417
 .000524789868975
 .00072903464597
 .000458443751188
 .000450264073795
 .00132438438573
 .00120809423135
 .000815100815101
 .000760765350786

 8
 12
 247
 195
 55
 22
 133
 169
 63
 66
 188
 181
 61
 65
 41
 37
 118
 128
 76
 73

 .000449364313897
 1.658980059062E-04
 1.591121541802E-04
 1.450666190551E-04
 1.175012818321E-04
 5.45643040323E-05
 1.468134801463E-04
 3.108444156122E-04
 1.08225108225E-05
 8.61549065219E-05
 3.382792086603E-04
 5.0839347661425E-04
 1.9787158994154E-04
 1.233750939333E-04
 6.70893294422E-05
 7.30157957505E-05
 5.724034209524E-04
 2.548323769254E-04
 .00027885027885
 1.563216474213E-04

 33.6341463414634
 10.2000000000022
 8.37500000000581
 7.00000000000414
 6.27272727272874
 1
 9.18181818181818
 14.478260869564
 1
 4.99999999999884
 9.20689655172832
 19.1176470588331
 8.82608695652728
 3.36363636363636
 6
 3.33333333333333
 29.7843137254907
 9.5185185185173
 20.5384615384615
 9.9333333333309

 .000032880315651
 3.31796011812E-05
 4.97225481812E-05
 4.46358827861E-05
 .000032045804136
 1.81881013441E-05
 0
 2.70299491837E-05
 1.08225108225E-05
 0
 1.049832026875E-04
 1.894014912875E-04
 1.290466890924E-04
 8.97273410424E-05
 0
 .000048677197167
 7.85651754248E-05
 .000066067653277
 .000032175032175
 2.08428863229E-05

 3
 3
 2.5999999999996
 2.50000000000112
 1.66666666666667
 1
 1
 2
 1
 1
 5.88888888888995
 9.52631578947382
 10.2000000000056
 4
 4
 4
 7
 7
 1.66666666666667
 2

 .000032880315651
 3.31796011812E-05
 4.97225481812E-05
 4.46358827861E-05
 .000032045804136
 1.81881013441E-05
 0
 2.70299491837E-05
 1.08225108225E-05
 0
 1.049832026875E-04
 1.894014912875E-04
 1.290466890924E-04
 8.97273410424E-05
 0
 .000048677197167
 7.85651754248E-05
 .000066067653277
 .000032175032175
 2.08428863229E-05

 3
 3
 2.5999999999996
 2.50000000000112
 1.66666666666667
 1
 1
 2
 1
 1
 5.88888888888995
 9.52631578947382
 10.2000000000056
 4
 4
 4
 7
 7
 1.66666666666667
 2

 .000405523893029
 .000132718404725
 .000109389605999
 .000100430736269
 8.54554776961E-05
 1.81881013441E-05
 .000133466800133
 .000243269542653
 0
 6.46161798914E-05
 .000163307204181
 .000279117987161
 .000060221788243
 2.24318352606E-05
 6.70893294422E-05
 2.43385985835E-05
 5.61179824463E-05
 7.55058894594E-05
 .000246675246675
 .000125057317937

 37
 12
 11
 9
 8
 1
 10
 18
 18
 6
 14
 28
 7
 2
 6
 2
 5
 8
 23
 12

 .000405523893029
 .000132718404725
 .000109389605999
 .000100430736269
 8.54554776961E-05
 1.81881013441E-05
 .000133466800133
 .000243269542653
 0
 6.46161798914E-05
 .000163307204181
 .000279117987161
 .000060221788243
 2.24318352606E-05
 6.70893294422E-05
 2.43385985835E-05
 5.61179824463E-05
 7.55058894594E-05
 .000246675246675
 .000125057317937

 37
 12
 11
 9
 8
 1
 10
 18
 18
 6
 14
 28
 7
 2
 6
 2
 5
 8
 23
 12

 .000010960105217
 0
 0
 0
 0
 1.81881013441E-05
 1.33466800133E-05
 4.05449237755E-05
 0
 2.15387266305E-05
 6.99888017918E-05
 3.987399816575E-05
 8.60311260614E-06
 1.12159176303E-05
 0
 0
 4.377202630813E-04
 .000113258834189
 0
 1.04214431614E-05

 1
 1
 1
 1
 1
 1
 1
 1.66666666666749
 1.66666666666749
 2
 3
 2.49999999999937
 1
 1
 1
 1
 37.051282051279
 12
 12
 1

 .000010960105217
 0
 0
 0
 0
 1.81881013441E-05
 1.33466800133E-05
 4.05449237755E-05
 0
 2.15387266305E-05
 6.99888017918E-05
 3.987399816575E-05
 8.60311260614E-06
 1.12159176303E-05
 0
 0
 4.377202630813E-04
 .000113258834189
 0
 1.04214431614E-05

 1
 1
 1
 1
 1
 1
 1
 1.66666666666749
 1.66666666666749
 2
 3
 2.49999999999937
 1
 1
 1
 1
 37.051282051279
 12
 12
 1

 .00062472599737
 .000132718404725
 .00144195389725
 .00159573280961
 .000138865151256
 .000109128608065
 .000493827160494
 .000527084009082
 .000140692640693
 .000161540449729
 .000664893617021
 .00047848797799
 .000447361855519
 .000515932210994
 .000424899086467
 .000450264073795
 .000392825877124
 .000122697070371
 9.65250965251E-05
 .000166743090583

 57
 12
 145
 143
 13
 6
 37
 39
 13
 15
 57
 48
 52
 46
 38
 37
 35
 13
 9
 16

 .00062472599737
 .000132718404725
 .00144195389725
 .00159573280961
 .000138865151256
 .000109128608065
 .000493827160494
 .000527084009082
 .000140692640693
 .000161540449729
 .000664893617021
 .00047848797799
 .000447361855519
 .000515932210994
 .000424899086467
 .000450264073795
 .000392825877124
 .000122697070371
 9.65250965251E-05
 .000166743090583

 57
 12
 145
 143
 13
 6
 37
 39
 13
 15
 57
 48
 52
 46
 38
 37
 35
 13
 9
 16

 .00062472599737
 .000132718404725
 .00144195389725
 .00159573280961
 .000138865151256
 .000109128608065
 .000493827160494
 .000527084009082
 .000140692640693
 .000161540449729
 .000664893617021
 .00047848797799
 .000447361855519
 .000515932210994
 .000424899086467
 .000450264073795
 .000392825877124
 .000122697070371
 9.65250965251E-05
 .000166743090583

 57
 12
 145
 143
 13
 6
 37
 39
 13
 15
 57
 48
 52
 46
 38
 37
 35
 13
 9
 16

 0
 0
 0
 0
 .000128183216544

 0
 0
 0
 0
 12

 0
 0
 0
 0
 .000128183216544

 0
 0
 0
 0
 12

 0
 0
 0
 0
 .000128183216544

 0
 0
 0
 0
 12

 1.7864971503711E-03
 .002787086499216
 .001253008214165
 .001260963688709
 3.8989061698882E-03
 3.2556701405962E-03
 2.9229229229234E-03
 2.7705697913279E-03
 2.0021645021641E-03
 .001906177306797
 2.7762224710693E-03
 3.2796363491327E-03
 1.32487934134594E-03
 .00151414888009
 1.3417865888384E-03
 1.3629615206712E-03
 .001279489999777
 7.267441860463E-04
 2.2737022737031E-03
 2.0426028596441E-03

 69.4417177914076
 75.7222222221694
 63.253968253952
 72.6637168141565
 92.7095890411022
 53.0000000000158
 53.6210045662122
 58.2585365853692
 60.7297297297258
 56.4802259886994
 92.8235294117459
 147.273556230929
 49.4025974025969
 38.2888888888967
 94.4666666666234
 80.4107142856848
 106.280701754393
 71.2337662337622
 68.179245283016
 64.397959183678

 .000263042525208
 .000497694017718
 .000835338809443
 .000993148391992
 .00110023927534
 .00080027645914
 .000867534200868
 .000932533246837
 .000660173160173
 .000603084345653
 .00150475923852
 .00202360540691
 .000610820995036
 .000628091387297
 .00118524482014
 .00114391413342
 .00123459561382
 .000698429477499
 .000890175890176
 .000875401225562

 24
 45
 84
 89
 103
 44
 65
 69
 61
 56
 129
 203
 71
 56
 106
 94
 110
 74
 83
 84

 .000263042525208
 .000497694017718
 .000835338809443
 .000993148391992
 .00110023927534
 .00080027645914
 .000867534200868
 .000932533246837
 .000660173160173
 .000603084345653
 .00150475923852
 .00202360540691
 .000610820995036
 .000628091387297
 .00118524482014
 .00114391413342
 .00123459561382
 .000698429477499
 .000890175890176
 .000875401225562

 24
 45
 84
 89
 103
 44
 65
 69
 61
 56
 129
 203
 71
 56
 106
 94
 110
 74
 83
 84

 .000263042525208
 .000497694017718
 .000835338809443
 .000993148391992
 .00110023927534
 .00080027645914
 .000867534200868
 .000932533246837
 .000660173160173
 .000603084345653
 .00150475923852
 .00202360540691
 .000610820995036
 .000628091387297
 .00118524482014
 .00114391413342
 .00123459561382
 .000698429477499
 .000890175890176
 .000875401225562

 24
 45
 84
 89
 103
 44
 65
 69
 61
 56
 129
 203
 71
 56
 106
 94
 110
 74
 83
 84

 .000263042525208
 .000497694017718
 .000835338809443
 .000993148391992
 .00110023927534
 .00080027645914
 .000867534200868
 .000932533246837
 .000660173160173
 .000603084345653
 .00150475923852
 .00202360540691
 .000610820995036
 .000628091387297
 .00118524482014
 .00114391413342
 .00123459561382
 .000698429477499
 .000890175890176
 .000875401225562

 24
 45
 84
 89
 103
 44
 65
 69
 61
 56
 129
 203
 71
 56
 106
 94
 110
 74
 83
 84

 .000263042525208
 .000497694017718
 .000835338809443
 .000993148391992
 .00110023927534
 .00080027645914
 .000867534200868
 .000932533246837
 .000660173160173
 .000603084345653
 .00150475923852
 .00202360540691
 .000610820995036
 .000628091387297
 .00118524482014
 .00114391413342
 .00123459561382
 .000698429477499
 .000890175890176
 .000875401225562

 24
 45
 84
 89
 103
 44
 65
 69
 61
 56
 129
 203
 71
 56
 106
 94
 110
 74
 83
 84

 .001161771153002
 .000442394682416
 0
 0
 8.652367116732E-04
 3.637620268822E-04
 5.872539205874E-04
 2.432695426529E-04
 8.65800865801E-05
 .000043077453261
 8.16536020903E-05
 5.98109972487E-05
 1.2044357648594E-04
 .000235534270236
 0
 0
 0
 0
 8.58000858001E-05
 7.29501021301E-05

 94.5094339622641
 40
 40
 40
 64.506172839501
 16.3000000000009
 32.2272727272753
 9.44444444444271
 8
 4
 7
 6
 8.99999999999817
 8.99999999999745
 8.99999999999745
 8.99999999999745
 8.99999999999745
 8.99999999999745
 8
 7

 .000032880315651
 0
 0
 0
 4.27277388481E-05
 1.81881013441E-05
 9.34267600934E-05
 9.46048221429E-05
 0
 0
 0
 0
 1.72062252123E-05
 8.97273410424E-05

 3
 3
 3
 3
 4
 1
 7
 7
 7
 7
 7
 7
 2
 8

 .000032880315651
 0
 0
 0
 4.27277388481E-05
 1.81881013441E-05
 9.34267600934E-05
 9.46048221429E-05
 0
 0
 0
 0
 1.72062252123E-05
 8.97273410424E-05

 3
 3
 3
 3
 4
 1
 7
 7
 7
 7
 7
 7
 2
 8

 .000032880315651
 0
 0
 0
 4.27277388481E-05
 1.81881013441E-05
 9.34267600934E-05
 9.46048221429E-05
 0
 0
 0
 0
 1.72062252123E-05
 8.97273410424E-05

 3
 3
 3
 3
 4
 1
 7
 7
 7
 7
 7
 7
 2
 8

 .000032880315651
 0
 0
 0
 4.27277388481E-05
 1.81881013441E-05
 9.34267600934E-05
 9.46048221429E-05
 0
 0
 0
 0
 1.72062252123E-05
 8.97273410424E-05

 3
 3
 3
 3
 4
 1
 7
 7
 7
 7
 7
 7
 2
 8

 .0010960105217
 .000442394682416
 0
 0
 5.34096735601E-05
 1.81881013441E-05
 0
 0
 0
 0
 0
 0
 8.60311260614E-06
 2.24318352606E-05

 100
 40
 40
 40
 5
 1
 1
 1
 1
 1
 1
 1
 1
 2

 .0010960105217
 .000442394682416
 0
 0
 5.34096735601E-05
 1.81881013441E-05
 0
 0
 0
 0
 0
 0
 8.60311260614E-06
 2.24318352606E-05

 100
 40
 40
 40
 5
 1
 1
 1
 1
 1
 1
 1
 1
 2

 .0010960105217
 .000442394682416
 0
 0
 5.34096735601E-05
 1.81881013441E-05
 0
 0
 0
 0
 0
 0
 8.60311260614E-06
 2.24318352606E-05

 100
 40
 40
 40
 5
 1
 1
 1
 1
 1
 1
 1
 1
 2

 .0010960105217
 .000442394682416
 0
 0
 5.34096735601E-05
 1.81881013441E-05
 0
 0
 0
 0
 0
 0
 8.60311260614E-06
 2.24318352606E-05

 100
 40
 40
 40
 5
 1
 1
 1
 1
 1
 1
 1
 1
 2

 .000032880315651
 0
 0
 0
 .000769099299265
 .000327385824194
 .000493827160494
 .00014866472051
 8.65800865801E-05
 .000043077453261
 8.16536020903E-05
 5.98109972487E-05
 9.46342386675E-05
 .000123375093933
 0
 0
 0
 0
 8.58000858001E-05
 7.29501021301E-05

 3
 3
 3
 3
 72
 18
 37
 11
 8
 4
 7
 6
 11
 11
 11
 11
 11
 11
 8
 7

 .000032880315651
 0
 0
 0
 .000769099299265
 .000327385824194
 .000493827160494
 .00014866472051
 8.65800865801E-05
 .000043077453261
 8.16536020903E-05
 5.98109972487E-05
 9.46342386675E-05
 .000123375093933
 0
 0
 0
 0
 8.58000858001E-05
 7.29501021301E-05

 3
 3
 3
 3
 72
 18
 37
 11
 8
 4
 7
 6
 11
 11
 11
 11
 11
 11
 8
 7

 .000032880315651
 0
 0
 0
 .000769099299265
 .000327385824194
 .000493827160494
 .00014866472051
 8.65800865801E-05
 .000043077453261
 8.16536020903E-05
 5.98109972487E-05
 9.46342386675E-05
 .000123375093933
 0
 0
 0
 0
 8.58000858001E-05
 7.29501021301E-05

 3
 3
 3
 3
 72
 18
 37
 11
 8
 4
 7
 6
 11
 11
 11
 11
 11
 11
 8
 7

 .000032880315651
 0
 0
 0
 .000769099299265
 .000327385824194
 .000493827160494
 .00014866472051
 8.65800865801E-05
 .000043077453261
 8.16536020903E-05
 5.98109972487E-05
 9.46342386675E-05
 .000123375093933
 0
 0
 0
 0
 8.58000858001E-05
 7.29501021301E-05

 3
 3
 3
 3
 72
 18
 37
 11
 8
 4
 7
 6
 11
 11
 11
 11
 11
 11
 8
 7

 .000284962735642
 .0012276452437
 .000248612740906
 .000145066619055
 .00127115023073
 .00136410760081
 .000667334000667
 .000864958373878
 .000422077922078
 .000635392435599
 .000711552818216
 .00061804697157
 .000249490265578
 .000224318352606
 8.94524392562E-05
 .000133862292209
 .000044894385957
 2.83147085473E-05
 .000546975546976
 .000562757930718

 26
 111
 25
 13
 119
 75
 50
 64
 39
 59
 61
 62
 29
 20
 8
 11
 4
 3
 51
 54

 .000284962735642
 .0012276452437
 .000248612740906
 .000145066619055
 .00127115023073
 .00136410760081
 .000667334000667
 .000864958373878
 .000422077922078
 .000635392435599
 .000711552818216
 .00061804697157
 .000249490265578
 .000224318352606
 8.94524392562E-05
 .000133862292209
 .000044894385957
 2.83147085473E-05
 .000546975546976
 .000562757930718

 26
 111
 25
 13
 119
 75
 50
 64
 39
 59
 61
 62
 29
 20
 8
 11
 4
 3
 51
 54

 .000284962735642
 .0012276452437
 .000248612740906
 .000145066619055
 .00127115023073
 .00136410760081
 .000667334000667
 .000864958373878
 .000422077922078
 .000635392435599
 .000711552818216
 .00061804697157
 .000249490265578
 .000224318352606
 8.94524392562E-05
 .000133862292209
 .000044894385957
 2.83147085473E-05
 .000546975546976
 .000562757930718

 26
 111
 25
 13
 119
 75
 50
 64
 39
 59
 61
 62
 29
 20
 8
 11
 4
 3
 51
 54

 .000284962735642
 .0012276452437
 .000248612740906
 .000145066619055
 .00127115023073
 .00136410760081
 .000667334000667
 .000864958373878
 .000422077922078
 .000635392435599
 .000711552818216
 .00061804697157
 .000249490265578
 .000224318352606
 8.94524392562E-05
 .000133862292209
 .000044894385957
 2.83147085473E-05
 .000546975546976
 .000562757930718

 26
 111
 25
 13
 119
 75
 50
 64
 39
 59
 61
 62
 29
 20
 8
 11
 4
 3
 51
 54

 .000284962735642
 .0012276452437
 .000248612740906
 .000145066619055
 .00127115023073
 .00136410760081
 .000667334000667
 .000864958373878
 .000422077922078
 .000635392435599
 .000711552818216
 .00061804697157
 .000249490265578
 .000224318352606
 8.94524392562E-05
 .000133862292209
 .000044894385957
 2.83147085473E-05
 .000546975546976
 .000562757930718

 26
 111
 25
 13
 119
 75
 50
 64
 39
 59
 61
 62
 29
 20
 8
 11
 4
 3
 51
 54

 7.67207365191E-05
 .000619352555382
 .000169056663816
 .000122748677662
 .000662279952145
 .000727524053764
 .000800800800801
 .00072980862796
 .000833333333333
 .000624623072284
 .000478256812243
 .000578172973404
 .000344124504246
 .000426204869951
 6.70893294422E-05
 8.51850950422E-05
 0
 0
 .000750750750751
 .000531493601234

 7
 56
 17
 11
 62
 40
 60
 54
 77
 58
 41
 58
 40
 38
 6
 7
 7
 7
 70
 51

 7.67207365191E-05
 .000619352555382
 .000169056663816
 .000122748677662
 .000662279952145
 .000727524053764
 .000800800800801
 .00072980862796
 .000833333333333
 .000624623072284
 .000478256812243
 .000578172973404
 .000344124504246
 .000426204869951
 6.70893294422E-05
 8.51850950422E-05
 0
 0
 .000750750750751
 .000531493601234

 7
 56
 17
 11
 62
 40
 60
 54
 77
 58
 41
 58
 40
 38
 6
 7
 7
 7
 70
 51

 7.67207365191E-05
 .000619352555382
 .000169056663816
 .000122748677662
 .000662279952145
 .000727524053764
 .000800800800801
 .00072980862796
 .000833333333333
 .000624623072284
 .000478256812243
 .000578172973404
 .000344124504246
 .000426204869951
 6.70893294422E-05
 8.51850950422E-05
 0
 0
 .000750750750751
 .000531493601234

 7
 56
 17
 11
 62
 40
 60
 54
 77
 58
 41
 58
 40
 38
 6
 7
 7
 7
 70
 51

 7.67207365191E-05
 .000619352555382
 .000169056663816
 .000122748677662
 .000662279952145
 .000727524053764
 .000800800800801
 .00072980862796
 .000833333333333
 .000624623072284
 .000478256812243
 .000578172973404
 .000344124504246
 .000426204869951
 6.70893294422E-05
 8.51850950422E-05
 0
 0
 .000750750750751
 .000531493601234

 7
 56
 17
 11
 62
 40
 60
 54
 77
 58
 41
 58
 40
 38
 6
 7
 7
 7
 70
 51

 7.67207365191E-05
 .000619352555382
 .000169056663816
 .000122748677662
 .000662279952145
 .000727524053764
 .000800800800801
 .00072980862796
 .000833333333333
 .000624623072284
 .000478256812243
 .000578172973404
 .000344124504246
 .000426204869951
 6.70893294422E-05
 8.51850950422E-05
 0
 0
 .000750750750751
 .000531493601234

 7
 56
 17
 11
 62
 40
 60
 54
 77
 58
 41
 58
 40
 38
 6
 7
 7
 7
 70
 51

 3.35927224901425E-02
 4.66836988619504E-02
 3.37616102150309E-02
 3.13901845693677E-02
 4.11147667065847E-02
 4.13233662537638E-02
 3.10310310310362E-02
 3.25981187155713E-02
 5.02813852814198E-02
 5.59899198759726E-02
 4.01619074281247E-02
 3.86379042226883E-02
 4.26456291886159E-02
 4.66582173420851E-02
 2.27209195710637E-02
 2.06026237009209E-02
 1.05389571034217E-02
 2.04809725158718E-02
 2.77134277133893E-02
 2.92425695110519E-02

 335.545513866251
 541.005212035442
 535.166126657297
 367.16850337726
 499.222915043685
 296.149647886892
 524.153548387415
 468.601160862966
 850.699526475415
 856.809578765973
 709.748765611199
 872.181630547297
 1727.3389146655
 1470.67115384661
 420.869094488226
 431.360307147043
 98.1011714590233
 355.038709677466
 513.601393188109
 529.296507485015

 6.57606313021E-05
 .000121658537664
 9.94450963623E-06
 5.57948534827E-05
 .000138865151256
 .000109128608065
 9.34267600934E-05
 8.10898475511E-05
 .00004329004329
 7.53855432067E-05
 4.66592011945E-05
 3.98739981658E-05
 8.60311260614E-06
 1.12159176303E-05
 0
 0
 1.12235964893E-05
 2.83147085473E-05
 .000010725010725
 4.16857726458E-05

 6
 11
 1
 5
 13
 6
 7
 6
 4
 7
 4
 4
 1
 1
 1
 1
 1
 3
 1
 4

 6.57606313021E-05
 .000121658537664
 9.94450963623E-06
 5.57948534827E-05
 .000138865151256
 .000109128608065
 9.34267600934E-05
 8.10898475511E-05
 .00004329004329
 7.53855432067E-05
 4.66592011945E-05
 3.98739981658E-05
 8.60311260614E-06
 1.12159176303E-05
 0
 0
 1.12235964893E-05
 2.83147085473E-05
 .000010725010725
 4.16857726458E-05

 6
 11
 1
 5
 13
 6
 7
 6
 4
 7
 4
 4
 1
 1
 1
 1
 1
 3
 1
 4

 6.57606313021E-05
 .000121658537664
 9.94450963623E-06
 5.57948534827E-05
 .000138865151256
 .000109128608065
 9.34267600934E-05
 8.10898475511E-05
 .00004329004329
 7.53855432067E-05
 4.66592011945E-05
 3.98739981658E-05
 8.60311260614E-06
 1.12159176303E-05
 0
 0
 1.12235964893E-05
 2.83147085473E-05
 .000010725010725
 4.16857726458E-05

 6
 11
 1
 5
 13
 6
 7
 6
 4
 7
 4
 4
 1
 1
 1
 1
 1
 3
 1
 4

 6.57606313021E-05
 .000121658537664
 9.94450963623E-06
 5.57948534827E-05
 .000138865151256
 .000109128608065
 9.34267600934E-05
 8.10898475511E-05
 .00004329004329
 7.53855432067E-05
 4.66592011945E-05
 3.98739981658E-05
 8.60311260614E-06
 1.12159176303E-05
 0
 0
 1.12235964893E-05
 2.83147085473E-05
 .000010725010725
 4.16857726458E-05

 6
 11
 1
 5
 13
 6
 7
 6
 4
 7
 4
 4
 1
 1
 1
 1
 1
 3
 1
 4

 6.57606313021E-05
 .000121658537664
 9.94450963623E-06
 5.57948534827E-05
 .000138865151256
 .000109128608065
 9.34267600934E-05
 8.10898475511E-05
 .00004329004329
 7.53855432067E-05
 4.66592011945E-05
 3.98739981658E-05
 8.60311260614E-06
 1.12159176303E-05
 0
 0
 1.12235964893E-05
 2.83147085473E-05
 .000010725010725
 4.16857726458E-05

 6
 11
 1
 5
 13
 6
 7
 6
 4
 7
 4
 4
 1
 1
 1
 1
 1
 3
 1
 4

 8.3077597544951E-03
 1.08939690544912E-02
 2.2574036874247E-03
 1.9528198718951E-03
 8.9514612886704E-03
 8.9667339626446E-03
 .005912579245912
 6.4196129311241E-03
 1.07359307359261E-02
 1.15232187473019E-02
 3.8727136991395E-03
 3.2198253518885E-03
 1.86687543553288E-03
 .002097376596867
 7.938903983984E-04
 5.962956652952E-04
 2.132483332959E-04
 5.7573240712836E-04
 .002145002145002
 2.6053607903635E-03

 108.725593667577
 122.520812182764
 51.0792951541734
 37.1485714285795
 92.9140811456079
 57.2758620689777
 52.3950338600386
 53.3199999999818
 140.149193548338
 139.702803738335
 50.6204819277179
 49.1176470588233
 29.3317972350272
 23.9946524064174
 13.000000000005
 11.4081632653005
 5.1052631578956
 18.2131147541025
 35.7200000000156
 43.1519999999892

 .000241122314774
 .000630412422443
 1.98890192725E-05
 0
 .0010681934712
 .000909405067205
 .000547213880547
 .00051356903449
 .000746753246753
 .000893857155165
 8.16536020903E-05
 5.98109972487E-05
 .000180665364729
 .000257966105497
 0
 0
 0
 9.43823618242E-06
 .000010725010725

 22
 57
 2
 2
 100
 50
 41
 38
 69
 83
 7
 6
 21
 23
 23
 23
 23
 1
 1

 .000241122314774
 .000630412422443
 1.98890192725E-05
 0
 .0010681934712
 .000909405067205
 .000547213880547
 .00051356903449
 .000746753246753
 .000893857155165
 8.16536020903E-05
 5.98109972487E-05
 .000180665364729
 .000257966105497
 0
 0
 0
 9.43823618242E-06
 .000010725010725

 22
 57
 2
 2
 100
 50
 41
 38
 69
 83
 7
 6
 21
 23
 23
 23
 23
 1
 1

 .000241122314774
 .000630412422443
 1.98890192725E-05
 0
 .0010681934712
 .000909405067205
 .000547213880547
 .00051356903449
 .000746753246753
 .000893857155165
 8.16536020903E-05
 5.98109972487E-05
 .000180665364729
 .000257966105497
 0
 0
 0
 9.43823618242E-06
 .000010725010725

 22
 57
 2
 2
 100
 50
 41
 38
 69
 83
 7
 6
 21
 23
 23
 23
 23
 1
 1

 .000241122314774
 .000630412422443
 1.98890192725E-05
 0
 .0010681934712
 .000909405067205
 .000547213880547
 .00051356903449
 .000746753246753
 .000893857155165
 8.16536020903E-05
 5.98109972487E-05
 .000180665364729
 .000257966105497
 0
 0
 0
 9.43823618242E-06
 .000010725010725

 22
 57
 2
 2
 100
 50
 41
 38
 69
 83
 7
 6
 21
 23
 23
 23
 23
 1
 1

 .00032880315651
 .000409215081235
 .000129278625271
 .000212020443234
 .000587506409161
 .00080027645914
 .000413747080414
 .000608173856633
 .000606060606061
 .000516929439132
 .000361608809257
 .000438613979824
 .000197871589941
 .000257966105497
 .000122997103977
 .000048677197167
 2.24471929785E-05
 9.43823618242E-06
 .00023595023595
 .000281378965359

 30
 37
 13
 19
 55
 44
 31
 45
 56
 48
 31
 44
 23
 23
 11
 4
 2
 1
 22
 27

 .00032880315651
 .000409215081235
 .000129278625271
 .000212020443234
 .000587506409161
 .00080027645914
 .000413747080414
 .000608173856633
 .000606060606061
 .000516929439132
 .000361608809257
 .000438613979824
 .000197871589941
 .000257966105497
 .000122997103977
 .000048677197167
 2.24471929785E-05
 9.43823618242E-06
 .00023595023595
 .000281378965359

 30
 37
 13
 19
 55
 44
 31
 45
 56
 48
 31
 44
 23
 23
 11
 4
 2
 1
 22
 27

 .00032880315651
 .000409215081235
 .000129278625271
 .000212020443234
 .000587506409161
 .00080027645914
 .000413747080414
 .000608173856633
 .000606060606061
 .000516929439132
 .000361608809257
 .000438613979824
 .000197871589941
 .000257966105497
 .000122997103977
 .000048677197167
 2.24471929785E-05
 9.43823618242E-06
 .00023595023595
 .000281378965359

 30
 37
 13
 19
 55
 44
 31
 45
 56
 48
 31
 44
 23
 23
 11
 4
 2
 1
 22
 27

 .00032880315651
 .000409215081235
 .000129278625271
 .000212020443234
 .000587506409161
 .00080027645914
 .000413747080414
 .000608173856633
 .000606060606061
 .000516929439132
 .000361608809257
 .000438613979824
 .000197871589941
 .000257966105497
 .000122997103977
 .000048677197167
 2.24471929785E-05
 9.43823618242E-06
 .00023595023595
 .000281378965359

 30
 37
 13
 19
 55
 44
 31
 45
 56
 48
 31
 44
 23
 23
 11
 4
 2
 1
 22
 27

 .000339763261727
 .00139354324961
 0
 0
 .000480687062041
 .000582019243011
 .000560560560561
 .000486539085307
 .00108225108225
 .00126001550788
 0
 0
 0
 0
 0
 0
 0
 0
 0
 2.08428863229E-05

 31
 126
 126
 126
 45
 32
 42
 36
 100
 117
 117
 117
 117
 117
 117
 117
 117
 117
 117
 2

 .000339763261727
 .00139354324961
 0
 0
 .000480687062041
 .000582019243011
 .000560560560561
 .000486539085307
 .00108225108225
 .00126001550788
 0
 0
 0
 0
 0
 0
 0
 0
 0
 2.08428863229E-05

 31
 126
 126
 126
 45
 32
 42
 36
 100
 117
 117
 117
 117
 117
 117
 117
 117
 117
 117
 2

 .000339763261727
 .00139354324961
 0
 0
 .000480687062041
 .000582019243011
 .000560560560561
 .000486539085307
 .00108225108225
 .00126001550788
 0
 0
 0
 0
 0
 0
 0
 0
 0
 2.08428863229E-05

 31
 126
 126
 126
 45
 32
 42
 36
 100
 117
 117
 117
 117
 117
 117
 117
 117
 117
 117
 2

 .000339763261727
 .00139354324961
 0
 0
 .000480687062041
 .000582019243011
 .000560560560561
 .000486539085307
 .00108225108225
 .00126001550788
 0
 0
 0
 0
 0
 0
 0
 0
 0
 2.08428863229E-05

 31
 126
 126
 126
 45
 32
 42
 36
 100
 117
 117
 117
 117
 117
 117
 117
 117
 117
 117
 2

 .00126041209996
 .00184699779909
 0
 2.23179413931E-05
 .000918646385233
 .000672959749732
 .000867534200868
 .00105416801816
 .00108225108225
 .00130309296114
 .000268290406868
 .000189401491288
 9.46342386675E-05
 .000100943258673
 4.47262196281E-05
 0
 0
 0
 .00015015015015
 8.33715452916E-05

 115
 167
 167
 2
 86
 37
 65
 78
 100
 121
 23
 19
 11
 9
 4
 4
 4
 4
 14
 8

 .00126041209996
 .00184699779909
 0
 2.23179413931E-05
 .000918646385233
 .000672959749732
 .000867534200868
 .00105416801816
 .00108225108225
 .00130309296114
 .000268290406868
 .000189401491288
 9.46342386675E-05
 .000100943258673
 4.47262196281E-05
 0
 0
 0
 .00015015015015
 8.33715452916E-05

 115
 167
 167
 2
 86
 37
 65
 78
 100
 121
 23
 19
 11
 9
 4
 4
 4
 4
 14
 8

 .00126041209996
 .00184699779909
 0
 2.23179413931E-05
 .000918646385233
 .000672959749732
 .000867534200868
 .00105416801816
 .00108225108225
 .00130309296114
 .000268290406868
 .000189401491288
 9.46342386675E-05
 .000100943258673
 4.47262196281E-05
 0
 0
 0
 .00015015015015
 8.33715452916E-05

 115
 167
 167
 2
 86
 37
 65
 78
 100
 121
 23
 19
 11
 9
 4
 4
 4
 4
 14
 8

 .00126041209996
 .00184699779909
 0
 2.23179413931E-05
 .000918646385233
 .000672959749732
 .000867534200868
 .00105416801816
 .00108225108225
 .00130309296114
 .000268290406868
 .000189401491288
 9.46342386675E-05
 .000100943258673
 4.47262196281E-05
 0
 0
 0
 .00015015015015
 8.33715452916E-05

 115
 167
 167
 2
 86
 37
 65
 78
 100
 121
 23
 19
 11
 9
 4
 4
 4
 4
 14
 8

 .001304252520821
 .0012276452437
 9.94450963623E-05
 6.69538241793E-05
 .000726371560417
 .000818464560485
 3.069736403067E-04
 4.459941615312E-04
 5.844155844155E-04
 .000829240975273
 .000384938409854
 2.3924398899445E-04
 1.4625291430414E-04
 .000112159176303
 2.23631098141E-05
 3.65078978752E-05
 8.97887719141E-05
 3.77529447297E-05
 .0001287001287
 .000208428863229

 113.151260504197
 111
 10
 6
 46.9705882353052
 34.5999999999866
 19.3478260869546
 24.5151515151553
 52.0370370370379
 59.5974025974258
 33
 22.083333333332
 15.1176470588221
 6.8
 2
 3
 8
 4
 12
 20

 .001304252520821
 .0012276452437
 9.94450963623E-05
 6.69538241793E-05
 .000726371560417
 .000818464560485
 3.069736403067E-04
 4.459941615312E-04
 5.844155844155E-04
 .000829240975273
 .000384938409854
 2.3924398899445E-04
 1.4625291430414E-04
 .000112159176303
 2.23631098141E-05
 3.65078978752E-05
 8.97887719141E-05
 3.77529447297E-05
 .0001287001287
 .000208428863229

 113.151260504197
 111
 10
 6
 46.9705882353052
 34.5999999999866
 19.3478260869546
 24.5151515151553
 52.0370370370379
 59.5974025974258
 33
 22.083333333332
 15.1176470588221
 6.8
 2
 3
 8
 4
 12
 20

 .00127137220517
 .0012276452437
 9.94450963623E-05
 6.69538241793E-05
 .000587506409161
 .00070933595242
 .00028028028028
 .000378419288572
 .000573593073593
 .000721547342121
 .000384938409854
 .000229275489453
 .000137649801698
 8.97273410424E-05
 2.23631098141E-05
 3.65078978752E-05
 8.97887719141E-05
 3.77529447297E-05
 .0001287001287
 .000208428863229

 116
 111
 10
 6
 55
 39
 21
 28
 53
 67
 33
 23
 16
 8
 2
 3
 8
 4
 12
 20

 .00127137220517
 .0012276452437
 9.94450963623E-05
 6.69538241793E-05
 .000587506409161
 .00070933595242
 .00028028028028
 .000378419288572
 .000573593073593
 .000721547342121
 .000384938409854
 .000229275489453
 .000137649801698
 8.97273410424E-05
 2.23631098141E-05
 3.65078978752E-05
 8.97887719141E-05
 3.77529447297E-05
 .0001287001287
 .000208428863229

 116
 111
 10
 6
 55
 39
 21
 28
 53
 67
 33
 23
 16
 8
 2
 3
 8
 4
 12
 20

 .000032880315651
 0
 0
 0
 .000138865151256
 .000109128608065
 2.66933600267E-05
 6.75748729592E-05
 1.08225108225E-05
 .000107693633152
 0
 9.96849954145E-06
 8.60311260614E-06
 2.24318352606E-05

 3
 3
 3
 3
 13
 6
 2
 5
 1
 10
 10
 1
 1
 2

 .000032880315651
 0
 0
 0
 .000138865151256
 .000109128608065
 2.66933600267E-05
 6.75748729592E-05
 1.08225108225E-05
 .000107693633152
 0
 9.96849954145E-06
 8.60311260614E-06
 2.24318352606E-05

 3
 3
 3
 3
 13
 6
 2
 5
 1
 10
 10
 1
 1
 2

 .000208241999123
 .000420274948295
 0
 0
 9.61374124081E-05
 0
 0
 2.70299491837E-05
 .00034632034632
 .000269234082881
 0
 9.96849954145E-06
 6.88249008491E-05
 5.60795881515E-05

 19
 38
 38
 38
 9
 9
 9
 2
 32
 25
 25
 1
 8
 5

 .000208241999123
 .000420274948295
 0
 0
 9.61374124081E-05
 0
 0
 2.70299491837E-05
 .00034632034632
 .000269234082881
 0
 9.96849954145E-06
 6.88249008491E-05
 5.60795881515E-05

 19
 38
 38
 38
 9
 9
 9
 2
 32
 25
 25
 1
 8
 5

 .000208241999123
 .000420274948295
 0
 0
 9.61374124081E-05
 0
 0
 2.70299491837E-05
 .00034632034632
 .000269234082881
 0
 9.96849954145E-06
 6.88249008491E-05
 5.60795881515E-05

 19
 38
 38
 38
 9
 9
 9
 2
 32
 25
 25
 1
 8
 5

 .000208241999123
 .000420274948295
 0
 0
 9.61374124081E-05
 0
 0
 2.70299491837E-05
 .00034632034632
 .000269234082881
 0
 9.96849954145E-06
 6.88249008491E-05
 5.60795881515E-05

 19
 38
 38
 38
 9
 9
 9
 2
 32
 25
 25
 1
 8
 5

 .000471284524331
 .000884789364832
 .000755782732353
 .00061374338831
 .000886600581097
 .00080027645914
 .000747414080747
 .000824413450103
 .00101731601732
 .00090462651848
 .000781541620007
 .000498424977072
 .000361330729458
 .000392557117061
 .000122997103977
 .000109523693626
 .000044894385957
 .000217079432196
 .000622050622051
 .00050022927175

 43
 80
 76
 55
 83
 44
 56
 61
 94
 84
 67
 50
 42
 35
 11
 9
 4
 23
 58
 48

 .000471284524331
 .000884789364832
 .000755782732353
 .00061374338831
 .000886600581097
 .00080027645914
 .000747414080747
 .000824413450103
 .00101731601732
 .00090462651848
 .000781541620007
 .000498424977072
 .000361330729458
 .000392557117061
 .000122997103977
 .000109523693626
 .000044894385957
 .000217079432196
 .000622050622051
 .00050022927175

 43
 80
 76
 55
 83
 44
 56
 61
 94
 84
 67
 50
 42
 35
 11
 9
 4
 23
 58
 48

 .000471284524331
 .000884789364832
 .000755782732353
 .00061374338831
 .000886600581097
 .00080027645914
 .000747414080747
 .000824413450103
 .00101731601732
 .00090462651848
 .000781541620007
 .000498424977072
 .000361330729458
 .000392557117061
 .000122997103977
 .000109523693626
 .000044894385957
 .000217079432196
 .000622050622051
 .00050022927175

 43
 80
 76
 55
 83
 44
 56
 61
 94
 84
 67
 50
 42
 35
 11
 9
 4
 23
 58
 48

 .000471284524331
 .000884789364832
 .000755782732353
 .00061374338831
 .000886600581097
 .00080027645914
 .000747414080747
 .000824413450103
 .00101731601732
 .00090462651848
 .000781541620007
 .000498424977072
 .000361330729458
 .000392557117061
 .000122997103977
 .000109523693626
 .000044894385957
 .000217079432196
 .000622050622051
 .00050022927175

 43
 80
 76
 55
 83
 44
 56
 61
 94
 84
 67
 50
 42
 35
 11
 9
 4
 23
 58
 48

 .000021920210434
 .000055299335302
 0
 0
 7.47735429841E-05
 1.81881013441E-05
 2.66933600267E-05
 4.05449237755E-05
 .000119047619048
 .00020461790299

 2
 5
 5
 5
 7
 1
 2
 3
 11
 19

 .000021920210434
 .000055299335302
 0
 0
 7.47735429841E-05
 1.81881013441E-05
 2.66933600267E-05
 4.05449237755E-05
 .000119047619048
 .00020461790299

 2
 5
 5
 5
 7
 1
 2
 3
 11
 19

 .000021920210434
 .000055299335302
 0
 0
 7.47735429841E-05
 1.81881013441E-05
 2.66933600267E-05
 4.05449237755E-05
 .000119047619048
 .00020461790299

 2
 5
 5
 5
 7
 1
 2
 3
 11
 19

 .000021920210434
 .000055299335302
 0
 0
 7.47735429841E-05
 1.81881013441E-05
 2.66933600267E-05
 4.05449237755E-05
 .000119047619048
 .00020461790299

 2
 5
 5
 5
 7
 1
 2
 3
 11
 19

 .00190705830776
 .00218985367796
 .000596670578174
 .000524471622738
 .00153819859853
 .00172786962769
 .000814147480814
 .000891988323062
 .00126623376623
 .0017661755837
 .000979843225084
 .000857290960565
 .000172062252123
 .000257966105497
 .000223631098141
 .000219047387251
 3.36707894678E-05
 .000217079432196
 .000504075504076
 .000771186793947

 174
 198
 60
 47
 144
 95
 61
 66
 117
 164
 84
 86
 20
 23
 20
 18
 3
 23
 47
 74

 .00190705830776
 .00218985367796
 .000596670578174
 .000524471622738
 .00153819859853
 .00172786962769
 .000814147480814
 .000891988323062
 .00126623376623
 .0017661755837
 .000979843225084
 .000857290960565
 .000172062252123
 .000257966105497
 .000223631098141
 .000219047387251
 3.36707894678E-05
 .000217079432196
 .000504075504076
 .000771186793947

 174
 198
 60
 47
 144
 95
 61
 66
 117
 164
 84
 86
 20
 23
 20
 18
 3
 23
 47
 74

 .00190705830776
 .00218985367796
 .000596670578174
 .000524471622738
 .00153819859853
 .00172786962769
 .000814147480814
 .000891988323062
 .00126623376623
 .0017661755837
 .000979843225084
 .000857290960565
 .000172062252123
 .000257966105497
 .000223631098141
 .000219047387251
 3.36707894678E-05
 .000217079432196
 .000504075504076
 .000771186793947

 174
 198
 60
 47
 144
 95
 61
 66
 117
 164
 84
 86
 20
 23
 20
 18
 3
 23
 47
 74

 .00190705830776
 .00218985367796
 .000596670578174
 .000524471622738
 .00153819859853
 .00172786962769
 .000814147480814
 .000891988323062
 .00126623376623
 .0017661755837
 .000979843225084
 .000857290960565
 .000172062252123
 .000257966105497
 .000223631098141
 .000219047387251
 3.36707894678E-05
 .000217079432196
 .000504075504076
 .000771186793947

 174
 198
 60
 47
 144
 95
 61
 66
 117
 164
 84
 86
 20
 23
 20
 18
 3
 23
 47
 74

 .000427444103463
 .000420274948295
 0
 0
 .000149547085968
 .000236445317473
 0
 5.40598983674E-05
 .000378787878788
 .000280003446196
 2.33296005972E-05
 1.99369990829E-05
 0
 0
 0
 0
 0
 9.43823618242E-06

 39
 38
 38
 38
 14
 13
 13
 4
 35
 26
 2
 2
 2
 2
 2
 2
 2
 1

 .000427444103463
 .000420274948295
 0
 0
 .000149547085968
 .000236445317473
 0
 5.40598983674E-05
 .000378787878788
 .000280003446196
 2.33296005972E-05
 1.99369990829E-05
 0
 0
 0
 0
 0
 9.43823618242E-06

 39
 38
 38
 38
 14
 13
 13
 4
 35
 26
 2
 2
 2
 2
 2
 2
 2
 1

 .000427444103463
 .000420274948295
 0
 0
 .000149547085968
 .000236445317473
 0
 5.40598983674E-05
 .000378787878788
 .000280003446196
 2.33296005972E-05
 1.99369990829E-05
 0
 0
 0
 0
 0
 9.43823618242E-06

 39
 38
 38
 38
 14
 13
 13
 4
 35
 26
 2
 2
 2
 2
 2
 2
 2
 1

 .000427444103463
 .000420274948295
 0
 0
 .000149547085968
 .000236445317473
 0
 5.40598983674E-05
 .000378787878788
 .000280003446196
 2.33296005972E-05
 1.99369990829E-05
 0
 0
 0
 0
 0
 9.43823618242E-06

 39
 38
 38
 38
 14
 13
 13
 4
 35
 26
 2
 2
 2
 2
 2
 2
 2
 1

 .000175361683472
 .000154838138846
 .000288390779451
 .000234338384627
 .000149547085968
 .000127316709409
 5.33867200534E-05
 .000175694669694
 .000194805194805
 .000172309813044
 .000209966405375
 .000199369990829
 8.60311260614E-06
 5.60795881515E-05
 .000156541768698
 2.43385985835E-05
 0
 2.83147085473E-05
 .000117975117975
 .000198007420068

 16
 14
 29
 21
 14
 7
 4
 13
 18
 16
 18
 20
 1
 5
 14
 2
 2
 3
 11
 19

 .000175361683472
 .000154838138846
 .000288390779451
 .000234338384627
 .000149547085968
 .000127316709409
 5.33867200534E-05
 .000175694669694
 .000194805194805
 .000172309813044
 .000209966405375
 .000199369990829
 8.60311260614E-06
 5.60795881515E-05
 .000156541768698
 2.43385985835E-05
 0
 2.83147085473E-05
 .000117975117975
 .000198007420068

 16
 14
 29
 21
 14
 7
 4
 13
 18
 16
 18
 20
 1
 5
 14
 2
 2
 3
 11
 19

 .000175361683472
 .000154838138846
 .000288390779451
 .000234338384627
 .000149547085968
 .000127316709409
 5.33867200534E-05
 .000175694669694
 .000194805194805
 .000172309813044
 .000209966405375
 .000199369990829
 8.60311260614E-06
 5.60795881515E-05
 .000156541768698
 2.43385985835E-05
 0
 2.83147085473E-05
 .000117975117975
 .000198007420068

 16
 14
 29
 21
 14
 7
 4
 13
 18
 16
 18
 20
 1
 5
 14
 2
 2
 3
 11
 19

 .000175361683472
 .000154838138846
 .000288390779451
 .000234338384627
 .000149547085968
 .000127316709409
 5.33867200534E-05
 .000175694669694
 .000194805194805
 .000172309813044
 .000209966405375
 .000199369990829
 8.60311260614E-06
 5.60795881515E-05
 .000156541768698
 2.43385985835E-05
 0
 2.83147085473E-05
 .000117975117975
 .000198007420068

 16
 14
 29
 21
 14
 7
 4
 13
 18
 16
 18
 20
 1
 5
 14
 2
 2
 3
 11
 19

 8.76808417361E-05
 .000055299335302
 .000328168817996
 .000223179413931
 .000512732866177
 .000600207344355
 .000333667000334
 .000297329441021
 .000119047619048
 .000118462996468
 .000454927211646
 .000448582479365
 .000275299603397
 .000269182023127
 8.94524392562E-05
 .000133862292209
 0
 0
 .0002145002145
 .000343907624328

 8
 5
 33
 20
 48
 33
 25
 22
 11
 11
 39
 45
 32
 24
 8
 11
 11
 11
 20
 33

 8.76808417361E-05
 .000055299335302
 .000328168817996
 .000223179413931
 .000512732866177
 .000600207344355
 .000333667000334
 .000297329441021
 .000119047619048
 .000118462996468
 .000454927211646
 .000448582479365
 .000275299603397
 .000269182023127
 8.94524392562E-05
 .000133862292209
 0
 0
 .0002145002145
 .000343907624328

 8
 5
 33
 20
 48
 33
 25
 22
 11
 11
 39
 45
 32
 24
 8
 11
 11
 11
 20
 33

 8.76808417361E-05
 .000055299335302
 .000328168817996
 .000223179413931
 .000512732866177
 .000600207344355
 .000333667000334
 .000297329441021
 .000119047619048
 .000118462996468
 .000454927211646
 .000448582479365
 .000275299603397
 .000269182023127
 8.94524392562E-05
 .000133862292209
 0
 0
 .0002145002145
 .000343907624328

 8
 5
 33
 20
 48
 33
 25
 22
 11
 11
 39
 45
 32
 24
 8
 11
 11
 11
 20
 33

 8.76808417361E-05
 .000055299335302
 .000328168817996
 .000223179413931
 .000512732866177
 .000600207344355
 .000333667000334
 .000297329441021
 .000119047619048
 .000118462996468
 .000454927211646
 .000448582479365
 .000275299603397
 .000269182023127
 8.94524392562E-05
 .000133862292209
 0
 0
 .0002145002145
 .000343907624328

 8
 5
 33
 20
 48
 33
 25
 22
 11
 11
 39
 45
 32
 24
 8
 11
 11
 11
 20
 33

 .000021920210434
 1.10598670604E-05
 0
 0
 9.61374124081E-05
 5.45643040323E-05
 8.00800800801E-05
 4.05449237755E-05
 1.08225108225E-05
 3.23080899457E-05

 2
 1
 1
 1
 9
 3
 6
 3
 1
 3

 .000021920210434
 1.10598670604E-05
 0
 0
 9.61374124081E-05
 5.45643040323E-05
 8.00800800801E-05
 4.05449237755E-05
 1.08225108225E-05
 3.23080899457E-05

 2
 1
 1
 1
 9
 3
 6
 3
 1
 3

 .000021920210434
 1.10598670604E-05
 0
 0
 9.61374124081E-05
 5.45643040323E-05
 8.00800800801E-05
 4.05449237755E-05
 1.08225108225E-05
 3.23080899457E-05

 2
 1
 1
 1
 9
 3
 6
 3
 1
 3

 .000021920210434
 1.10598670604E-05
 0
 0
 9.61374124081E-05
 5.45643040323E-05
 8.00800800801E-05
 4.05449237755E-05
 1.08225108225E-05
 3.23080899457E-05

 2
 1
 1
 1
 9
 3
 6
 3
 1
 3

 .00151249451995
 .0011723459084
 3.97780385449E-05
 5.57948534827E-05
 .00157024440267
 .00158236481694
 .00108108108108
 .000946048221429
 .00311688311688
 .00287542000517
 .000326614408361
 .000259180988078
 .000361330729458
 .000336477528909
 .000011181554907
 2.43385985835E-05
 2.24471929785E-05
 4.71911809121E-05
 .000160875160875
 .000198007420068

 138
 106
 4
 5
 147
 87
 81
 70
 288
 267
 28
 26
 42
 30
 1
 2
 2
 5
 15
 19

 .00151249451995
 .0011723459084
 3.97780385449E-05
 5.57948534827E-05
 .00157024440267
 .00158236481694
 .00108108108108
 .000946048221429
 .00311688311688
 .00287542000517
 .000326614408361
 .000259180988078
 .000361330729458
 .000336477528909
 .000011181554907
 2.43385985835E-05
 2.24471929785E-05
 4.71911809121E-05
 .000160875160875
 .000198007420068

 138
 106
 4
 5
 147
 87
 81
 70
 288
 267
 28
 26
 42
 30
 1
 2
 2
 5
 15
 19

 .00151249451995
 .0011723459084
 3.97780385449E-05
 5.57948534827E-05
 .00157024440267
 .00158236481694
 .00108108108108
 .000946048221429
 .00311688311688
 .00287542000517
 .000326614408361
 .000259180988078
 .000361330729458
 .000336477528909
 .000011181554907
 2.43385985835E-05
 2.24471929785E-05
 4.71911809121E-05
 .000160875160875
 .000198007420068

 138
 106
 4
 5
 147
 87
 81
 70
 288
 267
 28
 26
 42
 30
 1
 2
 2
 5
 15
 19

 .00151249451995
 .0011723459084
 3.97780385449E-05
 5.57948534827E-05
 .00157024440267
 .00158236481694
 .00108108108108
 .000946048221429
 .00311688311688
 .00287542000517
 .000326614408361
 .000259180988078
 .000361330729458
 .000336477528909
 .000011181554907
 2.43385985835E-05
 2.24471929785E-05
 4.71911809121E-05
 .000160875160875
 .000198007420068

 138
 106
 4
 5
 147
 87
 81
 70
 288
 267
 28
 26
 42
 30
 1
 2
 2
 5
 15
 19

 0
 2.21197341208E-05
 0
 0
 9.61374124081E-05
 3.63762026882E-05
 8.00800800801E-05
 1.35149745918E-05
 6.49350649351E-05
 9.69242698372E-05

 0
 2
 2
 2
 9
 2
 6
 1
 6
 9

 0
 2.21197341208E-05
 0
 0
 9.61374124081E-05
 3.63762026882E-05
 8.00800800801E-05
 1.35149745918E-05
 6.49350649351E-05
 9.69242698372E-05

 0
 2
 2
 2
 9
 2
 6
 1
 6
 9

 0
 2.21197341208E-05
 0
 0
 9.61374124081E-05
 3.63762026882E-05
 8.00800800801E-05
 1.35149745918E-05
 6.49350649351E-05
 9.69242698372E-05

 0
 2
 2
 2
 9
 2
 6
 1
 6
 9

 0
 2.21197341208E-05
 0
 0
 9.61374124081E-05
 3.63762026882E-05
 8.00800800801E-05
 1.35149745918E-05
 6.49350649351E-05
 9.69242698372E-05

 0
 2
 2
 2
 9
 2
 6
 1
 6
 9

 .000284962735642
 .00123870511076
 .00297340838123
 .00283437855692
 .00155956246795
 .00167330532366
 .000747414080747
 .000973078170613
 .00187229437229
 .00235849056604
 .00274122807018
 .00253199888353
 .000886120598433
 .000818761987012
 .000950432167097
 .00102222114051
 .00106624166648
 .00422832980973
 .00203775203775
 .00203218141648

 26
 112
 299
 254
 146
 92
 56
 72
 173
 219
 235
 254
 103
 73
 85
 84
 95
 448
 190
 195

 .000284962735642
 .00123870511076
 .00297340838123
 .00283437855692
 .00155956246795
 .00167330532366
 .000747414080747
 .000973078170613
 .00187229437229
 .00235849056604
 .00274122807018
 .00253199888353
 .000886120598433
 .000818761987012
 .000950432167097
 .00102222114051
 .00106624166648
 .00422832980973
 .00203775203775
 .00203218141648

 26
 112
 299
 254
 146
 92
 56
 72
 173
 219
 235
 254
 103
 73
 85
 84
 95
 448
 190
 195

 .000284962735642
 .00123870511076
 .00297340838123
 .00283437855692
 .00155956246795
 .00167330532366
 .000747414080747
 .000973078170613
 .00187229437229
 .00235849056604
 .00274122807018
 .00253199888353
 .000886120598433
 .000818761987012
 .000950432167097
 .00102222114051
 .00106624166648
 .00422832980973
 .00203775203775
 .00203218141648

 26
 112
 299
 254
 146
 92
 56
 72
 173
 219
 235
 254
 103
 73
 85
 84
 95
 448
 190
 195

 .000284962735642
 .00123870511076
 .00297340838123
 .00283437855692
 .00155956246795
 .00167330532366
 .000747414080747
 .000973078170613
 .00187229437229
 .00235849056604
 .00274122807018
 .00253199888353
 .000886120598433
 .000818761987012
 .000950432167097
 .00102222114051
 .00106624166648
 .00422832980973
 .00203775203775
 .00203218141648

 26
 112
 299
 254
 146
 92
 56
 72
 173
 219
 235
 254
 103
 73
 85
 84
 95
 448
 190
 195

 .000284962735642
 .00123870511076
 .00297340838123
 .00283437855692
 .00155956246795
 .00167330532366
 .000747414080747
 .000973078170613
 .00187229437229
 .00235849056604
 .00274122807018
 .00253199888353
 .000886120598433
 .000818761987012
 .000950432167097
 .00102222114051
 .00106624166648
 .00422832980973
 .00203775203775
 .00203218141648

 26
 112
 299
 254
 146
 92
 56
 72
 173
 219
 235
 254
 103
 73
 85
 84
 95
 448
 190
 195

 2.3125822007891E-03
 2.3336319497432E-03
 5.9667057817333E-04
 7.253330952745E-04
 2.3072978977921E-03
 2.6736508975857E-03
 .001067734401068
 1.2704076116336E-03
 .001742424242424
 2.5092616524487E-03
 1.7730496453868E-03
 1.34574743809535E-03
 5.075836437625E-04
 4.374207875821E-04
 3.801728668391E-04
 4.624333730859E-04
 2.805899122308E-04
 3.492147387498E-04
 8.794508794511E-04
 9.170869982071E-04

 54.6398104265471
 57.6350710900497
 43.0333333333258
 46.4153846154055
 117.574074074069
 63.3673469388112
 40.5000000000054
 52.9361702127846
 61.7453416149085
 72.1673819742133
 71.3157894736306
 74.1259259259376
 40.2881355932273
 27.2051282051326
 28.4117647058822
 16.6842105263134
 8.43999999999875
 21.9729729729762
 51.7804878048869
 54.0454545454693

 .000602805786936
 .000597232821261
 8.95005867261E-05
 .000111589706965
 .000117501281832
 .000254633418817
 .00004004004004
 1.35149745918E-05
 .00017316017316
 .000323080899457
 .000163307204181
 .00013955899358
 2.58093378184E-05
 1.12159176303E-05
 2.23631098141E-05
 2.43385985835E-05
 7.85651754248E-05
 3.77529447297E-05
 .00010725010725
 .000166743090583

 55
 54
 9
 10
 11
 14
 3
 1
 16
 30
 14
 14
 3
 1
 2
 2
 7
 4
 10
 16

 .000602805786936
 .000597232821261
 8.95005867261E-05
 .000111589706965
 .000117501281832
 .000254633418817
 .00004004004004
 1.35149745918E-05
 .00017316017316
 .000323080899457
 .000163307204181
 .00013955899358
 2.58093378184E-05
 1.12159176303E-05
 2.23631098141E-05
 2.43385985835E-05
 7.85651754248E-05
 3.77529447297E-05
 .00010725010725
 .000166743090583

 55
 54
 9
 10
 11
 14
 3
 1
 16
 30
 14
 14
 3
 1
 2
 2
 7
 4
 10
 16

 .000602805786936
 .000597232821261
 8.95005867261E-05
 .000111589706965
 .000117501281832
 .000254633418817
 .00004004004004
 1.35149745918E-05
 .00017316017316
 .000323080899457
 .000163307204181
 .00013955899358
 2.58093378184E-05
 1.12159176303E-05
 2.23631098141E-05
 2.43385985835E-05
 7.85651754248E-05
 3.77529447297E-05
 .00010725010725
 .000166743090583

 55
 54
 9
 10
 11
 14
 3
 1
 16
 30
 14
 14
 3
 1
 2
 2
 7
 4
 10
 16

 .000602805786936
 .000597232821261
 8.95005867261E-05
 .000111589706965
 .000117501281832
 .000254633418817
 .00004004004004
 1.35149745918E-05
 .00017316017316
 .000323080899457
 .000163307204181
 .00013955899358
 2.58093378184E-05
 1.12159176303E-05
 2.23631098141E-05
 2.43385985835E-05
 7.85651754248E-05
 3.77529447297E-05
 .00010725010725
 .000166743090583

 55
 54
 9
 10
 11
 14
 3
 1
 16
 30
 14
 14
 3
 1
 2
 2
 7
 4
 10
 16

 5.48005260851E-05
 3.31796011812E-05
 0
 0
 5.34096735601E-05
 3.63762026882E-05
 1.33466800133E-05
 1.35149745918E-05
 .000021645021645
 3.23080899457E-05
 3.49944008959E-05
 9.96849954145E-06
 0
 0
 .000011181554907
 1.21692992917E-05
 0
 0
 0
 1.04214431614E-05

 5
 3
 3
 3
 3.40000000000112
 2
 1
 1
 2
 3
 3
 1
 1
 1
 1
 1
 1
 1
 1
 1

 5.48005260851E-05
 3.31796011812E-05
 0
 0
 .000010681934712
 0
 0
 0
 0
 3.23080899457E-05
 3.49944008959E-05
 9.96849954145E-06
 0
 0
 .000011181554907
 1.21692992917E-05
 0
 0
 0
 1.04214431614E-05

 5
 3
 3
 3
 1
 1
 1
 1
 1
 3
 3
 1
 1
 1
 1
 1
 1
 1
 1
 1

 5.48005260851E-05
 3.31796011812E-05
 0
 0
 .000010681934712
 0
 0
 0
 0
 3.23080899457E-05
 3.49944008959E-05
 9.96849954145E-06
 0
 0
 .000011181554907
 1.21692992917E-05
 0
 0
 0
 1.04214431614E-05

 5
 3
 3
 3
 1
 1
 1
 1
 1
 3
 3
 1
 1
 1
 1
 1
 1
 1
 1
 1

 5.48005260851E-05
 3.31796011812E-05
 0
 0
 .000010681934712
 0
 0
 0
 0
 3.23080899457E-05
 3.49944008959E-05
 9.96849954145E-06
 0
 0
 .000011181554907
 1.21692992917E-05
 0
 0
 0
 1.04214431614E-05

 5
 3
 3
 3
 1
 1
 1
 1
 1
 3
 3
 1
 1
 1
 1
 1
 1
 1
 1
 1

 0
 0
 0
 0
 4.27277388481E-05
 3.63762026882E-05
 1.33466800133E-05
 1.35149745918E-05
 .000021645021645

 0
 0
 0
 0
 4
 2
 1
 1
 2

 0
 0
 0
 0
 4.27277388481E-05
 3.63762026882E-05
 1.33466800133E-05
 1.35149745918E-05
 .000021645021645

 0
 0
 0
 0
 4
 2
 1
 1
 2

 0
 0
 0
 0
 4.27277388481E-05
 3.63762026882E-05
 1.33466800133E-05
 1.35149745918E-05
 .000021645021645

 0
 0
 0
 0
 4
 2
 1
 1
 2

 .000295922840859
 .000519813751839
 0
 0
 .000352503845496
 .00070933595242
 .000226893560227
 .000243269542653
 .000411255411255
 .000506160075816
 8.16536020903E-05
 .000109653494956
 0
 0
 0
 .000146031591501
 0
 1.88764723648E-05
 7.50750750751E-05
 3.12643294843E-05

 27
 47
 47
 47
 33
 39
 17
 18
 38
 47
 7
 11
 11
 11
 11
 12
 12
 2
 7
 3

 .000295922840859
 .000519813751839
 0
 0
 .000352503845496
 .00070933595242
 .000226893560227
 .000243269542653
 .000411255411255
 .000506160075816
 8.16536020903E-05
 .000109653494956
 0
 0
 0
 .000146031591501
 0
 1.88764723648E-05
 7.50750750751E-05
 3.12643294843E-05

 27
 47
 47
 47
 33
 39
 17
 18
 38
 47
 7
 11
 11
 11
 11
 12
 12
 2
 7
 3

 .000295922840859
 .000519813751839
 0
 0
 .000352503845496
 .00070933595242
 .000226893560227
 .000243269542653
 .000411255411255
 .000506160075816
 8.16536020903E-05
 .000109653494956
 0
 0
 0
 .000146031591501
 0
 1.88764723648E-05
 7.50750750751E-05
 3.12643294843E-05

 27
 47
 47
 47
 33
 39
 17
 18
 38
 47
 7
 11
 11
 11
 11
 12
 12
 2
 7
 3

 .000295922840859
 .000519813751839
 0
 0
 .000352503845496
 .00070933595242
 .000226893560227
 .000243269542653
 .000411255411255
 .000506160075816
 8.16536020903E-05
 .000109653494956
 0
 0
 0
 .000146031591501
 0
 1.88764723648E-05
 7.50750750751E-05
 3.12643294843E-05

 27
 47
 47
 47
 33
 39
 17
 18
 38
 47
 7
 11
 11
 11
 11
 12
 12
 2
 7
 3

 .001359053046909
 .001183405775462
 5.0716999144723E-04
 6.137433883095E-04
 .001783883096904
 1.6733053236605E-03
 7.874541207877E-04
 .001000108119797
 .001136363636364
 .00164771258723
 1.4930944382196E-03
 1.0865664500179E-03
 4.817743059441E-04
 4.262048699518E-04
 .000346628202118
 2.798938837097E-04
 .000202024736806
 2.925853216553E-04
 .000697125697126
 7.086581349784E-04

 62.5000000000092
 65.6728971962716
 49.0392156862736
 53.0363636363668
 144.724550898204
 82.5434782608783
 49.847457627122
 62.8378378378449
 78.4476190475995
 89.5228758169471
 82.7031249999774
 88.8899082568836
 42.285714285719
 27.8947368421091
 31
 21.0869565217411
 9
 25.5806451612892
 63.0307692307706
 66.0294117647103

 .000504164839982
 .000232257208268
 9.94450963623E-06
 1.11589706965E-05
 .000128183216544
 9.09405067205E-05
 6.67334000667E-05
 6.75748729592E-05
 .00004329004329
 .000129232359783
 .000326614408361
 .000089716495873
 6.88249008491E-05
 6.72955057818E-05
 0
 1.21692992917E-05
 .000101012368403
 2.83147085473E-05
 .000010725010725
 1.04214431614E-05

 46
 21
 1
 1
 12
 5
 5
 5
 4
 12
 28
 9
 8
 6
 6
 1
 9
 3
 1
 1

 .000504164839982
 .000232257208268
 9.94450963623E-06
 1.11589706965E-05
 .000128183216544
 9.09405067205E-05
 6.67334000667E-05
 6.75748729592E-05
 .00004329004329
 .000129232359783
 .000326614408361
 .000089716495873
 6.88249008491E-05
 6.72955057818E-05
 0
 1.21692992917E-05
 .000101012368403
 2.83147085473E-05
 .000010725010725
 1.04214431614E-05

 46
 21
 1
 1
 12
 5
 5
 5
 4
 12
 28
 9
 8
 6
 6
 1
 9
 3
 1
 1

 .000504164839982
 .000232257208268
 9.94450963623E-06
 1.11589706965E-05
 .000128183216544
 9.09405067205E-05
 6.67334000667E-05
 6.75748729592E-05
 .00004329004329
 .000129232359783
 .000326614408361
 .000089716495873
 6.88249008491E-05
 6.72955057818E-05
 0
 1.21692992917E-05
 .000101012368403
 2.83147085473E-05
 .000010725010725
 1.04214431614E-05

 46
 21
 1
 1
 12
 5
 5
 5
 4
 12
 28
 9
 8
 6
 6
 1
 9
 3
 1
 1

 .000854888206927
 .000951148567194
 .000497225481811
 .000602584417613
 .00165569988036
 .00158236481694
 .000720720720721
 9.325332468378E-04
 .001093073593074
 .001518480227447
 1.1664800298586E-03
 9.968499541449E-04
 .000412949405095
 .00035890936417
 .000346628202118
 .000267724584418
 .000101012368403
 .000264270613108
 .000686400686401
 .000698236691817

 72.2307692307725
 76.5813953488354
 50
 54
 155
 87
 54
 67.0289855072501
 81.3960396039361
 96.1205673758504
 98.0199999999988
 96.0799999999998
 48
 32
 31
 22
 9
 28
 64
 67

 .000854888206927
 .000951148567194
 .000497225481811
 .000602584417613
 .00165569988036
 .00158236481694
 .000720720720721
 9.325332468378E-04
 .001093073593074
 .001518480227447
 1.1664800298586E-03
 9.968499541449E-04
 .000412949405095
 .00035890936417
 .000346628202118
 .000267724584418
 .000101012368403
 .000264270613108
 .000686400686401
 .000698236691817

 72.2307692307725
 76.5813953488354
 50
 54
 155
 87
 54
 67.0289855072501
 81.3960396039361
 96.1205673758504
 98.0199999999988
 96.0799999999998
 48
 32
 31
 22
 9
 28
 64
 67

 .000854888206927
 .000951148567194
 .000497225481811
 .000602584417613
 .00165569988036
 .00158236481694
 .000720720720721
 9.325332468378E-04
 .001093073593074
 .001518480227447
 1.1664800298586E-03
 9.968499541449E-04
 .000412949405095
 .00035890936417
 .000346628202118
 .000267724584418
 .000101012368403
 .000264270613108
 .000686400686401
 .000698236691817

 72.2307692307725
 76.5813953488354
 50
 54
 155
 87
 54
 67.0289855072501
 81.3960396039361
 96.1205673758504
 98.0199999999988
 96.0799999999998
 48
 32
 31
 22
 9
 28
 64
 67

 .00909688733012
 .0139686120973
 .0120428011695
 .00978641730087
 .0125833190908
 .0126771066368
 .013987320654
 .0134879446427
 .0195454545455
 .0203217885759
 .0167506532288
 .0170461342159
 .0231509760231
 .024787177963
 .00878870215692
 .00956506924331
 .00228961368381
 .00633305647841
 .0116795366795
 .0119325524199

 830
 1263
 1211
 877
 1178
 697
 1048
 998
 1806
 1887
 1436
 1710
 2691
 2210
 786
 786
 204
 671
 1089
 1145

 .00909688733012
 .0139686120973
 .0120428011695
 .00978641730087
 .0125833190908
 .0126771066368
 .013987320654
 .0134879446427
 .0195454545455
 .0203217885759
 .0167506532288
 .0170461342159
 .0231509760231
 .024787177963
 .00878870215692
 .00956506924331
 .00228961368381
 .00633305647841
 .0116795366795
 .0119325524199

 830
 1263
 1211
 877
 1178
 697
 1048
 998
 1806
 1887
 1436
 1710
 2691
 2210
 786
 786
 204
 671
 1089
 1145

 .00909688733012
 .0139686120973
 .0120428011695
 .00978641730087
 .0125833190908
 .0126771066368
 .013987320654
 .0134879446427
 .0195454545455
 .0203217885759
 .0167506532288
 .0170461342159
 .0231509760231
 .024787177963
 .00878870215692
 .00956506924331
 .00228961368381
 .00633305647841
 .0116795366795
 .0119325524199

 830
 1263
 1211
 877
 1178
 697
 1048
 998
 1806
 1887
 1436
 1710
 2691
 2210
 786
 786
 204
 671
 1089
 1145

 .00909688733012
 .0139686120973
 .0120428011695
 .00978641730087
 .0125833190908
 .0126771066368
 .013987320654
 .0134879446427
 .0195454545455
 .0203217885759
 .0167506532288
 .0170461342159
 .0231509760231
 .024787177963
 .00878870215692
 .00956506924331
 .00228961368381
 .00633305647841
 .0116795366795
 .0119325524199

 830
 1263
 1211
 877
 1178
 697
 1048
 998
 1806
 1887
 1436
 1710
 2691
 2210
 786
 786
 204
 671
 1089
 1145

 .00909688733012
 .0139686120973
 .0120428011695
 .00978641730087
 .0125833190908
 .0126771066368
 .013987320654
 .0134879446427
 .0195454545455
 .0203217885759
 .0167506532288
 .0170461342159
 .0231509760231
 .024787177963
 .00878870215692
 .00956506924331
 .00228961368381
 .00633305647841
 .0116795366795
 .0119325524199

 830
 1263
 1211
 877
 1178
 697
 1048
 998
 1806
 1887
 1436
 1710
 2691
 2210
 786
 786
 204
 671
 1089
 1145

 .00356203419553
 .00682393797627
 .000815449790171
 .00101546633339
 .00466800546915
 .00467434204543
 .00314981648315
 .00291923451184
 .00614718614719
 .0065693116223
 .00239128406122
 .00294070736473
 .00951504254239
 .0121804865465
 .000223631098141
 .000170370190084
 3.36707894678E-05
 .000141573542736
 .000600600600601
 .000604443703364

 325
 617
 82
 91
 437
 257
 236
 216
 568
 610
 205
 295
 1106
 1086
 20
 14
 3
 15
 56
 58

 .00356203419553
 .00682393797627
 .000815449790171
 .00101546633339
 .00466800546915
 .00467434204543
 .00314981648315
 .00291923451184
 .00614718614719
 .0065693116223
 .00239128406122
 .00294070736473
 .00951504254239
 .0121804865465
 .000223631098141
 .000170370190084
 3.36707894678E-05
 .000141573542736
 .000600600600601
 .000604443703364

 325
 617
 82
 91
 437
 257
 236
 216
 568
 610
 205
 295
 1106
 1086
 20
 14
 3
 15
 56
 58

 .00356203419553
 .00682393797627
 .000815449790171
 .00101546633339
 .00466800546915
 .00467434204543
 .00314981648315
 .00291923451184
 .00614718614719
 .0065693116223
 .00239128406122
 .00294070736473
 .00951504254239
 .0121804865465
 .000223631098141
 .000170370190084
 3.36707894678E-05
 .000141573542736
 .000600600600601
 .000604443703364

 325
 617
 82
 91
 437
 257
 236
 216
 568
 610
 205
 295
 1106
 1086
 20
 14
 3
 15
 56
 58

 .00356203419553
 .00682393797627
 .000815449790171
 .00101546633339
 .00466800546915
 .00467434204543
 .00314981648315
 .00291923451184
 .00614718614719
 .0065693116223
 .00239128406122
 .00294070736473
 .00951504254239
 .0121804865465
 .000223631098141
 .000170370190084
 3.36707894678E-05
 .000141573542736
 .000600600600601
 .000604443703364

 325
 617
 82
 91
 437
 257
 236
 216
 568
 610
 205
 295
 1106
 1086
 20
 14
 3
 15
 56
 58

 .00356203419553
 .00682393797627
 .000815449790171
 .00101546633339
 .00466800546915
 .00467434204543
 .00314981648315
 .00291923451184
 .00614718614719
 .0065693116223
 .00239128406122
 .00294070736473
 .00951504254239
 .0121804865465
 .000223631098141
 .000170370190084
 3.36707894678E-05
 .000141573542736
 .000600600600601
 .000604443703364

 325
 617
 82
 91
 437
 257
 236
 216
 568
 610
 205
 295
 1106
 1086
 20
 14
 3
 15
 56
 58

 7.67207365191E-05
 1.216585376644E-04
 7.1401579188186E-03
 6.9743566853328E-03
 1.281832165441E-04
 3.63762026882E-05
 1.067734401067E-04
 1.351497459184E-04
 0
 3.23080899456E-05
 2.099664053752E-04
 2.193069899118E-04
 1.1700233144354E-03
 7.514664812305E-04
 8.4532555097013E-03
 .006096818945159
 .001975352982111
 2.8692237994588E-03
 4.9871299871321E-03
 .005471257659752

 7
 5
 199.208913649033
 170.116800000001
 7.50000000000117
 1
 4.25000000000023
 4.60000000000192
 4.60000000000192
 1
 14.3333333333365
 15.0909090909078
 53.3970588235291
 29.6268656716425
 283.592592592612
 190.85628742511
 76.3295454545756
 228.894736842179
 129.227956989194
 151.030476190487

 7.67207365191E-05
 8.84789364832E-05
 .001581177032165
 .001685004575177
 0
 1.81881013441E-05
 6.67334000667E-05
 5.40598983673E-05
 0
 2.15387266304E-05
 0
 1.99369990829E-05
 1.290466890921E-04
 5.60795881515E-05
 .001677233236051
 .001083067636966
 .000213248333296
 1.321353065538E-04
 .00318532818533
 .00350160490224

 7
 6.25
 124.031446540892
 110.62913907284
 110.62913907284
 1
 5
 2.50000000000093
 2.50000000000093
 1
 1
 2
 9.13333333333116
 2.6
 83.3333333333134
 68.2359550561818
 19
 10.5714285714307
 163.693602693504
 186.005952380997

 7.67207365191E-05
 8.84789364832E-05
 .001581177032165
 .001685004575177
 0
 1.81881013441E-05
 6.67334000667E-05
 5.40598983673E-05
 0
 2.15387266304E-05
 0
 1.99369990829E-05
 1.290466890921E-04
 5.60795881515E-05
 .001677233236051
 .001083067636966
 .000213248333296
 1.321353065538E-04
 .00318532818533
 .00350160490224

 7
 6.25
 124.031446540892
 110.62913907284
 110.62913907284
 1
 5
 2.50000000000093
 2.50000000000093
 1
 1
 2
 9.13333333333116
 2.6
 83.3333333333134
 68.2359550561818
 19
 10.5714285714307
 163.693602693504
 186.005952380997

 7.67207365191E-05
 7.74190694228E-05
 .00138228683944
 .00141718927846
 0
 1.81881013441E-05
 6.67334000667E-05
 4.05449237755E-05
 0
 1.07693633152E-05
 0
 1.99369990829E-05
 9.46342386675E-05
 3.36477528909E-05
 .0011181554907
 .000937036045465
 .000213248333296
 .000113258834189
 .00108322608323
 .00117762307724

 7
 7
 139
 127
 127
 1
 5
 3
 3
 1
 1
 2
 11
 3
 100
 77
 19
 12
 101
 113

 7.67207365191E-05
 7.74190694228E-05
 .00138228683944
 .00141718927846
 0
 1.81881013441E-05
 6.67334000667E-05
 4.05449237755E-05
 0
 1.07693633152E-05
 0
 1.99369990829E-05
 9.46342386675E-05
 3.36477528909E-05
 .0011181554907
 .000937036045465
 .000213248333296
 .000113258834189
 .00108322608323
 .00117762307724

 7
 7
 139
 127
 127
 1
 5
 3
 3
 1
 1
 2
 11
 3
 100
 77
 19
 12
 101
 113

 0
 1.10598670604E-05
 .000198890192725
 .000267815296717
 0
 0
 0
 1.35149745918E-05
 0
 1.07693633152E-05
 0
 0
 3.44124504246E-05
 2.24318352606E-05
 .000559077745351
 .000146031591501
 0
 1.88764723648E-05
 .0021021021021
 .002323981825

 0
 1
 20
 24
 24
 24
 24
 1
 1
 1
 1
 1
 4
 2
 50
 12
 12
 2
 196
 223

 0
 1.10598670604E-05
 .000198890192725
 .000267815296717
 0
 0
 0
 1.35149745918E-05
 0
 1.07693633152E-05
 0
 0
 3.44124504246E-05
 2.24318352606E-05
 .000559077745351
 .000146031591501
 0
 1.88764723648E-05
 .0021021021021
 .002323981825

 0
 1
 20
 24
 24
 24
 24
 1
 1
 1
 1
 1
 4
 2
 50
 12
 12
 2
 196
 223

 0
 2.21197341208E-05
 .00196901290797
 .00198629678398
 9.61374124081E-05
 1.81881013441E-05
 .00004004004004
 8.10898475511E-05
 0
 1.07693633152E-05
 .000186636804778
 .000179432991746
 .000550599206793
 .000291613858388
 .00130824192412
 .00113174483413
 .00123459561382
 .00248225611598
 .000654225654226
 .000541915044395

 0
 2
 198
 178
 9
 1
 3
 6
 6
 1
 16
 18
 64
 26
 117
 93
 110
 263
 61
 52

 0
 2.21197341208E-05
 .00196901290797
 .00198629678398
 9.61374124081E-05
 1.81881013441E-05
 .00004004004004
 8.10898475511E-05
 0
 1.07693633152E-05
 .000186636804778
 .000179432991746
 .000550599206793
 .000291613858388
 .00130824192412
 .00113174483413
 .00123459561382
 .00248225611598
 .000654225654226
 .000541915044395

 0
 2
 198
 178
 9
 1
 3
 6
 6
 1
 16
 18
 64
 26
 117
 93
 110
 263
 61
 52

 0
 2.21197341208E-05
 .00196901290797
 .00198629678398
 9.61374124081E-05
 1.81881013441E-05
 .00004004004004
 8.10898475511E-05
 0
 1.07693633152E-05
 .000186636804778
 .000179432991746
 .000550599206793
 .000291613858388
 .00130824192412
 .00113174483413
 .00123459561382
 .00248225611598
 .000654225654226
 .000541915044395

 0
 2
 198
 178
 9
 1
 3
 6
 6
 1
 16
 18
 64
 26
 117
 93
 110
 263
 61
 52

 0
 2.21197341208E-05
 .00196901290797
 .00198629678398
 9.61374124081E-05
 1.81881013441E-05
 .00004004004004
 8.10898475511E-05
 0
 1.07693633152E-05
 .000186636804778
 .000179432991746
 .000550599206793
 .000291613858388
 .00130824192412
 .00113174483413
 .00123459561382
 .00248225611598
 .000654225654226
 .000541915044395

 0
 2
 198
 178
 9
 1
 3
 6
 6
 1
 16
 18
 64
 26
 117
 93
 110
 263
 61
 52

 0
 1.10598670604E-05
 .00283418524633
 .00264467605508
 .000032045804136
 0
 0
 0
 0
 0
 1.16648002986E-05
 1.99369990829E-05
 .000473171193338
 .000403773034691
 .00483043171983
 .00344391169956
 .000190801140317
 .000066067653277
 .000933075933076
 .00122973029305

 0
 1
 285
 237
 3
 3
 3
 3
 3
 3
 1
 2
 55
 36
 432
 283
 17
 7
 87
 118

 0
 1.10598670604E-05
 .00283418524633
 .00264467605508
 .000032045804136
 0
 0
 0
 0
 0
 1.16648002986E-05
 1.99369990829E-05
 .000473171193338
 .000403773034691
 .00483043171983
 .00344391169956
 .000190801140317
 .000066067653277
 .000933075933076
 .00122973029305

 0
 1
 285
 237
 3
 3
 3
 3
 3
 3
 1
 2
 55
 36
 432
 283
 17
 7
 87
 118

 0
 1.10598670604E-05
 .00283418524633
 .00264467605508
 .000032045804136
 0
 0
 0
 0
 0
 1.16648002986E-05
 1.99369990829E-05
 .000473171193338
 .000403773034691
 .00483043171983
 .00344391169956
 .000190801140317
 .000066067653277
 .000933075933076
 .00122973029305

 0
 1
 285
 237
 3
 3
 3
 3
 3
 3
 1
 2
 55
 36
 432
 283
 17
 7
 87
 118

 0
 1.10598670604E-05
 .00283418524633
 .00264467605508
 .000032045804136
 0
 0
 0
 0
 0
 1.16648002986E-05
 1.99369990829E-05
 .000473171193338
 .000403773034691
 .00483043171983
 .00344391169956
 .000190801140317
 .000066067653277
 .000933075933076
 .00122973029305

 0
 1
 285
 237
 3
 3
 3
 3
 3
 3
 1
 2
 55
 36
 432
 283
 17
 7
 87
 118

 0
 0
 .000507169991448
 .000446358827862
 0
 0
 0
 0
 0
 0
 1.16648002986E-05
 0
 1.72062252123E-05
 0
 .000424899086467
 .000340740380169
 2.24471929785E-05
 .000066067653277
 .0001287001287
 .000104214431614

 0
 0
 51
 40
 40
 40
 40
 40
 40
 40
 1
 1
 2
 2
 38
 28
 2
 7
 12
 10

 0
 0
 .000507169991448
 .000446358827862
 0
 0
 0
 0
 0
 0
 1.16648002986E-05
 0
 1.72062252123E-05
 0
 .000424899086467
 .000340740380169
 2.24471929785E-05
 .000066067653277
 .0001287001287
 .000104214431614

 0
 0
 51
 40
 40
 40
 40
 40
 40
 40
 1
 1
 2
 2
 38
 28
 2
 7
 12
 10

 0
 0
 .000507169991448
 .000446358827862
 0
 0
 0
 0
 0
 0
 1.16648002986E-05
 0
 1.72062252123E-05
 0
 .000424899086467
 .000340740380169
 2.24471929785E-05
 .000066067653277
 .0001287001287
 .000104214431614

 0
 0
 51
 40
 40
 40
 40
 40
 40
 40
 1
 1
 2
 2
 38
 28
 2
 7
 12
 10

 0
 0
 .000507169991448
 .000446358827862
 0
 0
 0
 0
 0
 0
 1.16648002986E-05
 0
 1.72062252123E-05
 0
 .000424899086467
 .000340740380169
 2.24471929785E-05
 .000066067653277
 .0001287001287
 .000104214431614

 0
 0
 51
 40
 40
 40
 40
 40
 40
 40
 1
 1
 2
 2
 38
 28
 2
 7
 12
 10

 0
 0
 1.98890192725E-05
 1.11589706965E-05
 0
 0
 0
 0
 0
 0
 0
 0
 0
 0
 3.35446647211E-05
 2.43385985835E-05
 2.24471929785E-05

 0
 0
 2
 1
 1
 1
 1
 1
 1
 1
 1
 1
 1
 1
 3
 2
 2

 0
 0
 1.98890192725E-05
 1.11589706965E-05
 0
 0
 0
 0
 0
 0
 0
 0
 0
 0
 3.35446647211E-05
 2.43385985835E-05
 2.24471929785E-05

 0
 0
 2
 1
 1
 1
 1
 1
 1
 1
 1
 1
 1
 1
 3
 2
 2

 0
 0
 1.98890192725E-05
 1.11589706965E-05
 0
 0
 0
 0
 0
 0
 0
 0
 0
 0
 3.35446647211E-05
 2.43385985835E-05
 2.24471929785E-05

 0
 0
 2
 1
 1
 1
 1
 1
 1
 1
 1
 1
 1
 1
 3
 2
 2

 0
 0
 1.98890192725E-05
 1.11589706965E-05
 0
 0
 0
 0
 0
 0
 0
 0
 0
 0
 3.35446647211E-05
 2.43385985835E-05
 2.24471929785E-05

 0
 0
 2
 1
 1
 1
 1
 1
 1
 1
 1
 1
 1
 1
 3
 2
 2

 0
 0
 .000139223134907
 8.92717655723E-05
 0
 0
 0
 0
 0
 0
 0
 0
 0
 0
 .000100633994163
 2.43385985835E-05
 .000291813508721
 .000122697070371
 6.43500643501E-05
 8.33715452916E-05

 0
 0
 14
 8
 8
 8
 8
 8
 8
 8
 8
 8
 8
 8
 9
 2
 26
 13
 6
 8

 0
 0
 .000139223134907
 8.92717655723E-05
 0
 0
 0
 0
 0
 0
 0
 0
 0
 0
 .000100633994163
 2.43385985835E-05
 .000291813508721
 .000122697070371
 6.43500643501E-05
 8.33715452916E-05

 0
 0
 14
 8
 8
 8
 8
 8
 8
 8
 8
 8
 8
 8
 9
 2
 26
 13
 6
 8

 0
 0
 .000139223134907
 8.92717655723E-05
 0
 0
 0
 0
 0
 0
 0
 0
 0
 0
 .000100633994163
 2.43385985835E-05
 .000291813508721
 .000122697070371
 6.43500643501E-05
 8.33715452916E-05

 0
 0
 14
 8
 8
 8
 8
 8
 8
 8
 8
 8
 8
 8
 9
 2
 26
 13
 6
 8

 0
 0
 .000139223134907
 8.92717655723E-05
 0
 0
 0
 0
 0
 0
 0
 0
 0
 0
 .000100633994163
 2.43385985835E-05
 .000291813508721
 .000122697070371
 6.43500643501E-05
 8.33715452916E-05

 0
 0
 14
 8
 8
 8
 8
 8
 8
 8
 8
 8
 8
 8
 9
 2
 26
 13
 6
 8

 0
 0
 8.95005867261E-05
 .000111589706965
 0
 0
 0
 0
 0
 0
 0
 0
 0
 0
 7.82708843492E-05
 .000048677197167
 0
 0
 .00002145002145
 1.04214431614E-05

 0
 0
 9
 10
 10
 10
 10
 10
 10
 10
 10
 10
 10
 10
 7
 4
 4
 4
 2
 1

 0
 0
 8.95005867261E-05
 .000111589706965
 0
 0
 0
 0
 0
 0
 0
 0
 0
 0
 7.82708843492E-05
 .000048677197167
 0
 0
 .00002145002145
 1.04214431614E-05

 0
 0
 9
 10
 10
 10
 10
 10
 10
 10
 10
 10
 10
 10
 7
 4
 4
 4
 2
 1

 0
 0
 8.95005867261E-05
 .000111589706965
 0
 0
 0
 0
 0
 0
 0
 0
 0
 0
 7.82708843492E-05
 .000048677197167
 0
 0
 .00002145002145
 1.04214431614E-05

 0
 0
 9
 10
 10
 10
 10
 10
 10
 10
 10
 10
 10
 10
 7
 4
 4
 4
 2
 1

 0
 0
 8.95005867261E-05
 .000111589706965
 0
 0
 0
 0
 0
 0
 0
 0
 0
 0
 7.82708843492E-05
 .000048677197167
 0
 0
 .00002145002145
 1.04214431614E-05

 0
 0
 9
 10
 10
 10
 10
 10
 10
 10
 10
 10
 10
 10
 7
 4
 4
 4
 2
 1

 .000252082419991
 .000331796011812
 .000248612740906
 .000412881915772
 .000929328319945
 .00130954329678
 .000373707040374
 .000527084009082
 .001829004329
 .00110924442147
 .000396603210153
 .000508393476614
 .0012646575531
 .00143563745668
 6.70893294422E-05
 .000121692992917
 0
 1.88764723648E-05
 .000203775203775
 .000156321647422

 23
 30
 25
 37
 87
 72
 28
 39
 169
 103
 34
 51
 147
 128
 6
 10
 10
 2
 19
 15

 .000252082419991
 .000331796011812
 .000248612740906
 .000412881915772
 .000929328319945
 .00130954329678
 .000373707040374
 .000527084009082
 .001829004329
 .00110924442147
 .000396603210153
 .000508393476614
 .0012646575531
 .00143563745668
 6.70893294422E-05
 .000121692992917
 0
 1.88764723648E-05
 .000203775203775
 .000156321647422

 23
 30
 25
 37
 87
 72
 28
 39
 169
 103
 34
 51
 147
 128
 6
 10
 10
 2
 19
 15

 .000252082419991
 .000331796011812
 .000248612740906
 .000412881915772
 .000929328319945
 .00130954329678
 .000373707040374
 .000527084009082
 .001829004329
 .00110924442147
 .000396603210153
 .000508393476614
 .0012646575531
 .00143563745668
 6.70893294422E-05
 .000121692992917
 0
 1.88764723648E-05
 .000203775203775
 .000156321647422

 23
 30
 25
 37
 87
 72
 28
 39
 169
 103
 34
 51
 147
 128
 6
 10
 10
 2
 19
 15

 .000252082419991
 .000331796011812
 .000248612740906
 .000412881915772
 .000929328319945
 .00130954329678
 .000373707040374
 .000527084009082
 .001829004329
 .00110924442147
 .000396603210153
 .000508393476614
 .0012646575531
 .00143563745668
 6.70893294422E-05
 .000121692992917
 0
 1.88764723648E-05
 .000203775203775
 .000156321647422

 23
 30
 25
 37
 87
 72
 28
 39
 169
 103
 34
 51
 147
 128
 6
 10
 10
 2
 19
 15

 .000252082419991
 .000331796011812
 .000248612740906
 .000412881915772
 .000929328319945
 .00130954329678
 .000373707040374
 .000527084009082
 .001829004329
 .00110924442147
 .000396603210153
 .000508393476614
 .0012646575531
 .00143563745668
 6.70893294422E-05
 .000121692992917
 0
 1.88764723648E-05
 .000203775203775
 .000156321647422

 23
 30
 25
 37
 87
 72
 28
 39
 169
 103
 34
 51
 147
 128
 6
 10
 10
 2
 19
 15

 .00232354230601
 .00303040357455
 .00223751466815
 .00228758899279
 .00441163903606
 .0044560848293
 .00192192192192
 .00239215050276
 .00388528138528
 .00528775738778
 .00519083613289
 .00458550978907
 .00154856026911
 .00140198970379
 .0006373486297
 .000352909679461
 .000561179824463
 .00115146481426
 .00126555126555
 .00139647338363

 212
 274
 225
 205
 413
 245
 144
 177
 359
 491
 445
 460
 180
 125
 57
 29
 50
 122
 118
 134

 .00232354230601
 .00303040357455
 .00223751466815
 .00228758899279
 .00441163903606
 .0044560848293
 .00192192192192
 .00239215050276
 .00388528138528
 .00528775738778
 .00519083613289
 .00458550978907
 .00154856026911
 .00140198970379
 .0006373486297
 .000352909679461
 .000561179824463
 .00115146481426
 .00126555126555
 .00139647338363

 212
 274
 225
 205
 413
 245
 144
 177
 359
 491
 445
 460
 180
 125
 57
 29
 50
 122
 118
 134

 .00232354230601
 .00303040357455
 .00223751466815
 .00228758899279
 .00441163903606
 .0044560848293
 .00192192192192
 .00239215050276
 .00388528138528
 .00528775738778
 .00519083613289
 .00458550978907
 .00154856026911
 .00140198970379
 .0006373486297
 .000352909679461
 .000561179824463
 .00115146481426
 .00126555126555
 .00139647338363

 212
 274
 225
 205
 413
 245
 144
 177
 359
 491
 445
 460
 180
 125
 57
 29
 50
 122
 118
 134

 .00232354230601
 .00303040357455
 .00223751466815
 .00228758899279
 .00441163903606
 .0044560848293
 .00192192192192
 .00239215050276
 .00388528138528
 .00528775738778
 .00519083613289
 .00458550978907
 .00154856026911
 .00140198970379
 .0006373486297
 .000352909679461
 .000561179824463
 .00115146481426
 .00126555126555
 .00139647338363

 212
 274
 225
 205
 413
 245
 144
 177
 359
 491
 445
 460
 180
 125
 57
 29
 50
 122
 118
 134

 .00232354230601
 .00303040357455
 .00223751466815
 .00228758899279
 .00441163903606
 .0044560848293
 .00192192192192
 .00239215050276
 .00388528138528
 .00528775738778
 .00519083613289
 .00458550978907
 .00154856026911
 .00140198970379
 .0006373486297
 .000352909679461
 .000561179824463
 .00115146481426
 .00126555126555
 .00139647338363

 212
 274
 225
 205
 413
 245
 144
 177
 359
 491
 445
 460
 180
 125
 57
 29
 50
 122
 118
 134

 .000010960105217
 0
 9.94450963623E-06
 2.23179413931E-05
 .000021363869424
 3.63762026882E-05
 2.66933600267E-05
 2.70299491837E-05
 1.08225108225E-05
 2.15387266305E-05
 3.49944008959E-05
 9.96849954145E-06
 0
 1.12159176303E-05
 0
 1.21692992917E-05
 0
 0
 .00002145002145

 1
 1
 1
 2
 2
 2
 2
 2
 1
 2
 3
 1
 1
 1
 1
 1
 1
 1
 2

 .000010960105217
 0
 9.94450963623E-06
 2.23179413931E-05
 .000021363869424
 3.63762026882E-05
 2.66933600267E-05
 2.70299491837E-05
 1.08225108225E-05
 2.15387266305E-05
 3.49944008959E-05
 9.96849954145E-06
 0
 1.12159176303E-05
 0
 1.21692992917E-05
 0
 0
 .00002145002145

 1
 1
 1
 2
 2
 2
 2
 2
 1
 2
 3
 1
 1
 1
 1
 1
 1
 1
 2

 .000010960105217
 0
 9.94450963623E-06
 2.23179413931E-05
 .000021363869424
 3.63762026882E-05
 2.66933600267E-05
 2.70299491837E-05
 1.08225108225E-05
 2.15387266305E-05
 3.49944008959E-05
 9.96849954145E-06
 0
 1.12159176303E-05
 0
 1.21692992917E-05
 0
 0
 .00002145002145

 1
 1
 1
 2
 2
 2
 2
 2
 1
 2
 3
 1
 1
 1
 1
 1
 1
 1
 2

 .000010960105217
 0
 9.94450963623E-06
 2.23179413931E-05
 .000021363869424
 3.63762026882E-05
 2.66933600267E-05
 2.70299491837E-05
 1.08225108225E-05
 2.15387266305E-05
 3.49944008959E-05
 9.96849954145E-06
 0
 1.12159176303E-05
 0
 1.21692992917E-05
 0
 0
 .00002145002145

 1
 1
 1
 2
 2
 2
 2
 2
 1
 2
 3
 1
 1
 1
 1
 1
 1
 1
 2

 .000010960105217
 0
 9.94450963623E-06
 2.23179413931E-05
 .000021363869424
 3.63762026882E-05
 2.66933600267E-05
 2.70299491837E-05
 1.08225108225E-05
 2.15387266305E-05
 3.49944008959E-05
 9.96849954145E-06
 0
 1.12159176303E-05
 0
 1.21692992917E-05
 0
 0
 .00002145002145

 1
 1
 1
 2
 2
 2
 2
 2
 1
 2
 3
 1
 1
 1
 1
 1
 1
 1
 2

 .000120561157387
 .000210137474148
 .000188945683088
 .000100430736269
 .000149547085968
 .000181881013441
 .000146813480147
 .000283814466429
 .000151515151515
 .000107693633152
 .000244960806271
 .000169464492205
 2.58093378184E-05
 4.48636705212E-05
 2.23631098141E-05
 3.65078978752E-05
 1.12235964893E-05
 9.43823618242E-06
 .0001716001716
 .000156321647422

 11
 19
 19
 9
 14
 10
 11
 21
 14
 10
 21
 17
 3
 4
 2
 3
 1
 1
 16
 15

 .000120561157387
 .000210137474148
 .000188945683088
 .000100430736269
 .000149547085968
 .000181881013441
 .000146813480147
 .000283814466429
 .000151515151515
 .000107693633152
 .000244960806271
 .000169464492205
 2.58093378184E-05
 4.48636705212E-05
 2.23631098141E-05
 3.65078978752E-05
 1.12235964893E-05
 9.43823618242E-06
 .0001716001716
 .000156321647422

 11
 19
 19
 9
 14
 10
 11
 21
 14
 10
 21
 17
 3
 4
 2
 3
 1
 1
 16
 15

 .000120561157387
 .000210137474148
 .000188945683088
 .000100430736269
 .000149547085968
 .000181881013441
 .000146813480147
 .000283814466429
 .000151515151515
 .000107693633152
 .000244960806271
 .000169464492205
 2.58093378184E-05
 4.48636705212E-05
 2.23631098141E-05
 3.65078978752E-05
 1.12235964893E-05
 9.43823618242E-06
 .0001716001716
 .000156321647422

 11
 19
 19
 9
 14
 10
 11
 21
 14
 10
 21
 17
 3
 4
 2
 3
 1
 1
 16
 15

 .000120561157387
 .000210137474148
 .000188945683088
 .000100430736269
 .000149547085968
 .000181881013441
 .000146813480147
 .000283814466429
 .000151515151515
 .000107693633152
 .000244960806271
 .000169464492205
 2.58093378184E-05
 4.48636705212E-05
 2.23631098141E-05
 3.65078978752E-05
 1.12235964893E-05
 9.43823618242E-06
 .0001716001716
 .000156321647422

 11
 19
 19
 9
 14
 10
 11
 21
 14
 10
 21
 17
 3
 4
 2
 3
 1
 1
 16
 15

 .000120561157387
 .000210137474148
 .000188945683088
 .000100430736269
 .000149547085968
 .000181881013441
 .000146813480147
 .000283814466429
 .000151515151515
 .000107693633152
 .000244960806271
 .000169464492205
 2.58093378184E-05
 4.48636705212E-05
 2.23631098141E-05
 3.65078978752E-05
 1.12235964893E-05
 9.43823618242E-06
 .0001716001716
 .000156321647422

 11
 19
 19
 9
 14
 10
 11
 21
 14
 10
 21
 17
 3
 4
 2
 3
 1
 1
 16
 15

 .002422183252959
 3.0967627769114E-03
 3.28168817995993E-03
 2.8232195862212E-03
 2.5636643308831E-03
 2.1825721612918E-03
 2.3089756423041E-03
 2.7300248675513E-03
 1.8181818181817E-03
 .002520031015766
 2.7062336692753E-03
 2.5020933849042E-03
 1.2904668909214E-03
 1.2673986922238E-03
 1.7331410105894E-03
 1.5211624114692E-03
 1.6274214909433E-03
 .001934838417397
 1.8983268983271E-03
 .002125974404935

 57.0090497737693
 82.1642857142706
 108.000000000024
 78.1146245059016
 60.2166666666714
 33.3666666666632
 58.1213872831864
 69.0594059405659
 52.3928571428626
 57.6068376068372
 64.9396551723503
 75.9800796812821
 46.2666666666658
 38.1150442477956
 57.8645161290392
 52.7920000000031
 41.9241379310252
 53.4878048780289
 49.0451977401254
 54.2450980392308

 .000964489259097
 1.5483813884554E-03
 1.33256429125493E-03
 1.2274867766162E-03
 .000982737993505
 8.548407631724E-04
 1.2545879212501E-03
 1.5001621796929E-03
 1.0064935064936E-03
 .001098475058155
 .001248133631948
 1.2161569440572E-03
 4.559649681257E-04
 4.935003757333E-04
 .000670893294422
 7.544965560887E-04
 7.968753507373E-04
 .000792811839323
 7.400257400261E-04
 8.128725665933E-04

 54.0227272727387
 116.242857142838
 126.194029850746
 102.290909090896
 69.4130434782706
 39.6808510638286
 82.7659574467885
 97.8828828828761
 76.7419354838714
 82.3725490195857
 90.5140186915328
 112.40983606558
 43.9433962264176
 42.0454545454547
 60
 60.0322580645204
 61.7042253521118
 84
 57.8985507246374
 72.1794871794924

 .000964489259097
 1.5483813884554E-03
 1.33256429125493E-03
 1.2274867766162E-03
 .000982737993505
 8.548407631724E-04
 1.2545879212501E-03
 1.5001621796929E-03
 1.0064935064936E-03
 .001098475058155
 .001248133631948
 1.2161569440572E-03
 4.559649681257E-04
 4.935003757333E-04
 .000670893294422
 7.544965560887E-04
 7.968753507373E-04
 .000792811839323
 7.400257400261E-04
 8.128725665933E-04

 54.0227272727387
 116.242857142838
 126.194029850746
 102.290909090896
 69.4130434782706
 39.6808510638286
 82.7659574467885
 97.8828828828761
 76.7419354838714
 82.3725490195857
 90.5140186915328
 112.40983606558
 43.9433962264176
 42.0454545454547
 60
 60.0322580645204
 61.7042253521118
 84
 57.8985507246374
 72.1794871794924

 .000964489259097
 1.5483813884554E-03
 1.33256429125493E-03
 1.2274867766162E-03
 .000982737993505
 8.548407631724E-04
 1.2545879212501E-03
 1.5001621796929E-03
 1.0064935064936E-03
 .001098475058155
 .001248133631948
 1.2161569440572E-03
 4.559649681257E-04
 4.935003757333E-04
 .000670893294422
 7.544965560887E-04
 7.968753507373E-04
 .000792811839323
 7.400257400261E-04
 8.128725665933E-04

 54.0227272727387
 116.242857142838
 126.194029850746
 102.290909090896
 69.4130434782706
 39.6808510638286
 82.7659574467885
 97.8828828828761
 76.7419354838714
 82.3725490195857
 90.5140186915328
 112.40983606558
 43.9433962264176
 42.0454545454547
 60
 60.0322580645204
 61.7042253521118
 84
 57.8985507246374
 72.1794871794924

 .000964489259097
 1.5483813884554E-03
 1.33256429125493E-03
 1.2274867766162E-03
 .000982737993505
 8.548407631724E-04
 1.2545879212501E-03
 1.5001621796929E-03
 1.0064935064936E-03
 .001098475058155
 .001248133631948
 1.2161569440572E-03
 4.559649681257E-04
 4.935003757333E-04
 .000670893294422
 7.544965560887E-04
 7.968753507373E-04
 .000792811839323
 7.400257400261E-04
 8.128725665933E-04

 54.0227272727387
 116.242857142838
 126.194029850746
 102.290909090896
 69.4130434782706
 39.6808510638286
 82.7659574467885
 97.8828828828761
 76.7419354838714
 82.3725490195857
 90.5140186915328
 112.40983606558
 43.9433962264176
 42.0454545454547
 60
 60.0322580645204
 61.7042253521118
 84
 57.8985507246374
 72.1794871794924

 .000120561157387
 .000210137474148
 .000318224308359
 .00025665632602
 .000224320628952
 .000163692912097
 .00028028028028
 .000283814466429
 .000292207792208
 .00045231325924
 .000209966405375
 .000189401491288
 .000180665364729
 .000201886517345
 .00011181554907
 .000097354394334
 .000101012368403
 .000302023557838
 .000203775203775
 .000312643294843

 11
 19
 32
 23
 21
 9
 21
 21
 27
 42
 18
 19
 21
 18
 10
 8
 9
 32
 19
 30

 .000120561157387
 .000210137474148
 .000318224308359
 .00025665632602
 .000224320628952
 .000163692912097
 .00028028028028
 .000283814466429
 .000292207792208
 .00045231325924
 .000209966405375
 .000189401491288
 .000180665364729
 .000201886517345
 .00011181554907
 .000097354394334
 .000101012368403
 .000302023557838
 .000203775203775
 .000312643294843

 11
 19
 32
 23
 21
 9
 21
 21
 27
 42
 18
 19
 21
 18
 10
 8
 9
 32
 19
 30

 .000120561157387
 .000210137474148
 .000318224308359
 .00025665632602
 .000224320628952
 .000163692912097
 .00028028028028
 .000283814466429
 .000292207792208
 .00045231325924
 .000209966405375
 .000189401491288
 .000180665364729
 .000201886517345
 .00011181554907
 .000097354394334
 .000101012368403
 .000302023557838
 .000203775203775
 .000312643294843

 11
 19
 32
 23
 21
 9
 21
 21
 27
 42
 18
 19
 21
 18
 10
 8
 9
 32
 19
 30

 .000120561157387
 .000210137474148
 .000318224308359
 .00025665632602
 .000224320628952
 .000163692912097
 .00028028028028
 .000283814466429
 .000292207792208
 .00045231325924
 .000209966405375
 .000189401491288
 .000180665364729
 .000201886517345
 .00011181554907
 .000097354394334
 .000101012368403
 .000302023557838
 .000203775203775
 .000312643294843

 11
 19
 32
 23
 21
 9
 21
 21
 27
 42
 18
 19
 21
 18
 10
 8
 9
 32
 19
 30

 .000898728627795
 .000497694017718
 .000169056663816
 .000100430736269
 .000470005127329
 .000345573925538
 .0002002002002
 .000243269542653
 .000194805194805
 .000333850262772
 .000606569615528
 .000438613979824
 .000060221788243
 4.48636705212E-05
 6.70893294422E-05
 2.43385985835E-05
 .000291813508721
 .000169888251284
 .0001716001716
 .000312643294843

 82
 45
 17
 9
 44
 19
 15
 18
 18
 31
 52
 44
 7
 4
 6
 2
 26
 18
 16
 30

 .000898728627795
 .000497694017718
 .000169056663816
 .000100430736269
 .000470005127329
 .000345573925538
 .0002002002002
 .000243269542653
 .000194805194805
 .000333850262772
 .000606569615528
 .000438613979824
 .000060221788243
 4.48636705212E-05
 6.70893294422E-05
 2.43385985835E-05
 .000291813508721
 .000169888251284
 .0001716001716
 .000312643294843

 82
 45
 17
 9
 44
 19
 15
 18
 18
 31
 52
 44
 7
 4
 6
 2
 26
 18
 16
 30

 .000898728627795
 .000497694017718
 .000169056663816
 .000100430736269
 .000470005127329
 .000345573925538
 .0002002002002
 .000243269542653
 .000194805194805
 .000333850262772
 .000606569615528
 .000438613979824
 .000060221788243
 4.48636705212E-05
 6.70893294422E-05
 2.43385985835E-05
 .000291813508721
 .000169888251284
 .0001716001716
 .000312643294843

 82
 45
 17
 9
 44
 19
 15
 18
 18
 31
 52
 44
 7
 4
 6
 2
 26
 18
 16
 30

 .000898728627795
 .000497694017718
 .000169056663816
 .000100430736269
 .000470005127329
 .000345573925538
 .0002002002002
 .000243269542653
 .000194805194805
 .000333850262772
 .000606569615528
 .000438613979824
 .000060221788243
 4.48636705212E-05
 6.70893294422E-05
 2.43385985835E-05
 .000291813508721
 .000169888251284
 .0001716001716
 .000312643294843

 82
 45
 17
 9
 44
 19
 15
 18
 18
 31
 52
 44
 7
 4
 6
 2
 26
 18
 16
 30

 .00043840420868
 .00084054989659
 .00146184291653
 .001238645747316
 8.866005810971E-04
 8.184645604844E-04
 .000573907240574
 7.027786787764E-04
 3.246753246751E-04
 .000635392435599
 6.415640164243E-04
 .000657920969735
 5.936147698237E-04
 5.271481286243E-04
 8.833428376552E-04
 .000644972862463
 .000437720263082
 .000670114768952
 .000782925782926
 6.878152486557E-04

 25
 57.1842105263378
 118.48299319729
 71.1801801801827
 68.5421686746992
 37.7111111111107
 37.4186046511658
 44.6153846153856
 20.3999999999956
 39.881355932195
 42.7818181818224
 46.3636363636538
 59.7246376811597
 45.0425531914897
 66.2405063291136
 53
 24.1282051281973
 36.0704225352126
 55.7397260274065
 55.0909090909091

 .00010960105217
 .000121658537664
 .00015911215418
 .00029013323811
 8.54554776961E-05
 7.27524053764E-05
 .00004004004004
 5.40598983674E-05
 6.49350649351E-05
 .000129232359783
 8.16536020903E-05
 .000119621994497
 4.30155630307E-05
 1.12159176303E-05
 7.82708843492E-05
 0
 .000112235964893
 .000292585321655
 .00010725010725
 6.25286589687E-05

 10
 11
 16
 26
 8
 4
 3
 4
 6
 12
 7
 12
 5
 1
 7
 7
 10
 31
 10
 6

 .00010960105217
 .000121658537664
 .00015911215418
 .00029013323811
 8.54554776961E-05
 7.27524053764E-05
 .00004004004004
 5.40598983674E-05
 6.49350649351E-05
 .000129232359783
 8.16536020903E-05
 .000119621994497
 4.30155630307E-05
 1.12159176303E-05
 7.82708843492E-05
 0
 .000112235964893
 .000292585321655
 .00010725010725
 6.25286589687E-05

 10
 11
 16
 26
 8
 4
 3
 4
 6
 12
 7
 12
 5
 1
 7
 7
 10
 31
 10
 6

 .00010960105217
 .000121658537664
 .00015911215418
 .00029013323811
 8.54554776961E-05
 7.27524053764E-05
 .00004004004004
 5.40598983674E-05
 6.49350649351E-05
 .000129232359783
 8.16536020903E-05
 .000119621994497
 4.30155630307E-05
 1.12159176303E-05
 7.82708843492E-05
 0
 .000112235964893
 .000292585321655
 .00010725010725
 6.25286589687E-05

 10
 11
 16
 26
 8
 4
 3
 4
 6
 12
 7
 12
 5
 1
 7
 7
 10
 31
 10
 6

 .00032880315651
 .000718891358926
 .00130273076235
 .000948512509206
 .000801145103401
 .000745712155108
 .000533867200534
 .000648718780409
 .00025974025974
 .000506160075816
 .000559910414334
 .000538298975238
 .000550599206793
 .000515932210994
 .000805071953306
 .000644972862463
 .000325484298189
 .000377529447297
 .000675675675676
 .000625286589687

 30
 65
 131
 85
 75
 41
 40
 48
 24
 47
 48
 54
 64
 46
 72
 53
 29
 40
 63
 60

 .00032880315651
 .000718891358926
 .00130273076235
 .000948512509206
 .000801145103401
 .000745712155108
 .000533867200534
 .000648718780409
 .00025974025974
 .000506160075816
 .000559910414334
 .000538298975238
 .000550599206793
 .000515932210994
 .000805071953306
 .000644972862463
 .000325484298189
 .000377529447297
 .000675675675676
 .000625286589687

 30
 65
 131
 85
 75
 41
 40
 48
 24
 47
 48
 54
 64
 46
 72
 53
 29
 40
 63
 60

 .00032880315651
 .000718891358926
 .00130273076235
 .000948512509206
 .000801145103401
 .000745712155108
 .000533867200534
 .000648718780409
 .00025974025974
 .000506160075816
 .000559910414334
 .000538298975238
 .000550599206793
 .000515932210994
 .000805071953306
 .000644972862463
 .000325484298189
 .000377529447297
 .000675675675676
 .000625286589687

 30
 65
 131
 85
 75
 41
 40
 48
 24
 47
 48
 54
 64
 46
 72
 53
 29
 40
 63
 60

 .00146865409908
 .00180275833084
 .000626504107082
 .000859240743634
 .000811827038113
 .000818464560485
 .000613947280614
 .000810898475511
 .00106060606061
 .00143232532093
 .00201801045166
 .00178436141792
 .000335521391639
 .000572011799145
 .000167723323605
 .00020687808796
 .000190801140317
 .00031146179402
 .000332475332475
 .000302221851682

 134
 163
 63
 77
 76
 45
 46
 60
 98
 133
 173
 179
 39
 51
 15
 17
 17
 33
 31
 29

 .00146865409908
 .00180275833084
 .000626504107082
 .000859240743634
 .000811827038113
 .000818464560485
 .000613947280614
 .000810898475511
 .00106060606061
 .00143232532093
 .00201801045166
 .00178436141792
 .000335521391639
 .000572011799145
 .000167723323605
 .00020687808796
 .000190801140317
 .00031146179402
 .000332475332475
 .000302221851682

 134
 163
 63
 77
 76
 45
 46
 60
 98
 133
 173
 179
 39
 51
 15
 17
 17
 33
 31
 29

 .00146865409908
 .00180275833084
 .000626504107082
 .000859240743634
 .000811827038113
 .000818464560485
 .000613947280614
 .000810898475511
 .00106060606061
 .00143232532093
 .00201801045166
 .00178436141792
 .000335521391639
 .000572011799145
 .000167723323605
 .00020687808796
 .000190801140317
 .00031146179402
 .000332475332475
 .000302221851682

 134
 163
 63
 77
 76
 45
 46
 60
 98
 133
 173
 179
 39
 51
 15
 17
 17
 33
 31
 29

 .00146865409908
 .00180275833084
 .000626504107082
 .000859240743634
 .000811827038113
 .000818464560485
 .000613947280614
 .000810898475511
 .00106060606061
 .00143232532093
 .00201801045166
 .00178436141792
 .000335521391639
 .000572011799145
 .000167723323605
 .00020687808796
 .000190801140317
 .00031146179402
 .000332475332475
 .000302221851682

 134
 163
 63
 77
 76
 45
 46
 60
 98
 133
 173
 179
 39
 51
 15
 17
 17
 33
 31
 29

 .00146865409908
 .00180275833084
 .000626504107082
 .000859240743634
 .000811827038113
 .000818464560485
 .000613947280614
 .000810898475511
 .00106060606061
 .00143232532093
 .00201801045166
 .00178436141792
 .000335521391639
 .000572011799145
 .000167723323605
 .00020687808796
 .000190801140317
 .00031146179402
 .000332475332475
 .000302221851682

 134
 163
 63
 77
 76
 45
 46
 60
 98
 133
 173
 179
 39
 51
 15
 17
 17
 33
 31
 29

 3.2880315651011E-03
 2.7096674297962E-03
 1.3325642912549E-03
 1.5399379561233E-03
 .001890702444029
 1.5278005129043E-03
 5.739072405733E-04
 5.405989836741E-04
 1.4393939393905E-03
 2.1215645731012E-03
 1.7847144456835E-03
 1.73451892021215E-03
 1.0753890757672E-03
 8.411938222729E-04
 5.031699708162E-04
 4.380947745027E-04
 2.2783900873243E-03
 2.5294472968873E-03
 .001480051480051
 1.5006878152485E-03

 210.226666666672
 147.955102040786
 43.3731343283577
 42.6231884057979
 109.926553672329
 42.8333333333294
 27.2325581395364
 29.8000000000008
 98.7293233082182
 123.91370558376
 76.9477124182568
 75.3563218390877
 68.8719999999916
 35.986666666665
 22.6000000000081
 16.4999999999976
 92.881773399061
 77.6268656716331
 45.797101449291
 52.9722222222225

 .000032880315651
 1.10598670604E-05
 3.97780385449E-05
 6.69538241793E-05
 .000021363869424
 1.81881013441E-05
 0
 0
 0
 1.07693633152E-05
 0
 5.98109972487E-05
 1.72062252123E-05
 3.36477528909E-05
 0
 2.43385985835E-05
 .000291813508721
 .000726744186047
 .000225225225225
 7.29501021301E-05

 3
 1
 4
 6
 2
 1
 1
 1
 1
 1
 1
 6
 2
 3
 3
 2
 26
 77
 21
 7

 .000032880315651
 1.10598670604E-05
 3.97780385449E-05
 6.69538241793E-05
 .000021363869424
 1.81881013441E-05
 0
 0
 0
 1.07693633152E-05
 0
 5.98109972487E-05
 1.72062252123E-05
 3.36477528909E-05
 0
 2.43385985835E-05
 .000291813508721
 .000726744186047
 .000225225225225
 7.29501021301E-05

 3
 1
 4
 6
 2
 1
 1
 1
 1
 1
 1
 6
 2
 3
 3
 2
 26
 77
 21
 7

 .000032880315651
 1.10598670604E-05
 3.97780385449E-05
 6.69538241793E-05
 .000021363869424
 1.81881013441E-05
 0
 0
 0
 1.07693633152E-05
 0
 5.98109972487E-05
 1.72062252123E-05
 3.36477528909E-05
 0
 2.43385985835E-05
 .000291813508721
 .000726744186047
 .000225225225225
 7.29501021301E-05

 3
 1
 4
 6
 2
 1
 1
 1
 1
 1
 1
 6
 2
 3
 3
 2
 26
 77
 21
 7

 .000032880315651
 1.10598670604E-05
 3.97780385449E-05
 6.69538241793E-05
 .000021363869424
 1.81881013441E-05
 0
 0
 0
 1.07693633152E-05
 0
 5.98109972487E-05
 1.72062252123E-05
 3.36477528909E-05
 0
 2.43385985835E-05
 .000291813508721
 .000726744186047
 .000225225225225
 7.29501021301E-05

 3
 1
 4
 6
 2
 1
 1
 1
 1
 1
 1
 6
 2
 3
 3
 2
 26
 77
 21
 7

 .000493204734765
 .000685711757745
 .000437558423994
 .000513312652041
 .000448641257905
 .000509266837635
 .00012012012012
 8.10898475511E-05
 .000205627705628
 .000506160075816
 .000536580813736
 .000628015471111
 .000249490265578
 .000325261611279
 6.70893294422E-05
 7.30157957505E-05
 5.61179824463E-05
 .000368091211114
 .000225225225225
 .000302221851682

 45
 62
 44
 46
 42
 28
 9
 6
 19
 47
 46
 63
 29
 29
 6
 6
 5
 39
 21
 29

 .000493204734765
 .000685711757745
 .000437558423994
 .000513312652041
 .000448641257905
 .000509266837635
 .00012012012012
 8.10898475511E-05
 .000205627705628
 .000506160075816
 .000536580813736
 .000628015471111
 .000249490265578
 .000325261611279
 6.70893294422E-05
 7.30157957505E-05
 5.61179824463E-05
 .000368091211114
 .000225225225225
 .000302221851682

 45
 62
 44
 46
 42
 28
 9
 6
 19
 47
 46
 63
 29
 29
 6
 6
 5
 39
 21
 29

 .000493204734765
 .000685711757745
 .000437558423994
 .000513312652041
 .000448641257905
 .000509266837635
 .00012012012012
 8.10898475511E-05
 .000205627705628
 .000506160075816
 .000536580813736
 .000628015471111
 .000249490265578
 .000325261611279
 6.70893294422E-05
 7.30157957505E-05
 5.61179824463E-05
 .000368091211114
 .000225225225225
 .000302221851682

 45
 62
 44
 46
 42
 28
 9
 6
 19
 47
 46
 63
 29
 29
 6
 6
 5
 39
 21
 29

 .000493204734765
 .000685711757745
 .000437558423994
 .000513312652041
 .000448641257905
 .000509266837635
 .00012012012012
 8.10898475511E-05
 .000205627705628
 .000506160075816
 .000536580813736
 .000628015471111
 .000249490265578
 .000325261611279
 6.70893294422E-05
 7.30157957505E-05
 5.61179824463E-05
 .000368091211114
 .000225225225225
 .000302221851682

 45
 62
 44
 46
 42
 28
 9
 6
 19
 47
 46
 63
 29
 29
 6
 6
 5
 39
 21
 29

 .0027071459886
 .00199077607087
 .000338113327632
 .000435199857165
 .0014206973167
 .000963969371237
 .00044044044044
 .000459509136123
 .00122294372294
 .00160463513397
 .00114315042926
 .000947007456438
 .00075707390934
 .000482284458103
 .000100633994163
 6.08464964587E-05
 .000505061842017
 .000386967683479
 .000740025740026
 .00079202968027

 247
 180
 34
 39
 133
 53
 33
 34
 113
 149
 98
 95
 88
 43
 9
 5
 45
 41
 69
 76

 .0027071459886
 .00199077607087
 .000338113327632
 .000435199857165
 .0014206973167
 .000963969371237
 .00044044044044
 .000459509136123
 .00122294372294
 .00160463513397
 .00114315042926
 .000947007456438
 .00075707390934
 .000482284458103
 .000100633994163
 6.08464964587E-05
 .000505061842017
 .000386967683479
 .000740025740026
 .00079202968027

 247
 180
 34
 39
 133
 53
 33
 34
 113
 149
 98
 95
 88
 43
 9
 5
 45
 41
 69
 76

 .0027071459886
 .00199077607087
 .000338113327632
 .000435199857165
 .0014206973167
 .000963969371237
 .00044044044044
 .000459509136123
 .00122294372294
 .00160463513397
 .00114315042926
 .000947007456438
 .00075707390934
 .000482284458103
 .000100633994163
 6.08464964587E-05
 .000505061842017
 .000386967683479
 .000740025740026
 .00079202968027

 247
 180
 34
 39
 133
 53
 33
 34
 113
 149
 98
 95
 88
 43
 9
 5
 45
 41
 69
 76

 .0027071459886
 .00199077607087
 .000338113327632
 .000435199857165
 .0014206973167
 .000963969371237
 .00044044044044
 .000459509136123
 .00122294372294
 .00160463513397
 .00114315042926
 .000947007456438
 .00075707390934
 .000482284458103
 .000100633994163
 6.08464964587E-05
 .000505061842017
 .000386967683479
 .000740025740026
 .00079202968027

 247
 180
 34
 39
 133
 53
 33
 34
 113
 149
 98
 95
 88
 43
 9
 5
 45
 41
 69
 76

 5.48005260851E-05
 2.21197341208E-05
 0
 0
 0
 3.63762026882E-05
 1.33466800133E-05
 0
 1.08225108225E-05
 0
 8.16536020903E-05
 .000089716495873
 1.72062252123E-05
 0
 0
 0
 0
 2.83147085473E-05
 .000010725010725
 1.04214431614E-05

 5
 2
 2
 2
 2
 2
 1
 1
 1
 1
 7
 9
 2
 2
 2
 2
 2
 3
 1
 1

 5.48005260851E-05
 2.21197341208E-05
 0
 0
 0
 3.63762026882E-05
 1.33466800133E-05
 0
 1.08225108225E-05
 0
 8.16536020903E-05
 .000089716495873
 1.72062252123E-05
 0
 0
 0
 0
 2.83147085473E-05
 .000010725010725
 1.04214431614E-05

 5
 2
 2
 2
 2
 2
 1
 1
 1
 1
 7
 9
 2
 2
 2
 2
 2
 3
 1
 1

 5.48005260851E-05
 2.21197341208E-05
 0
 0
 0
 3.63762026882E-05
 1.33466800133E-05
 0
 1.08225108225E-05
 0
 8.16536020903E-05
 .000089716495873
 1.72062252123E-05
 0
 0
 0
 0
 2.83147085473E-05
 .000010725010725
 1.04214431614E-05

 5
 2
 2
 2
 2
 2
 1
 1
 1
 1
 7
 9
 2
 2
 2
 2
 2
 3
 1
 1

 5.48005260851E-05
 2.21197341208E-05
 0
 0
 0
 3.63762026882E-05
 1.33466800133E-05
 0
 1.08225108225E-05
 0
 8.16536020903E-05
 .000089716495873
 1.72062252123E-05
 0
 0
 0
 0
 2.83147085473E-05
 .000010725010725
 1.04214431614E-05

 5
 2
 2
 2
 2
 2
 1
 1
 1
 1
 7
 9
 2
 2
 2
 2
 2
 3
 1
 1

 0
 0
 .000517114501084
 .000524471622738
 0
 0
 0
 0
 0
 0
 2.33296005972E-05
 9.96849954145E-06
 3.44124504246E-05
 0
 .000335446647211
 .00027989388371
 .00142539675414
 .0010193295077
 .00027885027885
 .000323064738005

 0
 0
 52
 47
 47
 47
 47
 47
 47
 47
 2
 1
 4
 4
 30
 23
 127
 108
 26
 31

 0
 0
 .000517114501084
 .000524471622738
 0
 0
 0
 0
 0
 0
 2.33296005972E-05
 9.96849954145E-06
 3.44124504246E-05
 0
 .000335446647211
 .00027989388371
 .00142539675414
 .0010193295077
 .00027885027885
 .000323064738005

 0
 0
 52
 47
 47
 47
 47
 47
 47
 47
 2
 1
 4
 4
 30
 23
 127
 108
 26
 31

 0
 0
 .000517114501084
 .000524471622738
 0
 0
 0
 0
 0
 0
 2.33296005972E-05
 9.96849954145E-06
 3.44124504246E-05
 0
 .000335446647211
 .00027989388371
 .00142539675414
 .0010193295077
 .00027885027885
 .000323064738005

 0
 0
 52
 47
 47
 47
 47
 47
 47
 47
 2
 1
 4
 4
 30
 23
 127
 108
 26
 31

 0
 0
 .000517114501084
 .000524471622738
 0
 0
 0
 0
 0
 0
 2.33296005972E-05
 9.96849954145E-06
 3.44124504246E-05
 0
 .000335446647211
 .00027989388371
 .00142539675414
 .0010193295077
 .00027885027885
 .000323064738005

 0
 0
 52
 47
 47
 47
 47
 47
 47
 47
 2
 1
 4
 4
 30
 23
 127
 108
 26
 31

 1.3152126260402E-03
 2.3004523485624E-03
 4.19658306649216E-03
 .003559711652195
 2.5850282003075E-03
 .003237482039252
 1.0944277610945E-03
 1.2568926370414E-03
 1.8506493506487E-03
 2.2077194796274E-03
 5.1675065322886E-03
 5.0939032656809E-03
 1.13561086401028E-03
 5.944436344067E-04
 2.9072042758316E-03
 2.1417966753458E-03
 5.9372825428186E-03
 5.5874358199865E-03
 4.5903045903121E-03
 5.1898786944076E-03

 23.7833333333305
 55.6057692307623
 142.53554502375
 95.8526645767868
 88.6528925619864
 49.0786516854021
 33.5609756097444
 40.4408602150565
 58.228070175435
 58.9024390244151
 132.521444695298
 194.855185910018
 30.0757575757543
 13.0754716981178
 147.892307692332
 119.579545454534
 195.688090737212
 183.18243243236
 76.0934579439825
 83.650602409665

 .000460324419114
 9.622084342544E-04
 .002287237216336
 .001874707077017
 .00159160827209
 .001818810134412
 7.340674007337E-04
 8.379284246944E-04
 9.956709956705E-04
 1.0553976048957E-03
 .002974524076149
 .003379321344552
 .000258093378184
 2.131024349762E-04
 .002169221651967
 1.7645483973017E-03
 .00347931491167
 .002548323769249
 .00142642642643
 .001552795031058

 36.4285714285714
 85.0229885057467
 198.513043478273
 131.250000000008
 130.342281879206
 74.4999999999923
 45.9090909090909
 54.5161290322568
 90.0217391304353
 92.1836734693953
 182.184313725515
 263.908554572302
 15.2666666666625
 12.6842105263212
 192.010309278355
 143.013793103451
 283.264516129001
 247.066666666652
 91.1654135338901
 97.6174496644446

 .000427444103463
 .000951148567194
 .00211818055252
 .00164036869239
 .00148478892497
 .00154598861425
 .000667334000667
 .000783868526327
 .000984848484848
 .00102308951495
 .00246127286301
 .00295067586427
 .00011184046388
 .000168238764455
 .00215804009706
 .00175237909801
 .00332218456082
 .00243506493506
 .00114757614758
 .00120888740673

 39
 86
 213
 147
 139
 85
 50
 58
 91
 95
 211
 296
 13
 15
 193
 144
 296
 258
 107
 116

 .000427444103463
 .000951148567194
 .00211818055252
 .00164036869239
 .00148478892497
 .00154598861425
 .000667334000667
 .000783868526327
 .000984848484848
 .00102308951495
 .00246127286301
 .00295067586427
 .00011184046388
 .000168238764455
 .00215804009706
 .00175237909801
 .00332218456082
 .00243506493506
 .00114757614758
 .00120888740673

 39
 86
 213
 147
 139
 85
 50
 58
 91
 95
 211
 296
 13
 15
 193
 144
 296
 258
 107
 116

 .000427444103463
 .000951148567194
 .00211818055252
 .00164036869239
 .00148478892497
 .00154598861425
 .000667334000667
 .000783868526327
 .000984848484848
 .00102308951495
 .00246127286301
 .00295067586427
 .00011184046388
 .000168238764455
 .00215804009706
 .00175237909801
 .00332218456082
 .00243506493506
 .00114757614758
 .00120888740673

 39
 86
 213
 147
 139
 85
 50
 58
 91
 95
 211
 296
 13
 15
 193
 144
 296
 258
 107
 116

 .000427444103463
 .000951148567194
 .00211818055252
 .00164036869239
 .00148478892497
 .00154598861425
 .000667334000667
 .000783868526327
 .000984848484848
 .00102308951495
 .00246127286301
 .00295067586427
 .00011184046388
 .000168238764455
 .00215804009706
 .00175237909801
 .00332218456082
 .00243506493506
 .00114757614758
 .00120888740673

 39
 86
 213
 147
 139
 85
 50
 58
 91
 95
 211
 296
 13
 15
 193
 144
 296
 258
 107
 116

 .000032880315651
 1.10598670604E-05
 .000169056663816
 .000234338384627
 .00010681934712
 .000272821520162
 6.67334000667E-05
 5.40598983674E-05
 1.08225108225E-05
 3.23080899457E-05
 .000513251213139
 .000428645480282
 .000146252914304
 4.48636705212E-05
 .000011181554907
 1.21692992917E-05
 .00015713035085
 .000113258834189
 .00027885027885
 .000343907624328

 3
 1
 17
 21
 10
 15
 5
 4
 1
 3
 44
 43
 17
 4
 1
 1
 14
 12
 26
 33

 .000032880315651
 1.10598670604E-05
 .000169056663816
 .000234338384627
 .00010681934712
 .000272821520162
 6.67334000667E-05
 5.40598983674E-05
 1.08225108225E-05
 3.23080899457E-05
 .000513251213139
 .000428645480282
 .000146252914304
 4.48636705212E-05
 .000011181554907
 1.21692992917E-05
 .00015713035085
 .000113258834189
 .00027885027885
 .000343907624328

 3
 1
 17
 21
 10
 15
 5
 4
 1
 3
 44
 43
 17
 4
 1
 1
 14
 12
 26
 33

 .000032880315651
 1.10598670604E-05
 .000169056663816
 .000234338384627
 .00010681934712
 .000272821520162
 6.67334000667E-05
 5.40598983674E-05
 1.08225108225E-05
 3.23080899457E-05
 .000513251213139
 .000428645480282
 .000146252914304
 4.48636705212E-05
 .000011181554907
 1.21692992917E-05
 .00015713035085
 .000113258834189
 .00027885027885
 .000343907624328

 3
 1
 17
 21
 10
 15
 5
 4
 1
 3
 44
 43
 17
 4
 1
 1
 14
 12
 26
 33

 .000032880315651
 1.10598670604E-05
 .000169056663816
 .000234338384627
 .00010681934712
 .000272821520162
 6.67334000667E-05
 5.40598983674E-05
 1.08225108225E-05
 3.23080899457E-05
 .000513251213139
 .000428645480282
 .000146252914304
 4.48636705212E-05
 .000011181554907
 1.21692992917E-05
 .00015713035085
 .000113258834189
 .00027885027885
 .000343907624328

 3
 1
 17
 21
 10
 15
 5
 4
 1
 3
 44
 43
 17
 4
 1
 1
 14
 12
 26
 33

 5.699254712841E-04
 8.516097636502E-04
 1.09389605998516E-03
 8.257638315439E-04
 4.700051273283E-04
 4.910787362909E-04
 8.00800800801E-05
 1.621796951022E-04
 4.761904761906E-04
 7.215473421219E-04
 1.3064576334446E-03
 .000847322461023
 6.1942410764174E-04
 2.916138583881E-04
 4.248990864675E-04
 2.190473872511E-04
 1.9080114031756E-03
 2.3595590456025E-03
 1.9948519948561E-03
 2.1259744049363E-03

 19.8076923076897
 40.3246753246657
 70.6181818182018
 39.1621621621599
 14.1363636363589
 7.81481481481367
 3.33333333333333
 4.66666666666667
 25.8181818181852
 29.537313432836
 64.2857142857226
 33.8470588235143
 39.2499999999975
 14.9230769230811
 13.7368421052695
 7.77777777777783
 82.505882352956
 154.775999999978
 75.6344086022064
 78.117647058824

 .000230162209557
 1.437782717852E-04
 2.0883470236046E-04
 1.897025018412E-04
 1.709109553922E-04
 2.000691147854E-04
 5.33867200534E-05
 5.40598983674E-05
 9.74025974026E-05
 2.369259929357E-04
 2.566256065693E-04
 2.492124885365E-04
 1.118404638798E-04
 6.72955057818E-05
 3.019019824902E-04
 1.703701900842E-04
 4.713910525486E-04
 4.719118091207E-04
 7.400257400261E-04
 8.441368960769E-04

 15.6666666666667
 6.84615384615384
 17.2857142857112
 10.4117647058803
 6.37500000000073
 5.90909090909255
 4
 4
 5.00000000000103
 16.6363636363694
 11.9090909090886
 11.0800000000039
 6.53846153846181
 4.33333333333333
 16.6296296296344
 9.28571428571362
 38.1428571428493
 42.6399999999961
 48.2463768116006
 61.2962962962944

 .000197281893906
 8.84789364832E-05
 .000188945683088
 .000145066619055
 7.47735429841E-05
 .000127316709409
 5.33867200534E-05
 5.40598983674E-05
 6.49350649351E-05
 .00020461790299
 .000174972004479
 .000149527493122
 5.16186756368E-05
 1.12159176303E-05
 7.82708843492E-05
 3.65078978752E-05
 .00044894385957
 .000434158864391
 .000611325611326
 .000729501021301

 18
 8
 19
 13
 7
 7
 4
 4
 6
 19
 15
 15
 6
 1
 7
 3
 40
 46
 57
 70

 .000197281893906
 8.84789364832E-05
 .000188945683088
 .000145066619055
 7.47735429841E-05
 .000127316709409
 5.33867200534E-05
 5.40598983674E-05
 6.49350649351E-05
 .00020461790299
 .000174972004479
 .000149527493122
 5.16186756368E-05
 1.12159176303E-05
 7.82708843492E-05
 3.65078978752E-05
 .00044894385957
 .000434158864391
 .000611325611326
 .000729501021301

 18
 8
 19
 13
 7
 7
 4
 4
 6
 19
 15
 15
 6
 1
 7
 3
 40
 46
 57
 70

 .000197281893906
 8.84789364832E-05
 .000188945683088
 .000145066619055
 7.47735429841E-05
 .000127316709409
 5.33867200534E-05
 5.40598983674E-05
 6.49350649351E-05
 .00020461790299
 .000174972004479
 .000149527493122
 5.16186756368E-05
 1.12159176303E-05
 7.82708843492E-05
 3.65078978752E-05
 .00044894385957
 .000434158864391
 .000611325611326
 .000729501021301

 18
 8
 19
 13
 7
 7
 4
 4
 6
 19
 15
 15
 6
 1
 7
 3
 40
 46
 57
 70

 .000032880315651
 .000055299335302
 1.988901927246E-05
 4.46358827862E-05
 9.61374124081E-05
 7.27524053764E-05
 0
 0
 3.24675324675E-05
 3.23080899457E-05
 8.16536020903E-05
 9.96849954145E-05
 .000060221788243
 5.60795881515E-05
 .000223631098141
 .000133862292209
 2.24471929786E-05
 3.77529447297E-05
 1.287001287001E-04
 1.146358747759E-04

 1.66666666666667
 5
 1
 2
 5.88888888889005
 4
 4
 4
 3
 1.6666666666677
 5.28571428571516
 5.2
 7
 5
 20
 11
 1
 4
 6.6666666666677
 5.90909090909043

 .000032880315651
 .000055299335302
 1.988901927246E-05
 4.46358827862E-05
 9.61374124081E-05
 7.27524053764E-05
 0
 0
 3.24675324675E-05
 3.23080899457E-05
 8.16536020903E-05
 9.96849954145E-05
 .000060221788243
 5.60795881515E-05
 .000223631098141
 .000133862292209
 2.24471929786E-05
 3.77529447297E-05
 1.287001287001E-04
 1.146358747759E-04

 1.66666666666667
 5
 1
 2
 5.88888888889005
 4
 4
 4
 3
 1.6666666666677
 5.28571428571516
 5.2
 7
 5
 20
 11
 1
 4
 6.6666666666677
 5.90909090909043

 .000032880315651
 .000055299335302
 1.988901927246E-05
 4.46358827862E-05
 9.61374124081E-05
 7.27524053764E-05
 0
 0
 3.24675324675E-05
 3.23080899457E-05
 8.16536020903E-05
 9.96849954145E-05
 .000060221788243
 5.60795881515E-05
 .000223631098141
 .000133862292209
 2.24471929786E-05
 3.77529447297E-05
 1.287001287001E-04
 1.146358747759E-04

 1.66666666666667
 5
 1
 2
 5.88888888889005
 4
 4
 4
 3
 1.6666666666677
 5.28571428571516
 5.2
 7
 5
 20
 11
 1
 4
 6.6666666666677
 5.90909090909043

 .000284962735642
 .000597232821261
 .000855227828716
 .00058026647622
 .000235002563664
 .000200069114785
 2.66933600267E-05
 8.10898475511E-05
 .000357142857143
 .00043077453261
 .000968178424785
 .000498424977072
 .000447361855519
 .000213102434976
 8.94524392562E-05
 3.65078978752E-05
 .00123459561382
 .00180270311084
 .00110467610468
 .00107340864563

 26
 54
 86
 52
 22
 11
 2
 6
 33
 40
 83
 50
 52
 19
 8
 3
 110
 191
 103
 103

 .000284962735642
 .000597232821261
 .000855227828716
 .00058026647622
 .000235002563664
 .000200069114785
 2.66933600267E-05
 8.10898475511E-05
 .000357142857143
 .00043077453261
 .000968178424785
 .000498424977072
 .000447361855519
 .000213102434976
 8.94524392562E-05
 3.65078978752E-05
 .00123459561382
 .00180270311084
 .00110467610468
 .00107340864563

 26
 54
 86
 52
 22
 11
 2
 6
 33
 40
 83
 50
 52
 19
 8
 3
 110
 191
 103
 103

 .000284962735642
 .000597232821261
 .000855227828716
 .00058026647622
 .000235002563664
 .000200069114785
 2.66933600267E-05
 8.10898475511E-05
 .000357142857143
 .00043077453261
 .000968178424785
 .000498424977072
 .000447361855519
 .000213102434976
 8.94524392562E-05
 3.65078978752E-05
 .00123459561382
 .00180270311084
 .00110467610468
 .00107340864563

 26
 54
 86
 52
 22
 11
 2
 6
 33
 40
 83
 50
 52
 19
 8
 3
 110
 191
 103
 103

 .000284962735642
 .000597232821261
 .000855227828716
 .00058026647622
 .000235002563664
 .000200069114785
 2.66933600267E-05
 8.10898475511E-05
 .000357142857143
 .00043077453261
 .000968178424785
 .000498424977072
 .000447361855519
 .000213102434976
 8.94524392562E-05
 3.65078978752E-05
 .00123459561382
 .00180270311084
 .00110467610468
 .00107340864563

 26
 54
 86
 52
 22
 11
 2
 6
 33
 40
 83
 50
 52
 19
 8
 3
 110
 191
 103
 103

 5.48005260851E-05
 .000110598670604
 2.98335289087E-05
 1.11589706965E-05
 6.40916082721E-05
 9.09405067205E-05
 0
 2.70299491837E-05
 1.08225108225E-05
 3.23080899457E-05
 8.16536020903E-05
 9.96849954145E-05
 5.16186756368E-05
 1.12159176303E-05
 3.35446647211E-05
 1.21692992917E-05
 .000202024736807
 8.49441256418E-05
 .000117975117975
 .000198007420068

 5
 10
 3
 1
 6
 5
 5
 2
 1
 3
 7
 10
 6
 1
 3
 1
 18
 9
 11
 19

 5.48005260851E-05
 .000110598670604
 2.98335289087E-05
 1.11589706965E-05
 6.40916082721E-05
 9.09405067205E-05
 0
 2.70299491837E-05
 1.08225108225E-05
 3.23080899457E-05
 8.16536020903E-05
 9.96849954145E-05
 5.16186756368E-05
 1.12159176303E-05
 3.35446647211E-05
 1.21692992917E-05
 .000202024736807
 8.49441256418E-05
 .000117975117975
 .000198007420068

 5
 10
 3
 1
 6
 5
 5
 2
 1
 3
 7
 10
 6
 1
 3
 1
 18
 9
 11
 19

 5.48005260851E-05
 .000110598670604
 2.98335289087E-05
 1.11589706965E-05
 6.40916082721E-05
 9.09405067205E-05
 0
 2.70299491837E-05
 1.08225108225E-05
 3.23080899457E-05
 8.16536020903E-05
 9.96849954145E-05
 5.16186756368E-05
 1.12159176303E-05
 3.35446647211E-05
 1.21692992917E-05
 .000202024736807
 8.49441256418E-05
 .000117975117975
 .000198007420068

 5
 10
 3
 1
 6
 5
 5
 2
 1
 3
 7
 10
 6
 1
 3
 1
 18
 9
 11
 19

 5.48005260851E-05
 .000110598670604
 2.98335289087E-05
 1.11589706965E-05
 6.40916082721E-05
 9.09405067205E-05
 0
 2.70299491837E-05
 1.08225108225E-05
 3.23080899457E-05
 8.16536020903E-05
 9.96849954145E-05
 5.16186756368E-05
 1.12159176303E-05
 3.35446647211E-05
 1.21692992917E-05
 .000202024736807
 8.49441256418E-05
 .000117975117975
 .000198007420068

 5
 10
 3
 1
 6
 5
 5
 2
 1
 3
 7
 10
 6
 1
 3
 1
 18
 9
 11
 19

 0
 0
 0
 4.46358827862E-05
 0
 0
 0
 0
 1.08225108225E-05
 2.15387266305E-05
 0
 0
 8.60311260614E-06
 0
 0
 0
 0
 0
 .000032175032175
 1.04214431614E-05

 0
 0
 0
 4
 4
 4
 4
 4
 1
 2
 2
 2
 1
 1
 1
 1
 1
 1
 3
 1

 0
 0
 0
 4.46358827862E-05
 0
 0
 0
 0
 1.08225108225E-05
 2.15387266305E-05
 0
 0
 8.60311260614E-06
 0
 0
 0
 0
 0
 .000032175032175
 1.04214431614E-05

 0
 0
 0
 4
 4
 4
 4
 4
 1
 2
 2
 2
 1
 1
 1
 1
 1
 1
 3
 1

 0
 0
 0
 4.46358827862E-05
 0
 0
 0
 0
 1.08225108225E-05
 2.15387266305E-05
 0
 0
 8.60311260614E-06
 0
 0
 0
 0
 0
 .000032175032175
 1.04214431614E-05

 0
 0
 0
 4
 4
 4
 4
 4
 1
 2
 2
 2
 1
 1
 1
 1
 1
 1
 3
 1

 0
 0
 0
 4.46358827862E-05
 0
 0
 0
 0
 1.08225108225E-05
 2.15387266305E-05
 0
 0
 8.60311260614E-06
 0
 0
 0
 0
 0
 .000032175032175
 1.04214431614E-05

 0
 0
 0
 4
 4
 4
 4
 4
 1
 2
 2
 2
 1
 1
 1
 1
 1
 1
 3
 1

 .000175361683472
 .000353915745933
 .000815449790171
 .000836922802241
 .000395231584345
 .000527454938979
 .000173506840174
 .000243269542653
 .000238095238095
 .000344619626088
 .000828200821202
 .000847322461023
 .00022368092776
 8.97273410424E-05
 .000290720427583
 .000158200890793
 .00044894385957
 .000198202959831
 .000815100815101
 .00105256575931

 16
 32
 82
 75
 37
 29
 13
 18
 22
 32
 71
 85
 26
 8
 26
 13
 40
 21
 76
 101

 .000175361683472
 .000353915745933
 .000815449790171
 .000836922802241
 .000395231584345
 .000527454938979
 .000173506840174
 .000243269542653
 .000238095238095
 .000344619626088
 .000828200821202
 .000847322461023
 .00022368092776
 8.97273410424E-05
 .000290720427583
 .000158200890793
 .00044894385957
 .000198202959831
 .000815100815101
 .00105256575931

 16
 32
 82
 75
 37
 29
 13
 18
 22
 32
 71
 85
 26
 8
 26
 13
 40
 21
 76
 101

 .000175361683472
 .000353915745933
 .000815449790171
 .000836922802241
 .000395231584345
 .000527454938979
 .000173506840174
 .000243269542653
 .000238095238095
 .000344619626088
 .000828200821202
 .000847322461023
 .00022368092776
 8.97273410424E-05
 .000290720427583
 .000158200890793
 .00044894385957
 .000198202959831
 .000815100815101
 .00105256575931

 16
 32
 82
 75
 37
 29
 13
 18
 22
 32
 71
 85
 26
 8
 26
 13
 40
 21
 76
 101

 .000175361683472
 .000353915745933
 .000815449790171
 .000836922802241
 .000395231584345
 .000527454938979
 .000173506840174
 .000243269542653
 .000238095238095
 .000344619626088
 .000828200821202
 .000847322461023
 .00022368092776
 8.97273410424E-05
 .000290720427583
 .000158200890793
 .00044894385957
 .000198202959831
 .000815100815101
 .00105256575931

 16
 32
 82
 75
 37
 29
 13
 18
 22
 32
 71
 85
 26
 8
 26
 13
 40
 21
 76
 101

 .000175361683472
 .000353915745933
 .000815449790171
 .000836922802241
 .000395231584345
 .000527454938979
 .000173506840174
 .000243269542653
 .000238095238095
 .000344619626088
 .000828200821202
 .000847322461023
 .00022368092776
 8.97273410424E-05
 .000290720427583
 .000158200890793
 .00044894385957
 .000198202959831
 .000815100815101
 .00105256575931

 16
 32
 82
 75
 37
 29
 13
 18
 22
 32
 71
 85
 26
 8
 26
 13
 40
 21
 76
 101

 .000054800526085
 6.63592023624E-05
 0
 2.23179413931E-05
 .000010681934712
 0
 0
 0
 3.24675324675E-05
 1.07693633152E-05
 4.66592011944E-05
 1.99369990829E-05
 0
 0
 2.23631098141E-05
 0
 0
 0
 .00006435006435
 6.25286589687E-05

 2.6
 3.33333333333333
 3.33333333333333
 2
 1
 1
 1
 1
 3
 1
 2
 2
 2
 2
 2
 2
 2
 2
 3
 6

 .000054800526085
 6.63592023624E-05
 0
 2.23179413931E-05
 .000010681934712
 0
 0
 0
 3.24675324675E-05
 1.07693633152E-05
 4.66592011944E-05
 1.99369990829E-05
 0
 0
 2.23631098141E-05
 0
 0
 0
 .00006435006435
 6.25286589687E-05

 2.6
 3.33333333333333
 3.33333333333333
 2
 1
 1
 1
 1
 3
 1
 2
 2
 2
 2
 2
 2
 2
 2
 3
 6

 .000054800526085
 6.63592023624E-05
 0
 2.23179413931E-05
 .000010681934712
 0
 0
 0
 3.24675324675E-05
 1.07693633152E-05
 4.66592011944E-05
 1.99369990829E-05
 0
 0
 2.23631098141E-05
 0
 0
 0
 .00006435006435
 6.25286589687E-05

 2.6
 3.33333333333333
 3.33333333333333
 2
 1
 1
 1
 1
 3
 1
 2
 2
 2
 2
 2
 2
 2
 2
 3
 6

 .000032880315651
 4.42394682416E-05
 0
 2.23179413931E-05
 .000010681934712
 0
 0
 0
 3.24675324675E-05
 1.07693633152E-05
 2.33296005972E-05
 0
 0
 0
 0
 0
 0
 0
 .000032175032175
 6.25286589687E-05

 3
 4
 4
 2
 1
 1
 1
 1
 3
 1
 2
 2
 2
 2
 2
 2
 2
 2
 3
 6

 .000032880315651
 4.42394682416E-05
 0
 2.23179413931E-05
 .000010681934712
 0
 0
 0
 3.24675324675E-05
 1.07693633152E-05
 2.33296005972E-05
 0
 0
 0
 0
 0
 0
 0
 .000032175032175
 6.25286589687E-05

 3
 4
 4
 2
 1
 1
 1
 1
 3
 1
 2
 2
 2
 2
 2
 2
 2
 2
 3
 6

 .000021920210434
 2.21197341208E-05
 0
 0
 0
 0
 0
 0
 0
 0
 2.33296005972E-05
 1.99369990829E-05
 0
 0
 2.23631098141E-05
 0
 0
 0
 .000032175032175

 2
 2
 2
 2
 2
 2
 2
 2
 2
 2
 2
 2
 2
 2
 2
 2
 2
 2
 3

 .000021920210434
 2.21197341208E-05
 0
 0
 0
 0
 0
 0
 0
 0
 2.33296005972E-05
 1.99369990829E-05
 0
 0
 2.23631098141E-05
 0
 0
 0
 .000032175032175

 2
 2
 2
 2
 2
 2
 2
 2
 2
 2
 2
 2
 2
 2
 2
 2
 2
 2
 3

 5.48005260851E-05
 4.42394682416E-05
 0
 0
 7.47735429841E-05
 .000381950128226
 9.34267600934E-05
 1.35149745918E-05
 .00004329004329
 7.53855432066E-05
 0
 0
 3.441245042454E-05
 0
 0
 0
 .000101012368403
 .000481350045304
 .000032175032175
 8.33715452916E-05

 5
 4
 4
 4
 3.57142857142914
 11.095238095235
 5.28571428571658
 1
 2.5
 5.28571428571618
 5.28571428571618
 5.28571428571618
 2.49999999999971
 2.49999999999971
 2.49999999999971
 2.49999999999971
 9
 51
 1.66666666666667
 5

 5.48005260851E-05
 4.42394682416E-05
 0
 0
 7.47735429841E-05
 .000381950128226
 9.34267600934E-05
 1.35149745918E-05
 .00004329004329
 7.53855432066E-05
 0
 0
 3.441245042454E-05
 0
 0
 0
 .000101012368403
 .000481350045304
 .000032175032175
 8.33715452916E-05

 5
 4
 4
 4
 3.57142857142914
 11.095238095235
 5.28571428571658
 1
 2.5
 5.28571428571618
 5.28571428571618
 5.28571428571618
 2.49999999999971
 2.49999999999971
 2.49999999999971
 2.49999999999971
 9
 51
 1.66666666666667
 5

 5.48005260851E-05
 4.42394682416E-05
 0
 0
 4.27277388481E-05
 .000145504810753
 1.33466800133E-05
 1.35149745918E-05
 3.24675324675E-05
 6.46161798914E-05
 0
 0
 2.58093378184E-05
 0
 0
 0
 .000101012368403
 .000481350045304
 .00002145002145
 6.25286589687E-05

 5
 4
 4
 4
 4
 8
 1
 1
 3
 6
 6
 6
 3
 3
 3
 3
 9
 51
 2
 6

 5.48005260851E-05
 4.42394682416E-05
 0
 0
 4.27277388481E-05
 .000145504810753
 1.33466800133E-05
 1.35149745918E-05
 3.24675324675E-05
 6.46161798914E-05
 0
 0
 2.58093378184E-05
 0
 0
 0
 .000101012368403
 .000481350045304
 .00002145002145
 6.25286589687E-05

 5
 4
 4
 4
 4
 8
 1
 1
 3
 6
 6
 6
 3
 3
 3
 3
 9
 51
 2
 6

 5.48005260851E-05
 4.42394682416E-05
 0
 0
 4.27277388481E-05
 .000145504810753
 1.33466800133E-05
 1.35149745918E-05
 3.24675324675E-05
 6.46161798914E-05
 0
 0
 2.58093378184E-05
 0
 0
 0
 .000101012368403
 .000481350045304
 .00002145002145
 6.25286589687E-05

 5
 4
 4
 4
 4
 8
 1
 1
 3
 6
 6
 6
 3
 3
 3
 3
 9
 51
 2
 6

 0
 0
 0
 0
 .000032045804136
 .000236445317473
 8.00800800801E-05
 0
 1.08225108225E-05
 1.07693633152E-05
 0
 0
 8.60311260614E-06
 0
 0
 0
 0
 0
 .000010725010725
 2.08428863229E-05

 0
 0
 0
 0
 3
 13
 6
 6
 1
 1
 1
 1
 1
 1
 1
 1
 1
 1
 1
 2

 0
 0
 0
 0
 .000032045804136
 .000236445317473
 8.00800800801E-05
 0
 1.08225108225E-05
 1.07693633152E-05
 0
 0
 8.60311260614E-06
 0
 0
 0
 0
 0
 .000010725010725
 2.08428863229E-05

 0
 0
 0
 0
 3
 13
 6
 6
 1
 1
 1
 1
 1
 1
 1
 1
 1
 1
 1
 2

 0
 0
 0
 0
 .000032045804136
 .000236445317473
 8.00800800801E-05
 0
 1.08225108225E-05
 1.07693633152E-05
 0
 0
 8.60311260614E-06
 0
 0
 0
 0
 0
 .000010725010725
 2.08428863229E-05

 0
 0
 0
 0
 3
 13
 6
 6
 1
 1
 1
 1
 1
 1
 1
 1
 1
 1
 1
 2

 0
 2.21197341208E-05
 0
 0
 4.27277388481E-05
 1.81881013441E-05
 1.33466800133E-05
 0
 6.49350649351E-05
 0
 1.16648002986E-05
 0
 0
 0
 0
 0
 0
 0
 .0002574002574
 .000312643294843

 0
 2
 2
 2
 4
 1
 1
 1
 6
 6
 1
 1
 1
 1
 1
 1
 1
 1
 24
 30

 0
 2.21197341208E-05
 0
 0
 4.27277388481E-05
 1.81881013441E-05
 1.33466800133E-05
 0
 6.49350649351E-05
 0
 1.16648002986E-05
 0
 0
 0
 0
 0
 0
 0
 .0002574002574
 .000312643294843

 0
 2
 2
 2
 4
 1
 1
 1
 6
 6
 1
 1
 1
 1
 1
 1
 1
 1
 24
 30

 0
 2.21197341208E-05
 0
 0
 4.27277388481E-05
 1.81881013441E-05
 1.33466800133E-05
 0
 6.49350649351E-05
 0
 1.16648002986E-05
 0
 0
 0
 0
 0
 0
 0
 .0002574002574
 .000312643294843

 0
 2
 2
 2
 4
 1
 1
 1
 6
 6
 1
 1
 1
 1
 1
 1
 1
 1
 24
 30

 0
 2.21197341208E-05
 0
 0
 4.27277388481E-05
 1.81881013441E-05
 1.33466800133E-05
 0
 6.49350649351E-05
 0
 1.16648002986E-05
 0
 0
 0
 0
 0
 0
 0
 .0002574002574
 .000312643294843

 0
 2
 2
 2
 4
 1
 1
 1
 6
 6
 1
 1
 1
 1
 1
 1
 1
 1
 24
 30

 0
 2.21197341208E-05
 0
 0
 4.27277388481E-05
 1.81881013441E-05
 1.33466800133E-05
 0
 6.49350649351E-05
 0
 1.16648002986E-05
 0
 0
 0
 0
 0
 0
 0
 .0002574002574
 .000312643294843

 0
 2
 2
 2
 4
 1
 1
 1
 6
 6
 1
 1
 1
 1
 1
 1
 1
 1
 24
 30

 1.11464270057031E-02
 3.8598936040832E-03
 6.4042642057323E-03
 .005847300644992
 .004625277730308
 4.8562230588761E-03
 6.4064064064067E-03
 5.9060438966347E-03
 .005898268398268
 5.0831394848002E-03
 5.1208473310861E-03
 5.19358826109575E-03
 1.6173851699553E-03
 1.6823876445454E-03
 .003544552905528
 .002920631830023
 .011863341489145
 5.80451525219212E-03
 2.9601029601031E-03
 3.2098044937266E-03

 276.128810226109
 97.0802292264077
 223.077639751562
 175.568702290117
 131.44803695153
 86.9925093633007
 140.458333333337
 122.441647597265
 163.03669724771
 137.974576271187
 159.487471526175
 193.095969289801
 69.053191489364
 55.906666666663
 120.817034700359
 92.2583333333572
 376.699148533553
 210.391869918729
 80.565217391307
 90.7792207792329

 1.11464270057031E-02
 3.8488337370228E-03
 .00630481910937
 5.7915057915093E-03
 .004603913860884
 4.8562230588761E-03
 6.3930597263934E-03
 5.8925289220429E-03
 .005898268398268
 .005072370121485
 5.1091825307875E-03
 5.1836197615543E-03
 1.5743696069246E-03
 1.6150921387636E-03
 .003253832477945
 .002665076544896
 .011863341489145
 5.80451525219212E-03
 .002906477906478
 .003126432948435

 276.128810226109
 97.356321839127
 226.438485804426
 177.211946050136
 132.048723897939
 86.9925093633007
 140.749478079334
 122.720183486248
 163.03669724771
 138.265392781315
 159.849315068472
 193.465384615358
 70.8032786885259
 57.9861111111068
 129.28865979387
 99.091324200939
 376.699148533553
 210.391869918729
 81.9594095940998
 92.9866666666791

 .00266330556773
 .000663592023624
 .00306290896796
 .00249960943603
 .00178388309691
 .00187337443844
 .00226893560227
 .00190561141745
 .00146103896104
 .00124924614457
 .00199468085106
 .00243231388811
 .000653836558067
 .000527148128624
 .00100633994163
 .000791004453964
 .00248041482413
 .00163281485956
 .000879450879451
 .00106298720247

 243
 60
 308
 224
 167
 103
 170
 141
 135
 116
 171
 244
 76
 47
 90
 65
 221
 173
 82
 102

 .00266330556773
 .000663592023624
 .00306290896796
 .00249960943603
 .00178388309691
 .00187337443844
 .00226893560227
 .00190561141745
 .00146103896104
 .00124924614457
 .00199468085106
 .00243231388811
 .000653836558067
 .000527148128624
 .00100633994163
 .000791004453964
 .00248041482413
 .00163281485956
 .000879450879451
 .00106298720247

 243
 60
 308
 224
 167
 103
 170
 141
 135
 116
 171
 244
 76
 47
 90
 65
 221
 173
 82
 102

 .00266330556773
 .000663592023624
 .00306290896796
 .00249960943603
 .00178388309691
 .00187337443844
 .00226893560227
 .00190561141745
 .00146103896104
 .00124924614457
 .00199468085106
 .00243231388811
 .000653836558067
 .000527148128624
 .00100633994163
 .000791004453964
 .00248041482413
 .00163281485956
 .000879450879451
 .00106298720247

 243
 60
 308
 224
 167
 103
 170
 141
 135
 116
 171
 244
 76
 47
 90
 65
 221
 173
 82
 102

 .00266330556773
 .000663592023624
 .00306290896796
 .00249960943603
 .00178388309691
 .00187337443844
 .00226893560227
 .00190561141745
 .00146103896104
 .00124924614457
 .00199468085106
 .00243231388811
 .000653836558067
 .000527148128624
 .00100633994163
 .000791004453964
 .00248041482413
 .00163281485956
 .000879450879451
 .00106298720247

 243
 60
 308
 224
 167
 103
 170
 141
 135
 116
 171
 244
 76
 47
 90
 65
 221
 173
 82
 102

 .0019947391495
 .000774190694228
 .00168062212852
 .001618050751
 .00158092633738
 .00189156253979
 .00158825492159
 .00140555735755
 .00147186147186
 .00138924786767
 .00225130645763
 .00189401491288
 .000705455233704
 .000852409739903
 .00188968277929
 .00158200890793
 .00558935105165
 .00248225611598
 .000954525954526
 .000917086998208

 182
 70
 169
 145
 148
 104
 119
 104
 136
 129
 193
 190
 82
 76
 169
 130
 498
 263
 89
 88

 .0019947391495
 .000774190694228
 .00168062212852
 .001618050751
 .00158092633738
 .00189156253979
 .00158825492159
 .00140555735755
 .00147186147186
 .00138924786767
 .00225130645763
 .00189401491288
 .000705455233704
 .000852409739903
 .00188968277929
 .00158200890793
 .00558935105165
 .00248225611598
 .000954525954526
 .000917086998208

 182
 70
 169
 145
 148
 104
 119
 104
 136
 129
 193
 190
 82
 76
 169
 130
 498
 263
 89
 88

 .0019947391495
 .000774190694228
 .00168062212852
 .001618050751
 .00158092633738
 .00189156253979
 .00158825492159
 .00140555735755
 .00147186147186
 .00138924786767
 .00225130645763
 .00189401491288
 .000705455233704
 .000852409739903
 .00188968277929
 .00158200890793
 .00558935105165
 .00248225611598
 .000954525954526
 .000917086998208

 182
 70
 169
 145
 148
 104
 119
 104
 136
 129
 193
 190
 82
 76
 169
 130
 498
 263
 89
 88

 .0019947391495
 .000774190694228
 .00168062212852
 .001618050751
 .00158092633738
 .00189156253979
 .00158825492159
 .00140555735755
 .00147186147186
 .00138924786767
 .00225130645763
 .00189401491288
 .000705455233704
 .000852409739903
 .00188968277929
 .00158200890793
 .00558935105165
 .00248225611598
 .000954525954526
 .000917086998208

 182
 70
 169
 145
 148
 104
 119
 104
 136
 129
 193
 190
 82
 76
 169
 130
 498
 263
 89
 88

 .0042525208242
 .00154838138846
 .00140217585871
 .0016068917803
 .000769099299265
 .000636583547044
 .00204204204204
 .00197318629041
 .00241341991342
 .00197079348669
 .00067655841732
 .00068782646836
 .000180665364729
 .000213102434976
 .000346628202118
 .000292063183002
 .00356910368358
 .00164225309574
 .000922350922351
 .00102130142982

 388
 140
 141
 144
 72
 35
 153
 146
 223
 183
 58
 69
 21
 19
 31
 24
 318
 174
 86
 98

 .0042525208242
 .00154838138846
 .00140217585871
 .0016068917803
 .000769099299265
 .000636583547044
 .00204204204204
 .00197318629041
 .00241341991342
 .00197079348669
 .00067655841732
 .00068782646836
 .000180665364729
 .000213102434976
 .000346628202118
 .000292063183002
 .00356910368358
 .00164225309574
 .000922350922351
 .00102130142982

 388
 140
 141
 144
 72
 35
 153
 146
 223
 183
 58
 69
 21
 19
 31
 24
 318
 174
 86
 98

 .0042525208242
 .00154838138846
 .00140217585871
 .0016068917803
 .000769099299265
 .000636583547044
 .00204204204204
 .00197318629041
 .00241341991342
 .00197079348669
 .00067655841732
 .00068782646836
 .000180665364729
 .000213102434976
 .000346628202118
 .000292063183002
 .00356910368358
 .00164225309574
 .000922350922351
 .00102130142982

 388
 140
 141
 144
 72
 35
 153
 146
 223
 183
 58
 69
 21
 19
 31
 24
 318
 174
 86
 98

 .0042525208242
 .00154838138846
 .00140217585871
 .0016068917803
 .000769099299265
 .000636583547044
 .00204204204204
 .00197318629041
 .00241341991342
 .00197079348669
 .00067655841732
 .00068782646836
 .000180665364729
 .000213102434976
 .000346628202118
 .000292063183002
 .00356910368358
 .00164225309574
 .000922350922351
 .00102130142982

 388
 140
 141
 144
 72
 35
 153
 146
 223
 183
 58
 69
 21
 19
 31
 24
 318
 174
 86
 98

 .00213722051732
 .00084054989659
 .00015911215418
 6.69538241793E-05
 .000470005127329
 .000436514432258
 .0004004004004
 .00051356903449
 .000530303030303
 .000420005169294
 .000139977603583
 .00013955899358
 3.44124504246E-05
 2.24318352606E-05
 .000011181554907
 0
 .000101012368403
 3.77529447297E-05
 .000139425139425
 .000125057317937

 195
 76
 16
 6
 44
 24
 30
 38
 49
 39
 12
 14
 4
 2
 1
 1
 9
 4
 13
 12

 .00213722051732
 .00084054989659
 .00015911215418
 6.69538241793E-05
 .000470005127329
 .000436514432258
 .0004004004004
 .00051356903449
 .000530303030303
 .000420005169294
 .000139977603583
 .00013955899358
 3.44124504246E-05
 2.24318352606E-05
 .000011181554907
 0
 .000101012368403
 3.77529447297E-05
 .000139425139425
 .000125057317937

 195
 76
 16
 6
 44
 24
 30
 38
 49
 39
 12
 14
 4
 2
 1
 1
 9
 4
 13
 12

 .00213722051732
 .00084054989659
 .00015911215418
 6.69538241793E-05
 .000470005127329
 .000436514432258
 .0004004004004
 .00051356903449
 .000530303030303
 .000420005169294
 .000139977603583
 .00013955899358
 3.44124504246E-05
 2.24318352606E-05
 .000011181554907
 0
 .000101012368403
 3.77529447297E-05
 .000139425139425
 .000125057317937

 195
 76
 16
 6
 44
 24
 30
 38
 49
 39
 12
 14
 4
 2
 1
 1
 9
 4
 13
 12

 .00213722051732
 .00084054989659
 .00015911215418
 6.69538241793E-05
 .000470005127329
 .000436514432258
 .0004004004004
 .00051356903449
 .000530303030303
 .000420005169294
 .000139977603583
 .00013955899358
 3.44124504246E-05
 2.24318352606E-05
 .000011181554907
 0
 .000101012368403
 3.77529447297E-05
 .000139425139425
 .000125057317937

 195
 76
 16
 6
 44
 24
 30
 38
 49
 39
 12
 14
 4
 2
 1
 1
 9
 4
 13
 12

 9.86409469531E-05
 2.21197341208E-05
 0
 0
 0
 1.81881013441E-05
 9.34267600934E-05
 9.46048221429E-05
 .000021645021645
 .000043077453261
 4.66592011945E-05
 2.99054986243E-05
 0
 0
 0
 0
 .000123459561382
 9.43823618242E-06
 .000010725010725

 9
 2
 2
 2
 2
 1
 7
 7
 2
 4
 4
 3
 3
 3
 3
 3
 11
 1
 1

 9.86409469531E-05
 2.21197341208E-05
 0
 0
 0
 1.81881013441E-05
 9.34267600934E-05
 9.46048221429E-05
 .000021645021645
 .000043077453261
 4.66592011945E-05
 2.99054986243E-05
 0
 0
 0
 0
 .000123459561382
 9.43823618242E-06
 .000010725010725

 9
 2
 2
 2
 2
 1
 7
 7
 2
 4
 4
 3
 3
 3
 3
 3
 11
 1
 1

 9.86409469531E-05
 2.21197341208E-05
 0
 0
 0
 1.81881013441E-05
 9.34267600934E-05
 9.46048221429E-05
 .000021645021645
 .000043077453261
 4.66592011945E-05
 2.99054986243E-05
 0
 0
 0
 0
 .000123459561382
 9.43823618242E-06
 .000010725010725

 9
 2
 2
 2
 2
 1
 7
 7
 2
 4
 4
 3
 3
 3
 3
 3
 11
 1
 1

 9.86409469531E-05
 2.21197341208E-05
 0
 0
 0
 1.81881013441E-05
 9.34267600934E-05
 9.46048221429E-05
 .000021645021645
 .000043077453261
 4.66592011945E-05
 2.99054986243E-05
 0
 0
 0
 0
 .000123459561382
 9.43823618242E-06
 .000010725010725

 9
 2
 2
 2
 2
 1
 7
 7
 2
 4
 4
 3
 3
 3
 3
 3
 11
 1
 1

 0
 1.10598670604E-05
 9.94450963623E-05
 5.57948534827E-05
 .000021363869424
 0
 1.33466800133E-05
 1.35149745918E-05
 0
 1.07693633152E-05
 1.16648002986E-05
 9.96849954145E-06
 4.30155630307E-05
 6.72955057818E-05
 .000290720427583
 .000255555285127
 0
 0
 5.36250536251E-05
 8.33715452916E-05

 0
 1
 10
 5
 2
 2
 1
 1
 1
 1
 1
 1
 5
 6
 26
 21
 21
 21
 5
 8

 0
 1.10598670604E-05
 9.94450963623E-05
 5.57948534827E-05
 .000021363869424
 0
 1.33466800133E-05
 1.35149745918E-05
 0
 1.07693633152E-05
 1.16648002986E-05
 9.96849954145E-06
 4.30155630307E-05
 6.72955057818E-05
 .000290720427583
 .000255555285127
 0
 0
 5.36250536251E-05
 8.33715452916E-05

 0
 1
 10
 5
 2
 2
 1
 1
 1
 1
 1
 1
 5
 6
 26
 21
 21
 21
 5
 8

 0
 1.10598670604E-05
 9.94450963623E-05
 5.57948534827E-05
 .000021363869424
 0
 1.33466800133E-05
 1.35149745918E-05
 0
 1.07693633152E-05
 1.16648002986E-05
 9.96849954145E-06
 4.30155630307E-05
 6.72955057818E-05
 .000290720427583
 .000255555285127
 0
 0
 5.36250536251E-05
 8.33715452916E-05

 0
 1
 10
 5
 2
 2
 1
 1
 1
 1
 1
 1
 5
 6
 26
 21
 21
 21
 5
 8

 0
 1.10598670604E-05
 9.94450963623E-05
 5.57948534827E-05
 .000021363869424
 0
 1.33466800133E-05
 1.35149745918E-05
 0
 1.07693633152E-05
 1.16648002986E-05
 9.96849954145E-06
 4.30155630307E-05
 6.72955057818E-05
 .000290720427583
 .000255555285127
 0
 0
 5.36250536251E-05
 8.33715452916E-05

 0
 1
 10
 5
 2
 2
 1
 1
 1
 1
 1
 1
 5
 6
 26
 21
 21
 21
 5
 8

 0
 1.10598670604E-05
 9.94450963623E-05
 5.57948534827E-05
 .000021363869424
 0
 1.33466800133E-05
 1.35149745918E-05
 0
 1.07693633152E-05
 1.16648002986E-05
 9.96849954145E-06
 4.30155630307E-05
 6.72955057818E-05
 .000290720427583
 .000255555285127
 0
 0
 5.36250536251E-05
 8.33715452916E-05

 0
 1
 10
 5
 2
 2
 1
 1
 1
 1
 1
 1
 5
 6
 26
 21
 21
 21
 5
 8

 .00010960105217
 .00026543680945
 .000308279798723
 .000278974267414
 .000459323192617
 .000545643040323
 6.67334000667E-05
 .00014866472051
 .000400432900433
 .000323080899457
 .00033827920866
 .000428645480282
 .000120443576486
 .000112159176303
 .000156541768698
 .000133862292209
 .000168353947339
 .000320900030202
 .000396825396825
 .000625286589687

 10
 24
 31
 25
 43
 30
 5
 11
 37
 30
 29
 43
 14
 10
 14
 11
 15
 34
 37
 60

 .00010960105217
 .00026543680945
 .000308279798723
 .000278974267414
 .000459323192617
 .000545643040323
 6.67334000667E-05
 .00014866472051
 .000400432900433
 .000323080899457
 .00033827920866
 .000428645480282
 .000120443576486
 .000112159176303
 .000156541768698
 .000133862292209
 .000168353947339
 .000320900030202
 .000396825396825
 .000625286589687

 10
 24
 31
 25
 43
 30
 5
 11
 37
 30
 29
 43
 14
 10
 14
 11
 15
 34
 37
 60

 .00010960105217
 .00026543680945
 .000308279798723
 .000278974267414
 .000459323192617
 .000545643040323
 6.67334000667E-05
 .00014866472051
 .000400432900433
 .000323080899457
 .00033827920866
 .000428645480282
 .000120443576486
 .000112159176303
 .000156541768698
 .000133862292209
 .000168353947339
 .000320900030202
 .000396825396825
 .000625286589687

 10
 24
 31
 25
 43
 30
 5
 11
 37
 30
 29
 43
 14
 10
 14
 11
 15
 34
 37
 60

 .00010960105217
 .00026543680945
 .000308279798723
 .000278974267414
 .000459323192617
 .000545643040323
 6.67334000667E-05
 .00014866472051
 .000400432900433
 .000323080899457
 .00033827920866
 .000428645480282
 .000120443576486
 .000112159176303
 .000156541768698
 .000133862292209
 .000168353947339
 .000320900030202
 .000396825396825
 .000625286589687

 10
 24
 31
 25
 43
 30
 5
 11
 37
 30
 29
 43
 14
 10
 14
 11
 15
 34
 37
 60

 .00010960105217
 .00026543680945
 .000308279798723
 .000278974267414
 .000459323192617
 .000545643040323
 6.67334000667E-05
 .00014866472051
 .000400432900433
 .000323080899457
 .00033827920866
 .000428645480282
 .000120443576486
 .000112159176303
 .000156541768698
 .000133862292209
 .000168353947339
 .000320900030202
 .000396825396825
 .000625286589687

 10
 24
 31
 25
 43
 30
 5
 11
 37
 30
 29
 43
 14
 10
 14
 11
 15
 34
 37
 60

 .00010960105217
 .00026543680945
 .000308279798723
 .000278974267414
 .000459323192617
 .000545643040323
 6.67334000667E-05
 .00014866472051
 .000400432900433
 .000323080899457
 .00033827920866
 .000428645480282
 .000120443576486
 .000112159176303
 .000156541768698
 .000133862292209
 .000168353947339
 .000320900030202
 .000396825396825
 .000625286589687

 10
 24
 31
 25
 43
 30
 5
 11
 37
 30
 29
 43
 14
 10
 14
 11
 15
 34
 37
 60

 .000043840420868
 0
 .00220768113924
 .00186354810632
 5.34096735601E-05
 1.81881013441E-05
 .00028028028028
 .000243269542653
 .00004329004329
 6.46161798914E-05
 .000641564016424
 .000468519478448
 1.72062252123E-05
 3.36477528909E-05
 .000335446647211
 .000304232482294
 0
 2.83147085473E-05
 .0003861003861
[truncated: 470,752 more chars]
